# Ni-catalyzed hydroarylation of alkynes with unactivated *β*-C(sp^2^)−H bonds

**DOI:** 10.1038/s41467-022-30367-8

**Published:** 2022-05-26

**Authors:** Shao-Long Qi, Yu-Peng Liu, Yi Li, Yu-Xin Luan, Mengchun Ye

**Affiliations:** 1 State Key Laboratory and Institute of Elemento-Organic Chemistry, College of Chemistry, Nankai University, Tianjin, 300071 China; 2 Haihe Laboratory of Sustainable Chemical Transformations, Tianjin, 300192 China

**Keywords:** Homogeneous catalysis, Synthetic chemistry methodology

## Abstract

Hydroarylation of alkynes with unactivated C(sp^2^)−H bonds via chelated C−H metalation mainly occurs at *γ*-position to the coordinating atom of directing groups via stable 5-membered metallacycles, while *β*-C(sp^2^)−H bond-involved hydroarylation has been a formidable challenge. Herein, we used a phosphine oxide-ligated Ni−Al bimetallic catalyst to enable *β*-C−H bond-involved hydroarylations of alkynes via a rare 7-membered nickelacycle.

## Abstract (web summary)

Directed activation of C–H bonds usually occurs through 5-membered metallacycles, which poses limitations of regiochemistry. Herein, the authors use a phosphine oxide-ligated Ni−Al bimetallic catalyst to enable β-C−H bond-involved hydroarylations of alkynes via a rare 7-membered nickelacycle.

## Introduction

**Fig. 1:**
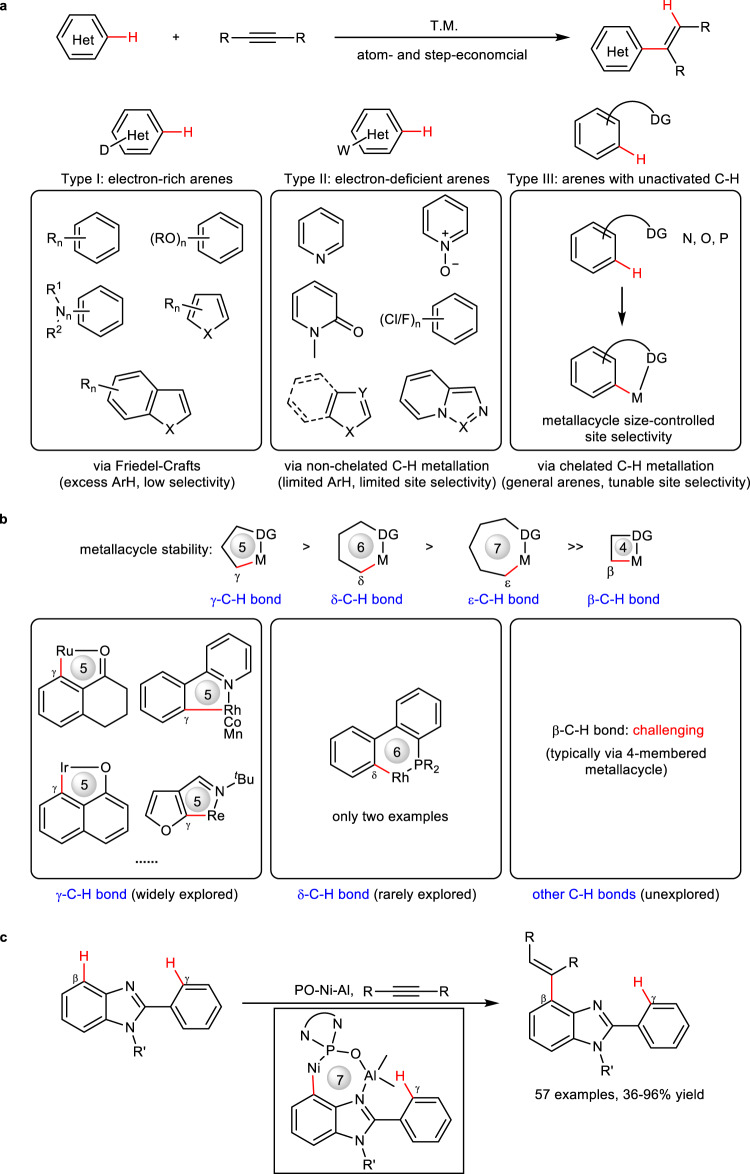
TM-catalyzed hydroarylation of alkynes with (hetero)arenes. **a** Three types of hydroarylations of alkynes and (hetero)arenes: electron-rich arenes (type I), electron-deficient arenes (type II), and general arenes with unactivated C(sp^2^)−H bonds (type III). **b** Stability of metallacycles and reported various metallacycle-involved hydroarylations: *γ*-C−H bond-activation-involved hydroarylation (widely explored); *δ*-C−H bond-activation-involved hydroarylation (rarely explored); *β*-C−H bond-activation-involved hydroarylation (elusive challenge); **c** *β*-C−H bond-involved hydroarylation via 7-membered metallacycles (this work). T.M. = transition metal. Het = heterocycle, D = electron-donating group. W = electron-withdrawing group. DG = directing group. PO = phosphine oxide ligand.

Hydroarylation of alkynes with arenes provides a highly atom- and step-economical route to aromatic alkenes that widely exist in natural products, bioactive compounds, and material molecules^1–8^. During the past several decades, a large number of transition metal-catalyzed hydroarylation reactions have been reported. According to electronic nature of arenes, these hydroarylations can be divided into three types (Fig. 1a): electron-rich (hetero)arenes (Type I), electron-deficient (hetero)arenes (Type II), and general arenes with unactivated C(sp^2^)−H bonds (Type III). Type I reactions mainly proceed via a Friedel-Crafts-type pathway, requiring multiple electron-rich substituents to enhance the electron density of (hetero)arenes^9–11^. This structural requirement results in a limited scope of substrates and difficult site selectivity. Especially in case of less electron-rich arenes, a large excess of substrates is often required for reasonable yields. Instead, most of type II reactions proceed via oxidative addition pathway, because the presence of strong electron-negative heteroatoms or electron-withdrawing groups in substrates leads to uneven distribution of electron density, and then electron-deficient C−H bonds will easily undergo oxidative addition with metal. However, unique electron demand renders substrates limited to special heterocycles such as pyridines, polyfluoroarenes, imidazoles, and other analogs^12–24^. To activate arenes with unactivated C(sp^2^)−H bonds for hydroarylation reactions, chelated C−H metalation has been devised by incorporating proper directing groups in substrates (Type III). With the aid of directing groups, unactivated C(sp^2^)−H bonds can be metallated, and tuning the size of the formed metallacycles would in principle achieve diverse site selectivities. Owing to these advantages, chelated C−H metalation-involved hydroarylation has been widely explored in the past decades by using various metal catalysts such as Ru^25–32^, Ir^33–36^, Rh^37–47^, Co^48–54^, Mn^55–57^, Re^58,59^, and Fe^60^ (Fig. 1b, left). However, most examples are limited to C−H bonds at *γ*-position to the coordinating atoms of directing groups, because the formation of stable 5-membered metallacycles has more favorable entropic effect and ring strain than other larger (6- or 7-membered) or smaller (4-membered) metallacycles^61–65^. Only two examples are reported for *δ*-C−H bond-involved hydroarylations via a 6-membered metallacycle (Fig. 1b, middle)^66,67^. In contrast, there are no reports on other C−H bond-involved hydroarylations, especially very challenging *β*-C−H bond-involved hydroarylations, because a highly strained 4-membered metallacycle is difficult to form (Fig. 1b, right). Here, we show that a phosphine oxide (PO)-ligated Ni−Al bimetallic catalyst can activate an unactivated *β*-C−H bond on the phenyl ring of benzimidazole to undergo hydroarylation via a rare 7-membered nickelacycle, bypassing an unstable 4-membered nickelacycle (Fig. 1c). A series of C4-alkenylated 2-phenylbenzimidazoles, including complex bioactive molecules, can be produced in 41−96% yield, providing a distinctive site selectivity beyond traditional selectivity that generally occurs at C2-phenyl ring via a 5-membered metallacycle.

## Results

### Reaction optimization

**Fig. 2:**
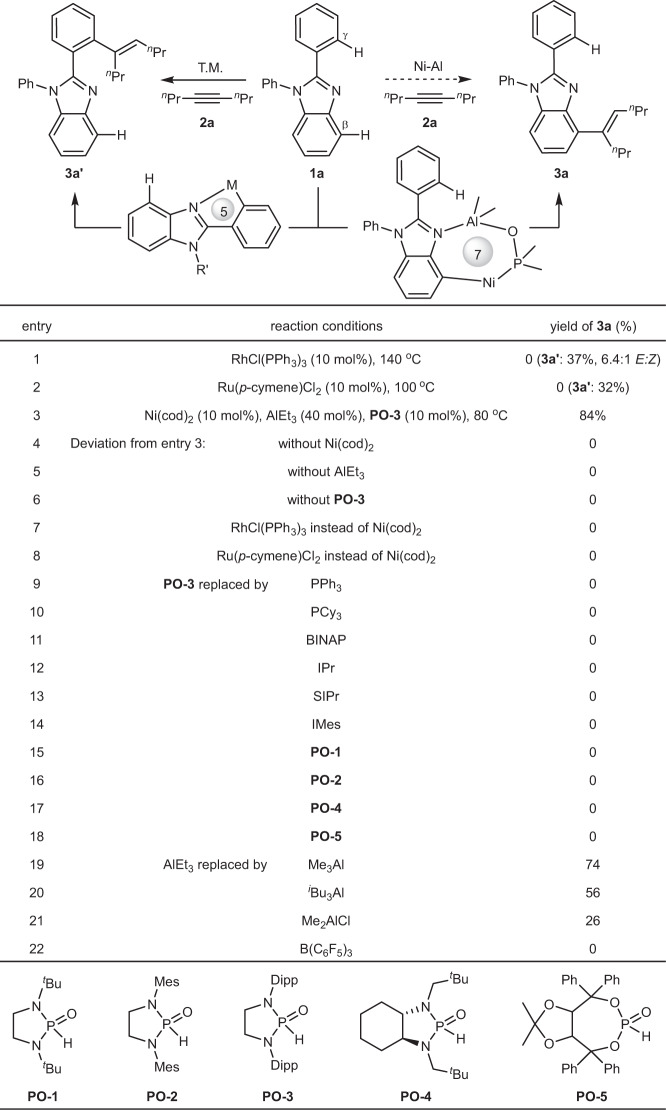
Reaction optimization. Reaction conditions: **1a** (0.2 mmol), **2a** (0.4 mmol), AlEt_3_ (1 mol/L in hexane, 0.08 mmol), toluene (0.4 mL) under N_2_ for 12 h. Yield was determined by ^1^H NMR. cod = 1,5-cyclooctadiene. Et = ethyl. Cy_3_P = triisopropylphosphine. BINAP = 2,2’-bis(diphenylphosphino)-1,1’-binaphthalene. IPr = 1,3-bis(2,6-diisopropylphenyl)-2,3-dihydro-1*H*-imidazole. SIPr = 1,3-bis (2,6-diisopropylphenyl)imidazolidine. IMes = 1,3-dimesityl-2,3-dihydro-1*H*-imidazole. ^*i*^Bu = isobutyl. ^*t*^Bu = *tert*-butyl. Mes = 2,4,6-trimethylphenyl. Dipp = 2,6-diisopropylphenyl.

2-Phenyl benzimidazole (**1a**) bearing both *β*-C−H bond and *γ*-C−H bond was selected as a model substrate, because such a structural motif can be found in a large number of bioactive and material molecules^68,69^. However, due to strong directing ability of imine N atom, transition metal-catalyzed selective C−H activation of 2-phenyl benzimidazoles mainly occurs at *γ*-C−H bond at *ortho*-position of C2-phenyl ring^38,70–76^. For example, with RhCl(PPh_3_)_3_ as a catalyst, the reaction of **1a** and oct-4-yne (**2a**) afforded **3a′** as the sole product in 37% yield with a *E*:*Z* ratio of 6.4:1 (Fig. 2, entry 1). Similarly, the use of [Ru(*p*-cymene)Cl_2_]_2_ as a catalyst also generated **3a′** in 32% yield (entry 2). Given important bioactivity of C4-alkenylated benzimidazoles and derivatives^77,78^, reversing traditional *γ*-C−H bond activation to *β*-C−H bond activation in the hydroarylation reaction (product **3a**) would be highly desirable. We envisioned to use a ligand-ligated Ni−Al bimetallic catalyst for the investigation:^79–81^ firstly, the coordination of Al-Lewis acid with benzimidazole could direct Ni to activate *β*-C−H bonds via a 7-membered nickelacycle, bypassing a highly strained 4-membered nickelacycle; secondly, the coordination of Al-Lewis acid would favor inhibiting the activation of *γ*-C−H bonds on the C2-phenyl ring via a 5-membered nickelacycle. Systematic survey on ligands, Lewis acids, solvents, and temperatures revealed the desired C4-alkenylated product **3a** can be indeed obtained. Under the optimal conditions: Ni(cod)_2_ (10 mol%), **PO-3** (10 mol%), AlEt_3_ (40 mol%) in toluene at 80 ^o^C, **3a** was produced in 84% yield (entry 3). Control experiments showed that the presence of nickel, Al-Lewis acid, and phosphine oxide ligand is essential to the reaction efficiency, the removal of any of them would inactivate the reaction (entries 4−6). Notably, under the optimal conditions, the use of Rh or Ru instead of Ni led to neither **3a** nor **3a′** (entries 7 and 8), suggesting that the PO−Ni−Al bimetallic system is sensitive to transition metals.

Ligand examination showed that various phosphines (entries 9−11), N-heterocyclic carbenes (entries 12−14), and commonly-used phosphine oxides (entries 15−18) were all ineffective, suggesting that the optimal **PO-3** plays a critical role in the reaction. In addition, the selection of Lewis acid proved important. Although AlMe_3_ gave a slightly lower yield than that of AlEt_3_, all other Lewis acids with either bulkier steric hindrance or weaker Lewis acidity led to a big decrease in yield (entries 19−22).

### Scope of benzimidazoles and alkynes

**Fig. 3:**
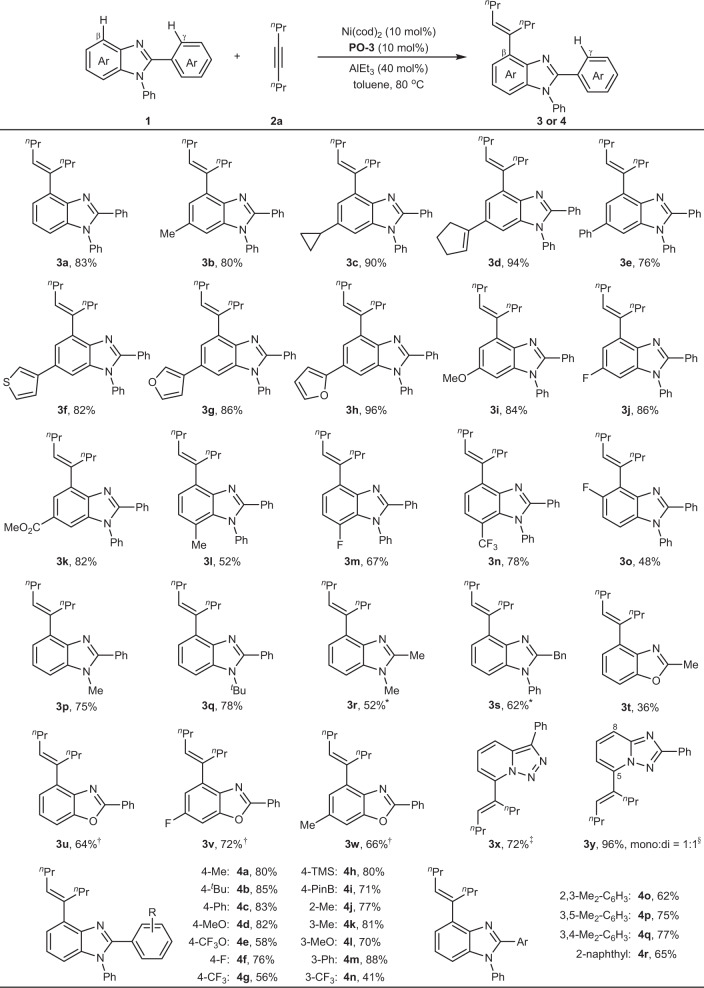
Scope of benzimidazoles and analogs. Reaction conditions: **1** (0.4 mmol), **2a** (0.8 mmol), AlEt_3_ (1 mol/L in hexane, 0.16 mmol), toluene (0.8 mL) under N_2_ for 12 h. Yield of isolated products. *100 °C, AlMe_3_ (1 mol/L in hexane, 0.32 mmol). ^†^**PO-3** was replaced by bulkier **PO-6** (see the structure in the SI). ^‡^30 °C. ^§^mono refers to C5-alkenylated product and di refers to C5 and C8-dialkenylated product. ^*n*^Pr = *n*-propyl.

With the optimized conditions in hand, various C2-aryl benzimidazoles were investigated first (Fig. 3). Results showed that various substituents on C6-position, including alkyl group (**3b** and **3c**), alkenyl group (**3d**), aryl group (**3e**), and heteroaryl group (**3f**, **3g**, and **3h**) did not have a strong influence on the reaction efficiency, providing the corresponding products in 76−96% yield. In addition, the investigation on electronic effect proved that either electron-rich group (**3i**) or electron-deficient group (**3j** and **3k**) were also well-tolerated, delivering 82−86% yield. Given that C7-substituent would have direct influence on the electronic density of the C4−H bond, various substituents with different electronic property were examined. Methyl group provided 52% yield (**3l**), but electron-deficient fluoro group (**3m**) and CF_3_ group (**3n**) increased yield to 67% and 78%, respectively. Different from C6- and C7-positions, C5-position locates closely to the reaction site and substituents on this position would have detrimental effect to the reactivity owing to steric hindrance. For example, C5-methyl afforded trace amount of products and only smaller C5-F group still led to the corresponding product in 48% yield (**3o**). Notably, various N1-substituents are far away from reaction site and can be well compatible with the reaction. For example, methyl group (**3p**) and *tert*-butyl group (**3q**) provided the corresponding products in 75% and 78% yield, respectively. When C2-phenyl group was replaced by alkyl group like methyl group (**3r**) or benzyl group (**3s**), the reaction still proceeded smoothly at a little elevated temperature and loadings of Al-Lewis acid. Beyond benzimidazoles, other heterocycles such as benzoxazoles (**3t**, **3u**, **3v**, **3w**) and triazoles (**3x** and **3y**) also proved to be suitable substrates under modified conditions. To compare the effect of C2-aryl ring, we also prepared a wide range of C2-arylated benzimidazoles and checked their reactivity.

Results showed that either electron-rich groups (**4a** to **4d**) or electron-deficient groups (**4e** to **4i**) at *para* position of C2-phenyl ring can be well-tolerated, providing C4-alkenylated products in 56−85% yield without observing C−H activation on the C2-aryl ring. Similar results appeared for substituents at other positions such as *ortho*- (**4j**), *meta* (**4k** to **4n**), and even multiple sites (**4o** to **4r**). These results showed that the current method can provide high *β*-site selectivity for all examined substrates.

**Fig. 4:**
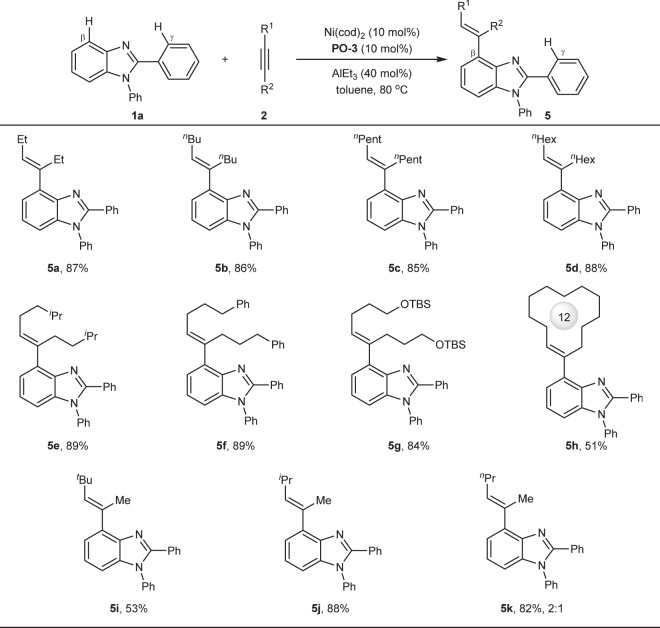
Scope of alkynes. Reaction conditions: **1a** (0.4 mmol), **2** (0.8 mmol), AlEt_3_ (1 mol/L in hexane, 0.16 mmol), toluene (0.8 mL) under N_2_ for 12 h. Yield of isolated products. Et = ethyl. ^*n*^Bu = *n*-butyl. ^*n*^Pent = *n*-pentyl. ^*n*^Hex = *n*-hexyl. ^*i*^Pr = isopropyl. TBS = *tert*-butyldimethylsilyl. ^*t*^Bu = *tert*-butyl.

Next, the scope of alkynes was investigated (Fig. 4). Aryl alkynes were in general ineffective, which may be attributed to their big steric hindrance and strong coordinative ability with nickel, while various alkyl alkynes such as ethyl (**5a**), *n*-butyl (**5b**), *n*-pentyl (**5c**), *n*-hexyl (**5d**) and *i*-hexyl (**5e**) were compatible very well, providing the corresponding products in 85−89% yield. When functional groups such as phenyl group (**5f**) or hydroxyl group (**5g**) were incorporated into the alkyl chain, no significant loss of yields were observed. In addition, cyclic alkyne was still compatible with the reaction, providing 51% yield (**5h**). Notably, besides symmetrical alkynes, non-symmetrical alkynes also displayed good reactivity (**5i**, **5j**, **5k**), but the regioselectivity was highly depending on steric hindrance of substituents of alkynes. For example, *tert*-butyl methyl alkyne (**5i**) and isopropyl methyl alkyne (**5j**) gave one single regioisomer product, while *n*-propyl methyl alkyne (**5k**) delivered a mixture of regioisomers in a ratio of 2:1.

### Synthetic utility

**Fig. 5:**
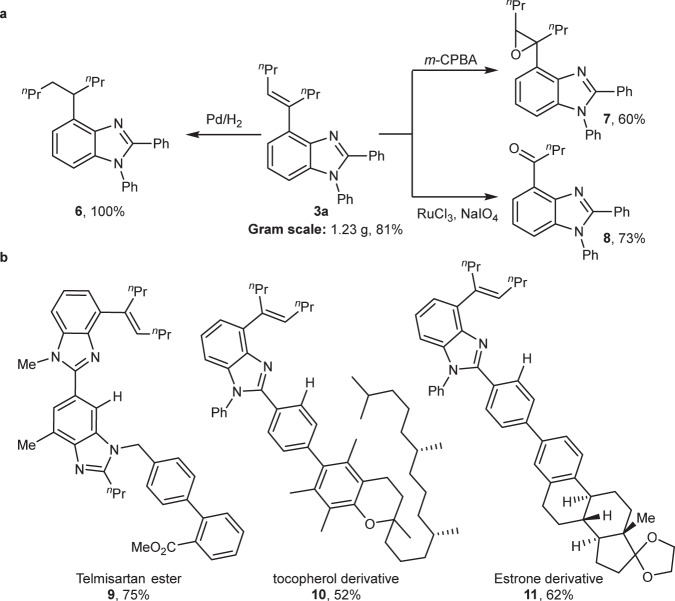
Synthetic utility. **a** Gram-scale reaction and product transformations. **b** Late-stage selective *β*-C−H bond alkenylation of bioactive molecules such as Telmisartan ester, tocopherol derivative and estrone derivative. ^*n*^Pr = *n*-propyl. *m*-CPBA = *m*-chloro perbenzoic acid.

To demonstrate the utility of the reaction, a gram-scale reaction of the model substrates was conducted, providing the desired product **3a** in 81% yield (Fig. 5a). The alkene motif in the product is a versatile synthetic precursor and it can be transformed into alkyl group through hydrogenation (**6**, quantitative yield), epoxide (**7**, 60% yield), and ketone (**8**, 73% yield) through different extent of oxidation. In addition, the selective C−H alkenylation method can be applied to the late-stage derivation of complex molecules (Fig. 5b). Telmisartan ester, a long-acting antihypertensive drug, can be selectively activated at C4−H of benzimidazole, achieving a new telmisartan derivative **9** in 75% yield. Other bioactive molecules such as tocopherol and estrone-derivatives were also well compatible with the reaction, providing the corresponding products **10** in 52% yield and **11** in 62% yield, respectively. Compared with traditional selectivity that dominantly occurs at *ortho*-C−H bond of C2-phenyl ring of benzimidazoles^66–69^, the current method provides a different molecular elaboration.

### Mechanistic discussion

**Fig. 6:**
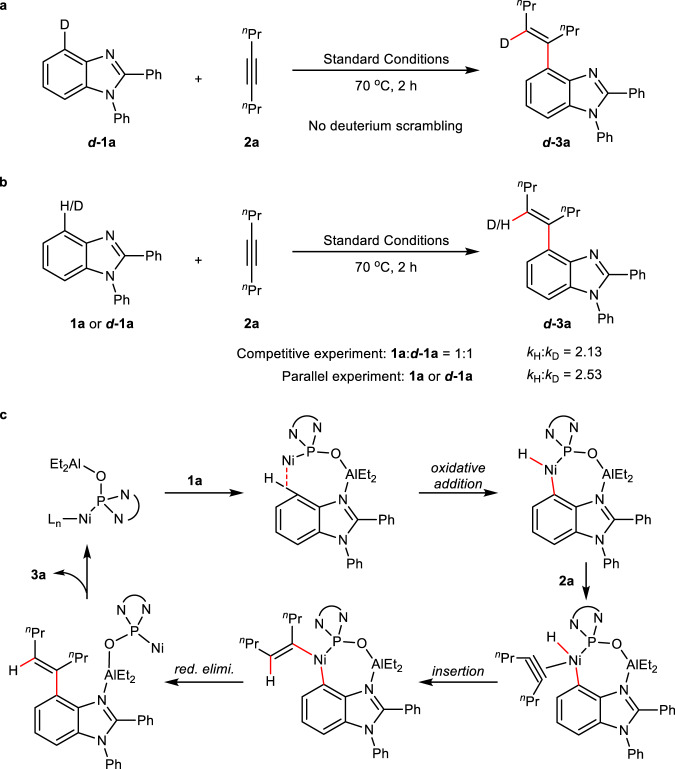
Mechanistic experiments. **a** Deuterium-labeling experiment, showing that C4−D was completely transferred to the alkene of the product. **b** Kinetic isotopic effect determination: a significant KIE for C4−H bond suggests that the activation of C4−H bond could be a rate-determining step. **c** Proposed mechanism.

To gain insight into this reaction, additional mechanistic experiments were conducted. Deuterium-labeling experiment disclosed that the alkenyl H completely came from aryl H on the C4-position of benzimidazole (Fig. 6a), suggesting a C4−H bond metalation. The determination of kinetic isotopic effect via either intermolecular competitive experiment or parallel experiments revealed significant isotopic effect (Fig. 6b), suggesting that C4−H bond cleavage may be involved into a rate-determining step. On basis of these results, a plausible mechanism was proposed in Fig. 6c. Al-Lewis acid of phosphine oxide-ligated Ni−Al bimetallic catalyst coordinates to N atom of the imidazole first, and then nickel is directed to selectively activate *β*-C−H bond to form a 7-membered nickelacycle, bypassing the formation of a more challenging 4-membered nickelacycle and a traditional 5-membered metallacycle. Subsequent alkyne insertion and reductive elimination delivered the desired product **3a** and regenerated the bimetallic catalyst.

In summary, we have developed an unactivated *β*-C(sp^2^)−H bond-involved hydroarylation of alkynes, providing a series of C4-alkenylated 2-phenyl benzimidazoles including bioactive complex molecules in 36−96% yield, overriding traditional γ-selectivity via a stable 5-membered metallacycle. The phosphine oxide-ligated Ni−Al bimetallic catalyst effectively directed Ni to generate *β*-selectivity via a 7-membered metallacycle, bypassing a highly strained 4-membered nickelacycle. The ligand-ligated bimetallic catalyst provides an efficient tool for site selective C−H bond activation and would find wide applications in other types of reactions in future.

## Methods

### General procedure for *β*-C(sp^2^)−H bond-involved hydroarylation

In an argon-filled glove-box, to an oven-dried sealed tube were added Ni(cod)_2_ (11.0 mg, 0.04 mmol), **PO-3** (17.2 mg, 0.04 mmol), toluene (0.8 mL), **1** (0.40 mmol), AlEt_3_ (1 mol/L in hexane, 160 μL, 0.16 mmol), and **2** (0.80 mmol) in sequence. The tube was then sealed, removed out of the glove-box, and heated at 80 °C with heating mantle as the heat source for 12 h. Then the mixture was cooled to room temperature and concentrated in vacuo. The crude product was purified by flash column chromatography using ethyl acetate/hexane as eluent.

## Supplementary information

Supplementary Information

## Data Availability

The authors declare that the data supporting the findings of this study are available within the article and its Supplementary Information file. For the experimental procedures and data of NMR see Supplementary Methods in Supplementary Information file. The X-ray crystallographic coordinates for structures reported in this study have been deposited at the Cambridge Crystallographic Data Centre (CCDC), under deposition number CCDC 2101580. These data can be obtained free of charge from The Cambridge Crystallographic Data Centre via https://www.ccdc.cam.ac.uk/structures/.
