# Supplementary material for: Ni-catalyzed hydroarylation of alkynes with unactivated β-C(sp2)−H bonds
Source: Nat Commun. 2022 May 26;13:2938. doi: 10.1038/s41467-022-30367-8 (PMC9135730; doi:10.1038/s41467-022-30367-8)
Supplement: Supplementary file 1 — Supplementary Information [file 41467_2022_30367_MOESM1_ESM.pdf]

Supplementary Information

Ni-Catalyzed Hydroarylation of Alkynes with Unactivated *β*-C(sp^2)–H Bonds

Qi et al.

Supplementary Methods

General Information

Unless stated otherwise, all reactions were conducted under N_2 atmosphere. All solvents were received from commercial sources without further purification. Commercially available reagents were used as received. Non-commercially available substrates were synthesized following reported protocols. Melting points were measured on X-4B microscope melting point apparatus (Beijing Tech. Instrument Co., Beijing, China) and uncorrected. Thin-layer chromatography (TLC) was performed by UV absorbance (254 nm). 200–300 mesh silica gel was used for column chromatography separation. NMR spectra were recorded on Bruker AV 400 spectrometer at 400 MHz (^{1}H NMR), 100 MHz (^{13}C NMR), 376 MHz (^{19}F NMR) and 162 MHz (^{31}P NMR). Proton and carbon chemical shifts are reported relative to the solvent used as an internal reference ($CDCl_3$: δ_H = 7.26 ppm; δ_C = 77.16 ppm). All coupling constants (*J* values) were reported in Hertz (Hz). Multiplicities are reported as follows: singlet (s), doublet (d), doublet of doublets (dd), triplet (t), triplet of doublets (td), quartet (q), and multiplet (m). High resolution mass spectra (HRMS) were recorded on an Agilent 6520 Q-TOF LC/MS with Electron Spray Ionization (ESI) resource.

Supplementary Note 1

Substrate Preparation

Method A

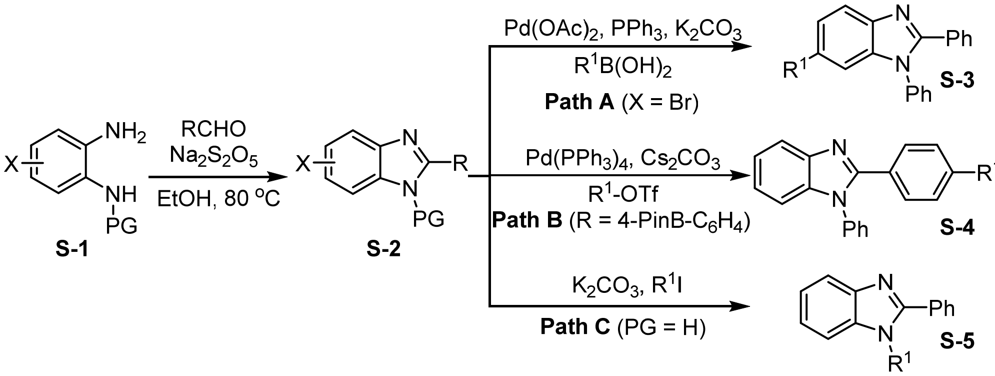

A mixture of **S-1** (5.0 mmol, 1.0 equiv.), aldehyde (5.0 mmol, 1.0 equiv.), $Na_2S_2O_5$ (1.43 g, 7.5 mmol, 1.5 equiv.), and ethanol was stirred at 80 ºC in a 100 mL round-bottomed flask under O_2 atmosphere. After completion of the reaction, the reaction mixture was cooled to rt, diluted with water, and extracted with DCM. The organic layers were combined, dried over anhydrous Na_2SO_4, filtered and concentrated in vacuo. The crude product was purified by flash column chromatography on silica gel (eluting with EtOAc/*n*-hexane) to give the product **S-2**.

Path A: In an argon-filled glove-box, to an oven-dried sealed tube were added

6-bromo-1,2-diphenyl-1*H*-benzo[*d*]imidazole (3.48 g, 10 mmol), $RB(OH)_2$ (1.2 equiv., 12 mmol), $Pd(OAc)_2$ (112 mg, 0.5 mmol), PPh_3 (393.4 mg, 1.5 mmol), K_2CO_3 (5.53 g, 40 mmol), EtOH (5 mL), water (10 mL) and toluene (20 mL) in sequence. The tube was then sealed and removed out of the glove-box. The mixture was heated at 80°C with heating mantle as the heat source for 12 h, then cooled to room temperature and filtered through a pad of Celite eluting with DCM. The filtrate was extracted with DCM and the organic phase was washed with brine, dried over anhydrous Na_2SO_4 and concentrated in vacuo. The residue was purified by column chromatography on silica gel (eluting with a gradient of EtOAc/*n*-hexane).

Path B: In an argon-filled glove-box, to an oven-dried sealed tube were added 4-(1-phenyl-1*H*-benzo[*d*]imidazol-2-yl)boronic ester (3.48 g, 10 mmol), ROTf (1.5 equiv., 15 mmol), $Pd(PPh_3)_4$ (1.16 g, 1.0 mmol), Cs_2CO_3 (9.78 g, 30 mmol), EtOH (5 mL) and THF (20 mL) in sequence. The tube was then sealed, removed out of the glove-box and heated at 120°C with heating mantle as the heat source for 24 h. Then the mixture was cooled to room temperature and filtered through a pad of Celite eluting with EA. The filtrate was extracted with EA and the combined organic phase was washed with brine, dried over anhydrous Na_2SO_4 and concentrated in vacuo. The residue was purified by column chromatography on silica gel (eluting with a gradient of EtOAc/*n*-hexane).

Path C: To the mixture of 2-phenyl-1*H*-benzimidazol (1.94 g, 10 mmol), K_2CO_3 (4.15 g, 30 mmol) was added MeI (15 mmol, 1.5 equiv.) dropwise at 0 ºC. After stirring 5 min at 0 ºC, the reaction solution was stirred 12 h at room temperature. After completion of the reaction, the mixture was diluted with DCM, washed with brine, dried over anhydrous Na_2SO_4, filtered and concentrated in vacuo. The residue was purified by column chromatography on silica gel (eluting with a gradient of EtOAc/ *n*-hexane) to give the product.

Method B

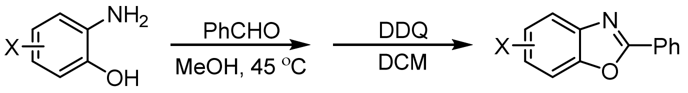

S-6 **S-7**

A mixture of **S-6** (5.0 mmol, 1.0 equiv.), aldehyde (5.0 mmol, 1.0 equiv.) and MeOH was stirred at 45 ºC in a 100 mL round-bottomed flask for 3-4 hours. After completion of the reaction, the reaction mixture was cooled to rt, concentrated in vacuo. Then DDQ (5.5 mmol, 1.1 equiv.) and DCM was added. The reaction was stirred at room temperature for 2 hours, and quenched by saturated $NaHCO_3$. The reaction mixture was washed with brine, and the organic layers were dried over anhydrous Na_2SO_4, filtered and concentrated in vacuo. The crude product was purified by flash column chromatography on silica gel (eluting with EtOAc/*n*-hexane) to give the product **S-7**.

Method C

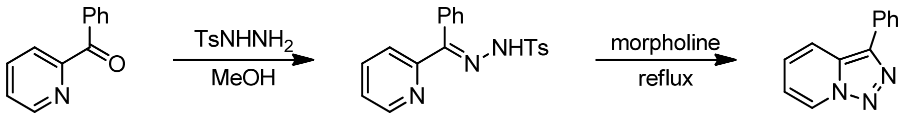

S-8 **S-9** **S-10**

To a mixture of *p*-toluenesulfonylhydrazide (5.5 mmol) and 3 mL of methanol was rapidly added **S-8** (5 mmol). Within a few minutes, tosylhydrazone **S-9** began to crystallize. The reaction was stirred for another one hour, and then cooled in an ice bath. The product was collected on a Büchner funnel, washed with a small amount of cold methanol, and dried under high vacuum. Then tosylhydrazone **S-9** was disssolved in morpholine (1.8 mL/g), and the mixture was stirred at 90–100 °C. After 1–4 h, excess morpholine was removed *in vacuo*. The resulting yellow solid was suspended in diethyl ether, and the residue was filtered to remove morpholine toluenesulphinate. The filtrate was concentrated *in vacuo*, and the resulting solid residue was purified by flash column chromatography on silica gel to provide the product **S-10**.

Method D

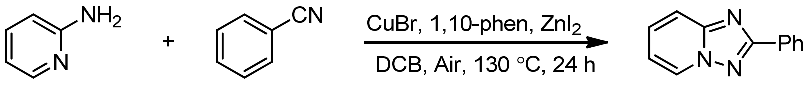

S-11 **S-12** **S-13**

To a dried screw-cap vial were added nitrile (5 mmol), 2-aminopyridine (6 mmol), CuBr (35.9 mg, 0.25 mmol), 1,10-phenanthroline (45.1 mg, 0.25 mmol) and ZnI_2 (159.6 mg, 0.5 mmol). 1,2-Dichlorobenzene (10.0 mL) was then added and the vial was sealed under air atmosphere. The reaction mixture was stirred at 130 °C for 24 h in pre-heated oil bath. After cooling to room temperature, the reaction was diluted with EtOAc and filtered over glass filter. The filtrate was concentrated and purified by column chromatography on silica gel.

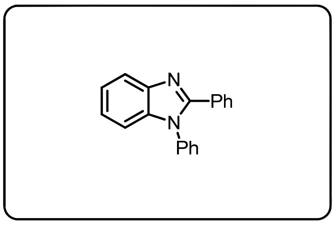

1,2-Diphenyl-1*H*-benzo[*d*]imidazole (1a)

White solid (1.35 g, 99% yield), m.p. 106–108 °C. **^{1}H NMR** (400 MHz, $CDCl_3$) δ 7.89 (d, J = 8.0 Hz, 1H), 7.60 – 7.55 (m, 2H), 7.53 – 7.44 (m, 3H), 7.38 – 7.26 (m, 8H). **^{13}C NMR** (100 MHz, $CDCl_3$) δ 152.5, 143.1, 137.3, 137.1, 130.1, 130.0, 129.7, 129.6, 128.7, 128.4, 127.5, 123.5, 123.1, 120.0, 110.6. **HRMS(ESI)** m/z: $[M+H]^+$ Calcd. for $C_{19}H_{15}N_2$ 271.1230; Found 271.1229.

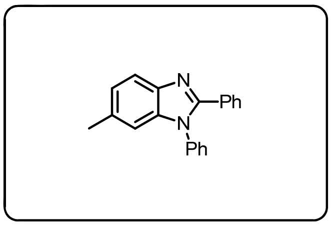

6-Methyl-1,2-diphenyl-1*H*-benzo[*d*]imidazole (1b)

White solid (1.41 g, 99% yield), m.p. 139–141 °C.^{1}**H NMR** (400 MHz, $CDCl_3$) δ 7.76 (d, J = 8.2 Hz, 1H), 7.58 – 7.43 (m, 5H), 7.37 – 7.26 (m, 5H), 7.16 (d, J = 8.2 Hz, 1H), 7.03 (s, 1H). ^{13}**C NMR** (100 MHz, $CDCl_3$) δ 152.0, 141.2, 137.5, 137.2, 133.5, 130.2, 129.9, 129.4, 129.3, 128.5, 128.3, 127.5, 124.6, 119.4, 110.4, 21.9. **HRMS(ESI)** m/z: $[M+H]^+$ Calcd. for $C_{20}H_{17}N_2$ 285.1386; Found 285.1384.

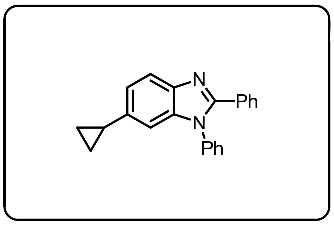

6-Cyclopropyl-1,2-diphenyl-1*H*-benzo[*d*]imidazole (1c)

Path A. White solid (2.76 g, 89% yield), m.p. 74–76 °C. ^{1}**H NMR** (400 MHz, $CDCl_3$) δ 7.76 (d, J = 8.2 Hz, 1H), 7.57 – 7.44 (m, 5H), 7.36 – 7.26 (m, 5H), 7.06 (dd, J = 8.4, 1.6 Hz, 1H), 6.96 (s, 1H), 2.04 – 1.92 (m, 1H), 0.99 – 0.91 (m, 2H), 0.72 – 0.66 (m, 2H). ^{13}**C NMR** (100 MHz, $CDCl_3$) δ 152.1, 141.4, 139.8, 137.5, 137.2, 130.1, 130.0, 129.4, 128.6, 128.4, 127.6, 121.3, 119.7, 119.5, 107.5, 16.0, 16.0, 9.4. **HRMS(ESI)** m/z: $[M+H]^+$ Calcd. for $C_{22}H_{19}N_2$ 311.1543; Found 311.1538.

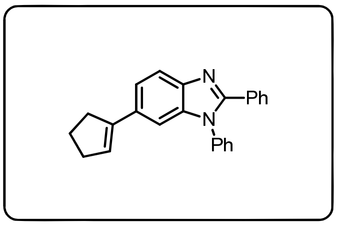

6-(Cyclopent-1-en-1-yl)-1,2-diphenyl-1*H*-benzo[*d*]imidazole (1d)

Path A. White solid (2.99 g, 89% yield), m.p. 142–144 °C. ^{1}**H NMR** (400 MHz, $CDCl_3$) δ 7.80 (d, J = 8.4 Hz, 1H), 7.57 – 7.48 (m, 6H), 7.36 – 7.28 (m, 5H), 7.22 (s, 1H), 6.19 – 6.14 (m, 1H), 2.75 – 2.67 (m, 2H), 2.56 – 2.48 (m, 2H), 2.07 – 1.96 (m, 2H). ^{13}**C NMR** (100 MHz, $CDCl_3$) δ 152.6, 142.8, 142.3, 137.5, 137.1, 133.0, 130.0, 130.0, 129.6, 129.5, 128.7, 128.4, 127.6, 125.9, 121.6, 119.5, 107.2, 33.7, 33.5, 23.5. **HRMS(ESI)** m/z: $[M+H]^+$ Calcd. for $C_{24}H_{21}N_2$ 337.1699; Found 337.1695.

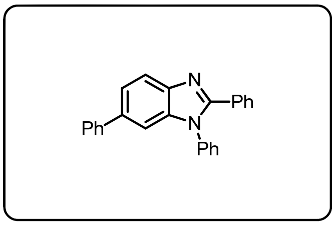

1,2,6-Triphenyl-1*H*-benzo[*d*]imidazole (1e)

Path A. White solid (3.08 g, 89% yield), m.p. 210–212 °C. ^{1}**H NMR** (400 MHz, $CDCl_3$) δ 7.94 (d, J = 8.4 Hz, 1H), 7.62 – 7.56 (m, 5H), 7.55 – 7.48 (m, 3H), 7.45 – 7.40 (m, 3H), 7.39 – 7.28 (m, 6H). ^{13}**C NMR** (100 MHz, $CDCl_3$) δ 153.1, 142.6, 141.9, 137.9, 137.3, 137.1, 133.7, 133.5, 130.1, 129.6, 129.6, 128.9, 128.8, 128.5, 127.6, 127.1, 123.1, 120.1, 109.1. **HRMS(ESI)** m/z: $[M+H]^+$ Calcd. for $C_{25}H_{19}N_2$ 347.1543; Found

347.1539.

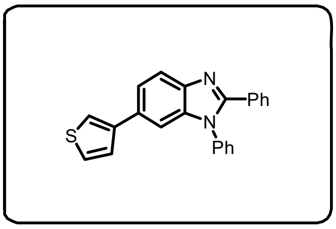

1,2-Diphenyl-6-(thiophen-3-yl)-1*H*-benzo[*d*]imidazole (1f)

Path A. White solid (3.13 g, 89% yield), m.p. 207–209 °C. **^{1}H NMR** (400 MHz, $CDCl_3$) δ 7.89 (d, J = 8.4 Hz, 1H), 7.63 – 7.47 (m, 6H), 7.42 – 7.39 (m, 2H), 7.39 – 7.28 (m, 7H). **^{13}C NMR** (100 MHz, $CDCl_3$) δ 153.0, 142.9, 142.5, 137.9, 137.0, 131.9, 130.1, 130.0, 129.6, 129.5, 128.8, 128.4, 127.6, 126.8, 126.3, 122.5, 120.2, 120.1, 108.2. **HRMS(ESI)** m/z: $[M+H]^+$ Calcd. for $C_{23}H_{17}N_2S$ 353.1107; Found 353.1103.

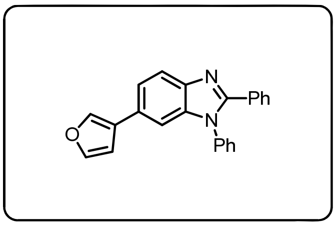

6-(Furan-3-yl)-1,2-diphenyl-1*H*-benzo[*d*]imidazole (1g)

Path A. White solid (2.99 g, 89% yield), m.p. 170–172 °C. **^{1}H NMR** (400 MHz, $CDCl_3$) δ 7.82 (d, J = 8.4 Hz, 1H), 7.65 (s, 1H), 7.55 – 7.37 (m, 7H), 7.33 – 7.21 (m, 6H), 6.62 (s, 1H). **^{13}C NMR** (100 MHz, $CDCl_3$) δ 152.8, 143.7, 142.5, 138.4, 137.9, 137.0, 130.1, 130.0, 129.6, 129.5, 128.8, 128.4, 128.2, 127.6, 127.0, 121.8, 120.2, 109.3, 107.5. **HRMS(ESI)** m/z: $[M+H]^+$ Calcd. for $C_{23}H_{17}N_2O$ 337.1335; Found 337.1329.

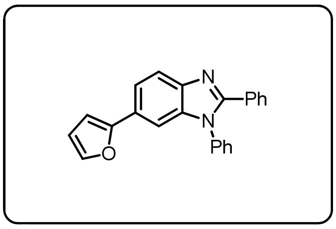

6-(Furan-2-yl)-1,2-diphenyl-1*H*-benzo[*d*]imidazole (1h)

Path A. White solid (2.99 g, 89% yield), m.p. 186–188 °C. **^{1}H NMR** (400 MHz, $CDCl_3$) δ 7.87 (d, J = 8.4 Hz, 1H), 7.66 (d, J = 8.4 Hz, 1H), 7.61 – 7.47 (m, 6H), 7.43 (s, 1H), 7.39 – 7.27 (m, 5H), 6.63 (d, J = 3.0 Hz, 1H), 6.46 (s, 1H). **^{13}C NMR** (100 MHz, $CDCl_3$) δ 154.6, 153.1, 142.7, 141.9, 137.8, 137.0, 130.1, 129.9, 129.6, 129.5, 128.8, 128.4, 127.6, 126.8, 120.2, 120.0, 111.9, 105.7, 104.8. **HRMS(ESI)** m/z: $[M+H]^+$ Calcd. for $C_{23}H_{17}N_2O$ 337.1335; Found 337.1332.

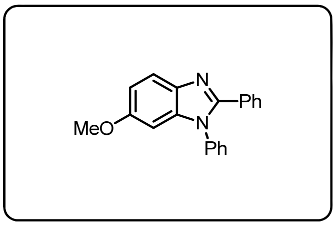

6-Methoxy-1,2-diphenyl-1*H*-benzo[*d*]imidazole (1i)

White solid (1.48 g, 99% yield), m.p. 153–155 °C. **^{1}H NMR** (400 MHz, $CDCl_3$) δ 7.77 (d, J = 8.8 Hz, 1H), 7.57 – 7.44 (m, 5H), 7.37 – 7.22 (m, 5H), 6.97 (dd, J = 8.8, 2.2 Hz, 1H), 6.69 (d, J = 2.0 Hz, 1H), 3.8 (s, 3H). **^{13}C NMR** (100 MHz, $CDCl_3$) δ 157.3, 151.8, 138.0, 137.6, 137.2, 130.2, 130.0, 129.3, 129.3, 128.7, 128.4, 127.5, 120.5, 112.4, 94.1, 56.0. **HRMS(ESI)** m/z: $[M+H]^+$ Calcd. for $C_{20}H_{17}N_2O$ 301.1335; Found 301.1333.

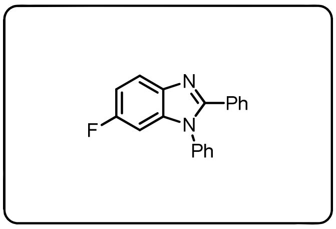

6-Fluoro-1,2-diphenyl-1*H*-benzo[*d*]imidazole (1j)

White solid (1.43 g, 99% yield), m.p. 126–128 °C. **^{1}H NMR** (400 MHz, $CDCl_3$) δ 7.83 – 7.77 (m, 1H), 7.58 – 7.45 (m, 5H), 7.39 – 7.27 (m, 5H), 7.12 – 7.04 (m, 1H), 6.93 (dd, J = 8.8, 2.4 Hz, 1H). **^{13}C NMR** (100 MHz, $CDCl_3$) δ 160.2 (d, J = 239.0 Hz), 153.2 (d, J = 3.0 Hz), 139.5, 137.5 (d, J = 13.0 Hz), 136.8, 130.1, 129.8, 129.7, 129.4, 128.9, 128.5, 127.3, 120.7 (d, J = 10.0 Hz), 111.5 (d, J = 25.0 Hz), 97.3 (d, J = 28.0 Hz). **^{19}F NMR** (376 MHz, $CDCl_3$) δ -117.9. **HRMS(ESI)** m/z: $[M+H]^+$ Calcd. for $C_{19}H_{14}FN_2$ 289.1136; Found 289.1133.

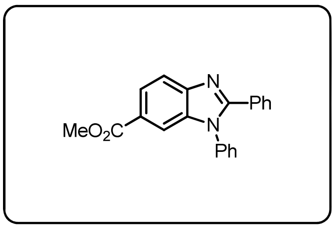

Methyl 1,2-diphenyl-1*H*-benzo[*d*]imidazole-6-carboxylate (1k)

White solid (1.62 g, 99% yield), m.p. 195–197 °C. **^{1}H NMR** (400 MHz, $CDCl_3$) δ 8.09 – 8.03 (m, 1H), 7.96 (s, 1H), 7.90 (d, J = 8.4 Hz, 1H), 7.61 – 7.51 (m, 5H), 7.42 – 7.28 (m, 5H), 3.91 (s, 3H). **^{13}C NMR** (100 MHz, $CDCl_3$) δ 167.5, 155.1, 146.4, 137.1, 136.5, 130.3, 130.2, 129.7, 129.4, 129.2, 128.6, 127.6, 125.3, 124.7, 119.6, 112.9, 52.3. **HRMS(ESI)** m/z: $[M+H]^+$ Calcd. for $C_{21}H_{17}N_2O_2$ 329.1285; Found 329.1280.

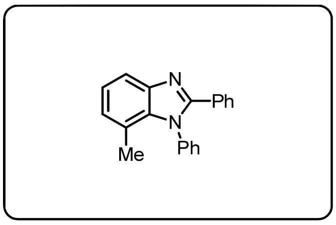

7-Methyl-1,2-diphenyl-1*H*-benzo[*d*]imidazole (1l)

White solid (1.41 g, 99% yield), m.p. 121–123 °C. **^{1}H NMR** (400 MHz, $CDCl_3$) δ 7.76 (d, J = 8.0 Hz, 1H), 7.54 – 7.41 (m, 5H), 7.39 – 7.34 (m, 2H), 7.32 – 7.21 (m, 4H), 6.98 (d, J = 7.2 Hz, 1H), 1.94 (s, 3H). **^{13}C NMR** (100 MHz, $CDCl_3$) δ 153.0, 143.1, 138.5, 135.7, 130.4, 129.7, 129.6, 129.4, 129.3, 129.2, 128.2, 125.9, 122.8, 122.1, 118.0, 18.4. **HRMS(ESI)** m/z: $[M+H]^+$ Calcd. for $C_{20}H_{17}N_2$ 285.1386; Found 285.1386.

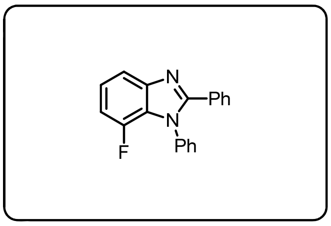

7-Fluoro-1,2-diphenyl-1*H*-benzo[*d*]imidazole (1m)
White solid (1.43 g, 99% yield), m.p. 110–112 °C. **^{1}H NMR** (400 MHz, $CDCl_3$) δ 7.67 (d, J = 8.0 Hz, 1H), 7.55 – 7.49 (m, 2H), 7.46 – 7.38 (m, 3H), 7.37 – 7.20 (m, 6H), 6.99 – 6.90 (m, 1H). **^{13}C NMR** (100 MHz, $CDCl_3$) δ 151.92 (d, J = 298.0 Hz), 148.0, 146.2 (d, J = 3.0 Hz), 137.5, 129.7, 129.6, 129.5, 129.3, 128.9, 128.4, 128.0, 125.1 (d, J = 8.0 Hz), 122.8 (d, J = 7.0 Hz), 116.0 (d, J = 4.0 Hz), 109.6 (d, J = 17.0 Hz). **^{19}F NMR** (376 MHz, $CDCl_3$) δ -130.7. **HRMS(ESI)** m/z: $[M+H]^+$ Calcd. for $C_{19}H_{14}FN_2$ 289.1136; Found 289.1131.

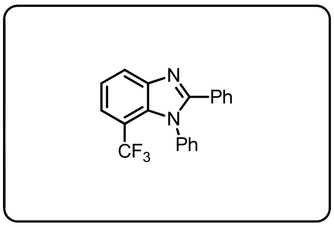

1,2-Diphenyl-7-(trifluoromethyl)-1*H*-benzo[*d*]imidazole (1n)
White solid (1.64 g, 97% yield), m.p. 91–93 °C. **^{1}H NMR** (400 MHz, $CDCl_3$) δ 8.09 (d, J = 8.0 Hz, 1H), 7.61 (d, J = 7.6 Hz, 1H), 7.51 – 7.37 (m, 6H), 7.36 – 7.29 (m, 3H), 7.28 – 7.23 (m, 2H). **^{13}C NMR** (100 MHz, $CDCl_3$) δ 155.3, 144.9, 137.4, 133.4, 129.9, 129.8, 129.7, 129.7, 129.1, 128.3, 124.6, 124.4, 122.1 (q, J = 6.0 Hz), 122.0, 121.9, 114.7 (q, J = 33.0 Hz). **^{19}F NMR** (376 MHz, $CDCl_3$) δ -56.1. **HRMS(ESI)** m/z: $[M+H]^+$ Calcd. for $C_{20}H_{14}F_3N_2$ 339.1104; Found 339.1106.

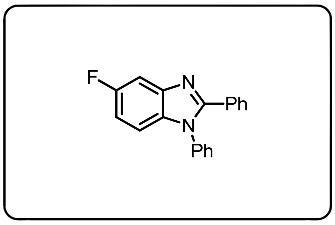

5-Fluoro-1,2-diphenyl-1*H*-benzo[*d*]imidazole (1o)
White solid (1.43 g, 99% yield), m.p. 119–121 °C. **^{1}H NMR** (400 MHz, $CDCl_3$) δ 7.58 – 7.45 (m, 6H), 7.40 – 7.27 (m, 5H), 7.19 – 7.13 (m, 1H), 7.05 – 6.97 (m, 1H). **^{13}C NMR** (100 MHz, $CDCl_3$) δ 160.1 (d, J = 237.0 Hz), 153.8, 143.4 (d, J = 13.0 Hz),, 136.8, 133.9, 130.1, 129.8, 129.7, 129.5, 128.9, 128.5, 127.4, 111.7 (d, J = 26.0 Hz), 111.0 (d, J = 10.0 Hz), 105.6 (d, J = 24.0 Hz). **^{19}F NMR** (376 MHz, $CDCl_3$) δ -120.1. **HRMS(ESI)** m/z: $[M+H]^+$ Calcd. for $C_{19}H_{14}FN_2$ 289.1136; Found 289.1132.

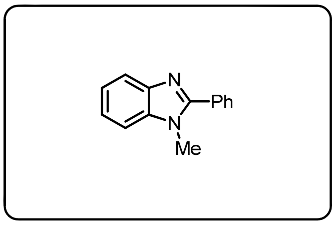

1-Methyl-2-phenyl-1*H*-benzo[*d*]imidazole (1p)

Path C. White solid (2.02 g, 97% yield), m.p. 94–96 °C. **^{1}H NMR** (400 MHz, $CDCl_3$) δ 7.86 – 7.81 (m, 1H), 7.79 – 7.75 (m, 2H), 7.57 – 7.50 (m, 3H), 7.43 – 7.38 (m, 1H), 7.37 – 7.29 (m, 2H), 3.88 (s, 3H). **^{13}C NMR** (100 MHz, $CDCl_3$) δ 153.7, 142.9, 136.6, 130.2, 129.7, 129.4, 128.7, 122.8, 122.4, 119.8, 109.7, 31.6. **HRMS(ESI)** m/z: $[M+H]^+$ Calcd. for $C_{14}H_{13}N_2$ 209.1073; Found 209.1071.

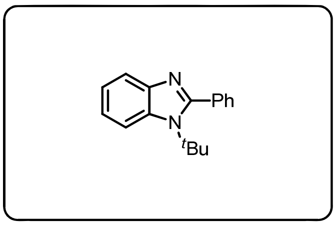

1-(*tert*-Butyl)-2-phenyl-1*H*-benzo[*d*]imidazole (1q)

White solid (2.99 g, 89% yield) , m.p. 118–120 °C. **^{1}H NMR** (400 MHz, $CDCl_3$) δ 7.82 – 7.70 (m, 2H), 7.52 – 7.38 (m, 5H), 7.31 – 7.24 (m, 2H), 1.62 (s, 9H). **^{13}C NMR** (100 MHz, $CDCl_3$) δ 153.7, 143.4, 136.0, 135.0, 129.8, 129.1, 127.9, 122.1, 122.0, 120.3, 114.8, 59.0, 31.5. **HRMS(ESI)** m/z: $[M+H]^+$ Calcd. for $C_{17}H_{19}N_2$ 251.1543; Found 251.1542.

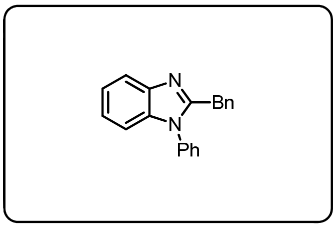

2-Benzyl-1-phenyl-1*H*-benzo[*d*]imidazole (1s)

White solid (1.37 g, 96% yield), m.p. 104–106 °C. **^{1}H NMR** (400 MHz, $CDCl_3$) δ 7.51 – 7.43 (m, 1H), 7.33 – 7.24 (m, 3H), 7.22 – 7.13 (m, 2H), 7.09 – 7.00 (m, 6H), 4.18 (s, 3H). **^{13}C NMR** (100 MHz, $CDCl_3$) δ 153.5, 142.6, 136.9, 136.7, 135.9, 129.8, 129.1, 128.8, 128.5, 127.7, 126.7, 122.9, 122.5, 119.5, 110.2, 34.4. **HRMS(ESI)** m/z: $[M+H]^+$ Calcd. for $C_{20}H_{17}N_2$ 285.1386; Found 285.1386.

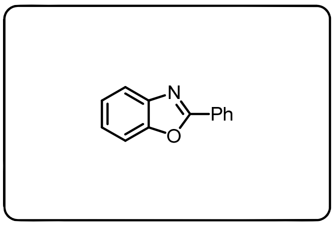

2-Phenylbenzo[*d*]oxazole (1u)

Method B. White solid (965.7 mg, 99% yield), m.p. 100–102 °C. **^{1}H NMR** (400 MHz,

CDCl3) δ 8.27 (m, 2H), 7.82 – 7.76 (m, 1H), 7.62 – 7.57 (m, 1H), 7.56 – 7.50 (m, 3H), 7.39 – 7.33 (m, 2H). **^{13}C NMR** (100 MHz, CDCl3) δ 163.2, 150.9, 142.2, 131.7, 129.1, 127.8, 127.3, 125.3, 124.7, 120.1, 110.8. **HRMS(ESI)** m/z: $[M+H]^+$ Calcd. for $C_{13}H_{10}NO$ 196.0757; Found 196.0759.

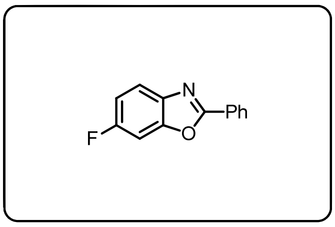

6-Fluoro-2-phenylbenzo[*d*]oxazole (1v)

Method B. White solid (1.02 g, 96% yield), m.p. 100–102 °C. **^{1}H NMR** (400 MHz, CDCl3) δ 8.24 – 8.17 (m, 2H), 7.68 (dd, *J* = 8.7, 4.9 Hz, 1H), 7.56 – 7.48 (m, 3H), 7.29 (dd, *J* = 8.0, 2.3 Hz, 1H), 7.13 – 7.05 (m, 1H). **^{13}C NMR** (100 MHz, CDCl3) δ 163.8 (d, *J* = 3.0 Hz), 160.8 (d, *J* = 243.0 Hz), 150.8 (d, *J* = 14.0 Hz), 138.5 (d, *J* = 2.0 Hz), 131.7, 129.1, 127.6, 127.0, 120.4 (d, *J* = 10.0 Hz), 112.7 (d, *J* = 25.0 Hz), 98.8 (d, *J* = 28.0 Hz). **^{19}F NMR** (376 MHz, CDCl3) δ -115.1. **HRMS(ESI)** m/z: $[M+H]^+$ Calcd. for $C_{13}H_9FNO$ 214.0663; Found 214.0666.

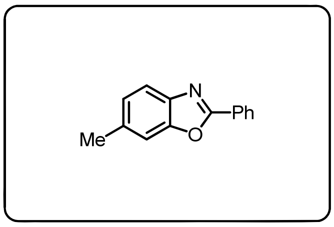

6-Methyl-2-phenylbenzo[*d*]oxazole (1w)

Method B. White solid (972.3 mg, 93% yield), m.p. 91–93 °C. **^{1}H NMR** (400 MHz, CDCl3) δ 8.25 – 8.17 (m, 2H), 7.63 (d, *J* = 8.1 Hz, 1H), 7.53 – 7.47 (m, 3H), 7.36 (s, 1H), 7.15 (d, *J* = 8.0 Hz, 1H), 2.48 (s, 3H). **^{13}C NMR** (100 MHz, CDCl3) δ 162.7, 151.2, 140.0, 135.7, 131.4, 129.0, 127.6, 127.5, 125.9, 119.4, 110.9, 21.9. **HRMS(ESI)** m/z: $[M+H]^+$ Calcd. for $C_{14}H_{12}NO$ 210.0913; Found 210.0914.

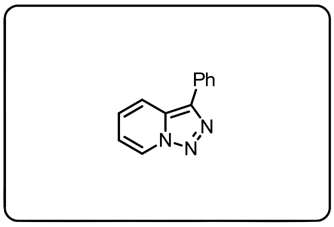

3-Phenyl-[1,2,3]triazolo[1,5-*a*]pyridine (1x)

Method C.White solid (936.5 mg, 96% yield), m.p. 111–113 °C. **^{1}H NMR** (400 MHz, CDCl3) δ 8.74 (d, *J* = 7.1 Hz, 1H), 8.03 – 7.93 (m, 3H), 7.51 (t, *J* = 7.6 Hz, 2H), 7.39 (t, *J* = 7.4 Hz, 1H), 7.29 (dd, *J* = 8.4, 6.8 Hz, 1H), 6.99 (t, *J* = 6.8 Hz, 1H). **^{13}C NMR** (100 MHz, CDCl3) δ 138.1, 131.6, 130.6, 129.1, 128.0, 126.8, 125.7, 118.5, 115.4. **HRMS(ESI)** m/z: $[M+H]^+$ Calcd. for $C_{12}H_{10}N_3$ 196.0869; Found 196.0871.

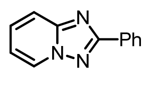

2-Phenyl-[1,2,4]triazolo[1,5-*a*]pyridine (1y)

Method D.White solid (663.3 mg, 68% yield), m.p. 138–140 °C. **^{1}H NMR** (400 MHz, $CDCl_3$) δ 8.60 (d, *J* = 6.8 Hz, 1H), 8.30 (d, *J* = 6.6 Hz, 2H), 7.76 (d, *J* = 8.9 Hz, 1H), 7.54 – 7.43 (m, 4H), 6.99 (t, *J* = 6.8 Hz, 1H). **^{13}C NMR** (100 MHz, $CDCl_3$) *δ* 164.3, 151.8, 130.9, 130.2, 129.6, 128.8, 128.5, 127.4, 116.5, 113.7. **HRMS(ESI)** m/z: $[M+H]^+$ Calcd. for $C_{12}H_{10}N_3$ 196.0869; Found 196.0871.

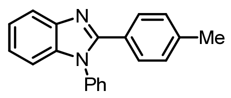

1-Phenyl-2-(*p*-tolyl)-1*H*-benzo[*d*]imidazole (1aa)

White solid (1.35 g, 95% yield), m.p. 125–127 °C. **^{1}H NMR** (400 MHz, $CDCl_3$) *δ* 7.88 (d, *J* = 8.0 Hz, 1H), 7.54 – 7.42 (m, 5H), 7.35 – 7.28 (m, 3H), 7.27 – 7.20 (m, 2H), 7.11 (s, 1H), 7.09 (s, 1H), 2.33 (s, 3H). **^{13}C NMR** (100 MHz, $CDCl_3$) *δ* 152.7, 143.1, 139.7, 137.3, 137.2, 129.9, 129.4, 129.1, 128.6, 127.5, 127.1, 123.2, 123.0, 119.8, 110.5, 21.5. **HRMS(ESI)** m/z: $[M+H]^+$ Calcd. for $C_{20}H_{17}N_2$ 285.1386; Found 283.1383.

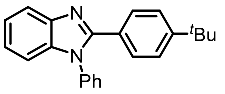

2-(4-(*tert*-Butyl)phenyl)-1-phenyl-1*H*-benzo[*d*]imidazole (1ab)

White solid (1.5 g, 92% yield), m.p. 101–103 °C. **^{1}H NMR** (400 MHz, $CDCl_3$) *δ* 7.88 (d, *J* = 8.0 Hz, 1H), 7.55 – 7.45 (m, 5H), 7.36 – 7.28 (m, 5H), 7.27 – 7.19 (m, 2H), 1.29 (s, 9H). **^{13}C NMR** (100 MHz, $CDCl_3$) *δ* 152.8, 152.6, 143.1, 137.4, 137.3, 130.0, 129.2, 128.7, 127.6, 127.1, 125.4, 123.2, 123.0, 119.8, 110.5, 34.9, 31.3. **HRMS(ESI)** m/z: $[M+H]^+$ Calcd. for $C_{23}H_{23}N_2$ 327.1856; Found 327.1855.

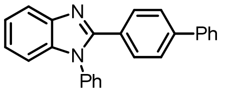

2-([1,1'-Biphenyl]-4-yl)-1-phenyl-1*H*-benzo[*d*]imidazole (1ac)

White solid (1.66 g, 96% yield), m.p. 195–197 °C. **^{1}H NMR** (400 MHz, $CDCl_3$) *δ* 7.91

(d, J = 8.0 Hz, 1H), 7.65 (d, J = 8.3 Hz, 2H), 7.60 – 7.47 (m, 7H), 7.45 – 7.31 (m, 6H), 7.30 – 7.21 (m, 2H). **^{13}C NMR** (100 MHz, $CDCl_3$) δ 152.2, 149.7, 143.2, 142.2, 140.2, 137.5, 137.2, 130.1, 129.9, 129.0, 128.8, 127.9, 127.6, 127.2, 127.0, 123.5, 123.2, 120.0, 110.6. **HRMS(ESI)** m/z: $[M+H]^+$ Calcd. for $C_{25}H_{19}N_2$ 347.1543; Found 347.1542.

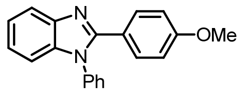

2-(4-Methoxyphenyl)-1-phenyl-1*H*-benzo[*d*]imidazole (1ad)
White solid (1.44 g, 96% yield), m.p. 135–137 °C. **^{1}H NMR** (400 MHz, $CDCl_3$) δ 7.86 (d, J = 8.0 Hz, 1H), 7.55 – 7.43 (m, 5H), 7.36 – 7.28 (m, 3H), 7.28 – 7.19 (m, 2H), 6.85 – 6.78 (m, 2H), 3.79 (s, 3H). **^{13}C NMR** (100 MHz, $CDCl_3$) δ 160.6, 152.5, 143.1, 137.3, 137.3, 131.0, 130.0, 128.6, 127.6, 123.1, 123.0, 122.4, 119.7, 113.9, 110.4, 55.4. **HRMS(ESI)** m/z: $[M+H]^+$ Calcd. for $C_{20}H_{17}N_2O$ 301.1335; Found 301.1332.

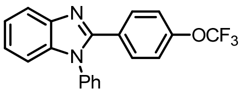

1-Phenyl-2-(4-(trifluoromethoxy)phenyl)-1*H*-benzo[*d*]imidazole (1ae)
White solid (1.72 g, 97% yield), m.p. 132–134 °C. **^{1}H NMR** (400 MHz, $CDCl_3$) δ 7.88 (d, J = 8.0 Hz, 1H), 7.64 – 7.57 (m, 2H), 7.56 – 7.46 (m, 3H), 7.38 – 7.21 (m, 5H), 7.14 (d, J = 8.4 Hz, 2H). **^{13}C NMR** (100 MHz, $CDCl_3$) δ 151.0, 150.1, 143.0, 137.4, 136.8, 131.1, 130.2, 129.0, 128.7, 127.5, 123.8, 123.3, 120.6, 120.5 (d, J = 256.0 Hz), 120.0, 110.6. **^{19}F NMR** (376 MHz, $CDCl_3$) δ -57.7. **HRMS(ESI)** m/z: $[M+H]^+$ Calcd. for $C_{20}H_{14}F_3N_2O$ 355.1053; Found 355.1050.

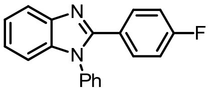

2-(4-Fluorophenyl)-1-phenyl-1*H*-benzo[*d*]imidazole (1af)
White solid (1.41 g, 98% yield), m.p. 99–101 °C. **^{1}H NMR** (400 MHz, $CDCl_3$) δ 7.87 (d, J = 8.0 Hz, 1H), 7.60 – 7.44 (m, 5H), 7.37 – 7.20 (m, 5H), 6.98 (t, J = 8.6 Hz, 2H). **^{13}C NMR** (100 MHz, $CDCl_3$) δ 163.5 (d, J = 249.0 Hz), 151.5, 143.0, 137.3 (d, J = 37.0 Hz), 131.5 (d, J = 8.0 Hz), 130.1, 128.8, 127.5, 126.2 (d, J = 3.0 Hz), 123.5, 123.2, 119.9, 115.7, 115.5, 110.6. **^{19}F NMR** (376 MHz, $CDCl_3$) δ -110.7. **HRMS(ESI)** m/z: $[M+H]^+$ Calcd. for $C_{19}H_{14}FN_2$ 289.1136; Found 289.1132.

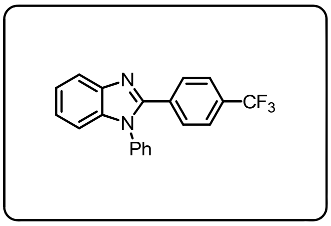

1-Phenyl-2-(4-(trifluoromethyl)phenyl)-1*H*-benzo[*d*]imidazole (1ag)
White solid (1.66 g, 98% yield), m.p. 130–132 °C. **^{1}H NMR** (400 MHz, $CDCl_3$) δ 7.91 (d, J = 8.0 Hz, 1H), 7.71 (s, 1H), 7.69 (s, 1H), 7.59 – 7.47 (m, 5H), 7.40 – 7.23 (m, 5H). **^{13}C NMR** (100 MHz, $CDCl_3$) δ 150.8, 143.0, 137.5, 136.7, 133.6, 131.3 (q, J = 32.0 Hz), 130.2, 129.8, 129.1, 127.5, 125.4 (q, J = 4.0 Hz), 124.1, 123.5, 122.6, 120.3, 110.8. **^{19}F NMR** (377 MHz, $CDCl_3$) δ -62.8. **HRMS(ESI)** m/z: $[M+H]^+$ Calcd. for $C_{20}H_{14}F_3N_2$ 339.1104; Found 339.1101.

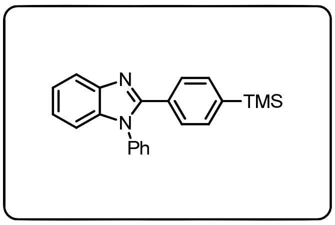

1-Phenyl-2-(4-(trimethylsilyl)phenyl)-1*H*-benzo[*d*]imidazole (1ah)
White solid (1.71 g, 99% yield), m.p. 116–118 °C. **^{1}H NMR** (400 MHz, $CDCl_3$) δ 7.89 (d, J = 8.0 Hz, 1H), 7.59 – 7.42 (m, 7H), 7.36 – 7.31 (m, 3H), 7.29 – 7.21 (m, 2H), 0.24 (s, 9H). **^{13}C NMR** (100 MHz, $CDCl_3$) δ 152.5, 143.1, 142.5, 137.5, 137.2, 133.3, 130.2, 130.0, 128.7, 128.6, 127.6, 123.4, 123.1, 120.0, 110.6, -1.1. **HRMS(ESI)** m/z: $[M+H]^+$ Calcd. for $C_{22}H_{23}N_2Si$ 343.1625; Found 343.1624.

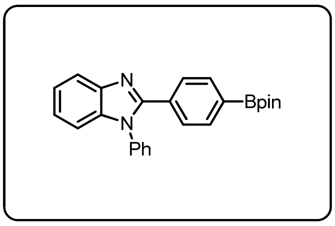

1-Phenyl-2-(4-(4,4,5,5-tetramethyl-1,3,2-dioxaborolan-2-yl)phenyl)-1*H*-benzo[*d*]imidazole (1ai)
Route A. White solid (1.98 g, 99% yield), m.p. 191–193 °C. **^{1}H NMR** (400 MHz, $CDCl_3$) δ 7.90 (d, J = 8.0 Hz, 1H), 7.73 (d, J = 8.2 Hz, 2H), 7.57 (d, J = 8.2 Hz, 2H), 7.52 – 7.43 (m, 3H), 7.37 – 7.23 (m, 5H), 1.33 (s, 12H). **^{13}C NMR** (100 MHz, $CDCl_3$) δ 152.4, 143.1, 137.4, 137.1, 134.7, 132.5, 130.0, 128.8, 128.7, 127.5, 123.6, 123.2, 120.1, 110.6, 84.1, 25.0. **HRMS(ESI)** m/z: $[M+H]^+$ Calcd. for $C_{25}H_{26}BN_2O_2$ 397.2082; Found 397.2087.

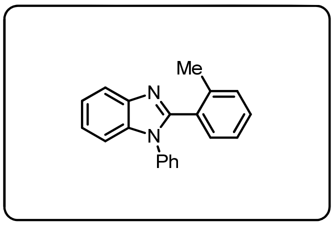

1-Phenyl-2-(*o*-tolyl)-1*H*-benzo[*d*]imidazole (1aj)
White solid (1.36 g, 96% yield), m.p. 103–105 °C. **^{1}H NMR** (400 MHz, $CDCl_3$) δ 7.90 (d, J = 7.8 Hz, 1H), 7.41 – 7.24 (m, 8H), 7.24 – 7.10 (m, 4H), 2.18 (s, 3H). **^{13}C NMR** (100 MHz, $CDCl_3$) δ 153.1, 143.2, 137.9, 136.5, 135.8, 131.0, 130.4, 130.3, 129.6, 129.6, 128.0, 126.7, 125.6, 123.4, 122.9, 120.2, 110.6, 20.1. **HRMS(ESI)** m/z: $[M+H]^+$ Calcd. for $C_{20}H_{17}N_2$ 285.1386; Found 285.1384.

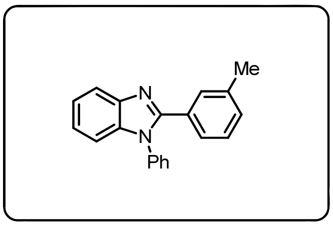

1-Phenyl-2-(*m*-tolyl)-1*H*-benzo[*d*]imidazole (1ak)
White solid (1.36 g, 96% yield), m.p. 69–71 °C. **^{1}H NMR** (400 MHz, $CDCl_3$) δ 7.89 (d, J = 8.0 Hz, 1H), 7.57 (s, 1H), 7.53 – 7.43 (m, 3H), 7.37 – 7.29 (m, 3H), 7.27 – 7.22 (d, J = 3.0 Hz, 2H), 7.20 – 7.09 (m, 3H), 2.31 (s, 3H). **^{13}C NMR** (100 MHz, $CDCl_3$) δ 152.7, 143.1, 138.3, 137.3, 137.2, 130.4, 130.3, 129.9, 129.9, 128.6, 128.1, 127.5, 126.5, 123.4, 123.0, 119.9, 110.5, 21.4. **HRMS(ESI)** m/z: $[M+H]^+$ Calcd. for $C_{20}H_{17}N_2$ 285.1386; Found 285.1385.

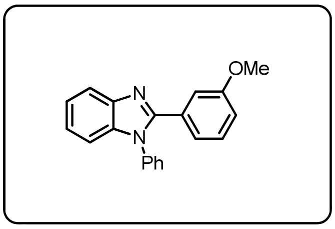

2-(3-Methoxyphenyl)-1-phenyl-1*H*-benzo[*d*]imidazole (1al)
White solid (1.44 g, 96% yield), m.p. 108–110 °C. **^{1}H NMR** (400 MHz, $CDCl_3$) δ 7.90 (d, J = 8.0 Hz, 1H), 7.55 – 7.43 (m, 3H), 7.37 – 7.30 (m, 3H), 7.29 – 7.22 (m, 2H), 7.21 – 7.08 (m, 3H), 6.92 – 6.87 (m, 1H), 3.68 (s, 3H). **^{13}C NMR** (100 MHz, $CDCl_3$) δ 159.4, 152.3, 143.0, 137.3, 137.2, 131.2, 130.0, 129.4, 128.7, 127.6, 123.5, 123.1, 122.0, 120.0, 116.3, 114.1, 110.6, 55.3. **HRMS(ESI)** m/z: $[M+H]^+$ Calcd. for $C_{20}H_{17}N_2O$ 301.1335; Found 301.1333.

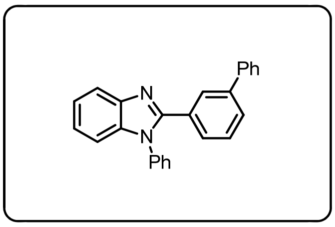

2-([1,1'-Biphenyl]-3-yl)-1-phenyl-1*H*-benzo[*d*]imidazole (1am)

White solid (1.66 g, 96% yield), m.p. 103–105 °C. **^{1}H NMR** (400 MHz, $CDCl_3$) δ 7.91 (d, J = 8.0 Hz, 1H), 7.77 (s, 1H), 7.63 – 7.50 (m, 5H), 7.43 – 7.27 (m, 11H). **^{13}C NMR** (100 MHz, $CDCl_3$) δ 152.3, 143.1, 141.2, 140.3, 137.3, 137.2, 130.4, 130.1, 128.9, 128.8, 128.7, 128.3, 128.2, 127.6, 127.6, 127.1, 123.5, 123.1, 120.0, 110.5, 100.0. **HRMS(ESI)** m/z: $[M+H]^+$ Calcd. for $C_{25}H_{19}N_2$ 347.1543; Found 347.1542.

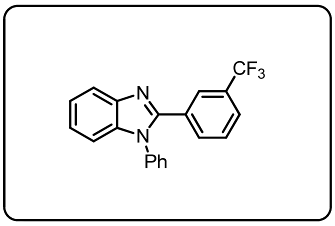

1-Phenyl-2-(3-(trifluoromethyl)phenyl)-1*H*-benzo[*d*]imidazole (1an)

White solid (1.66 g, 98% yield), m.p. 103–105 °C. **^{1}H NMR** (400 MHz, $CDCl_3$) δ 7.91 (d, J = 7.2 Hz, 2H), 7.71 (d, J = 7.8 Hz, 1H), 7.59 (d, J = 7.8 Hz, 1H), 7.55 – 7.47 (m, 3H), 7.43 – 7.33 (m, 2H), 7.32 – 7.23 (m, 4H). **^{13}C NMR** (100 MHz, $CDCl_3$) δ 150.7, 143.0, 137.4, 136.6, 132.5, 131.1 (q, J = 32.0 Hz), 131.0, 130.2, 129.1, 128.9, 127.5, 126.4 (q, J = 4.0 Hz), 126.1 (q, J = 4.0 Hz), 124.0, 123.8 (q, J = 156.0 Hz), 123.4, 120.2, 110.7. **^{19}F NMR** (376 MHz, $CDCl_3$) δ -63.0. **HRMS(ESI)** m/z: $[M+H]^+$ Calcd. for $C_{20}H_{14}F_3N_2$ 339.1104; Found 339.1099.

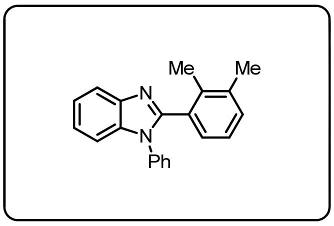

2-(2,3-Dimethylphenyl)-1-phenyl-1*H*-benzo[*d*]imidazole (1ao)

White solid (1.48 g, 99% yield), m.p. 122–124 °C. **^{1}H NMR** (400 MHz, $CDCl_3$) δ 7.90 (d, J = 7.9 Hz, 1H), 7.40 – 7.27 (m, 6H), 7.23 – 7.13 (m, 4H), 7.05 (t, J = 7.6 Hz, 1H), 2.21 (s, 3H), 2.04 (s, 3H). **^{13}C NMR** (100 MHz, $CDCl_3$) δ 153.7, 143.2, 137.3, 136.5, 136.4, 135.8, 131.1, 130.4, 129.5, 128.8, 128.0, 126.7, 125.3, 123.3, 122.9, 120.1, 110.6, 20.4, 17.1. **HRMS(ESI)** m/z: $[M+H]^+$ Calcd. for $C_{21}H_{19}N_2$ 299.1543; Found 299.1542.

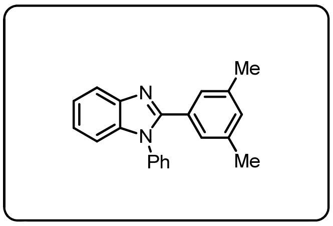

2-(3,5-Dimethylphenyl)-1-phenyl-1*H*-benzo[*d*]imidazole (1ap)

White solid (1.48 g, 99% yield), m.p. 91–93 °C. **^{1}H NMR** (400 MHz, $CDCl_3$) δ 7.88 (d, J = 8.0 Hz, 1H), 7.53 – 7.42 (m, 3H), 7.36 – 7.29 (m, 3H), 7.26 – 7.22 (m, 2H), 7.17 (s, 2H), 6.98 (s, 1H), 2.21 (s, 6H). **^{13}C NMR** (100 MHz, $CDCl_3$) δ 152.8, 143.1, 137.9, 137.3, 137.3, 131.2, 129.8, 129.8, 128.6, 127.6, 127.4, 123.3, 123.0, 119.9, 110.5, 21.3.

HRMS(ESI) m/z: $[M+H]^+$ Calcd. for $C_{21}H_{19}N_2$ 299.1543; Found 299.1542.

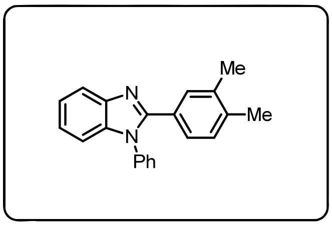

2-(3,4-Dimethylphenyl)-1-phenyl-1*H*-benzo[*d*]imidazole (1aq)

White solid (1.48 g, 99% yield), m.p. 140–142 °C. **^{1}H NMR** (400 MHz, $CDCl_3$) δ 7.88 (d, J = 8.0 Hz, 1H), 7.54 (s, 1H), 7.52 – 7.42 (m, 3H), 7.35 – 7.28 (m, 3H), 7.27 – 7.20 (m, 2H), 7.09 (dd, J = 7.8, 1.2 Hz, 1H), 6.98 (d, J = 7.8 Hz, 1H), 2.23 (s, 3H), 2.21 (s, 3H). **^{13}C NMR** (100 MHz, $CDCl_3$) δ 152.8, 143.1, 138.4, 137.3, 137.3, 136.9, 130.8, 129.9, 129.5, 128.5, 127.6, 127.4, 126.7, 123.2, 122.9, 119.8, 110.4, 19.8, 19.8. **HRMS(ESI)** m/z: $[M+H]^+$ Calcd. for $C_{21}H_{19}N_2$ 299.1543; Found 299.1542.

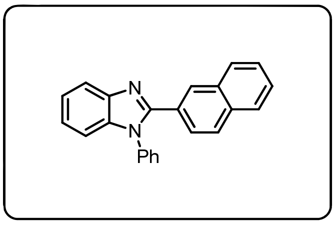

2-(Naphthalen-2-yl)-1-phenyl-1*H*-benzo[*d*]imidazole (1ar)

White solid (1.58 g, 99% yield), m.p. 166–168 °C. **^{1}H NMR** (400 MHz, $CDCl_3$) δ 8.12 (s, 1H), 7.93 (d, J = 8.0 Hz, 1H), 7.78 (d, J = 7.8 Hz, 1H), 7.73 (t, J = 7.4 Hz, 2H), 7.62 (d, J = 8.4 Hz, 1H), 7.53 – 7.42 (m, 5H), 7.39 – 7.32 (m, 3H), 7.31 – 7.25 (m, 2H). **^{13}C NMR** (100 MHz, $CDCl_3$) δ 152.5, 143.2, 137.5, 137.2, 133.6, 133.0, 130.0, 129.8, 128.7, 128.0, 127.8, 127.6, 127.4, 127.2, 126.6, 126.3, 123.5, 123.2, 120.0, 110.6. **HRMS(ESI)** m/z: $[M+H]^+$ Calcd. for $C_{23}H_{17}N_2$ 321.1386; Found 321.1382.

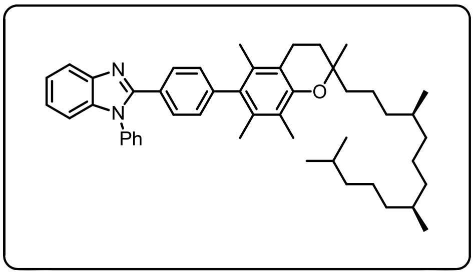

1-Phenyl-2-(4-(2,5,7,8-tetramethyl-2-((*4S,8S*)-4,8,12-trimethyltridecyl)chroman-6-yl)phenyl)-1*H*-benzo[*d*]imidazole (S10)

Path B. Colorless oil (6.14 g, 90% yield). **^{1}H NMR** (400 MHz, $CDCl_3$) δ 7.90 (d, J = 7.8 Hz, 1H), 7.59 (d, J = 8.2 Hz, 2H), 7.54 – 7.45 (m, 3H), 7.41 – 7.32 (m, 3H), 7.31 – 7.26 (m, 2H), 7.07 (d, J = 8.0 Hz, 2H), 2.61 (t, J = 6.4 Hz, 2H), 2.13 (s, 3H), 1.93 – 1.75 (m, 9H), 1.58 – 1.26 (m, 15H), 1.19 – 0.98 (m, 8H), 0.91 – 0.81 (m, 12H). **^{13}C NMR** (100 MHz, $CDCl_3$) δ 152.8, 151.0, 144.3, 143.2, 137.3, 137.2, 132.9, 132.7, 131.5, 130.4, 130.3, 129.9, 129.4, 128.7, 127.9, 127.5, 123.3, 123.1, 122.0, 119.9, 116.8, 110.6, 75.2, 40.4, 40.4, 39.5, 37.7, 37.7, 37.6, 37.6, 37.5, 37.5, 37.5, 37.5, 37.4, 32.9, 32.9, 32.8, 32.8, 31.4, 31.3, 28.1, 25.0, 24.9, 24.6, 24.1, 22.9, 22.8, 21.2, 20.9, 19.9, 19.8,

19.8, 19.8, 17.8, 16.8, 12.0. **HRMS(ESI)** m/z: $[M+H]^+$ Calcd. for $C_{48}H_{63}N_2O$ 683.4935; Found 683.4932.

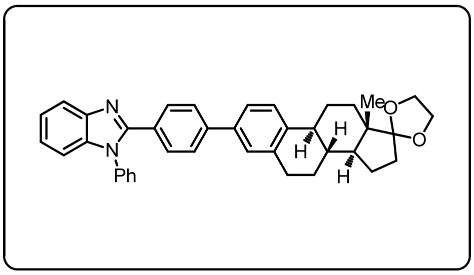

2-(4-((*8R,9S,13S,14S*)-13-Methyl-6,7,8,9,11,12,13,14,15,16-decahydrospiro[cyclopenta[*a*]phenanthrene-17,2'-[1,3]dioxolan]-3-yl)phenyl)-1-phenyl-1*H*-benzo[*d*]imidazole (S11)

Path B. Colorless oil (5.09 g, 90% yield). **^{1}H NMR** (400 MHz, $CDCl_3$) δ 7.91 (d, J = 8.0 Hz, 1H), 7.65 – 7.60 (m, 2H), 7.57 – 7.47 (m, 5H), 7.39 – 7.33 (m, 5H), 7.32 – 7.27 (m, 3H), 4.00 – 3.87 (m, 4H), 2.95 – 2.89 (m, 2H), 2.44 – 2.29 (m, 2H), 1.99 – 1.75 (m, 4H), 1.72 – 1.31 (m, 7H), 0.89 (s, 3H). **^{13}C NMR** (100 MHz, $CDCl_3$) δ 152.3, 143.2, 142.1, 140.4, 137.5, 137.4, 137.4, 137.2, 130.1, 129.8, 128.7, 128.5, 127.7, 127.6, 126.9, 126.1, 124.4, 123.4, 123.1, 119.9, 119.5, 110.5, 65.4, 64.7, 49.6, 46.2, 44.2, 38.9, 34.3, 30.8, 29.8, 27.1, 26.1, 22.5, 14.5. **HRMS(ESI)** m/z: $[M+H]^+$ Calcd. for $C_{39}H_{39}N_2O_2$ 567.3006; Found 567.3002.

Supplementary Note 2

Phosphine Oxide Ligand Synthesis

PO-1 to **PO-5** ligands have been reported in our previous works.[3,4] The synthesis of **PO-6** was shown below.

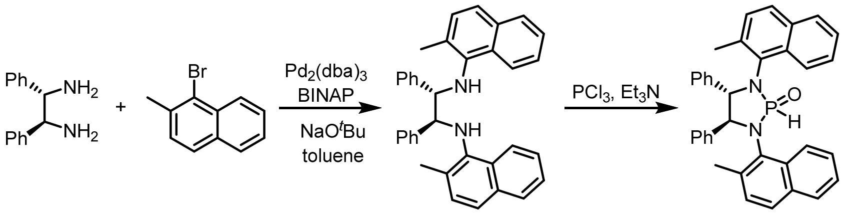

S-14 **S-15** **S-16** **PO-6**

A 48 mL schlenk flask was charged with $Pd_2(dba)_3$ (228.9 mg, 0.25 mmol), (±)-BINAP (187 mg, 0.3 mmol), NaO^{t}Bu (1.45 g, 15 mmol) and toluene (15 mL) and stirred for 20 min. **S-14** (1.06 g, 5 mmol) and **S-15** (2.43 g, 11 mmol) were then added and the solution was heated to 110 °C for 48 h. After cooling to room temperature, the resulting mixture was filtered through a pad of Celite and washed with DCM. The filtrate was concentrated and the residue was purified by flash chromatography to afford **S-16** as a white solid (1.67 g, 68%).

To a solution of **S-16** (2 mmol) and Et_3N (16 mmol, 8.0 equiv.) in DCM (5 mL) was added dropwise PCl_3 (4 mmol, 2 M in DCM, 2.0 equiv) at -78 °C. The reaction mixture was warmed to 23 °C and stirred overnight. After cooled to -78 °C again, the mixture was subjected to H_2O (2 mmol, 1.0 equiv.). The resulting mixture was warmed up to

23 °C and stirred at this temperature for 0.5 h. After removing all volatiles in vacuo, the residue was suspended in Et_2O (10 mL) and filtered through a pad of Celite eluting with Et_2O. The filtrate was evaporated in vacuo to afford the crude **PO-6**, which was further purified by column chromatography on silica gel.

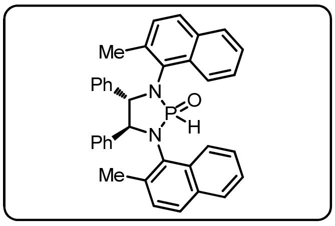

(*4S,5S*)-1,3-Bis(2-methylnaphthalen-1-yl)-4,5-diphenyl-1,3,2-diazaphospholidine 2-oxide (PO-6)

White solid (1.75 g, 65% yield), m.p. 142–144 °C. **^{1}H NMR** (400 MHz, $CDCl_3$) δ 9.20 – 8.81 (m, 1H), 8.60 – 8.20 (m, 1H), 7.95 – 7.50 (m, 6H), 7.47 – 7.25 (m, 7H), 7.24 – 6.90 (m, 7H), 6.01 – 5.19 (m, 2H), 3.15 – 2.27 (m, 6H). **^{13}C NMR** (100 MHz, $CDCl_3$) δ 139.5, 139.2, 136.9, 136.3, 136.2, 136.2, 135.8, 135.7, 135.7, 135.6, 135.2, 135.1, 134.3, 134.1, 134.0, 133.8, 133.7, 133.6, 133.5, 133.3, 133.3, 132.9, 132.8, 132.5, 132.4, 131.3, 131.2, 131.1, 130.8, 129.9, 129.6, 129.1, 129.1, 128.6, 128.5, 128.2, 128.2, 127.1, 127.0, 126.5, 126.3, 125.7, 125.3, 125.2, 125.1, 125.1, 124.3, 123.4, 122.1, 72.9, 72.5, 71.4, 71.0, 70.3, 69.5, 20.7, 20.1, 19.6.**^{31}P NMR** (162 MHz, $CDCl_3$) δ 8.3, 7.4, 5.6, 4.8, 4.7, 4.1. **HRMS(ESI)** m/z: $[M+H]^+$ Calcd. for $C_{36}H_{32}N_2OP$ 539.2247; Found 539.2246.

Supplementary Note 3

Traditional Hydroarylation of Alkynes with *γ*-C–H Bonds

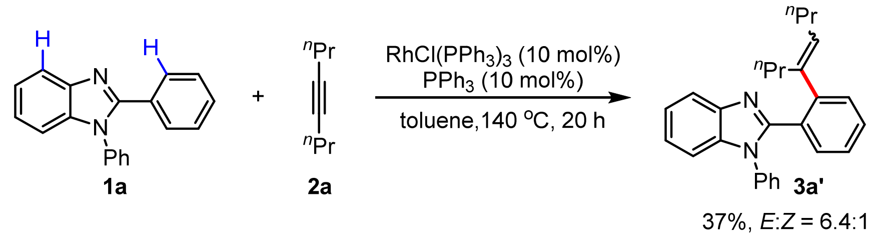

Following the reported procedure[1]: In an argon-filled glove-box, to an oven-dried sealed tube were added $RhCl(PPh_3)_3$ (37.0 mg, 0.04 mmol), PPh_3 (10.5 mg, 0.04 mmol), toluene (0.8 mL), **1a** (0.40 mmol) and **2a** (0.80 mmol) in sequence. The tube was then sealed, removed out of the glove-box and heated at 140 °C with heating mantle as the heat source for 12 h. Then the mixture was cooled to room temperature and concentrated in vacuo. The crude product was purified by column chromatography using ethyl acetate/hexane as eluent, providing product **3a′** in 37% yield (56.2 mg).

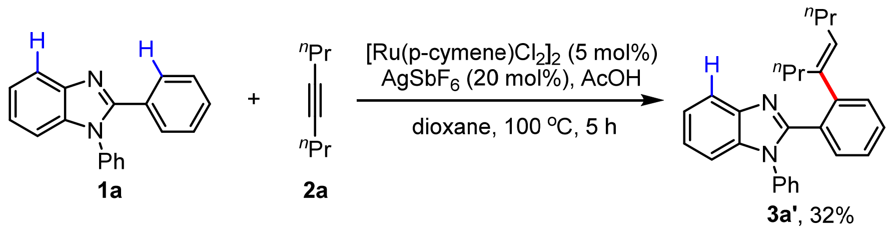

Following the reported procedure[2]: In an argon-filled glove-box, to an oven-dried sealed tube were added [Ru(p-cymene)Cl_2]$_2$ (12.2 mg, 0.02 mmol), $AgSbF_6$ (27.5 mg, 0.08 mmol), HOAc (91.5 μL, 1.6 mmol), dioxane (4.0 mL), **1a** (0.40 mmol) and **2a** (0.80 mmol) in sequence. The tube was then sealed, removed out of the glove-box and heated at 100 °C with heating mantle as the heat source for 5 h. Then the mixture was cooled to room temperature and concentrated in vacuo. The crude product was purified by column chromatography using ethyl acetate/hexane as eluent, providing product **3a′** in 32% yield (48.7 mg).

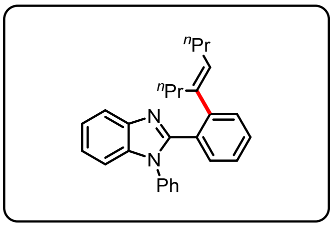

(*E*)-2-(2-(Oct-4-en-4-yl)phenyl)-1-phenyl-1*H*-benzo[*d*]imidazole (3a′)
Colorless oil. **^{1}H NMR** (400 MHz, $CDCl_3$) δ 7.89 (d, J = 7.9 Hz, 1H), 7.62 – 7.57 (m, 1H), 7.43 – 7.27 (m, 8H), 7.10 – 7.05 (m, 3H), 4.73 (t, J = 7.0 Hz, 1H), 1.79 (q, J = 7.2 Hz, 2H), 1.70 – 1.64 (m, 2H), 1.23 – 1.14 (m, 2H), 1.13 – 1.03 (m, 2H), 0.79 (t, J = 7.2 Hz, 3H), 0.66 (t, J = 7.2 Hz, 3H). **^{13}C NMR** (100 MHz, $CDCl_3$) δ 153.6, 145.0, 143.2, 140.0, 136.7, 135.6, 131.7, 131.7, 130.0, 129.8, 129.1, 128.5, 127.5, 126.6, 126.2, 123.2, 122.9, 120.2, 110.6, 32.2, 30.6, 22.7, 21.9, 14.0. **HRMS(ESI)** m/z: $[M+H]^+$ Calcd. for $C_{27}H_{29}N_2$ 381.2325; Found 381.2324.

Supplementary Note 4

Typical Procedure for *β*-C–H Bond-Involved Hydroarylation

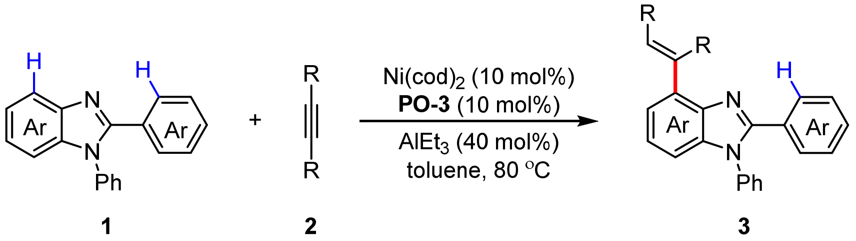

In an argon-filled glove-box, to an oven-dried sealed tube were added Ni(cod)$_2$ (11.0 mg, 0.04 mmol), **PO-3** (16.0 mg, 0.04 mmol), toluene (0.8 mL), **1** (0.40 mmol), $AlEt_3$ (1 mol/L in hexane, 160 μL, 0.16 mmol) and **2** (0.80 mmol) in sequence. The tube was then sealed, removed out of the glove-box and heated at 80 °C with heating mantle as the heat source for 12 h. Then the mixture was cooled to room temperature

and concentrated in vacuo. The crude product was purified by flash column chromatography using ethyl acetate/hexane as eluent.

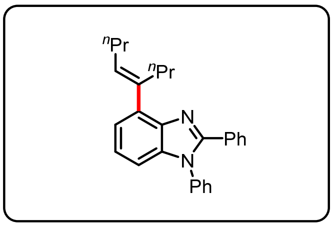

(*E*)-4-(Oct-4-en-4-yl)-1,2-diphenyl-1*H*-benzo[*d*]imidazole (3a)

White solid (126.3 mg, 83% yield), m.p. 84–86 °C. **^{1}H NMR** (400 MHz, $CDCl_3$) δ 7.59 – 7.54 (m, 2H), 7.52 – 7.42 (m, 3H), 7.35 – 7.24 (m, 5H), 7.22 – 7.15 (m, 2H), 7.13 – 7.06 (m, 1H), 6.06 (t, J = 7.2 Hz, 1H), 2.95 (t, J = 7.6, 2H), 2.31 (q, J = 7.2 Hz, 2H), 1.61 – 1.49 (m, 2H), 1.48 – 1.36 (m, 2H), 1.02 (t, J = 7.6 Hz, 3H), 0.93 (t, J = 7.6 Hz, 3H). **^{13}C NMR** (100 MHz, $CDCl_3$) δ 151.5, 140.9, 139.3, 137.6, 137.4, 136.2, 131.8, 130.5, 129.9, 129.7, 129.3, 128.5, 128.3, 127.7, 123.2, 122.2, 108.8, 32.5, 30.8, 23.3, 22.2, 14.3, 14.3. **HRMS(ESI)** m/z: $[M+H]^+$ Calcd. for $C_{27}H_{29}N_2$ 381.2325; Found 381.2324.

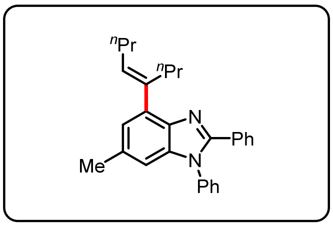

(*E*)-6-Methyl-4-(oct-4-en-4-yl)-1,2-diphenyl-1*H*-benzo[*d*]imidazole (3b)

Colorless oil (126.1 mg, 80% yield). **^{1}H NMR** (400 MHz, $CDCl_3$) δ 7.57 – 7.52 (m, 2H), 7.51 – 7.41 (m, 3H), 7.32 – 7.22 (m, 5H), 7.02 (d, J = 1.1 Hz, 1H), 6.88 (s, 1H), 6.02 (t, J = 7.2 Hz, 1H), 2.99 – 2.90 (m, 2H), 2.42 (s, 3H), 2.30 (q, J = 7.2 Hz, 2H), 1.60 – 1.48 (m, 2H), 1.47 – 1.36 (m, 2H), 1.02 (t, J = 7.4 Hz, 3H), 0.93 (t, J = 7.2 Hz, 3H). **^{13}C NMR** (100 MHz, $CDCl_3$) δ 151.1, 139.4, 139.1, 137.8, 137.5, 135.7, 133.11, 131.5, 130.6, 129.9, 129.6, 129.1, 128.4, 128.2, 127.7, 123.8, 108.7, 32.5, 30.8, 23.3, 22.2, 22.0, 14.3, 14.3. **HRMS(ESI)** m/z: $[M+H]^+$ Calcd. for $C_{28}H_{31}N_2$ 395.2482; Found 395.2480.

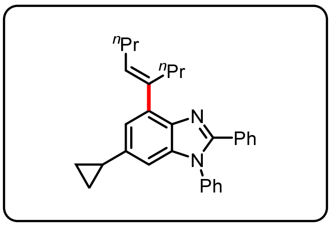

(*E*)-6-Cyclopropyl-4-(oct-4-en-4-yl)-1,2-diphenyl-1*H*-benzo[*d*]imidazole (3c)

Colorless oil (151.3 mg, 90% yield). **^{1}H NMR** (400 MHz, $CDCl_3$) δ 7.55 – 7.44 (m, 5H), 7.32 – 7.24 (m, 5H), 6.94 (d, J = 1.4 Hz, 1H), 6.78 (d, J = 1.2 Hz, 1H), 6.01 (t, J = 7.2 Hz, 1H), 2.92 (t, J = 8.0 Hz, 2H), 2.30 (q, J = 7.6 Hz, 2H), 2.02 – 1.92 (m, 1H), 1.60 – 1.49 (m, 2H), 1.46 – 1.36 (m, 2H), 1.02 (t, J = 7.4 Hz, 3H), 0.96 – 0.89 (m, 5H), 0.71 – 0.66 (m, 2H). **^{13}C NMR** (100 MHz, $CDCl_3$) δ 151.2, 139.4, 139.3, 137.8, 137.5, 135.8, 131.6, 131.5, 130.6, 129.9, 129.6, 129.1, 128.5, 128.2, 127.7, 120.8, 105.5, 32.5, 30.8,

23.31, 22.2, 16.0, 14.3, 9.3. **HRMS(ESI)** m/z: $[M+H]^+$ Calcd. for $C_{30}H_{33}N_2$ 421.2638; Found 421.2633.

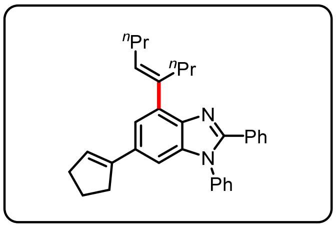

(*E*)-6-(Cyclopent-1-en-1-yl)-4-(oct-4-en-4-yl)-1,2-diphenyl-1*H*-benzo[*d*]imidazole (3d)

White solid (167.8 mg, 94% yield) , m.p. 89–91 °C. **^{1}H NMR** (400 MHz, $CDCl_3$) δ 7.57 – 7.52 (m, 2H), 7.52 – 7.45 (m, 3H), 7.37 (d, J = 1.5 Hz, 1H), 7.33 – 7.25 (m, 5H), 7.07 (d, J = 1.4 Hz, 1H), 6.18 – 6.14 (m, 1H), 6.03 (t, J = 7.2 Hz, 1H), 2.94 (t, J = 8.0 Hz, 2H), 2.75 – 2.67 (m, 2H), 2.58 – 2.48 (m, 2H), 2.31 (q, J = 7.2 Hz, 2H), 2.06 – 1.95 (m, 2H), 1.60 – 1.49 (m, 2H), 1.48 – 1.36 (m, 2H), 1.02 (t, J = 7.4 Hz, 3H), 0.93 (t, J = 7.4 Hz, 3H). **^{13}C NMR** (100 MHz, $CDCl_3$) δ 151.7, 143.1, 140.3, 139.5, 137.8, 137.4, 135.7, 132.5, 131.6, 131.5, 130.4, 129.9, 129.6, 129.2, 128.5, 128.3, 127.7, 125.6, 125.5, 120.6, 105.6, 33.8, 33.5, 32.5, 30.8, 23.5, 23.3, 22.2, 14.3, 14.3. **HRMS(ESI)** m/z: $[M+H]^+$ Calcd. for $C_{32}H_{35}N_2$ 447.2795; Found 447.2792.

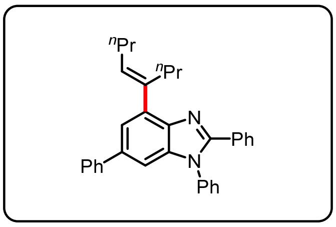

(*E*)-4-(Oct-4-en-4-yl)-1,2,6-triphenyl-1*H*-benzo[*d*]imidazole (3e)

White solid (138.7 mg, 76% yield), m.p. 157–159 °C. **^{1}H NMR** (400 MHz, $CDCl_3$) δ 7.59 (t, J = 7.8 Hz, 4H), 7.53 – 7.45 (m, 4H), 7.40 (t, J = 7.6 Hz, 2H), 7.36 – 7.24 (m, 7H), 6.11 (t, J = 7.2 Hz, 1H), 2.99 (t, J = 7.6 Hz, 2H), 2.33 (q, J = 7.2 Hz, 2H), 1.61 – 1.52 (m, 2H), 1.50 – 1.40 (m, 2H), 1.03 (t, J = 7.2 Hz, 3H), 0.95 (t, J = 7.2 Hz, 3H). **^{13}C NMR** (100 MHz, $CDCl_3$) δ 152.0, 142.2, 140.4, 139.3, 138.2, 137.3, 136.9, 136.3, 132.1, 130.3, 130.0, 129.7, 129.3, 128.8, 128.7, 128.3, 127.8, 127.6, 127.0, 122.2, 107.3, 32.5, 30.8, 23.3, 22.2, 14.3, 14.3. **HRMS(ESI)** m/z: $[M+H]^+$ Calcd. for $C_{33}H_{33}N_2$ 457.2638; Found 457.2635.

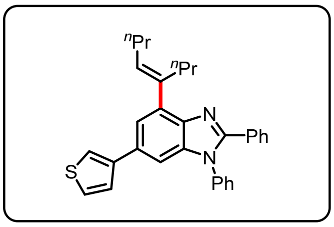

(*E*)-4-(Oct-4-en-4-yl)-1,2-diphenyl-6-(thiophen-3-yl)-1*H*-benzo[*d*]imidazole (3f)

White solid (151.6 mg, 82% yield), m.p. 134–136 °C. **^{1}H NMR** (400 MHz, $CDCl_3$) δ 7.59 – 7.54 (m, 2H), 7.53 – 7.44 (m, 4H), 7.42 – 7.39 (m, 1H), 7.39 – 7.31 (m, 5H), 7.31 – 7.25 (m, 3H), 6.08 (t, J = 7.2 Hz, 1H), 3.01 – 2.94 (t, J = 7.6 Hz, 2H), 2.33 (q, J = 7.6

Hz, 2H), 1.61 – 1.51 (m, 2H), 1.50 – 1.39 (m, 2H), 1.03 (t, *J* = 7.4 Hz, 3H), 0.94 (t, *J* = 7.4 Hz, 3H). **^{13}C NMR** (100 MHz, $CDCl_3$) *δ* 152.0, 143.2, 140.4, 139.3, 138.1, 137.3, 136.4, 131.9, 131.5, 130.3, 130.0, 129.7, 129.3, 128.7, 128.3, 127.7, 127.0, 126.1, 121.6, 120.0, 106.5, 32.5, 30.8, 23.3, 22.2, 14.3. **HRMS(ESI)** m/z: $[M+H]^+$ Calcd. for $C_{31}H_{31}N_2S$ 463.2202; Found 463.2197.

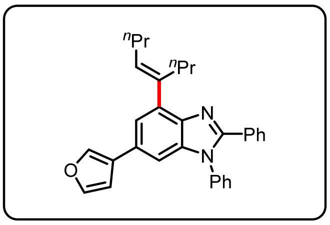

(*E*)-6-(Furan-3-yl)-4-(oct-4-en-4-yl)-1,2-diphenyl-1*H*-benzo[*d*]imidazole (3g)
Colorless oil (153.5 mg, 86% yield). **^{1}H NMR** (400 MHz, $CDCl_3$) *δ* 7.71 (s, 1H), 7.59 – 7.54 (m, 2H), 7.54 – 7.48 (m, 3H), 7.46 – 7.43 (m, 1H), 7.36 – 7.25 (m, 6H), 7.15 (d, *J* = 1.6 Hz, 1H), 6.71 – 6.67 (m, 1H), 6.05 (t, *J* = 7.2 Hz, 1H), 2.97 (t, *J* = 7.6 Hz, 2H), 2.32 (q, *J* = 7.6 Hz, 2H), 1.61 – 1.51 (m, 2H), 1.49 – 1.38 (m, 2H), 1.03 (t, *J* = 7.4 Hz, 3H), 0.94 (t, *J* = 7.2 Hz, 3H). **^{13}C NMR** (100 MHz, $CDCl_3$) *δ* 151.8, 143.6, 140.4, 139.3, 138.5, 138.1, 137.3, 136.5, 131.9, 130.4, 130.0, 129.7, 129.3, 128.7, 128.3, 127.8, 127.8, 127.2, 120.9, 109.4, 105.9, 32.5, 30.8, 23.3, 22.2, 14.3, 14.3. **HRMS(ESI)** m/z: $[M+H]^+$ Calcd. for $C_{31}H_{31}N_2O$ 447.2431; Found 447.2425.

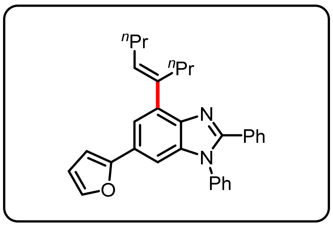

(*E*)-6-(Furan-2-yl)-4-(oct-4-en-4-yl)-1,2-diphenyl-1*H*-benzo[*d*]imidazole (3h)
Colorless oil (171.3 mg, 96% yield). **^{1}H NMR** (400 MHz, $CDCl_3$) *δ* 7.59 – 7.47 (m, 6H), 7.43 (d, *J* = 1.2 Hz, 1H), 7.38 (d, *J* = 1.2 Hz, 1H), 7.36 – 7.26 (m, 5H), 6.62 (d, *J* = 3.2 Hz, 1H), 6.47 – 6.43 (m, 1H), 6.06 (t, *J* = 7.2 Hz, 1H), 2.96 (t, *J* = 8.0 Hz, 2H), 2.32 (q, *J* = 7.2 Hz, 2H), 1.62 – 1.51 (m, 2H), 1.48 – 1.37 (m, 2H), 1.03 (t, *J* = 7.2 Hz, 3H), 0.94 (t, *J* = 7.2 Hz, 3H). **^{13}C NMR** (100 MHz, $CDCl_3$) *δ* 154.9, 152.1, 141.8, 140.7, 139.3, 138.0, 137.3, 136.5, 132.0, 130.3, 130.0, 129.7, 129.3, 128.7, 128.3, 127.8, 126.4, 119.0, 111.8, 104.6, 104.1, 32.5, 30.8, 23.3, 22.2, 14.3. **HRMS(ESI)** m/z: $[M+H]^+$ Calcd. for $C_{31}H_{31}N_2O$ 447.2431; Found 447.2425.

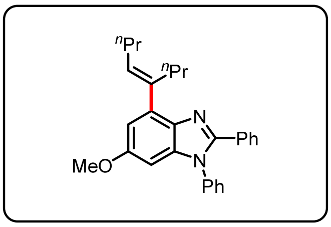

(*E*)-6-Methoxy-4-(oct-4-en-4-yl)-1,2-diphenyl-1*H*-benzo[*d*]imidazole (3i)
Colorless oil (137.8 mg, 84% yield). **^{1}H NMR** (400 MHz, $CDCl_3$) *δ* 7.56 – 7.44 (m, 5H), 7.34 – 7.23 (m, 5H), 6.86 (d, *J* = 2.4 Hz, 1H), 6.55 (d, *J* = 2.4 Hz, 1H), 6.10 (t, *J* = 7.2

Hz, 1H), 3.78 (s, 3H), 2.95 – 2.88 (t, J = 8.0 Hz, 2H), 2.30 (q, J = 7.6 Hz, 2H), 1.59 – 1.49 (m, 2H), 1.48 – 1.37 (m, 2H), 1.01 (t, J = 7.4 Hz, 3H), 0.93 (t, J = 7.2 Hz, 3H). **^{13}C NMR** (100 MHz, $CDCl_3$) δ 156.9, 150.7, 138.9, 138.1, 137.4, 136.8, 135.4, 132.2, 130.2, 130.0, 129.5, 129.0, 128.6, 128.5, 128.2, 127.7, 111.7, 91.9, 55.9, 32.38, 30.8, 23.2, 22.2, 14.3, 14.3. **HRMS(ESI)** m/z: $[M+H]^+$ Calcd. for $C_{28}H_{31}N_2O$ 411.2431; Found 411.2430.

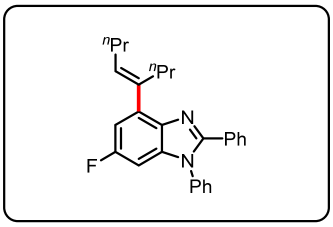

(*E*)-6-Fluoro-4-(oct-4-en-4-yl)-1,2-diphenyl-1*H*-benzo[*d*]imidazole (3j)

Colorless oil (137.0 mg, 86% yield). **^{1}H NMR** (400 MHz, $CDCl_3$) δ 7.58 – 7.44 (m, 5H), 7.32 – 7.27 (m, 5H), 6.96 (dd, J = 11.0, 2.4 Hz, 1H), 6.76 (dd, J = 8.4, 2.4 Hz, 1H), 6.15 (t, J = 7.2 Hz, 1H), 2.94 – 2.88 (t, J = 8.0 Hz, 2H), 2.30 (q, J = 7.3 Hz, 2H), 1.61 – 1.49 (m, 2H), 1.48 – 1.37 (m, 2H), 1.02 (t, J = 7.6 Hz, 3H), 0.93 (t, J = 7.2 Hz, 3H). **^{13}C NMR** (100 MHz, $CDCl_3$) δ 160.0 (d, J = 239.0 Hz), 152.0 (d, J = 6.0 Hz), 138.4, 137.7 (d, J = 14.0 Hz), 137.2 (d, J = 18.0 Hz), 137.2, 133.0, 130.2, 130.1, 129.6, 129.4, 128.8, 128.3, 127.7, 127.5, 110.3 (d, J = 25.0 Hz), 95.3 (d, J = 27.0 Hz), 32.3, 30.8, 23.2, 22.2, 14.3, 14.2. **^{19}F NMR** (376 MHz, $CDCl_3$) δ -118.8. **HRMS(ESI)** m/z: $[M+H]^+$ Calcd. for $C_{27}H_{28}FN_2$ 399.2231; Found 399.2229.

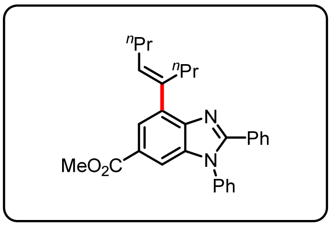

(*E*)-Methyl 4-(oct-4-en-4-yl)-1,2-diphenyl-1*H*-benzo[*d*]imidazole-6-carboxylate (3k)

White solid (143.8 mg, 82% yield), m.p. 174–176 °C. **^{1}H NMR** (400 MHz, $CDCl_3$) δ 7.91 (d, J = 1.4 Hz, 1H), 7.80 (d, J = 1.4 Hz, 1H), 7.58 (d, J = 7.2 Hz, 2H), 7.56 – 7.49 (m, 3H), 7.38 – 7.27 (m, 5H), 6.07 (t, J = 7.2 Hz, 1H), 3.91 (s, 3H), 3.00 (t, J = 8.0 Hz, 2H), 2.32 (q, J = 7.3 Hz, 2H), 1.59 – 1.49 (m, 2H), 1.46 – 1.34 (m, 2H), 1.03 (t, J = 7.4 Hz, 3H), 0.93 (t, J = 7.2 Hz, 3H). **^{13}C NMR** (100 MHz, $CDCl_3$) δ 167.8, 154.0, 144.4, 138.7, 137.5, 136.8, 135.9, 132.6, 130.1, 129.9, 129.8, 129.0, 128.4, 127.7, 124.9, 123.5, 111.0, 52.2, 32.3, 30.8, 23.2, 22.2, 14.3. **HRMS(ESI)** m/z: $[M+H]^+$ Calcd. for $C_{29}H_{31}N_2O_2$ 439.2380; Found 439.2377.

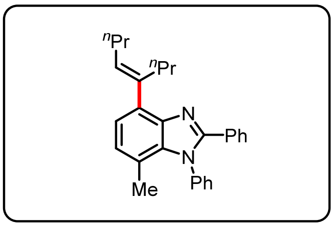

(*E*)-7-Methyl-4-(oct-4-en-4-yl)-1,2-diphenyl-1*H*-benzo[*d*]imidazole (3l)
Colorless oil (82.0 mg, 52% yield). **^{1}H NMR** (400 MHz, $CDCl_3$) δ 7.53 – 7.41 (m, 5H), 7.40 – 7.35 (m, 2H), 7.30 – 7.19 (m, 3H), 7.08 (d, J = 7.6 Hz, 1H), 6.91 (d, J = 8.0 Hz, 1H), 5.96 (t, J = 7.2 Hz, 1H), 2.97 – 2.87 (m, 2H), 2.29 (q, J = 7.6 Hz, 2H), 1.90 (s, 3H), 1.58 – 1.48 (m, 2H), 1.46 – 1.34 (m, 2H), 1.01 (t, J = 7.4 Hz, 3H), 0.92 (t, J = 7.2 Hz, 3H). **^{13}C NMR** (100 MHz, $CDCl_3$) δ 152.1, 141.0, 139.5, 138.7, 135.7, 134.3, 131.1, 130.8, 129.9, 129.8, 129.2, 129.1, 129.0, 128.1,125.6, 122.0, 120.0, 32.6, 30.7, 23.3, 22.2, 18.4, 14.3, 14.3. **HRMS(ESI)** m/z: $[M+H]^+$ Calcd. for $C_{28}H_{31}N_2$ 395.2482; Found 395.2481.

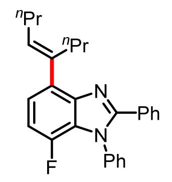

(*E*)-7-Fluoro-4-(oct-4-en-4-yl)-1,2-diphenyl-1*H*-benzo[*d*]imidazole (3m)
Colorless oil (106.8 mg, 67% yield). **^{1}H NMR** (400 MHz, $CDCl_3$) δ 7.54 – 7.49 (m, 2H), 7.46 – 7.39 (m, 3H), 7.38 – 7.22 (m, 5H), 7.07 (dd, J = 8.3, 4.8 Hz, 1H), 6.87 (dd, J = 11.2, 8.4 Hz, 1H), 5.94 (t, J = 7.2 Hz, 1H), 2.90 (t, J = 7.6 Hz, 2H), 2.29 (q, J = 7.2 Hz, 2H), 1.58 – 1.48 (m, 2H), 1.45 – 1.34 (m, 2H), 1.01 (t, J = 7.4 Hz, 3H), 0.92 (t, J = 7.2 Hz, 3H). **^{13}C NMR** (100 MHz, $CDCl_3$) δ 150.9 (d, J = 302.0 Hz), 146.95, 143.6 (d, J = 3.0 Hz), 138.81, 137.75, 132.5 (d, J = 5.0 Hz), 131.55, 129.94, 129.85, 129.47, 129.21, 128.83, 128.27, 128.17, 125.0 (d, J = 9.0 Hz), 121.8 (d, J = 6.0 Hz), 109.2 (d, J = 17.0 Hz), 32.6, 30.7, 23.3, 22.1, 14.2. **^{19}F NMR** (376 MHz, $CDCl_3$) δ -133.6. **HRMS(ESI)** m/z: $[M+H]^+$ Calcd. for $C_{27}H_{28}FN_2$ 399.2231; Found 399.2230.

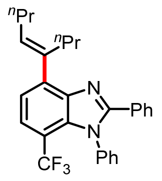

(*E*)-4-(Oct-4-en-4-yl)-1,2-diphenyl-7-(trifluoromethyl)-1*H*-benzo[*d*]imidazole (3n)
Colorless oil (139.8 mg, 78% yield). **^{1}H NMR** (400 MHz, $CDCl_3$) δ 7.51 (d, J = 7.8 Hz, 1H), 7.48 – 7.38 (m, 5H), 7.33 (d, J = 7.1 Hz, 2H), 7.29 – 7.19 (m, 5H), 5.99 (t, J = 7.0 Hz, 1H), 2.95 (t, J = 7.4 Hz, 2H), 2.31 (q, J = 7.6 Hz, 2H), 1.59 – 1.48 (m, 2H), 1.45 – 1.32 (m, 2H), 1.02 (t, J = 7.2 Hz, 3H), 0.92 (t, J = 7.2 Hz, 3H). **^{13}C NMR** (100 MHz, $CDCl_3$) δ 154.1, 142.6, 140.7, 139.0, 137.6, 133.6, 133.1, 130.4, 130.0, 129.9, 129.9, 129.5, 129.4, 129.0, 128.1, 123.5 (d, J = 270.0 Hz), 121.9 (q, J = 6.0 Hz), 121.3, 112.6 (d, J = 33.0 Hz), 32.5, 30.8, 23.2, 22.1, 14.2. **^{19}F NMR** (376 MHz, $CDCl_3$) δ -55.6. **HRMS(ESI)** m/z: $[M+H]^+$ Calcd. for $C_{28}H_{28}F_3N_2$ 449.2199; Found 449.2203.

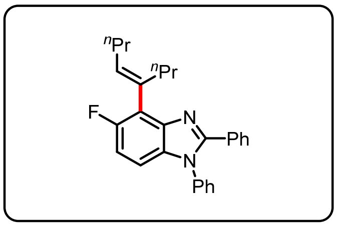

(*E*)-5-Fluoro-4-(oct-4-en-4-yl)-1,2-diphenyl-1*H*-benzo[*d*]imidazole (3o)
Colorless oil (76.5 mg, 48% yield). **^{1}H NMR** (400 MHz, $CDCl_3$) δ 7.59 – 7.42 (m, 5H), 7.35 – 7.23 (m, 5H), 7.02 – 6.95 (m, 2H), 5.75 (t, J = 7.2 Hz, 1H), 2.80 (t, J = 7.6 Hz, 2H), 2.33 (q, J = 7.2 Hz, 2H), 1.60 – 1.49 (m, 2H), 1.45 – 1.33 (m, 2H), 1.02 (t, J = 7.4 Hz, 3H), 0.93 (t, J = 7.4 Hz, 3H). **^{13}C NMR** (100 MHz, $CDCl_3$) δ 156.5 (d, J = 234.0 Hz), 152.9, 142.3 (d, J = 8.0 Hz), 137.1, 133.7, 133.7, 131.8, 130.2, 130.0, 129.7, 129.4, 128.7, 128.3, 127.6, 122.9 (d, J = 19.0 Hz), 111.8 (d, J = 28.0 Hz), 108.5 (d, J = 10.0 Hz), 33.1, 30.6, 23.1, 21.9, 14.2, 14.1. **^{19}F NMR** (376 MHz, $CDCl_3$) δ -124.6. **HRMS(ESI)** m/z: $[M+H]^+$ Calcd. for $C_{27}H_{28}FN_2$ 399.2231; Found 399.2228.

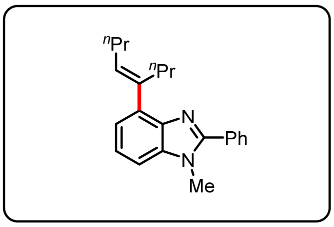

(*E*)-1-Methyl-4-(oct-4-en-4-yl)-2-phenyl-1*H*-benzo[*d*]imidazole (3p)
Colorless oil (95.5 mg, 75% yield). **^{1}H NMR** (400 MHz, $CDCl_3$) δ 7.80 – 7.71 (m, 2H), 7.56 – 7.45 (m, 3H), 7.30 – 7.24 (m, 2H), 7.21 – 7.14 (m, 1H), 5.97 (t, J = 7.2 Hz, 1H), 3.83 (s, 3H), 2.89 (t, J = 7.2 Hz, 2H), 2.27 (q, J = 7.6 Hz, 2H), 1.57 – 1.45 (m, 2H), 1.40 – 1.29 (m, 2H), 0.99 (t, J = 7.2 Hz, 3H), 0.88 (t, J = 7.2 Hz, 3H). **^{13}C NMR** (100 MHz, $CDCl_3$) δ 153.0, 140.9, 139.4, 136.8, 136.3, 131.5, 130.8, 129.8, 129.6, 128.7, 122.7, 121.9, 107.9, 32.5, 31.8, 30.7, 23.3, 22.2, 14.2. **HRMS(ESI)** m/z: $[M+H]^+$ Calcd. for $C_{22}H_{27}N_2$ 319.2169; Found 319.2166.

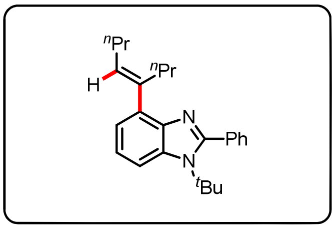

(*E*)-1-(tert-Butyl)-4-(oct-4-en-4-yl)-2-phenyl-1*H*-benzo[*d*]imidazole (3q)
White solid (112.4 mg, 78% yield), m.p. 153–155 °C. **^{1}H NMR** (400 MHz, $CDCl_3$) δ 7.61 (d, J = 8.4 Hz, 1H), 7.51 – 7.46 (m, 2H), 7.45 – 7.37 (m, 3H), 7.24 – 7.18 (m, 1H), 7.13 (d, J = 7.2 Hz, 1H), 5.77 (t, J = 7.2 Hz, 1H), 2.79 (t, J = 7.6 Hz, 2H), 2.22 (q, J = 7.6 Hz, 2H), 1.61 (s, 9H), 1.51 – 1.41 (m, 2H), 1.34 – 1.26 (m, 2H), 0.95 (t, J = 7.4 Hz, 3H), 0.86 (t, J = 7.2 Hz, 3H). **^{13}C NMR** (100 MHz, $CDCl_3$) δ 152.6, 141.3, 139.9, 136.7, 136.4, 135.1, 131.0, 130.2, 128.9, 127.7, 121.6, 121.6, 113.1, 58.7, 32.7, 31.6, 30.7, 23.2, 22.1, 14.2, 14.1. **HRMS(ESI)** m/z: $[M+H]^+$ Calcd. for $C_{25}H_{33}N_2$ 361.2638; Found 361.2633.

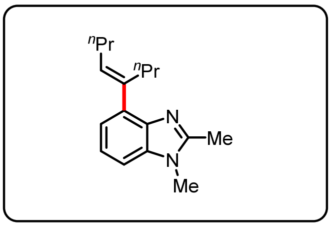

(E)-1,2-Dimethyl-4-(oct-4-en-4-yl)-1H-benzo[d]imidazole (3r)
Colorless oil (53.3 mg, 52% yield). **^{1}H NMR** (400 MHz, $CDCl_3$) δ 7.21 – 7.13 (m, 2H), 7.11 – 7.05 (m, 1H), 5.82 (t, J = 7.2 Hz, 1H), 3.70 (s, 3H), 2.85 – 2.76 (m, 2H), 2.60 (s, 3H), 2.26 (q, J = 7.6 Hz, 2H), 1.54 – 1.44 (m, 2H), 1.37 – 1.22 (m, 3H), 0.98 (t, J = 7.2 Hz, 3H), 0.85 (t, J = 7.2 Hz, 3H). **^{13}C NMR** (100 MHz, $CDCl_3$) δ 151.0, 140.3, 139.3, 135.9, 135.7, 131.2, 121.9, 121.6, 107.2, 32.6, 30.7, 30.0, 23.3, 22.1, 14.3, 14.2, 14.1. **HRMS(ESI)** m/z: $[M+H]^+$ Calcd. for $C_{17}H_{25}N_2$ 257.2012; Found 257.2010.

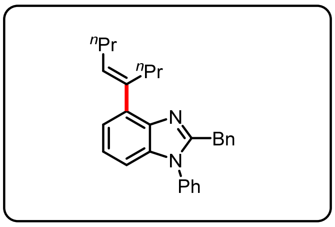

(E)-1,2-Dimethyl-4-(oct-4-en-4-yl)-1H-benzo[d]imidazole (3s)
Colorless oil (97.8 mg, 62% yield). **^{1}H NMR** (400 MHz, $CDCl_3$) δ 7.48 – 7.39 (m, 3H), 7.18 – 7.09 (m, 7H), 7.05 – 6.99 (m, 2H), 6.91 (dd, J = 7.6, 1.6 Hz, 1H), 5.96 (t, J = 7.2 Hz, 1H), 4.19 (s, 2H), 2.92 (t, J = 7.6 Hz, 2H), 2.31 (q, J = 7.6 Hz, 2H), 1.60 – 1.48 (m, 2H), 1.45 – 1.35 (m, 2H), 1.02 (t, J = 7.4 Hz, 3H), 0.92 (t, J = 7.2 Hz, 3H). **^{13}C NMR** (100 MHz, $CDCl_3$) δ 152.4, 140.5, 139.4, 137.2, 137.1, 136.3, 135.9, 131.5, 129.7, 128.9, 128.8, 128.4, 127.9, 126.6, 122.6, 121.9, 108.5, 34.4, 32.7, 30.7, 23.3, 22.2, 14.3, 14.3. **HRMS(ESI)** m/z: $[M+H]^+$ Calcd. for $C_{28}H_{31}N_2$ 395.2482; Found 395.2481.

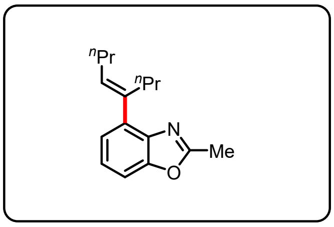

(E)-2-Methyl-4-(oct-4-en-4-yl)benzo[d]oxazole (3t)
Colorless oil (35.0 mg, 36% yield). **^{1}H NMR** (400 MHz, $CDCl_3$) δ 7.32 (d, J = 7.8 Hz, 1H), 7.23 – 7.14 (m, 3H), 5.89 (t, J = 7.2 Hz, 1H), 2.73 (t, J = 7.6 Hz, 2H), 2.63 (s, 3H), 2.25 (q, J = 7.6 Hz, 2H), 1.53 – 1.46 (m, 2H), 1.35 – 1.25 (m, 2H), 0.98 (t, J = 7.2 Hz, 3H), 0.86 (t, J = 7.2 Hz, 3H). **^{13}C NMR** (100 MHz, $CDCl_3$) δ 162.9, 151.1, 139.2, 138.0, 136.2, 132.3, 124.2, 123.3, 108.3, 32.3, 30.7, 23.2, 22.0, 14.8, 14.2, 14.1. **HRMS(ESI)** m/z: $[M+H]^+$ Calcd. for $C_{16}H_{22}NO$ 244.1696; Found 244.1696.

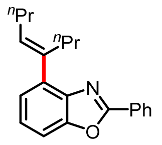

(*E*)-4-(Oct-4-en-4-yl)-2-phenylbenzo[*d*]oxazole (3u)

Colorless oil (78.1 mg, 64% yield). **^{1}H NMR** (400 MHz, $CDCl_3$) δ 8.27 (dd, J = 6.7, 3.0 Hz, 2H), 7.54 – 7.48 (m, 3H), 7.44 (dd, J = 7.5, 1.5 Hz, 1H), 7.30 – 7.22 (m, 2H), 6.09 (t, J = 7.2 Hz, 1H), 2.83 (t, J = 7.6 Hz, 2H), 2.30 (q, J = 7.2 Hz, 2H), 1.60 – 1.50 (m, 2H), 1.44 – 1.32 (m, 2H), 1.03 (t, J = 7.2 Hz, 3H), 0.90 (t, J = 7.2 Hz, 3H). **^{13}C NMR** (100 MHz, $CDCl_3$) δ 162.1, 151.0, 139.9, 138.0, 136.5, 132.7, 131.3, 128.9, 127.8, 127.7, 124.9, 123.4, 108.6, 32.1, 30.8, 23.2, 22.1, 14.2. **HRMS(ESI)** m/z: $[M+H]^+$ Calcd. for $C_{21}H_{24}$ NO 306.1852; Found 306.1852.

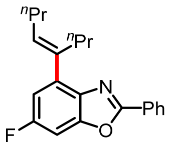

(*E*)-6-Fluoro-4-(oct-4-en-4-yl)-2-phenylbenzo[*d*]oxazole (3v)

Colorless oil (93.1 mg, 72% yield). **^{1}H NMR** (400 MHz, $CDCl_3$) δ 8.25 – 8.17 (m, 2H), 7.54 – 7.46 (m, 3H), 7.14 (dd, J = 7.7, 2.3 Hz, 1H), 7.03 – 6.97 (m, 1H), 6.17 (t, J = 7.2 Hz, 1H), 2.80 (t, J = 7.6 Hz, 2H), 2.29 (q, J = 7.2 Hz, 2H), 1.60 – 1.50 (m, 2H), 1.44 – 1.32 (m, 2H), 1.03 (t, J = 7.2 Hz, 3H), 0.91 (t, J = 7.2 Hz, 3H). **^{13}C NMR** (100 MHz, $CDCl_3$) δ 162.6 (d, J = 4.0 Hz), 160.6 (d, J = 241.0 Hz), 150.9 (d, J = 16.0 Hz), 137.1 (d, J = 2.0 Hz), 137.0, 136.2 (d, J = 2.0 Hz), 133.9 (d, J = 14.0 Hz), 131.4 (d, J = 5.0 Hz), 129.0 (d, J = 5.0 Hz), 127.6 (d, J = 9.0 Hz), 127.41, 111.1 (d, J = 24.0 Hz), 96.4 (dd, J = 28.0, 3.0 Hz), 31.9, 30.8, 23.1, 22.1, 14.2, 14.1. **^{19}F NMR** (376 MHz, $CDCl_3$) δ -116.1. **HRMS(ESI)** m/z: $[M+H]^+$ Calcd. for $C_{21}H_{23}FNO$ 324.1758; Found 324.1759.

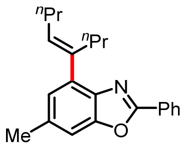

(*E*)-6-Methyl-4-(oct-4-en-4-yl)-2-phenylbenzo[*d*]oxazole (3w)

Colorless oil (84.3 mg, 66% yield). **^{1}H NMR** (400 MHz, $CDCl_3$) δ 8.28 – 8.20 (m, 2H), 7.52 – 7.46 (m, 3H), 7.24 (s, 1H), 7.05 (s, 1H), 6.03 (t, J = 7.2 Hz, 1H), 2.86 – 2.79 (m, 2H), 2.48 (s, 3H), 2.29 (q, J = 7.6 Hz, 2H), 1.59 – 1.49 (m, 2H), 1.42 – 1.33 (m, 2H), 1.02 (t, J = 7.2 Hz, 3H), 0.90 (t, J = 7.2 Hz, 3H). **^{13}C NMR** (100 MHz, $CDCl_3$) δ 161.6, 151.4, 138.2, 137.8, 135.9, 135.1, 132.4, 131.1, 128.8, 127.9, 127.7, 124.7, 108.9, 32.1, 30.8, 23.2, 22.1, 22.0, 14.2. **HRMS(ESI)** m/z: $[M+H]^+$ Calcd. for $C_{22}H_{26}NO$ 320.2009; Found 320.2013.

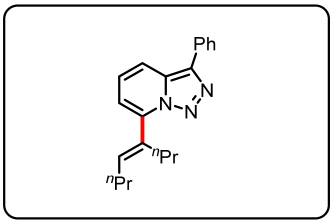

***(E)*-7-(Oct-4-en-4-yl)-3-phenyl-[1,2,3]triazolo[1,5-*a*]pyridine (3x)**

Colorless oil (87.6 mg, 72% yield). **^{1}H NMR** (400 MHz, $CDCl_3$) *δ* 7.98 (d, *J* = 7.4 Hz, 2H), 7.92 (d, *J* = 8.8 Hz, 1H), 7.51 (t, *J* = 7.6 Hz, 2H), 7.38 (t, *J* = 7.4 Hz, 1H), 7.28 (d, *J* = 6.9 Hz, 1H), 6.82 (d, *J* = 6.7 Hz, 1H), 6.03 (t, *J* = 7.2 Hz, 1H), 2.84 (t, *J* = 8.0 Hz, 2H), 2.32 (q, *J* = 7.6 Hz, 2H), 1.58 – 1.50 (m, 2H), 1.33 – 1.24 (m, 2H), 1.02 (t, *J* = 7.2 Hz, 3H), 0.88 (t, *J* = 7.2 Hz, 3H). **^{13}C NMR** (100 MHz, $CDCl_3$) *δ* 141.6, 137.6, 136.3, 134.8, 131.9, 131.1, 128.9, 127.7, 126.7, 125.9, 116.3, 114.6, 30.2, 30.2, 22.6, 21.7, 14.0, 13.9. **HRMS(ESI)** m/z: $[M+H]^+$ Calcd. for $C_{20}H_{23}N_3$ 306.1965; Found 306.1966.

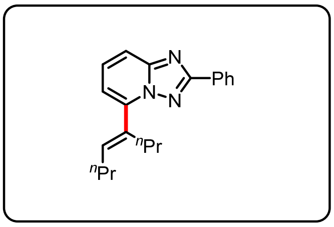

(*E*)-5-(Oct-4-en-4-yl)-2-phenyl-[1,2,4]triazolo[1,5-*a*]pyridine (3y-mono)

Colorless oil (58.8 mg, 48% yield). **^{1}H NMR** (400 MHz, $CDCl_3$) *δ* 8.47 (d, *J* = 6.6 Hz, 1H), 8.31 (d, *J* = 6.6 Hz, 2H), 7.51 – 7.44 (m, 3H), 7.34 (d, *J* = 7.1 Hz, 1H), 6.94 (t, *J* = 7.0 Hz, 1H), 6.40 (t, *J* = 7.2 Hz, 1H), 2.83 (t, *J* = 8.0 Hz, 2H), 2.31 (q, *J* = 7.4 Hz, 2H), 1.60 – 1.50 (m, 2H), 1.42 – 1.34 (m, 2H), 1.03 (t, *J* = 7.4 Hz, 3H), 0.91 (t, *J* = 7.4 Hz, 3H). **^{13}C NMR** (100 MHz, $CDCl_3$) *δ* 163.7, 150.9, 136.4, 134.5, 132.6, 131.3, 130.0, 128.7, 127.5, 126.5, 126.3, 113.6, 31.3, 30.8, 23.1, 22.1, 14.2, 14.2. **HRMS(ESI)** m/z: $[M+H]^+$ Calcd. for $C_{20}H_{24}$ N_3 306.1965; Found 306.1967.

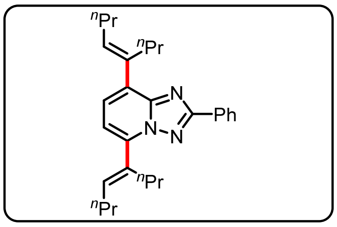

5,8-Di((*E*)-oct-4-en-4-yl)-2-phenyl-[1,2,4]triazolo[1,5-*a*]pyridine (3y-di)

Colorless oil (79.9 mg, 48% yield). **^{1}H NMR** (400 MHz, $CDCl_3$) *δ* 8.33 (d, *J* = 6.8 Hz, 2H), 7.49 – 7.38 (m, 3H), 7.29 (d, *J* = 7.4 Hz, 1H), 6.76 (d, *J* = 7.4 Hz, 1H), 6.37 (t, *J* = 7.2 Hz, 1H), 5.98 (t, *J* = 7.2 Hz, 1H), 2.83 (t, *J* = 7.6 Hz, 4H), 2.31 (q, *J* = 7.2 Hz, 4H), 1.60 – 1.49 (m, 4H), 1.44 – 1.34 (m, 2H), 1.33 – 1.23 (m, 2H), 1.06 – 1.00 (m, 6H), 0.94 – 0.84 (m, 6H). **^{13}C NMR** (100 MHz, $CDCl_3$) *δ* 162.9, 151.2, 141.9, 136.7, 135.4, 135.4, 133.7, 131.8, 130.2, 129.7, 128.5, 127.7, 126.7, 113.3, 31.3, 30.8, 30.4, 23.1, 22.8, 22.2, 21.9, 14.2, 14.1. **HRMS(ESI)** m/z: $[M+H]^+$ Calcd. for $C_{28}H_{38}$ N_3 416.3060; Found 416.3063.

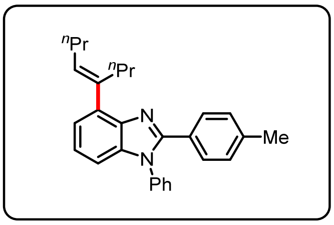

(*E*)-4-(Oct-4-en-4-yl)-1-phenyl-2-(p-tolyl)-1*H*-benzo[*d*]imidazole (4a)
White solid (126.2 mg, 80% yield), m.p. 108–110 °C. **^{1}H NMR** (400 MHz, $CDCl_3$) δ 7.51 – 7.42 (m, 5H), 7.33 – 7.27 (m, 2H), 7.21 – 7.13 (m, 2H), 7.11 – 7.04 (m, 3H), 6.07 (t, J = 7.2 Hz, 1H), 2.94 (t, J = 7.2 Hz, 2H), 2.35 – 2.27 (m, 5H), 1.60 – 1.49 (m, 2H), 1.47 – 1.36 (m, 2H), 1.02 (t, J = 7.6 Hz, 3H), 0.93 (t, J = 7.2 Hz, 3H). **^{13}C NMR** (100 MHz, $CDCl_3$) δ 151.7, 141.0, 139.4, 139.3, 137.6, 136.1, 131.7, 129.9, 129.6, 129.0, 128.5, 127.7, 127.7, 123.0, 122.1, 108.7, 32.5, 30.8, 23.3, 22.2, 21.5, 14.3, 14.3. **HRMS(ESI)** m/z: $[M+H]^+$ Calcd. for $C_{28}H_{31}N_2$ 395.2482; Found 395.2478.

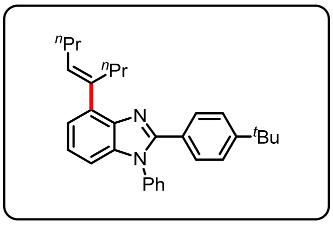

(*E*)-2-(4-(*tert*-Butyl)phenyl)-4-(oct-4-en-4-yl)-1-phenyl-1*H*-benzo[*d*]imidazole (4b)
Colorless oil (148.4 mg, 85% yield). **^{1}H NMR** (400 MHz, $CDCl_3$) δ 7.54 – 7.42 (m, 5H), 7.36 – 7.26 (m, 4H), 7.21 – 7.12 (m, 2H), 7.09 – 7.03 (m, 1H), 6.08 (t, J = 7.2 Hz, 1H), 2.95 (t, J = 7.6 Hz, 2H), 2.31 (q, J = 7.6 Hz, 2H), 1.61 – 1.49 (m, 2H), 1.46 – 1.38 (m, 2H), 1.27 (s, 9H), 1.02 (t, J = 7.2 Hz, 3H), 0.92 (t, J = 7.2 Hz, 3H). **^{13}C NMR** (100 MHz, $CDCl_3$) δ 152.4, 151.5, 141.0, 139.4, 137.7, 137.6, 136.0, 131.7, 129.9, 129.3, 128.5, 127.8, 127.6, 125.3, 123.0, 122.1, 108.7, 34.8, 32.5, 31.3, 30.8, 23.3, 22.2, 14.3. **HRMS(ESI)** m/z: $[M+H]^+$ Calcd. for $C_{31}H_{37}N_2$ 437.2951; Found 437.2950.

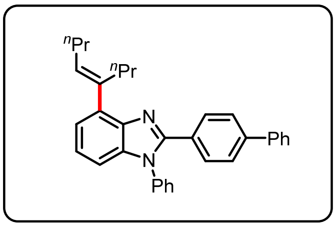

(*E*)-2-([1,1'-Biphenyl]-4-yl)-4-(oct-4-en-4-yl)-1-phenyl-1*H*-benzo[*d*]imidazole (4c)
White solid (138.7 mg, 76% yield), m.p. 97–99 °C. **^{1}H NMR** (400 MHz, $CDCl_3$) δ 7.58 (s, 1H), 7.56 (s, 1H), 7.49 (d, J = 7.2 Hz, 2H), 7.46 – 7.38 (m, 5H), 7.37 – 7.31 (m, 2H), 7.27 – 7.24 (m, 3H), 7.17 – 7.07 (m, 2H), 7.03 – 6.97 (m, 1H), 6.01 (t, J = 7.2 Hz, 1H), 2.89 (t, J = 7.6 Hz, 2H), 2.24 (q, J = 7.2 Hz, 2H), 1.54 – 1.42 (m, 2H), 1.41 – 1.30 (m, 2H), 0.95 (t, J = 7.4 Hz, 3H), 0.86 (t, J = 7.3 Hz, 3H). **^{13}C NMR** (100 MHz, $CDCl_3$) δ 151.2, 141.9, 141.1, 140.5, 139.3, 137.8, 137.6, 136.2, 131.8, 130.1, 130.0, 129.4, 128.9, 128.6, 127.8, 127.2, 126.9, 123.2, 122.2, 108.8, 100.1, 32.5, 30.8, 23.3, 22.3, 14.3, 14.3. **HRMS(ESI)** m/z: $[M+H]^+$ Calcd. for $C_{33}H_{33}N_2$ 457.2638; Found 457.2634.

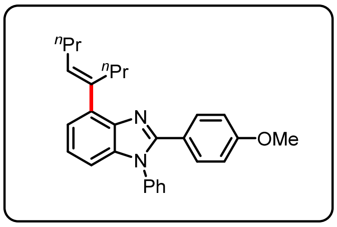

(*E*)-2-(4-Methoxyphenyl)-4-(oct-4-en-4-yl)-1-phenyl-1*H*-benzo[*d*]imidazole (4d)
White solid (134.6 mg, 82% yield), m.p. 110–112 °C. **^{1}H NMR** (400 MHz, $CDCl_3$) δ 7.57 – 7.39 (m, 7H), 7.29 (d, J = 7.8 Hz, 2H), 7.21 – 7.11 (m, 2H), 7.05 (d, J = 7.6 Hz, 1H), 6.78 (d, J = 8.8 Hz, 2H), 6.07 (t, J = 7.2 Hz, 1H), 3.76 (s, 3H), 2.95 (t, J = 7.6 Hz, 2H), 2.31 (q, J = 7.2 Hz, 2H), 1.60 – 1.50 (m, 2H), 1.48 – 1.36 (m, 2H), 1.02 (t, J = 7.2 Hz, 3H), 0.93 (t, J = 7.2 Hz, 3H). **^{13}C NMR** (100 MHz, $CDCl_3$) δ 160.4, 151.5, 140.9, 139.3, 137.6, 135.9, 131.6, 131.1, 129.9, 128.9, 128.4, 127.7, 122.9, 122.8, 122.1, 113.7, 108.6, 55.3, 32.5, 30.8, 23.3, 22.2, 14.3, 14.2. **HRMS(ESI)** m/z: $[M+H]^+$ Calcd. for $C_{28}H_{31}N_2O$ 411.2431; Found 411.2428.

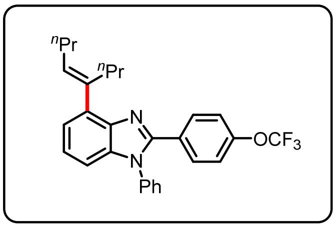

(*E*)-4-(Oct-4-en-4-yl)-1-phenyl-2-(4-(trifluoromethoxy)phenyl)-1*H*-benzo[*d*]imidazole (4e)
White solid (107.7 mg, 58% yield), m.p. 92–94 °C. **^{1}H NMR** (400 MHz, $CDCl_3$) δ 7.53 (d, J = 8.8 Hz, 2H), 7.47 – 7.37 (m, 3H), 7.26 – 7.19 (m, 2H), 7.16 – 7.07 (m, 2H), 7.07 – 6.95 (m, 3H), 5.99 (t, J = 7.2 Hz, 1H), 2.85 (t, J = 7.6 Hz, 2H), 2.23 (q, J = 7.2 Hz, 2H), 1.54 – 1.41 (m, 2H), 1.40 – 1.28 (m, 2H), 0.95 (t, J = 7.2 Hz, 3H), 0.85 (t, J = 7.6 Hz, 3H). **^{13}C NMR** (100 MHz, $CDCl_3$) δ 150.0, 149.9 (q, J = 1.7 Hz),140.9, 139.1, 137.7, 137.2, 136.3, 132.0, 131.2, 130.1, 129.2, 128.9, 127.7, 123.5, 122.4, 120.6, 120.5 (q, J = 256.1 Hz), 108.8, 32.5, 30.8, 23.3, 22.2, 14.3, 14.2. **HRMS(ESI)** m/z: $[M+H]^+$ Calcd. for $C_{28}H_{28}F_3N_2O$ 465.2148; Found 465.2147.

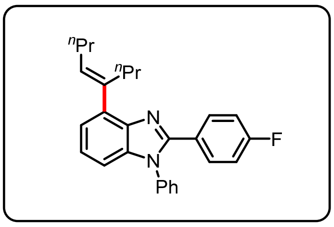

(*E*)-2-(4-Fluorophenyl)-4-(oct-4-en-4-yl)-1-phenyl-1*H*-benzo[*d*]imidazole (4f)
White solid (132.2 mg, 83% yield), m.p. 84–86 °C. **^{1}H NMR** (400 MHz, $CDCl_3$) δ 7.51 – 7.35 (m, 5H), 7.24 – 7.18 (m, 2H), 7.15 – 7.06 (m, 2H), 7.02 – 6.97 (m, 1H), 6.93 – 6.84 (m, 2H), 5.98 (t, J = 7.2 Hz, 1H), 2.86 (t, J = 7.6 Hz, 2H), 2.23 (q, J = 7.2 Hz, 2H), 1.52 – 1.41 (m, 2H), 1.39 – 1.28 (m, 2H), 0.94 (t, J = 7.6 Hz, 3H), 0.85 (t, J = 7.2 Hz, 3H). **^{13}C NMR** (100 MHz, $CDCl_3$) δ 163.4 (q, J = 248.4 Hz), 150.6, 140.9, 139.2, 137.5 (q, J = 28.6 Hz), 136.2, 131.9 131.7, 131.6, 130.0, 128.7, 127.7, 126.7 (q, J = 3.1 Hz),

123.3, 122.3, 115.4 (q, J = 21.7 Hz), 108.8, 32.5, 30.8, 23.3, 22.2, 14.3, 14.2. **HRMS(ESI)** m/z: $[M+H]^+$ Calcd. for $C_{27}H_{28}FN_2$ 399.2231; Found 399.2229.

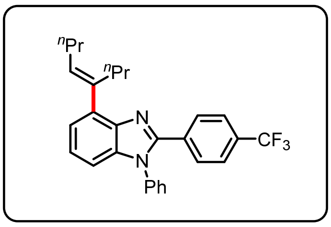

(*E*)-4-(Oct-4-en-4-yl)-1-phenyl-2-(4-(trifluoromethyl)phenyl)-1*H*-benzo[*d*]imidazole (4g)

White solid (100.4 mg, 56% yield), m.p. 101–103 °C. **^{1}H NMR** (400 MHz, $CDCl_3$) δ 7.71 (s, 1H), 7.69 (s, 1H), 7.57 – 7.48 (m, 5H), 7.34 – 7.29 (m, 2H), 7.24 – 7.18 (m, 2H), 7.13 – 7.07 (m, 1H), 6.08 (t, J = 7.2 Hz, 1H), 2.97 – 2.90 (t, J = 8.0 Hz, 2H), 2.32 (q, J = 7.6 Hz, 2H), 1.61 – 1.49 (m, 2H), 1.48 – 1.36 (m, 2H), 1.03 (t, J = 7.8 Hz, 3H), 0.93 (t, J = 7.6 Hz, 3H). **^{13}C NMR** (100 MHz, $CDCl_3$) δ 149.8, 140.9, 139.1, 137.8, 137.1, 136.5, 134.0, 132.1, 130.9 (q, J = 33.0 Hz), 130.2, 129.9, 129.0, 127.7, 125.4, 125.3 (q, J = 3.7 Hz), 123.9, 122.5, 108.9, 32.49, 30.8, 23.3, 22.2, 14.3, 14.2. **^{19}F NMR** (377 MHz, $CDCl_3$) δ -62.8. **HRMS(ESI)** m/z: $[M+H]^+$ Calcd. for $C_{28}H_{28}F_3N_2$ 449.2199; Found 449.2196.

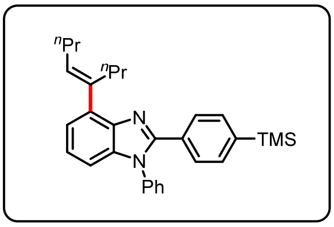

(*E*)-4-(Oct-4-en-4-yl)-1-phenyl-2-(4-(trimethylsilyl)phenyl)-1*H*-benzo[*d*]imidazole (4h)

White solid (144.7 mg, 80% yield), m.p. 109–111 °C. **^{1}H NMR** (400 MHz, $CDCl_3$) δ 7.56 (s, 1H), 7.54 (s, 1H), 7.53 – 7.45 (m, 3H), 7.43 (s, 1H), 7.41 (s, 1H), 7.35 – 7.30 (m, 2H), 7.22 – 7.14 (m, 2H), 7.10 – 7.04 (m, 1H), 6.09 (t, J = 7.2 Hz, 1H), 2.95 (t, J = 7.2 Hz, 2H), 2.31 (q, J = 7.2 Hz, 2H), 1.61 – 1.49 (m, 2H), 1.48 – 1.37 (m, 2H), 1.03 (t, J = 7.2 Hz, 3H), 0.92 (t, J = 7.2 Hz, 3H), 0.23 (s, 9H). **^{13}C NMR** (100 MHz, $CDCl_3$) δ 151.5, 142.0, 141.0, 139.3, 137.8, 137.6, 136.1, 133.2, 131.8, 130.7, 129.9, 128.7, 128.6, 127.8, 123.2, 122.1, 108.8, 32.5, 30.8, 23.3, 22.2, 14.3, 14.3, -1.1. **HRMS(ESI)** m/z: $[M+H]^+$ Calcd. for $C_{30}H_{37}N_2Si$ 453.2721; Found 453.2721.

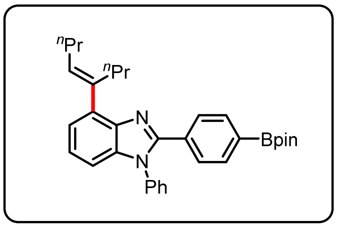

(*E*)-4-(Oct-4-en-4-yl)-1-phenyl-2-(4-(4,4,5,5-tetramethyl-1,3,2-dioxaborolan-2-yl)phenyl)-1*H*-benzo[*d*]imidazole (4i)

White solid (143.8 mg, 71% yield), m.p. 88–90 °C. **^{1}H NMR** (400 MHz, $CDCl_3$) δ 7.70

(d, J = 7.6 Hz, 2H), 7.56 (d, J = 8.0 Hz, 2H), 7.50 – 7.39 (m, 4H), 7.34 – 7.24 (m, 3H), 7.23 – 7.14 (m, 2H), 7.12 – 7.06 (m, 1H), 6.08 (t, J = 6.8 Hz, 1H), 2.94 (t, J = 7.2 Hz, 2H), 2.31 (q, J = 7.2 Hz, 2H), 1.58 – 1.51 (m, 2H), 1.46 – 1.30 (m, 14H), 1.02 (t, J = 7.2 Hz, 3H), 0.92 (t, J = 7.2 Hz, 3H). **^{13}C NMR** (100 MHz, $CDCl_3$) δ 151.4, 141.0, 139.3, 137.7, 137.5, 136.3, 134.6, 133.0, 131.9, 129.9, 128.9, 128.5, 127.7, 123.3, 122.2, 108.8, 84.1, 32.5, 30.8, 29.9, 25.0, 23.3, 22.3, 14.3, 14.2. **HRMS(ESI)** m/z: $[M+H]^+$ Calcd. for $C_{33}H_{40}BN_2O_2$ 507.3177; Found 507.3183.

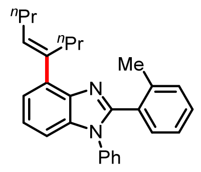

(*E*)-4-(Oct-4-en-4-yl)-1-phenyl-2-(o-tolyl)-1*H*-benzo[*d*]imidazole (4j)

Colorless oil (121.4 mg, 77% yield). **^{1}H NMR** (400 MHz, $CDCl_3$) δ 7.40 – 7.28 (m, 3H), 7.26 – 7.15 (m, 8H), 7.06 (t, J = 7.2 Hz, 1H), 5.97 (t, J = 7.2 Hz, 1H), 2.92 (t, J = 8.0 Hz, 2H), 2.34 – 2.25 (m, 5H), 1.57 – 1.46 (m, 2H), 1.45 – 1.34 (m, 2H), 0.99 (t, J = 7.2 Hz, 3H), 0.90 (t, J = 7.2 Hz, 3H). **^{13}C NMR** (100 MHz, $CDCl_3$) δ 151.8, 140.9, 139.4, 138.3, 136.8, 136.4, 136.0, 131.6, 131.2, 130.5, 130.4, 129.5, 129.4, 127.9, 126.9, 125.4, 123.1, 122.3, 108.8, 32.7, 30.7, 23.3, 22.1, 20.4, 14.3, 14.2. **HRMS(ESI)** m/z: $[M+H]^+$ Calcd. for $C_{28}H_{31}N_2$ 395.2482; Found 395.2481.

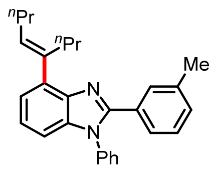

(*E*)-4-(Oct-4-en-4-yl)-1-phenyl-2-(m-tolyl)-1*H*-benzo[*d*]imidazole (4k)

Colorless oil (127.8 mg, 81% yield). **^{1}H NMR** (400 MHz, $CDCl_3$) δ 7.52 (s, 1H), 7.50 – 7.40 (m, 3H), 7.32 – 7.26 (m, 2H), 7.22 – 7.14 (m, 3H), 7.14 – 7.05 (m, 3H), 6.05 (t, J = 7.2 Hz, 1H), 2.95 (t, J = 7.6 Hz, 2H), 2.36 – 2.25 (m, 5H), 1.60 – 1.49 (m, 2H), 1.47 – 1.36 (m, 2H), 1.02 (t, J = 7.3 Hz, 3H), 0.93 (t, J = 7.3 Hz, 3H). **^{13}C NMR** (100 MHz, $CDCl_3$) δ 151.7, 140.9, 139.3, 138.0, 137.5, 137.5, 136.2, 131.7, 130.6, 130.3, 130.0, 129.8, 128.4, 128.0, 127.7, 126.7, 123.1, 122.2, 108.8, 32.5, 30.8, 23.3, 22.2, 21.5, 14.3, 14.3. **HRMS(ESI)** m/z: $[M+H]^+$ Calcd. for $C_{28}H_{31}N_2$ 395.2482; Found 395.2478.

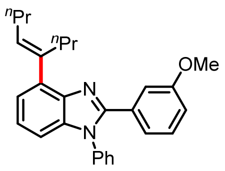

(*E*)-2-(3-Methoxyphenyl)-4-(oct-4-en-4-yl)-1-phenyl-1*H*-benzo[*d*]imidazole (4l)

Colorless oil (114.9 mg, 70% yield). **^{1}H NMR** (400 MHz, $CDCl_3$) δ 7.53 – 7.40 (m, 3H), 7.34 – 7.27 (m, 2H), 7.25 – 7.05 (m, 6H), 6.89 – 6.82 (m, 1H), 6.09 (t, J = 7.2 Hz, 1H),

3.66 (s, 3H), 2.94 (t, J = 8.0 Hz, 2H), 2.31 (q, J = 7.2 Hz, 2H), 1.60 – 1.50 (m, 2H), 1.48 – 1.38 (m, 2H), 1.03 (t, J = 7.6 Hz, 3H), 0.93 (t, J = 7.2 Hz, 3H). **^{13}C NMR** (100 MHz, $CDCl_3$) δ 159.3, 151.3, 140.8, 139.2, 137.6, 137.5, 136.1, 131.8, 131.6, 129.9, 129.3, 128.5, 127.7, 123.2, 122.2, 115.8, 114.5, 108.8, 55.2, 32.5, 30.8, 23.3, 22.2, 14.3, 14.2. **HRMS(ESI)** m/z: $[M+H]^+$ Calcd. for $C_{28}H_{31}N_2O$ 411.2431; Found 411.2430.

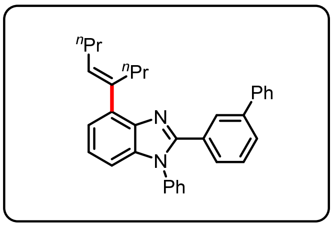

(*E*)-2-([1,1'-Biphenyl]-3-yl)-4-(oct-4-en-4-yl)-1-phenyl-1*H*-benzo[*d*]imidazole (4m)

White solid (160.6 mg, 88% yield), m.p. 94–96 °C. **^{1}H NMR** (400 MHz, $CDCl_3$) δ 7.75 (t, J = 1.6 Hz, 1H), 7.64 – 7.59 (m, 1H), 7.56 – 7.46 (m, 4H), 7.40 – 7.28 (m, 8H), 7.25 – 7.16 (m, 2H), 7.13 – 7.08 (m, 1H), 6.11 (t, J = 7.2 Hz, 1H), 2.96 (t, J = 7.6 Hz, 2H), 2.32 (q, J = 7.2 Hz, 2H), 1.62 – 1.51 (m, 2H), 1.49 – 1.39 (m, 2H), 1.03 (t, J = 7.4 Hz, 3H), 0.93 (t, J = 7.3 Hz, 3H). **^{13}C NMR** (100 MHz, $CDCl_3$) δ 151.3, 141.0, 141.0, 140.6, 139.2, 137.7, 137.6, 136.2, 131.9, 130.9, 130.0, 128.8, 128.8, 128.6, 128.6, 128.5, 127.9, 127.9, 127.5, 127.2, 108.8, 32.5, 30.8, 23.3, 22.2, 14.3, 14.3. **HRMS(ESI)** m/z: $[M+H]^+$ Calcd. for $C_{33}H_{33}N_2$ 457.2638; Found 457.2636.

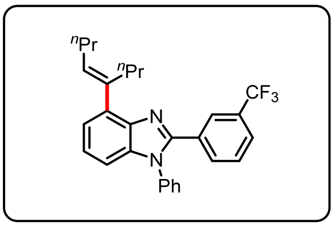

(*E*)-4-(Oct-4-en-4-yl)-1-phenyl-2-(3-(trifluoromethyl)phenyl)-1*H*-benzo[*d*]imidazole (4n)

Colorless oil (73.5 mg, 41% yield). **^{1}H NMR** (400 MHz, $CDCl_3$) δ 7.86 (s, 1H), 7.73 (d, J = 7.6 Hz, 1H), 7.60 – 7.45 (m, 4H), 7.44 – 7.37 (m, 1H), 7.34 – 7.30 (m, 2H), 7.26 – 7.17 (m, 2H), 7.14 – 7.06 (m, 1H), 6.09 (t, J = 7.2 Hz, 1H), 2.93 (t, J = 7.6 Hz, 2H), 2.32 (q, J = 7.2 Hz, 2H), 1.62 – 1.51 (m, 2H), 1.48 – 1.37 (m, 2H), 1.03 (t, J = 7.2 Hz, 3H), 0.93 (t, J = 7.2 Hz, 3H). **^{13}C NMR** (100 MHz, $CDCl_3$) δ 149.8, 140.9, 139.0, 137.7, 137.0, 136.5, 132.7, 132.1, 131.3, 131.0, 130.7, 130.1, 129.0, 128.8, 127.7, 126.54 (q, J = 4.0 Hz), 125.8 (q, J = 4.0 Hz), 123.8, 122.4, 108.9, 32.5, 30.8, 23.3, 22.2, 14.3, 14.2. **^{19}F NMR** (376 MHz, $CDCl_3$) δ – 63.0. **HRMS(ESI)** m/z: $[M+H]^+$ Calcd. for $C_{28}H_{28}F_3N_2$ 449.2199; Found 449.2198.

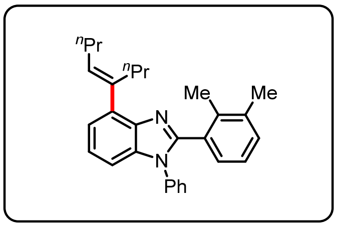

(*E*)-2-(2,3-Dimethylphenyl)-4-(oct-4-en-4-yl)-1-phenyl-1*H*-benzo[*d*]imidazole (4o) White solid (101.3 mg, 62% yield), m.p. 94–96 °C. **^{1}H NMR** (400 MHz, $CDCl_3$) δ 7.40 – 7.28 (m, 3H), 7.23 – 7.16 (m, 5H), 7.15 – 7.06 (m, 2H), 6.99 (t, J = 7.6 Hz, 1H), 5.95 (t, J = 7.2 Hz, 1H), 2.92 (t, J = 7.6 Hz, 2H), 2.27 (q, J = 7.2 Hz, 2H), 2.22 (s, 3H), 2.10 (s, 3H), 1.57 – 1.46 (m, 2H), 1.44 – 1.34 (m, 2H), 0.99 (t, J = 7.2 Hz, 3H), 0.90 (t, J = 7.2 Hz, 3H). **^{13}C NMR** (100 MHz, $CDCl_3$) δ 152.5, 140.9, 139.5, 137.3, 136.8, 136.7, 136.4, 136.0, 131.4, 130.9, 130.6, 129.4, 129.0, 127.8, 126.9, 125.1, 123.0, 122.3, 108.8, 32.7, 30.7, 23.3, 22.1, 20.5, 17.1, 14.3, 14.2. **HRMS(ESI)** m/z: $[M+H]^+$ Calcd. for $C_{29}H_{33}N_2$ 409.2638; Found 409.2639.

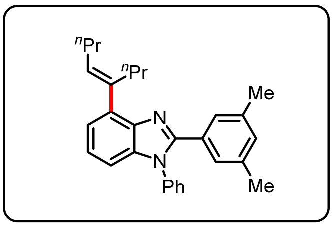

(*E*)-2-(3,5-Dimethylphenyl)-4-(oct-4-en-4-yl)-1-phenyl-1*H*-benzo[*d*]imidazole (4p) Colorless oil (122.5 mg, 75% yield). **^{1}H NMR** (400 MHz, $CDCl_3$) δ 7.50 – 7.39 (m, 3H), 7.32 – 7.26 (m, 2H), 7.21 – 7.13 (m, 4H), 7.11 – 7.06 (m, 1H), 6.94 (s, 1H), 6.05 (t, J = 7.2 Hz, 1H), 2.96 (t, J = 7.6 Hz, 2H), 2.31 (q, J = 7.2 Hz, 2H), 2.20 (s, 6H), 1.60 – 1.49 (m, 2H), 1.47 – 1.36 (m, 2H), 1.02 (t, J = 7.2 Hz, 3H), 0.93 (t, J = 7.2 Hz, 3H). **^{13}C NMR** (100 MHz, $CDCl_3$) δ 151.8, 140.9, 139.4, 137.7, 137.6, 137.5, 136.1, 131.6, 130.9, 130.2, 129.7, 128.4, 127.7, 127.6, 123.0, 122.2, 108.7, 32.5, 30.8, 23.3, 22.2, 21.4, 14.3. HRMS(ESI) m/z: $[M+H]^+$ Calcd. for $C_{29}H_{33}N_2$ 409.2638; Found 409.2636.

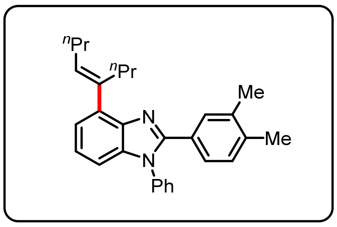

(*E*)-2-(3,4-Dimethylphenyl)-4-(oct-4-en-4-yl)-1-phenyl-1*H*-benzo[*d*]imidazole (4q) Colorless oil (125.8 mg, 77% yield). **^{1}H NMR** (400 MHz, $CDCl_3$) δ 7.51 – 7.40 (m, 4H), 7.30 (d, J = 7.2 Hz, 2H), 7.21 – 7.10 (m, 3H), 7.06 (d, J = 7.6 Hz, 1H), 6.97 (d, J = 7.8 Hz, 1H), 6.07 (t, J = 7.2 Hz, 1H), 2.95 (t, J = 7.6 Hz, 2H), 2.31 (q, J = 7.2 Hz, 2H), 2.22 (s, 3H), 2.19 (s, 3H), 1.61 – 1.50 (m, 2H), 1.48 – 1.36 (m, 2H), 1.03 (t, J = 7.2 Hz, 3H), 0.92 (t, J = 7.2 Hz, 3H). **^{13}C NMR** (100 MHz, $CDCl_3$) δ 151.8, 141.0, 139.4, 138.0, 137.7, 137.7, 136.6, 136.0, 131.7, 131.0, 129.8, 129.4, 128.4, 128.0, 127.8, 127.0, 122.9, 122.1, 108.7, 32.5, 30.8, 23.3, 22.3, 19.8, 19.8, 14.3, 14.3. **HRMS(ESI)** m/z: $[M+H]^+$ Calcd. for $C_{29}H_{33}N_2$ 409.2638; Found 409.2637.

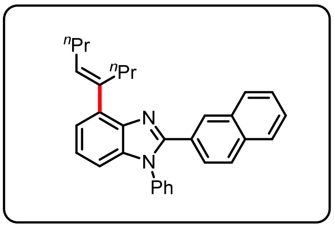

(*E*)-2-(Naphthalen-2-yl)-4-(oct-4-en-4-yl)-1-phenyl-1*H*-benzo[*d*]imidazole (4r)

White solid (111.9 mg, 65% yield), m.p. 134–136 °C. **^{1}H NMR** (400 MHz, $CDCl_3$) δ 8.04 (s, 1H), 7.77 (d, J = 7.6 Hz, 1H), 7.74 – 7.66 (m, 3H), 7.52 – 7.40 (m, 5H), 7.38 – 7.31 (m, 2H), 7.26 – 7.16 (m, 2H), 7.14 – 7.10 (m, 1H), 6.10 (t, J = 7.2 Hz, 1H), 2.98 (t, J = 7.6 Hz, 2H), 2.33 (q, J = 7.2 Hz, 2H), 1.62 – 1.51 (m, 2H), 1.50 – 1.39 (m, 2H), 1.04 (t, J = 7.2 Hz, 3H), 0.95 (t, J = 7.2 Hz, 3H). **^{13}C NMR** (100 MHz, $CDCl_3$) δ 151.4, 141.1, 140.4, 139.3, 137.8, 137.6, 136.3, 133.5, 133.0, 131.9, 130.0, 129.7, 128.8, 128.6, 127.9, 127.8, 127.8, 127.8, 127.0, 126.8, 126.4, 123.3, 122.3, 108.8, 32.6, 30.8, 23.3, 22.3, 14.3, 14.3. **HRMS(ESI)** m/z: $[M+H]^+$ Calcd. for $C_{31}H_{31}N_2$ 431.2482; Found 431.2481.

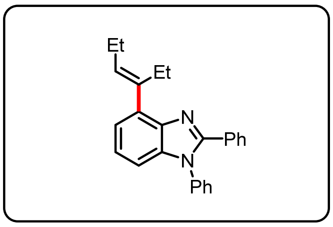

(*E*)-4-(Hex-3-en-3-yl)-1,2-diphenyl-1*H*-benzo[*d*]imidazole (5a)

White solid (122.6 mg, 87% yield), m.p. 74–76 °C. **^{1}H NMR** (400 MHz, $CDCl_3$) δ 7.59 – 7.55 (m, 2H), 7.52 – 7.42 (m, 3H), 7.34 – 7.24 (m, 5H), 7.23 – 7.15 (m, 2H), 7.12 – 7.07 (m, 1H), 6.05 (t, J = 7.2 Hz, 1H), 2.96 (q, J = 7.6 Hz, 2H), 2.39 – 2.30 (m, 2H), 1.13 (t, J = 7.6 Hz, 3H), 1.03 (t, J = 7.6 Hz, 3H). **^{13}C NMR** (100 MHz, $CDCl_3$) δ 151.6, 141.0, 140.4, 137.7, 137.5, 135.8, 132.8, 130.5, 129.9, 129.8, 129.3, 128.5, 128.3, 127.7, 123.2, 122.3, 108.8, 23.7, 21.8, 14.7, 14.0. **HRMS(ESI)** m/z: $[M+H]^+$ Calcd. for $C_{25}H_{25}N_2$ 353.2012; Found 353.2008.

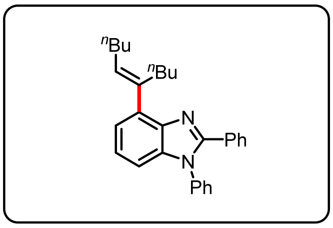

(*E*)-4-(Dec-5-en-5-yl)-1,2-diphenyl-1*H*-benzo[*d*]imidazole (5b)

White solid (140.5 mg, 86% yield), m.p. 84–86 °C. **^{1}H NMR** (400 MHz, $CDCl_3$) δ 7.59 – 7.53 (m, 2H), 7.50 – 7.40 (m, 3H), 7.33 – 7.22 (m, 5H), 7.22 – 7.14 (m, 2H), 7.11 – 7.06 (m, 1H), 6.06 (t, J = 7.2 Hz, 1H), 2.97 (t, J = 7.2 Hz, 2H), 2.33 (q, J = 7.2 Hz, 2H), 1.57 – 1.31 (m, 8H), 0.96 (t, J = 7.2 Hz, 3H), 0.88 (t, J = 6.8 Hz, 3H). **^{13}C NMR** (100 MHz, $CDCl_3$) δ 151.5, 141.0, 139.3, 137.6, 137.5, 136.2, 131.8, 130.5, 129.9, 129.7, 129.2, 128.5, 128.3, 127.7, 123.2, 122.2, 108.7, 32.4, 31.4, 30.2, 28.4, 23.0, 22.7, 14.3, 14.2. **HRMS(ESI)** m/z: $[M+H]^+$ Calcd. for $C_{29}H_{33}N_2$ 409.2638; Found 409.2635.

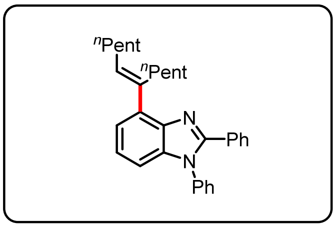

(*E*)-4-(Dodec-6-en-6-yl)-1,2-diphenyl-1*H*-benzo[*d*]imidazole (5c)
Colorless oil (148.4 mg, 85% yield). **^{1}H NMR** (400 MHz, $CDCl_3$) δ 7.59 – 7.53 (m, 2H), 7.51 – 7.42 (m, 3H), 7.34 – 7.24 (m, 5H), 7.22 – 7.14 (m, 2H), 7.11 – 7.07 (m, 1H), 6.08 (t, *J* = 7.2 Hz, 1H), 2.95 (t, *J* = 7.2 Hz, 2H), 2.32 (q, *J* = 7.2 Hz, 2H), 1.57 – 1.47 (m, 2H), 1.44 – 1.25 (m, 10H), 0.93 (t, *J* = 7.2 Hz, 3H), 0.85 (t, *J* = 7.2 Hz, 3H). **^{13}C NMR** (100 MHz, $CDCl_3$) δ 151.5, 141.0, 139.3, 137.7, 137.5, 136.2, 131.9, 130.5, 129.9, 129.7, 129.2, 128.5, 128.3, 127.7, 123.2, 122.2, 108.7, 32.1, 32.0, 30.5, 29.9, 28.8, 28.7, 22.9, 22.8, 14.3, 14.3. **HRMS(ESI)** m/z: $[M+H]^+$ Calcd. for $C_{31}H_{37}N_2$ 437.2951; Found 437.2949.

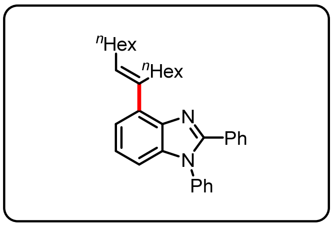

(*E*)-1,2-Diphenyl-4-(tetradec-7-en-7-yl)-1*H*-benzo[*d*]imidazole (5d)
Colorless oil (163.5 mg, 88% yield). **^{1}H NMR** (400 MHz, $CDCl_3$) δ 7.57 (s, 1H), 7.56 (s, 1H), 7.51 – 7.42 (m, 3H), 7.34 – 7.23 (m, 5H), 7.22 – 7.14 (m, 2H), 7.11 – 7.06 (m, 1H), 6.07 (t, *J* = 7.2 Hz, 1H), 2.95 (t, *J* = 7.2 Hz, 2H), 2.32 (q, *J* = 7.2 Hz, 2H), 1.54 – 1.22 (m, 16H), 0.91 (t, *J* = 6.8 Hz, 3H), 0.85 (t, *J* = 6.8 Hz, 3H). **^{13}C NMR** (100 MHz, $CDCl_3$) δ 151.5, 141.0, 139.4, 137.7, 137.5, 136.2, 131.8, 130.6, 129.9, 129.8, 129.2, 128.5, 128.3, 127.7, 123.2, 122.2, 108.8, 32.1, 32.0, 30.5, 30.1, 29.6, 29.4, 29.1, 28.7, 22.9, 22.8, 14.3, 14.3. **HRMS(ESI)** m/z: $[M+H]^+$ Calcd. for $C_{33}H_{41}N_2$ 465.3264; Found 465.3262.

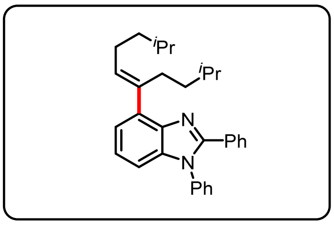

(*E*)-4-(2,9-Dimethyldec-5-en-5-yl)-1,2-diphenyl-1*H*-benzo[*d*]imidazole (5e)
Colorless oil (155.3 mg, 89% yield). **^{1}H NMR** (400 MHz, $CDCl_3$) δ 7.59 – 7.54 (m, 2H), 7.51 – 7.41 (m, 3H), 7.33 – 7.23 (m, 5H), 7.21 – 7.15 (m, 2H), 7.10 – 7.07 (m, 1H), 6.06 (t, *J* = 7.2 Hz, 1H), 3.02 – 2.93 (m, 2H), 2.32 (q, *J* = 7.2 Hz, 2H), 1.76 – 1.57 (m, 2H), 1.45 – 1.37 (m, 2H), 1.35 – 1.25 (m, 2H), 0.96 (d, *J* = 6.8 Hz, 6H), 0.90 (d, *J* = 6.8 Hz, 6H). **^{13}C NMR** (100 MHz, $CDCl_3$) δ 151.5, 140.9, 139.4, 137.6, 137.5, 136.1, 131.7, 130.5, 129.9, 129.7, 129.2, 128.5, 128.3, 127.7, 123.2, 122.1, 108.7, 39.4, 38.4, 28.5, 28.5, 28.0, 26.5, 22.8, 22.8. **HRMS(ESI)** m/z: $[M+H]^+$ Calcd. for $C_{31}H_{37}N_2$ 437.2949; Found 437.2951.

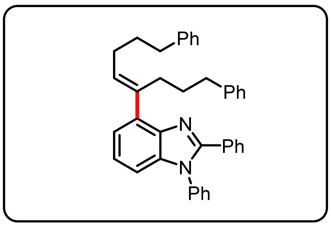

(*E*)-4-(1,8-Diphenyloct-4-en-4-yl)-1,2-diphenyl-1*H*-benzo[*d*]imidazole (5f)
Colorless oil (189.5 mg, 89% yield). **^{1}H NMR** (400 MHz, $CDCl_3$) δ 7.56 – 7.52 (m, 2H), 7.50 – 7.41 (m, 3H), 7.32 – 7.16 (m, 14H), 7.14 – 7.08 (m, 4H), 6.11 (t, J = 7.2 Hz, 1H), 3.00 (t, J = 8.0 Hz, 2H), 2.78 – 2.70 (m, 2H), 2.67 – 2.60 (m, 2H), 2.33 (q, J = 7.2 Hz, 2H), 1.89 – 1.80 (m, 2H), 1.78 – 1.69 (m, 2H). **^{13}C NMR** (100 MHz, $CDCl_3$) δ 151.6, 143.0, 142.8, 141.0, 139.4, 137.6, 137.4, 135.8, 131.5, 130.5, 129.9, 129.7, 129.3, 128.7, 128.6, 128.5, 128.4, 128.3, 128.2, 127.7, 125.8, 125.6, 123.2, 122.1, 108.9, 36.0, 35.8, 31.8, 30.7, 30.2, 28.2. **HRMS(ESI)** m/z: $[M+H]^+$ Calcd. for $C_{39}H_{37}N_2$ 533.2951; Found 533.2952.

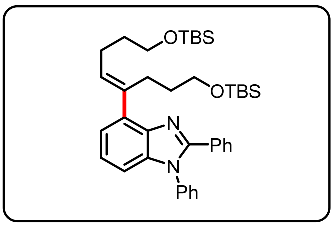

(*E*)-4-(2,2,3,3,14,14,15,15-octamethyl-4,13-dioxa-3,14-disilahexadec-8-en-8-yl)-1,2-diphenyl-1*H*-benzo[*d*]imidazole (5g)
Colorless oil (215.2 mg, 84% yield). **^{1}H NMR** (400 MHz, $CDCl_3$) δ 7.58 – 7.54 (m, 2H), 7.52 – 7.42 (m, 3H), 7.34 – 7.23 (m, 5H), 7.21 – 7.14 (m, 2H), 7.11 – 7.06 (m, 1H), 6.11 (t, J = 7.2 Hz, 1H), 3.73 (t, J = 6.4 Hz, 2H), 3.63 (t, J = 6.8 Hz, 2H), 3.06 – 2.95 (m, 2H), 2.38 (q, J = 7.2 Hz, 2H), 1.81 – 1.71 (m, 2H), 1.68 – 1.57 (m, 2H), 0.92 (s, 9H), 0.86 (s, 9H), 0.08 (s, 6H), -0.00 (s, 6H). **^{13}C NMR** (100 MHz, $CDCl_3$) δ 151.5, 141.0, 139.2, 137.7, 137.5, 135.7, 131.3, 130.5, 129.9, 129.7, 129.2, 128.5, 128.3, 127.7, 123.2, 122.2, 108.9, 63.4, 63.1, 33.3, 32.3, 26.7, 26.2, 26.1, 25.0, 18.5, 18.5, -5.1, -5.1. **HRMS(ESI)** m/z: $[M+H]^+$ Calcd. for $C_{39}H_{57}N_2O_2Si_2$ 641.3953; Found 641.3950.

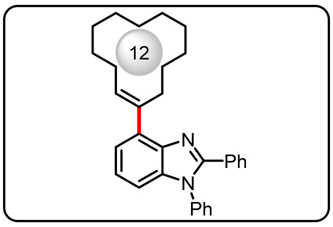

(*E*)-4-(Cyclododec-1-en-1-yl)-1,2-diphenyl-1*H*-benzo[*d*]imidazole (5h)
Colorless oil (88.6 mg, 51% yield). **^{1}H NMR** (400 MHz, $CDCl_3$) δ 7.60 – 7.54 (m, 2H), 7.52 – 7.42 (m, 3H), 7.34 – 7.24 (m, 5H), 7.22 – 7.16 (m, 2H), 7.13 – 7.08 (m, 1H), 5.81 (t, J = 8.0 Hz, 1H), 3.12 (t, J = 6.4 Hz, 2H), 2.37 (q, J = 7.2 Hz, 2H), 1.64 – 1.58 (m, 2H), 1.50 – 1.34 (m, 12H), 1.30 – 1.23 (m, 2H). **^{13}C NMR** (100 MHz, $CDCl_3$) δ 151.5, 141.0, 140.0, 137.5, 137.4, 136.5, 132.2, 130.5, 129.9, 129.7, 129.2, 128.5, 128.3, 127.7, 123.2, 122.8, 108.8, 27.6, 26.5, 26.0, 25.4, 25.1, 24.7, 24.6, 22.7, 22.6. **HRMS(ESI)**

m/z: $[M+H]^+$ Calcd. for $C_{31}H_{35}N_2$ 435.2795; Found 435.2790.

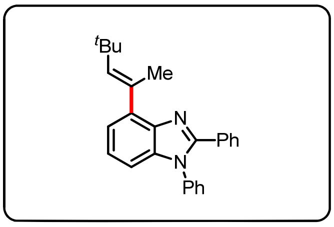

(*E*)-4-(4,4-Dimethylpent-2-en-2-yl)-1,2-diphenyl-1*H*-benzo[*d*]imidazole (5i)
Colorless oil (77.6 mg, 53% yield). **^{1}H NMR** (400 MHz, $CDCl_3$) δ 7.60 – 7.55 (m, 2H), 7.52 – 7.43 (m, 3H), 7.34 – 7.27 (m, 5H), 7.21 – 7.15 (m, 2H), 7.11 – 7.06 (m, 1H), 6.11 (d, J = 1.2 Hz, 1H), 2.51 (d, J = 1.2 Hz, 3H), 1.31 (s, 9H). **^{13}C NMR** (100 MHz, $CDCl_3$) δ 151.5, 141.7, 140.7, 138.7, 137.8, 137.5, 133.7, 130.5, 129.9, 129.7, 129.3, 128.6, 128.3, 127.7, 123.3, 121.6, 108.8, 33.2, 31.3, 18.5. **HRMS(ESI)** m/z: $[M+H]^+$ Calcd. for $C_{26}H_{27}N_2$ 367.2169; Found 367.2172.

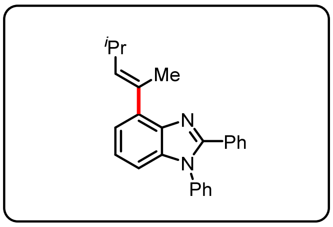

(*E*)-4-(4-Methylpent-2-en-2-yl)-1,2-diphenyl-1*H*-benzo[*d*]imidazole (5j)
Colorless oil (124.0 mg, 88% yield). **^{1}H NMR** (400 MHz, $CDCl_3$) δ 7.61 – 7.56 (m, 2H), 7.52 – 7.42 (m, 3H), 7.33 – 7.17 (m, 7H), 7.10 – 7.05 (m, 1H), 6.11 – 6.05 (m, 1H), 2.92 – 2.78 (m, 1H), 2.41 (s, 3H), 1.13 (d, J = 6.8 Hz, 6H). **^{13}C NMR** (100 MHz, $CDCl_3$) δ 151.5, 140.8, 139.3, 137.9, 137.5, 136.8, 132.0, 130.5, 129.9, 129.7, 129.3, 128.6, 128.3, 127.7, 123.3, 121.4, 108.8, 28.0, 23.2, 17.1. **HRMS(ESI)** m/z: $[M+H]^+$ Calcd. for $C_{25}H_{25}N_2$ 353.2012; Found 353.2007.

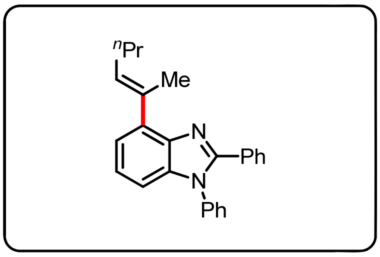

(*E*)-4-(hex-2-en-2-yl)-1,2-diphenyl-1*H*-benzo[*d*]imidazole (5k)
Colorless oil (76.1 mg, 54% yield). **^{1}H NMR** (400 MHz, $CDCl_3$) δ 7.56 (d, J = 7.0 Hz, 2H), 7.51 – 7.44 (m, 3H), 7.35 – 7.26 (m, 5H), 7.21 – 7.16 (m, 2H), 7.13 – 7.07 (m, 1H), 6.20 (q, J = 6.8 Hz, 1H), 2.93 (t, J = 7.6 Hz, 2H), 1.91 (d, J = 6.8 Hz, 3H), 1.49 – 1.38 (m, 2H), 0.94 (t, J = 7.2 Hz, 3H). **^{13}C NMR** (100 MHz, $CDCl_3$) δ 151.6, 142.6, 140.9, 140.0, 137.6, 137.5, 136.1, 129.9, 129.8, 129.3, 128.5, 128.3, 127.7, 125.8, 123.2, 122.1, 108.8, 32.2, 24.4, 22.0, 14.2. **HRMS(ESI)** m/z: $[M+H]^+$ Calcd. for $C_{25}H_{25}N_2$ 353.2012; Found 353.2014.

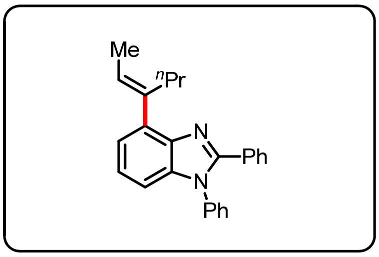

(*E*)-4-(Hex-2-en-3-yl)-1,2-diphenyl-1*H*-benzo[*d*]imidazole (5k′)
Colorless oil (39.5 mg, 28% yield). ^{1}**H NMR** (400 MHz, $CDCl_3$) δ 7.60 – 7.55 (m, 2H), 7.52 – 7.42 (m, 3H), 7.34 – 7.26 (m, 5H), 7.24 – 7.15 (m, 2H), 7.08 (d, *J* = 7.6 Hz, 1H), 6.35 (t, *J* = 6.8 Hz, 1H), 2.39 – 2.27 (m, 5H), 1.62 – 1.50 (m, 2H), 1.03 (t, *J* = 7.2 Hz, 3H). ^{13}**C NMR** (100 MHz, $CDCl_3$) δ 151.6, 140.8, 137.9, 137.5, 136.8, 133.7, 132.2, 130.5, 129.9, 129.7, 129.3, 128.6, 128.3, 127.7, 123.3, 121.2, 108.8, 31.1, 23.0, 17.1, 14.2. **HRMS(ESI)** m/z: $[M+H]^+$ Calcd. for $C_{25}H_{25}N_2$ 353.2012; Found 353.2015

Supplementary Note 5

Reaction of Imine, Pyridine and Other Heterocycles

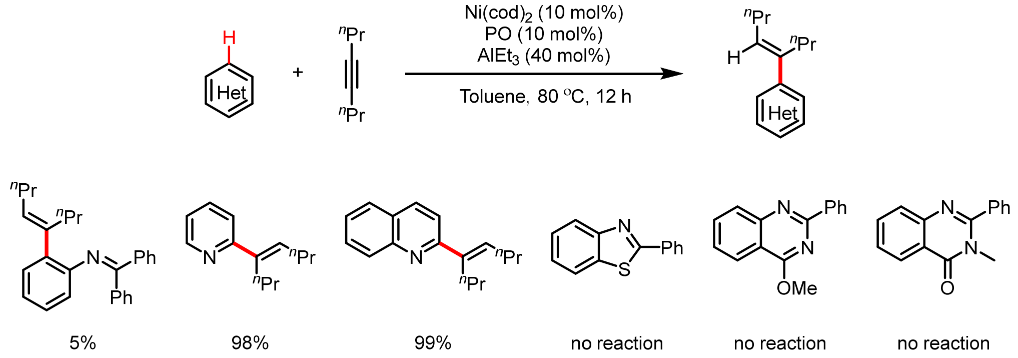

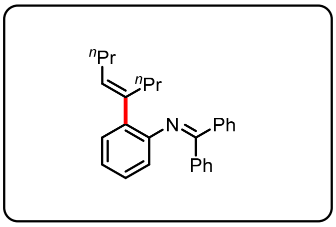

(*E*)-N-(diphenylmethylene)-2-(oct-4-en-4-yl)aniline
Colorless oil (7.4 mg, 5% yield).^{1}**H NMR** (400 MHz, $CDCl_3$) δ 7.74 – 7.69 (m, 2H), 7.44 – 7.39 (m, 1H), 7.38 – 7.32 (m, 2H), 7.26 – 7.22 (m, 2H), 7.20 – 7.14 (m, 2H), 7.13 – 7.06 (m, 4H), 6.91 (t, *J* = 7.4 Hz, 1H), 6.71 (d, *J* = 7.4 Hz, 2H), 2.10 – 1.97 (m, 1H), 1.91 – 1.76 (m, 2H), 1.23 – 1.16 (m, 2H), 1.15 – 1.06 (m, 2H), 0.81 (t, *J* = 7.4 Hz, 3H), 0.69 (t, *J* = 7.4 Hz, 3H). ^{13}**C NMR** (151 MHz, $CDCl_3$) δ 168.3, 150.7, 144.2, 140.4, 139.7, 134.4, 132.1, 130.5, 130.1, 129.1, 128.7, 128.4, 125.8, 123.6, 121.7, 32.9, 30.7, 22.7, 22.3, 14.2, 14.1. **HRMS(ESI)** m/z: $[M+H]^+$ Calcd. for $C_{27}H_{30}N$ 368.2373; Found 368.2375.

Supplementary Note 6

Gram-Scale Reaction

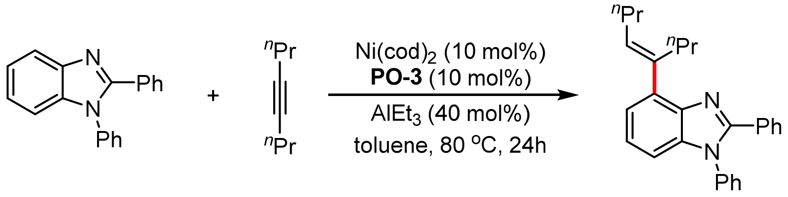

1a **2a** **3a**

In an argon-filled glove-box, to an oven-dried sealed tube were added Ni(cod)$_2$ (110 mg, 0.4 mmol), **1a** (4 mmol), **PO-3** (160 mg, 0.4 mmol), toluene (8 mL), **2a** (8 mmol), and $AlEt_3$ (1 mol/L in hexane, 1.60 mL, 1.6 mmol) in sequence. The tube was then sealed, removed out of the glove-box, and heated at 80°C with heating mantle as the heat source for 24 h. The mixture was then cooled to room temperature and concentrated in vacuo. The crude product was purified by flash column chromatography using ethyl acetate/hexane as the eluent to give the product 3a (1.23g) in 81% yield.

Supplementary Note 7

Product Transformation

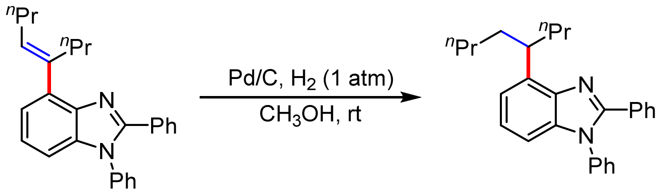

3a **6**

The olefinic compound **3a** (380.2 mg, 1.0 mmol) and Pd/C (53.0 mg, containing 10% Pd) in CH_3OH (10 mL) was stirred under 1 atm pressure of hydrogen at room temperature for 24 hours. The mixture was filtered and concentrated under vacuum to provide the crude product, which was further purification by flash column chromatography using ethyl acetate/hexane as the eluent to provide **6**.

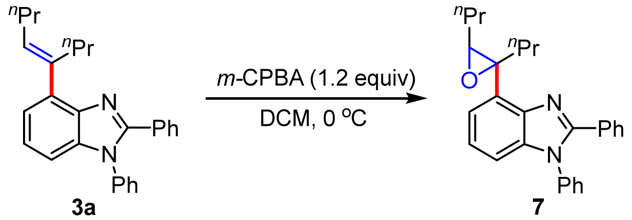

3a **7**

To a stirred solution of **3a** (380.2 mg, 1.0 mol) in anhydrous CH_2Cl_2 (1 mL) was added *m*-CPBA (207.1 mg, 1.2 mol). The solution was stirred at 0 °C for 2 h and filtered to remove the solids. The filtrate was diluted with water, and extracted with DCM. The combined organic layer was then washed with $NaHCO_3$ solution, dried

over anhydrous Na_2SO_4, filtered and concentrated in vacuo. The crude residue was purified by flash silica gel column chromatography to afford **7**.

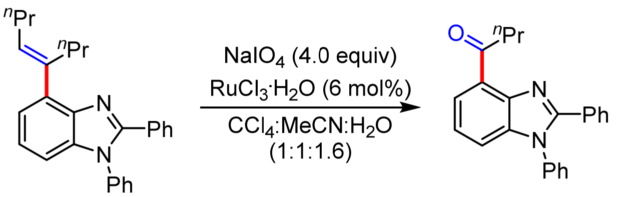

To a solution of **3a** (380.2 mg, 1.0 mmol) in a mixed solvent $CCl_4/CH_3CN/H_2O$ (1:1:1.6, v/v/v, 3.6 mL) was added $NaIO_4$ (863.7 mg, 4.0 mmol). The mixture was stirred for 5 min before the addition of $RuCl_3·3H_2O$ (153.4 mg, 0.6 mmol). The resulting brown mixture was stirred for 24 h, quenched with 1N HCl, and extracted with CH_2Cl_2. The combined organic phase was dried over anhydrous Na_2SO_4 and concentrated in vaccuo.The residue was purified by silica column chromatography with petroleum ether/ethyl acetate as the eluent to give product **8**.

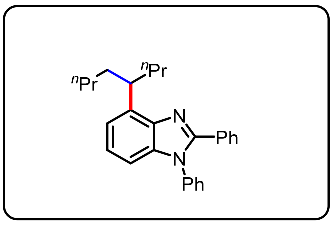

4-(Octan-4-yl)-1,2-diphenyl-1*H*-benzo[*d*]imidazole (6)

Colorless oil (382.3 mg, 100% yield). **^{1}H NMR** (400 MHz, $CDCl_3$) δ 7.57 – 7.52 (m, 2H), 7.48 – 7.37 (m, 3H), 7.33 – 7.23 (m, 5H), 7.23 – 7.16 (m, 1H), 7.14 – 7.09 (m, 1H), 7.06 – 7.01 (m, 1H), 3.68 – 3.57 (m, 1H), 1.95 – 1.75 (m, 4H), 1.41 – 1.17 (m, 6H), 0.93 – 0.81 (m, 6H). **^{13}C NMR** (100 MHz, $CDCl_3$) δ 151.4, 142.6, 138.9, 137.5, 137.1, 130.7, 129.8, 129.2, 128.3, 127.6, 123.2, 120.3, 107.6, 40.1, 38.6, 35.9, 30.0, 23.1, 21.0, 14.6, 14.2. **HRMS(ESI)** m/z: $[M+H]^+$ Calcd. for $C_{27}H_{31}N_2$ 383.2482; Found 394.2479.

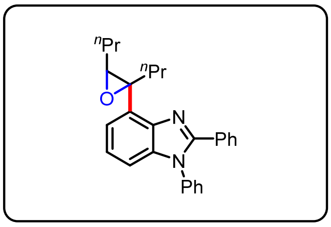

4-(2,3-Dipropyloxiran-2-yl)-1,2-diphenyl-1*H*-benzo[*d*]imidazole (7)

Colorless oil (237.8 mg, 60% yield). **^{1}H NMR** (400 MHz, $CDCl_3$) δ 7.61 – 7.56 (m, 2H), 7.54 – 7.45 (m, 3H), 7.35 – 7.27 (m, 6H), 7.19 (t, J = 7.6 Hz, 1H), 7.13 (dd, J = 8.0, 1.2 Hz, 1H), 3.03 (t, J = 6.0 Hz, 1H), 2.86 – 2.77 (m, 1H), 1.96 – 1.76 (m, 3H), 1.73 – 1.62 (m, 2H), 1.52 – 1.28 (m, 2H), 1.10 (t, J = 7.6 Hz, 3H), 0.92 (t, J = 7.6 Hz, 3H). **^{13}C NMR** (100 MHz, $CDCl_3$) δ 151.7, 140.9, 137.4, 137.4, 133.7, 130.4, 130.0, 129.7, 129.4, 128.6, 128.3, 127.6, 123.0, 120.1, 109.6, 65.9, 64.4, 33.8, 30.8, 20.1, 19.0, 14.6, 14.3. **HRMS(ESI)** m/z: $[M+H]^+$ Calcd. for $C_{27}H_{29}N_2O$ 397.2274; Found 397.2270.

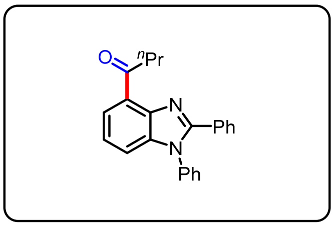

1-(1,2-Diphenyl-1*H*-benzo[*d*]imidazol-4-yl)butan-1-one (8)

Colorless oil (248.3 mg, 73% yield). **^{1}H NMR** (400 MHz, $CDCl_3$) δ 7.98 (dd, J = 7.4, 1.2 Hz, 1H), 7.67 – 7.62 (m, 2H), 7.57 – 7.52 (m, 3H), 7.44 – 7.39 (m, 2H), 7.39 – 7.32 (m, 5H), 3.63 (t, J = 7.2 Hz, 2H), 1.96 – 1.84 (m, 2H), 1.10 (t, J = 7.2 Hz, 3H). **^{13}C NMR** (100 MHz, $CDCl_3$) δ 202.0, 153.0, 142.0, 138.8, 136.9, 130.2, 129.9, 129.8, 129.7, 129.1, 129.1, 128.5, 127.7, 124.0, 122.9, 114.8, 45.7, 18.2, 14.3. **HRMS(ESI)** m/z: $[M+H]^+$ Calcd. for $C_{23}H_{21}N_2O$ 341.1648; Found 341.1645.

Supplementary Note 8

Late-Stage C–H Alkenylation of Bioactive Molecules

In an argon-filled glove-box, to an oven-dried sealed tube were added Ni(cod)$_2$ (5.5 mg, 0.04 mmol), **PO-3** (16.0 mg, 0.04 mmol), toluene (0.8 mL), bioactive molecule (0.40 mmol), $AlEt_3$ (1 mol/L in hexane, 160 µL, 0.16 mmol) and oct-4-yne (**2a**, 0.80 mmol) in sequence. The tube was then sealed, removed out of the glove-box and heated at 80 °C with heating mantle as the heat source for 12 h. Then the mixture was cooled to room temperature and concentrated in vacuo. The crude product was purified by flash column chromatography using ethyl acetate/hexane as eluent.

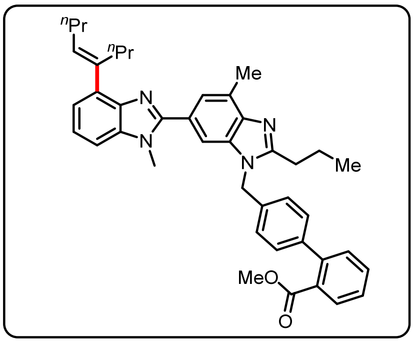

(*E*)-Methyl-4'-((1,7'-dimethyl-4-(oct-4-en-4-yl)-2'-propyl-1*H*,3'*H*-[2,5'-bibenzo[*d*] imidazol]-3'-yl)methyl)-[1,1'-biphenyl]-2-carboxylate (9)

Colorless oil (191.6 mg, 75% yield). **^{1}H NMR** (400 MHz, $CDCl_3$) δ 7.81 (dd, J = 7.7, 1.2 Hz, 1H), 7.53 – 7.47 (m, 1H), 7.45 – 7.43 (m, 1H), 7.43 – 7.40 (m, 1H), 7.38 (dd, J = 7.6, 1.3 Hz, 1H), 7.29 (dd, J = 7.6, 0.8 Hz, 1H), 7.27 – 7.21 (m, 4H), 7.15 (q, J = 4.4 Hz, 1H), 7.10 (d, J = 8.2 Hz, 2H), 5.94 (t, J = 7.2 Hz, 1H), 5.43 (s, 2H), 3.74 (s, 3H), 3.56 (s, 3H), 2.97 – 2.91 (m, 2H), 2.90 – 2.84 (m, 2H), 2.76 (s, 3H), 2.25 (q, J = 7.6 Hz, 2H), 1.93 – 1.81 (m, 2H), 1.54 – 1.44 (m, 2H), 1.39 – 1.27 (m, 2H), 1.05 (t, J = 7.4 Hz, 3H), 0.97 (t, J = 7.4 Hz, 3H), 0.86 (t, J = 7.4 Hz, 3H). **^{13}C NMR** (100 MHz, $CDCl_3$) δ 168.8, 156.3, 153.9, 143.1, 141.9, 141.2, 140.8, 139.4, 136.8, 136.0, 135.1, 134.9,

131.5, 131.3, 131.3, 130.8, 130.6, 130.0, 129.4, 129.1, 127.5, 126.1, 124.4, 124.2, 122.4, 121.8, 109.3, 107.8, 52.0, 52.0, 47.3, 32.5, 31.8, 30.7, 30.0, 23.2, 22.1, 22.0, 17.1, 17.0, 14.2, 14.2. **HRMS(ESI)** m/z: $[M+H]^+$ Calcd. for $C_{42}H_{47}N_4O_2$ 639.3694; Found 639.3690.

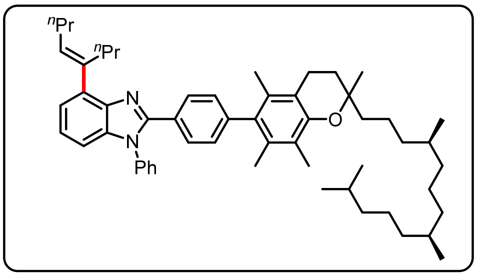

4-((*E*)-Oct-4-en-4-yl)-1-phenyl-2-(4-(2,5,7,8-tetramethyl-2-((*4S,8S*)-4,8,12-trimethyltridecyl)chroman-6-yl)phenyl)-1*H*-benzo[*d*]imidazole (10)

Colorless oil (164.9 mg, 52% yield). ^{1}**H NMR** (400 MHz, $CDCl_3$) δ 7.61 – 7.56 (d, *J* = 8.4 Hz, 2H), 7.53 – 7.42 (m, 3H), 7.38 – 7.32 (m, 2H), 7.23 – 7.15 (m, 2H), 7.13 – 7.09 (m, 1H), 7.06 – 7.01 (m, 2H), 6.06 (t, *J* = 7.2 Hz, 1H), 3.04 – 2.92 (t, *J* = 7.6 Hz, 2H), 2.61 (t, *J* = 6.6 Hz, 2H), 2.32 (q, *J* = 7.2 Hz, 2H), 2.13 (s, 3H), 1.92 – 1.76 (m, 9H), 1.65 – 1.37 (m, 14H), 1.33 – 1.21 (m, 14H), 1.05 – 1.01 (m, 3H), 0.94 (t, *J* = 7.2 Hz, 3H), 0.86 (t, *J* = 6.4 Hz, 11H). ^{13}**C NMR** (100 MHz, $CDCl_3$) δ 151.8, 151.0, 143.9, 141.0, 139.4, 137.6, 136.2, 133.0, 132.9, 131.7, 131.6, 130.2, 129.8, 129.6, 128.5, 128.3, 127.7, 123.1, 122.3, 122.0, 116.8, 108.8, 75.2, 40.5, 40.4, 39.5, 37.7, 37.6, 37.6, 37.6, 37.5, 37.5, 37.4, 32.9, 32.9, 32.9, 32.8, 32.6, 31.4, 31.4, 30.8, 28.1, 25.0, 24.6, 24.1, 23.3, 22.9, 22.8, 22.2, 21.2, 20.9, 19.9, 19.8, 19.8, 19.8, 17.8, 16.8, 14.3, 14.3, 12.0. **HRMS(ESI)** m/z: $[M+H]^+$ Calcd. for $C_{56}H_{77}N_2O$ 793.6030; Found 793.6026.

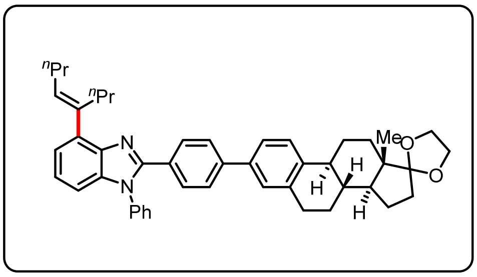

2-(4-((*8R,9S,13S,14S*)-13-Methyl-6,7,8,9,11,12,13,14,15,16-decahydrospiro[cyclopenta[*a*]phenanthrene-17,2'-[1,3]dioxolan]-3-yl)phenyl)-4-((*E*)-oct-4-en-4-yl)-1-phenyl-1*H*-benzo[*d*]imidazole (11)

Colorless oil (167.8 mg, 62% yield). ^{1}**H NMR** (400 MHz, $CDCl_3$) δ 7.63 (s, 1H), 7.61 (s, 1H), 7.55 – 7.44 (m, 5H), 7.38 – 7.34 (m, 4H), 7.30 (s, 1H), 7.23 – 7.15 (m, 2H), 7.09 (dd, *J* = 7.4, 1.6 Hz, 1H), 6.08 (t, *J* = 7.2 Hz, 1H), 4.03 – 3.86 (m, 4H), 3.02 – 2.86 (m, 4H), 2.45 – 2.26 (m, 4H), 2.10 – 2.00 (m, 1H), 1.98 – 1.75 (m, 5H), 1.60 – 1.35 (m, 9H), 1.03 (t, *J* = 7.2 Hz, 3H), 0.93 (t, *J* = 7.2 Hz, 3H), 0.89 (s, 3H). ^{13}**C NMR** (100 MHz, $CDCl_3$) δ 151.3, 141.8, 141.0, 140.2, 139.3, 137.7, 137.7, 137.5, 137.4, 136.1, 131.8, 130.0, 130.0, 129.0, 128.6, 127.8, 127.7, 126.8, 126.1, 124.4, 123.2, 122.2, 119.5, 108.7, 65.4, 64.7, 49.6, 46.3, 44.2, 39.0, 34.4, 32.5, 30.9, 30.8, 29.8, 27.1, 26.1, 23.3, 22.5, 22.2, 14.5, 14.3, 14.3. **HRMS(ESI)** m/z: $[M+H]^+$ Calcd. for $C_{47}H_{53}N_2O_2$ 677.4102; Found 677.4097.

Supplementary Note 9

Mechanistic Experiments

Preparation of Deuterated Substrates

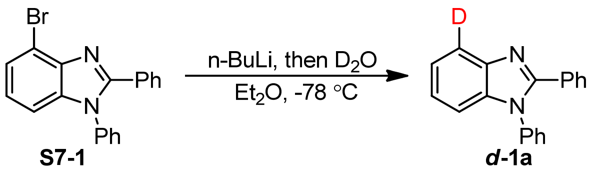

To a 250 mL round bottom flask with a stir bar were added **S7-1** (3.48 g, 10.0 mmol) and Et_2O (100 mL) under N_2 atmosphere. Then ^{n}BuLi (6 mL, 2.5 M) was added dropwise at –78 °C and the reaction mixture was stirred at –78 °C for 2 h. Then D_2O (600 µL, 3.0 equiv.) was added, and the solution was stirred for half an hour at –78 °C, and another 1h at room temperature. The reaction was quenched with NH_4Cl (aq.), and the organic layer was dried over anhydrous Na_2SO_4, filtered and concentrated in vacuo. The residue was purified by column chromatography on silica gel (petroleum ether/ ethyl acetate, v/v, 8:1) (100%, 2.74 g, 96% D). **^{1}H NMR** (400 MHz, $CDCl_3$) δ 7.59 – 7.55 (m, 2H), 7.54 – 7.44 (m, 3H), 7.38 – 7.29 (m, 6H), 7.29 – 7.26 (m, 2H).

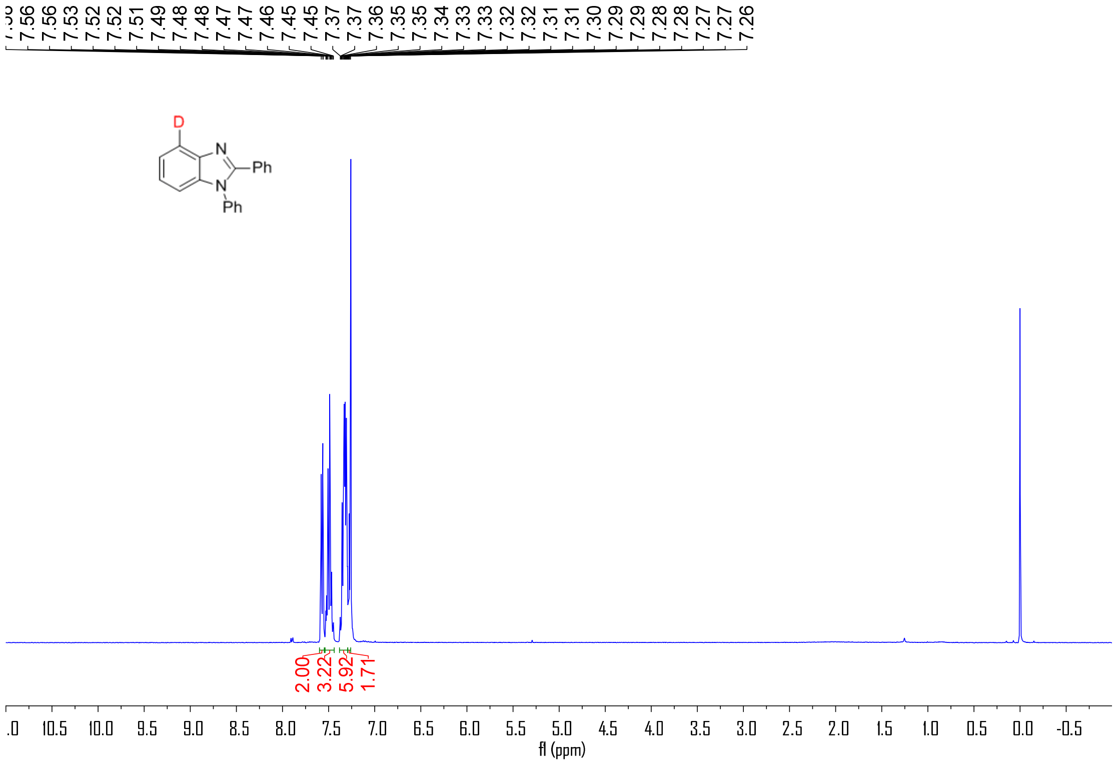

Supplementary Figure 1. ^{1}H NMR spectrum of deuterated substrates

Parallel Reactions for KIE Determination

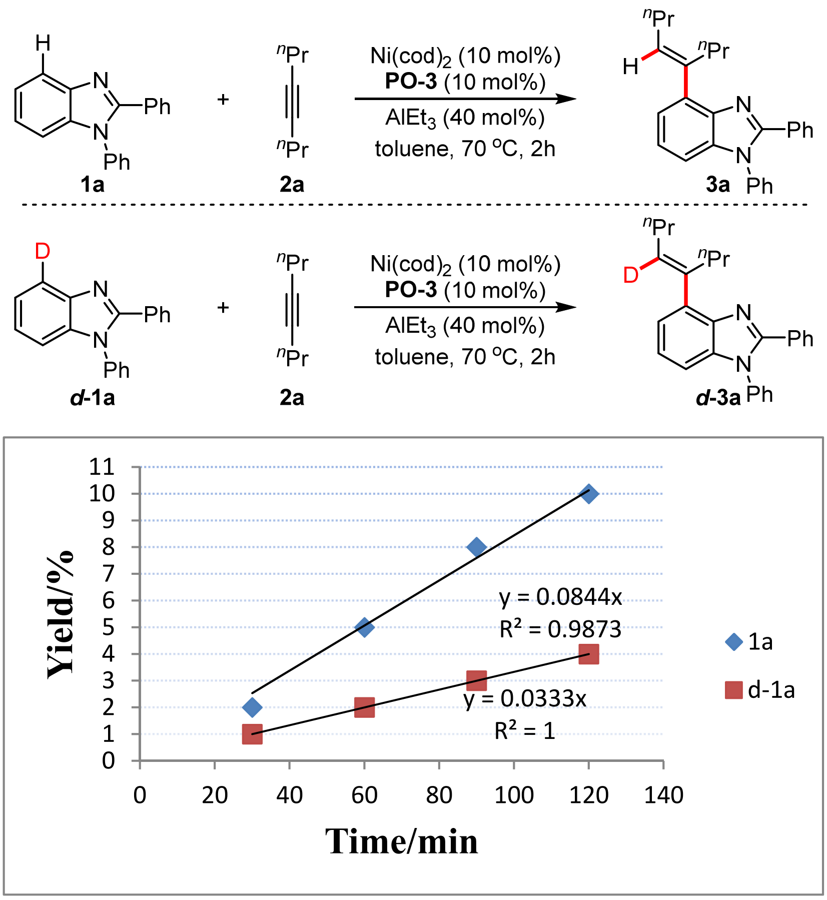

Supplementary Figure 2. Parallel reactions for KIE determination

Parallel reactions were set up following the general procedure at 70 °C by using **1a** and ***d*-1a** as substrate respectively. Aliquots were taken at 30 minute intervals for the first two hours. Product yield was determined by ^{1}H NMR using CH_2Br_2 as an internal standard. Data points represent the average of two runs. KIE = k_H/k_D = 0.0844/0.0333 = 2.53.

Intermolecular Competitive Reaction

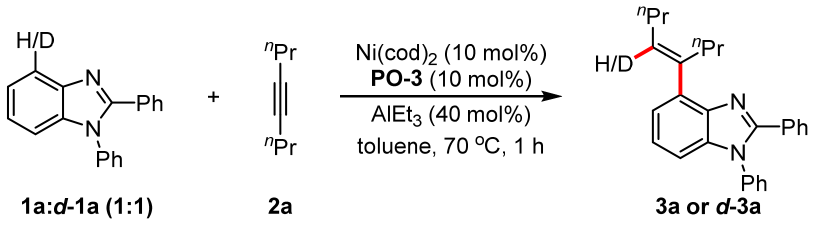

To a 15 mL oven dried tube were added **PO-3** (8.6 mg, 10 mol%), Ni(cod)$_2$ (5.6 mg, 10 mol%), dry degassed toluene (1.0 mL), **1a** (27.0 mg, 0.1 mmol), ***d*-1a** (27.0 mg, 0.1 mmol), $AlEt_3$ (80 µL, 40 mol%) and **2a** (44.1 mg, 0.4 mmol) sequentially in an N_2-filled glove-box. The tube was sealed and removed out of the glove-box. After heated at 70 °C in a dry block heater for 1 h, the mixture was cooled to r.t. and quenched with 0.6 mL of 5% EDTA disodium salt solution, then filtered through a short plug of silica gel (EtOAc as the eluent) and concentrated in vacuo. Further purification by flash column chromatography on silica gel gave the product **3a** and ***d*-3a** in 5% yield. The

KIE was obtained by calculation of the ratio of two products ($k_H/k_D = 0.68/0.32 = 2.13$).

Deuterium-labeling Experiment

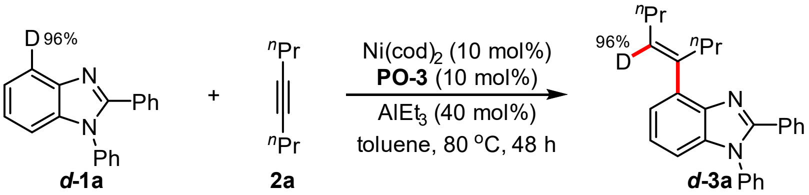

Same procedure as above.

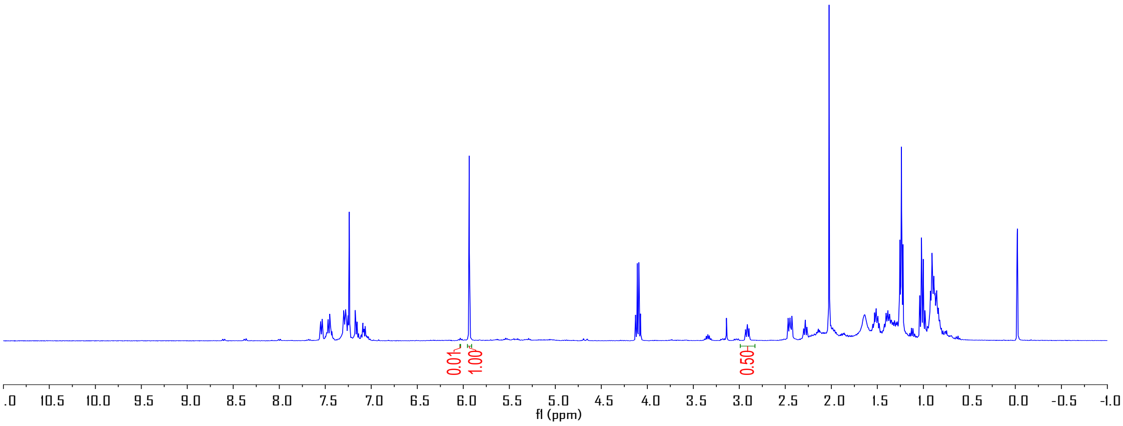

Supplementary Figure 3. Deuterium-labeling experiment

Supplementary Note 10

Crystal Structure Information of 4s

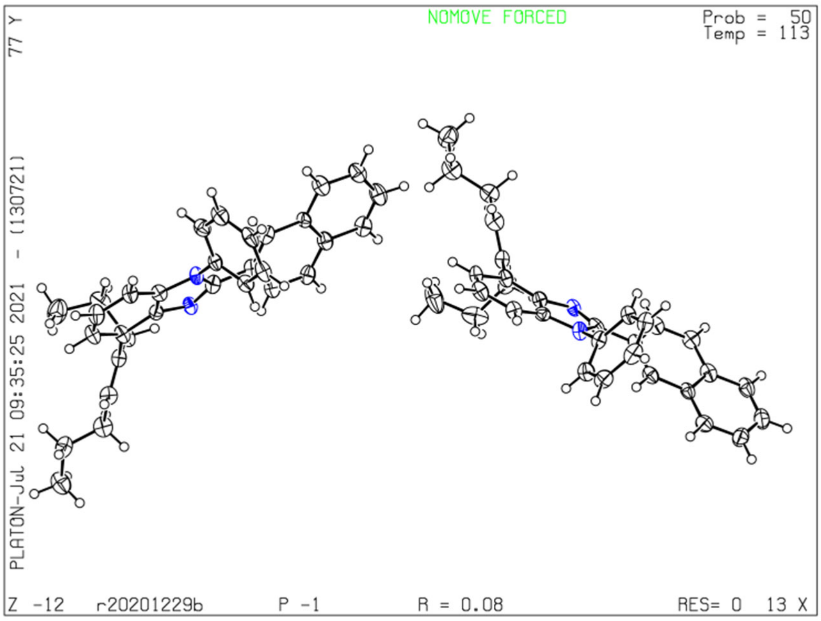

Supplementary Figure 4. Crystal structure of compound **4s**

Deposition Number 2101580
Compound Name:
(*E*)-2-(Naphthalen-2-yl)-4-(oct-4-en-4-yl)-1-phenyl-1*H*-benzo[*d*]imidazole
Data Block Name: data_r20201229b
Unit Cell Parameters: a 9.3395(4) b 9.8937(6) c 26.4722(17) P-1

Supplementary Figures

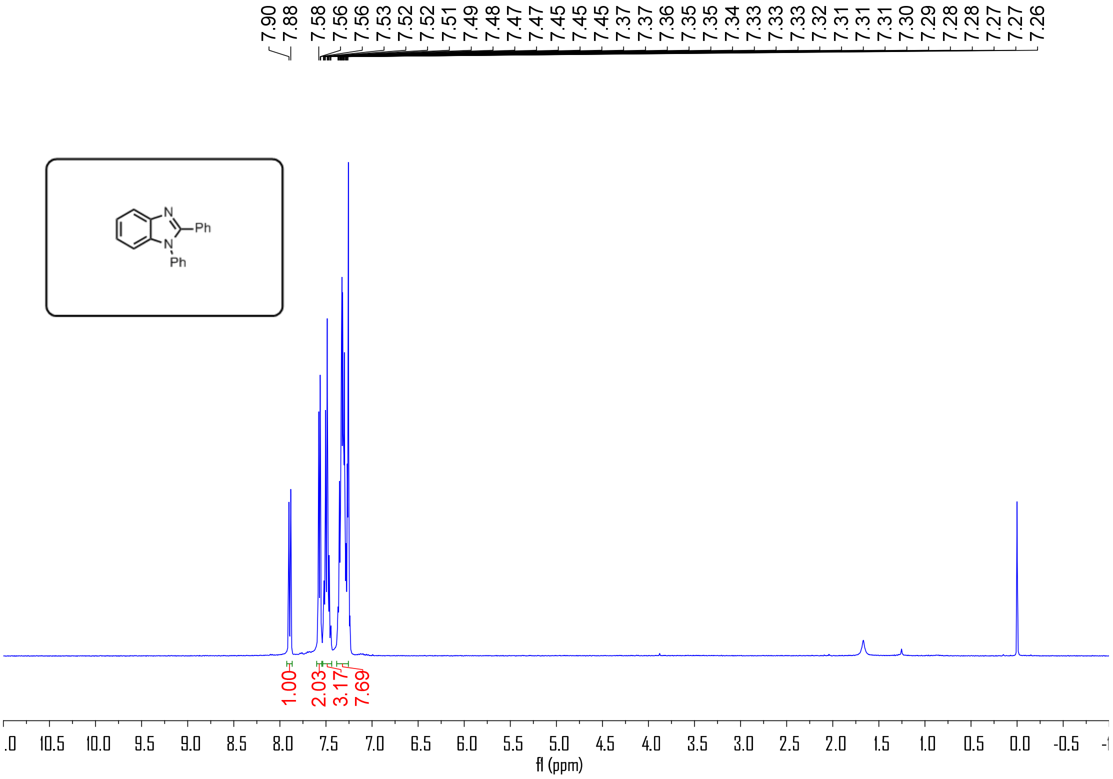

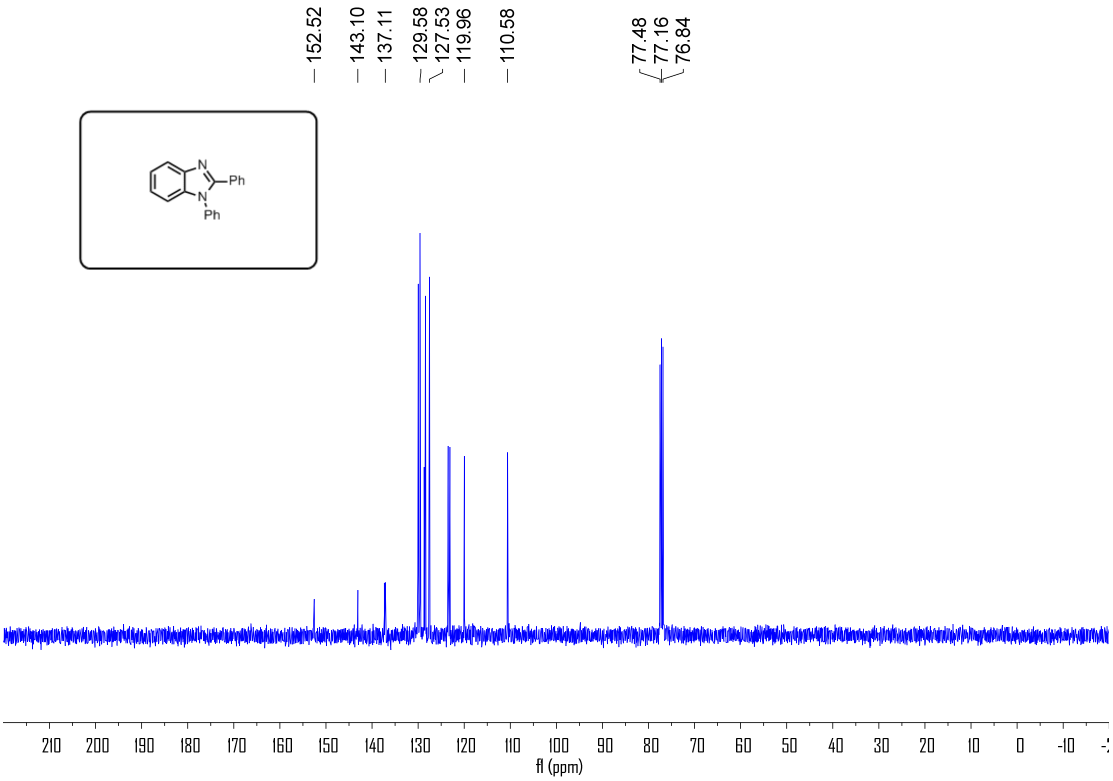

Supplementary Figure 5. ^{1}H and ^{13}C NMR spectra of **1a** in $CDCl_3$.

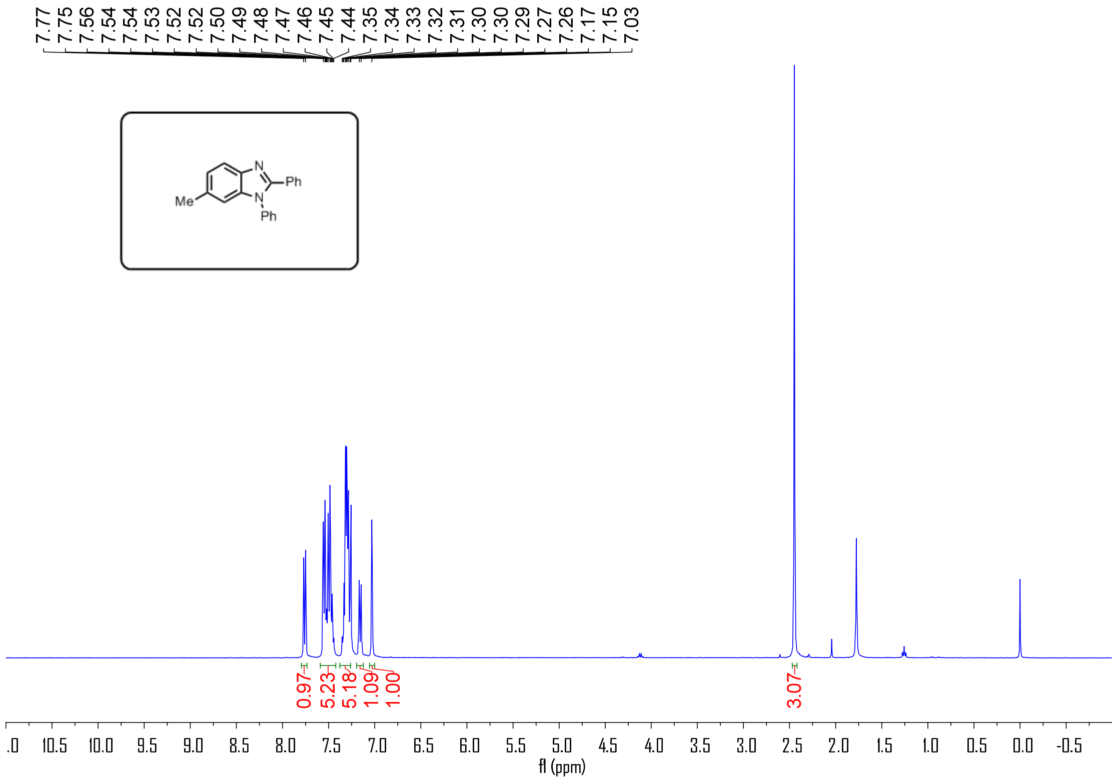

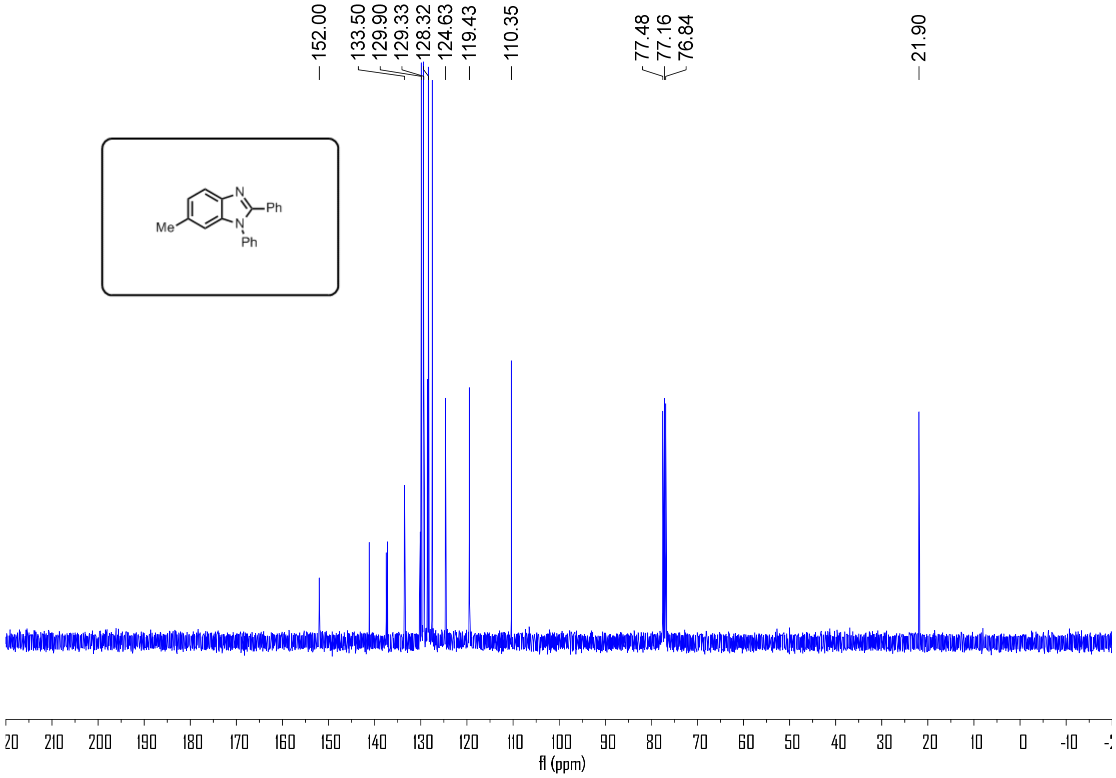

Supplementary Figure 6. ^{1}H and ^{13}C NMR spectra of **1b** in $CDCl_3$.

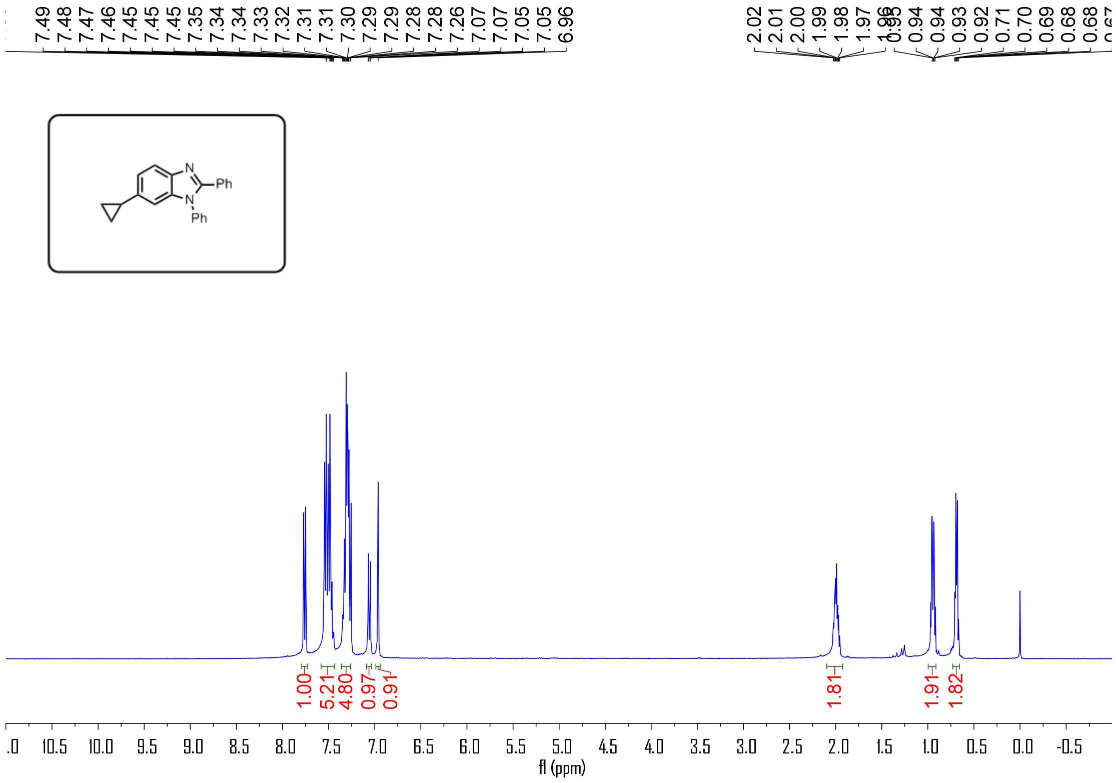

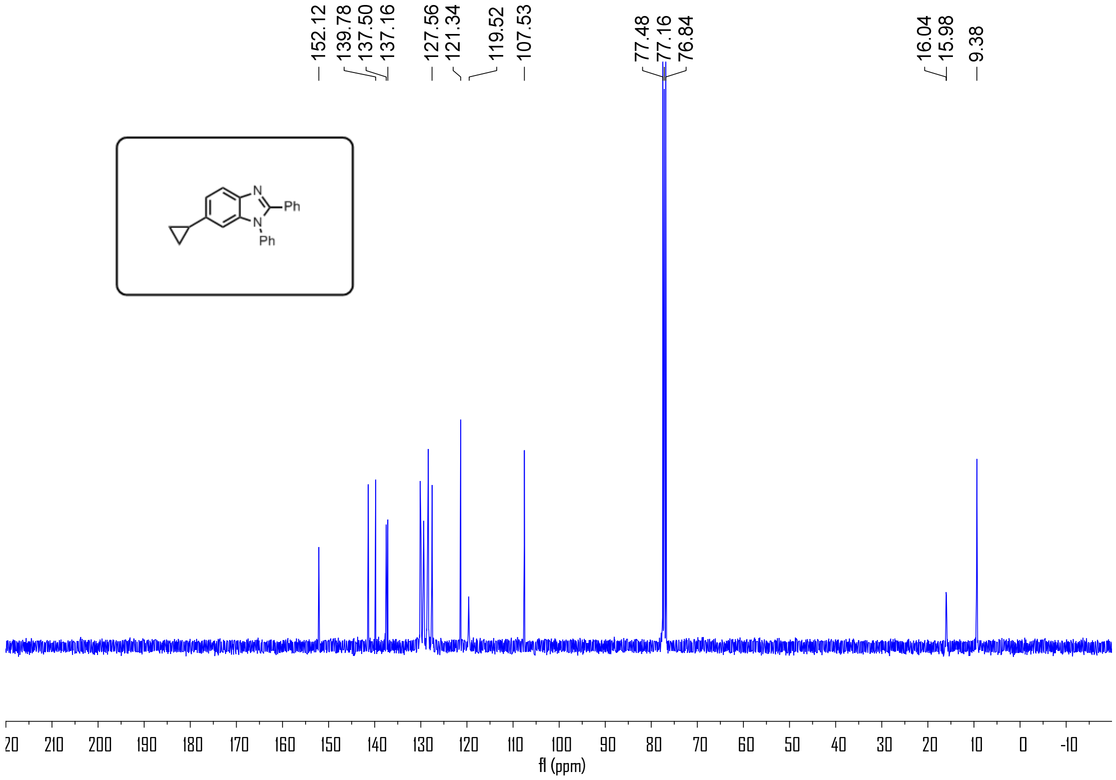

Supplementary Figure 7. ^{1}H and ^{13}C NMR spectra of **1c** in $CDCl_3$.

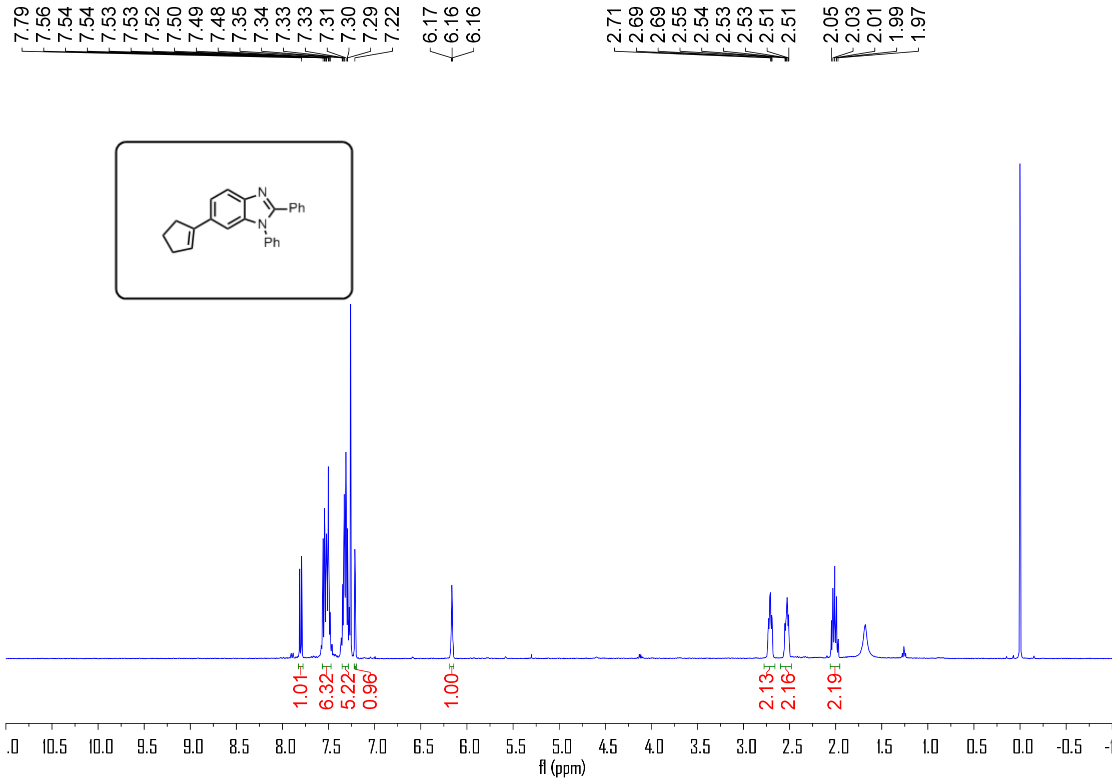

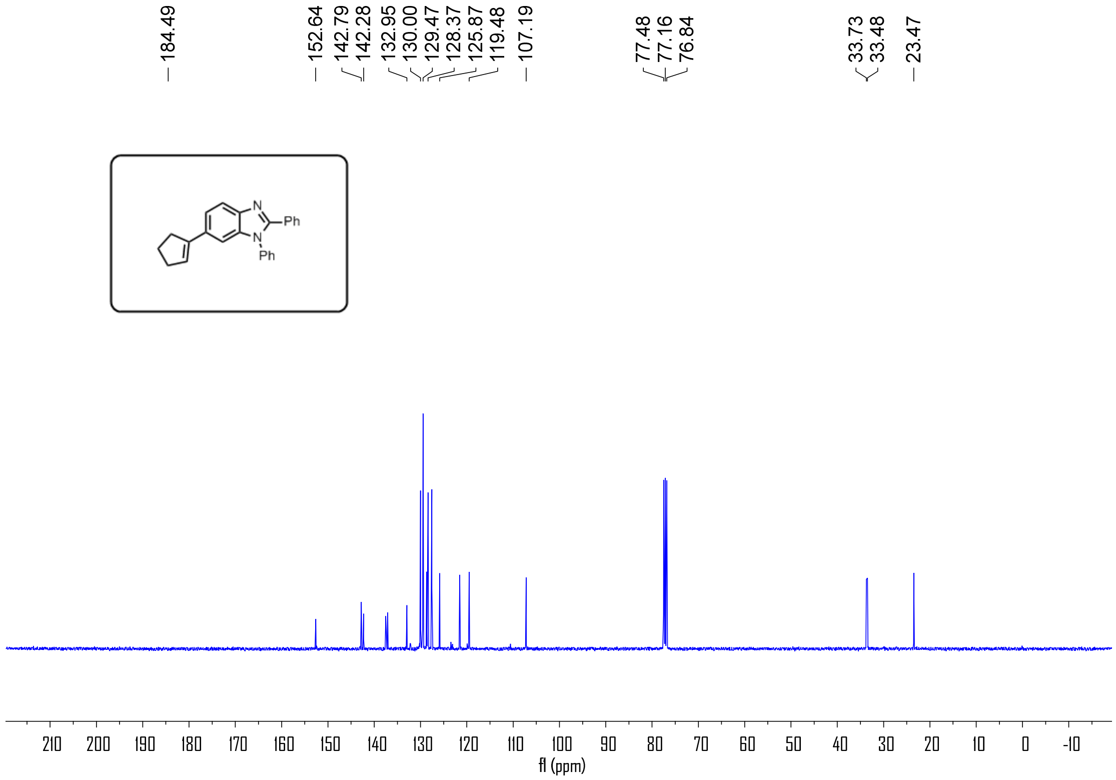

Supplementary Figure 8. ^{1}H and ^{13}C NMR spectra of **1d** in $CDCl_3$.

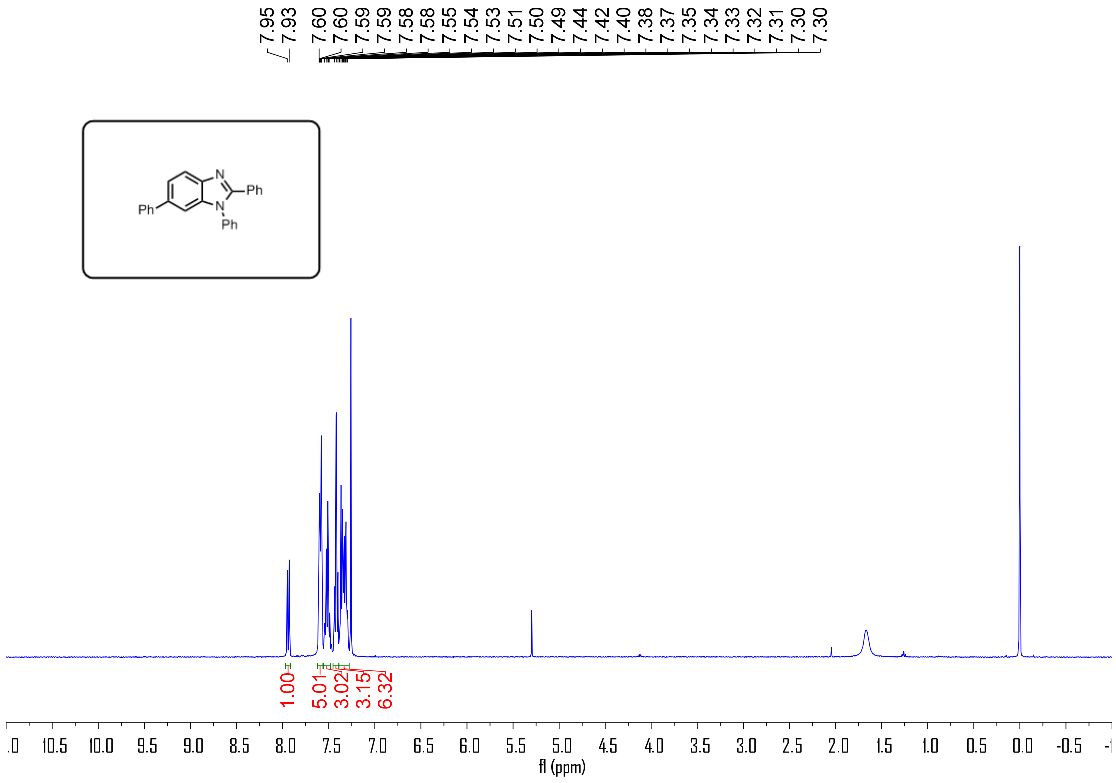

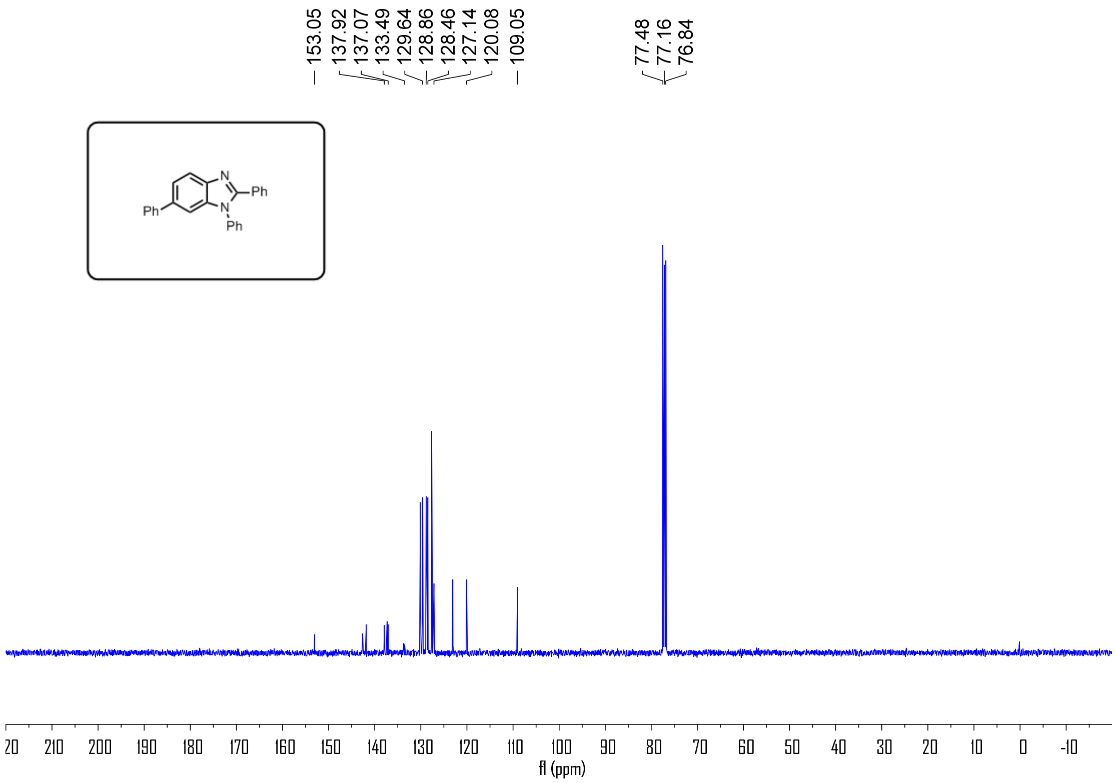

Supplementary Figure 9. ^{1}H and ^{13}C NMR spectra of **1e** in $CDCl_3$.

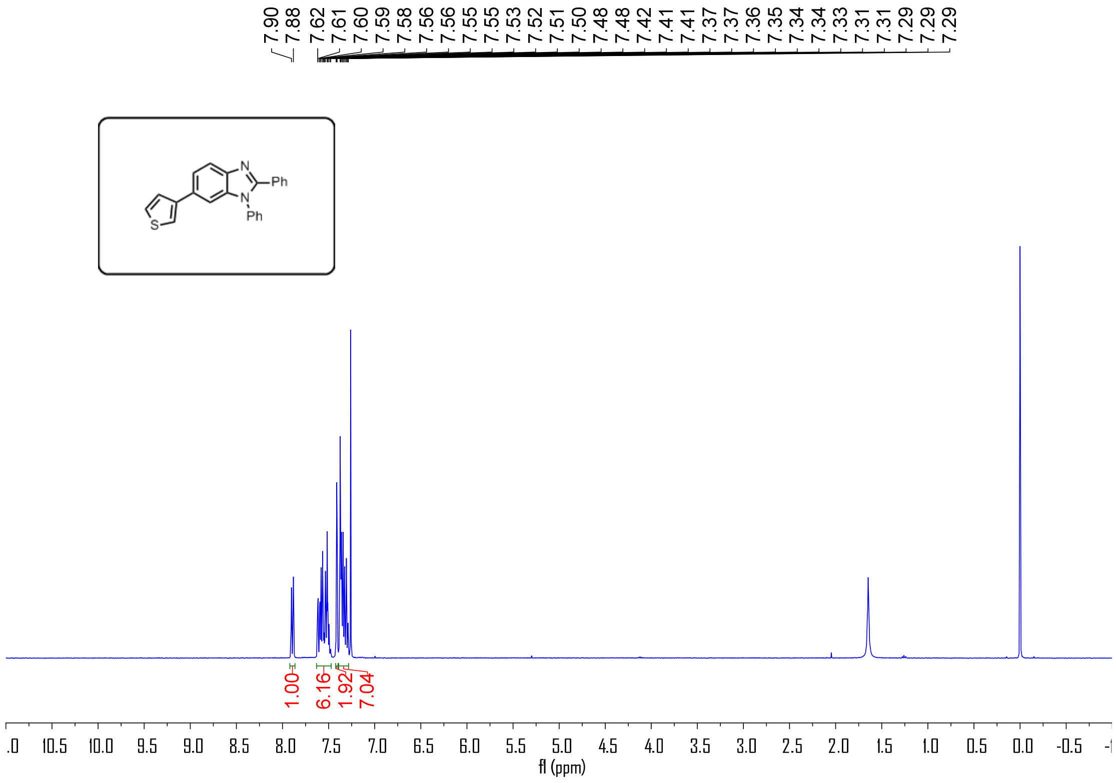

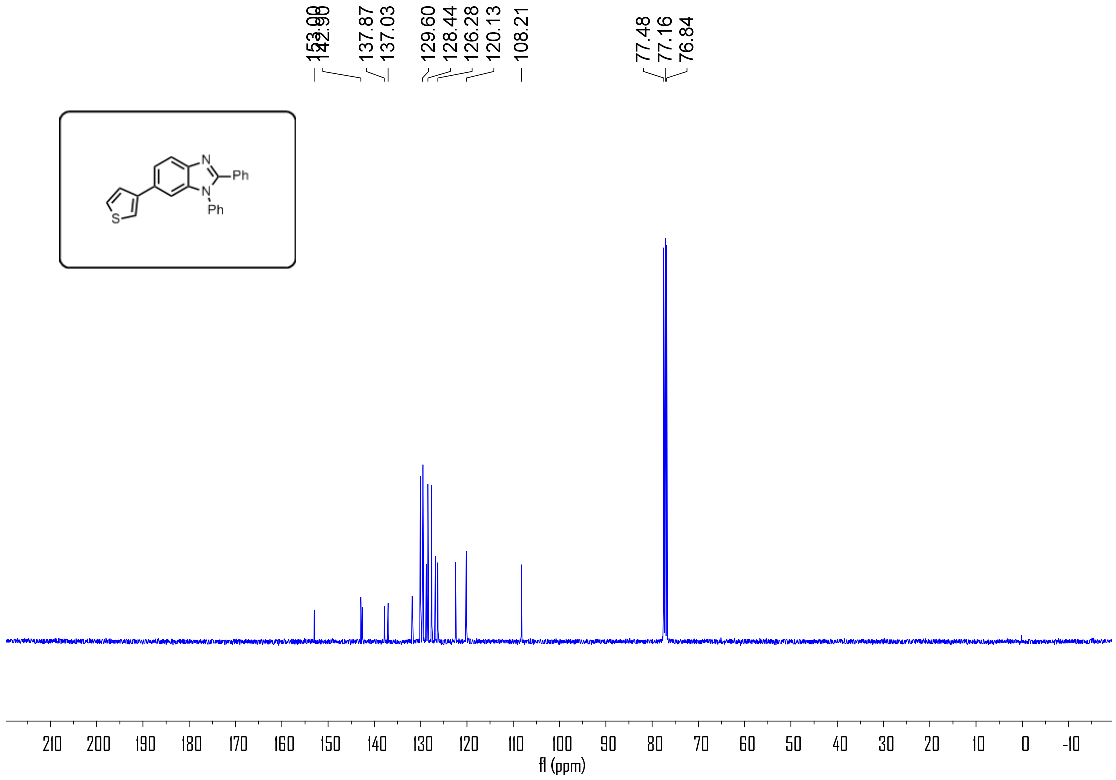

Supplementary Figure 10. ^{1}H and ^{13}C NMR spectra of **1f** in $CDCl_3$.

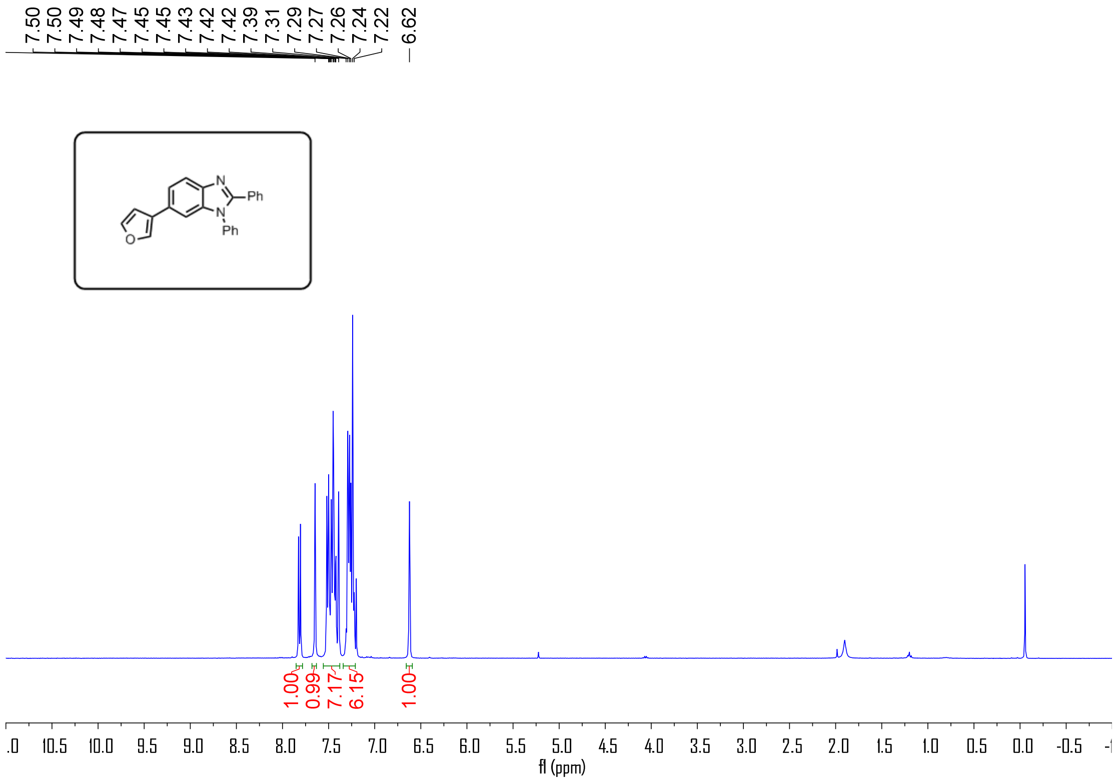

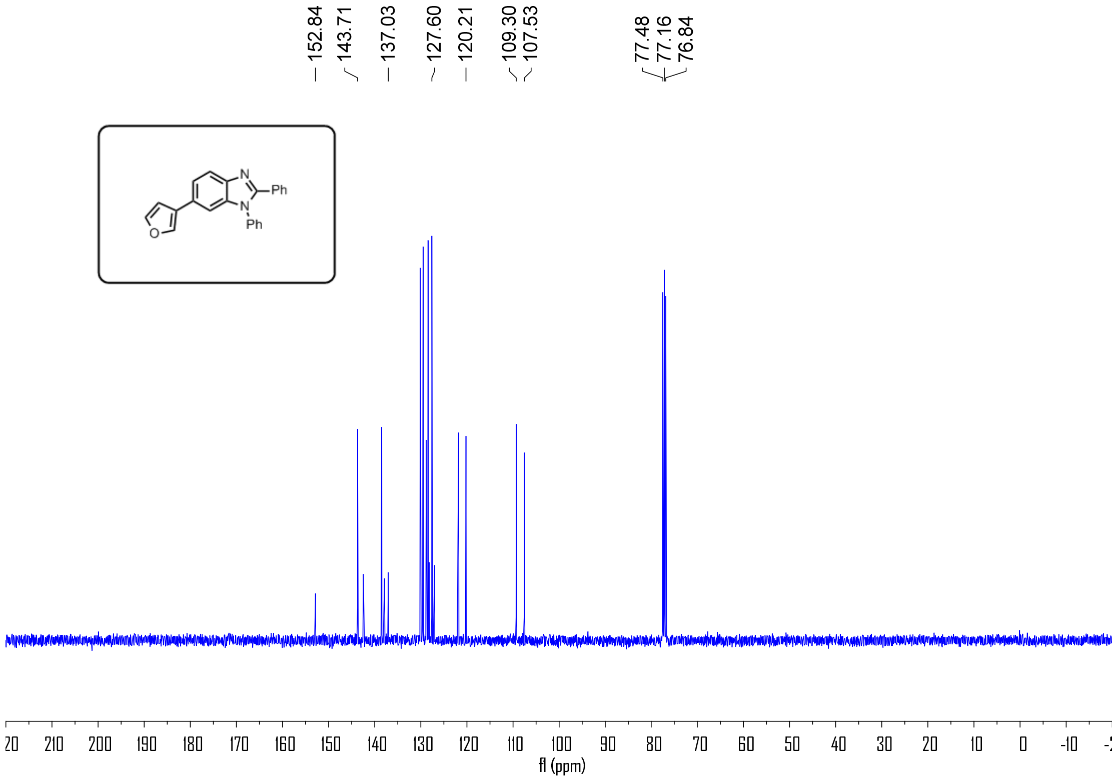

Supplementary Figure 11. ^{1}H and ^{13}C NMR spectra of **1g** in $CDCl_3$.

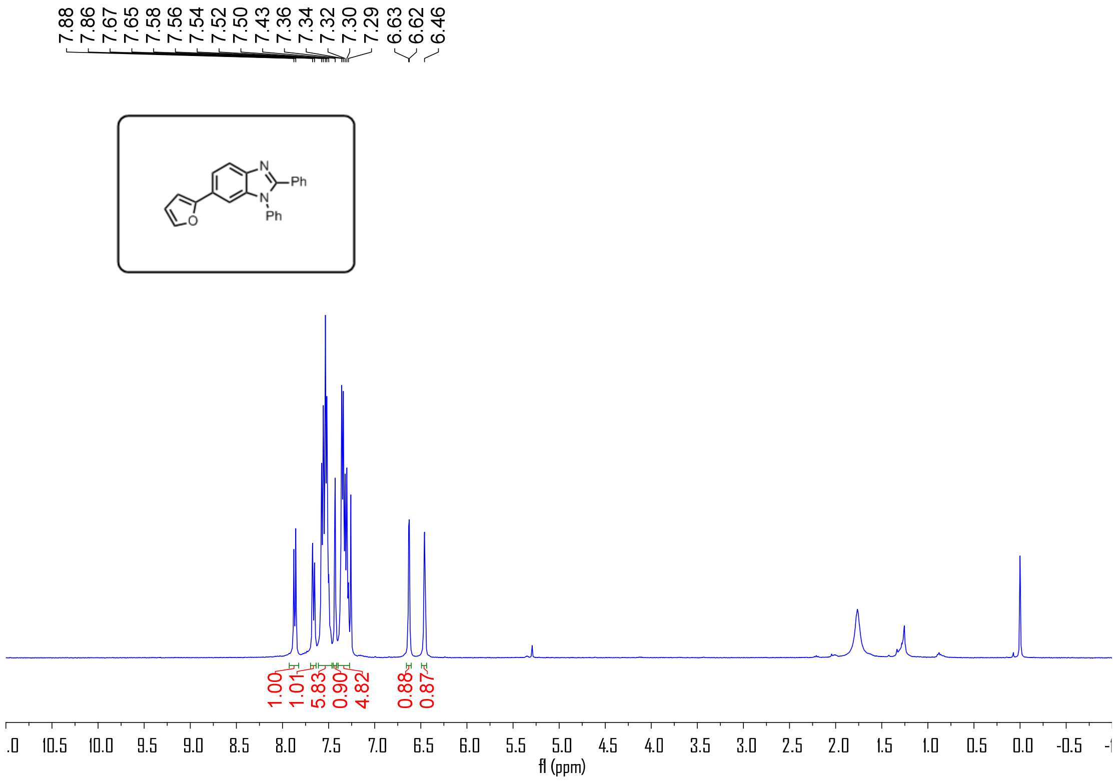

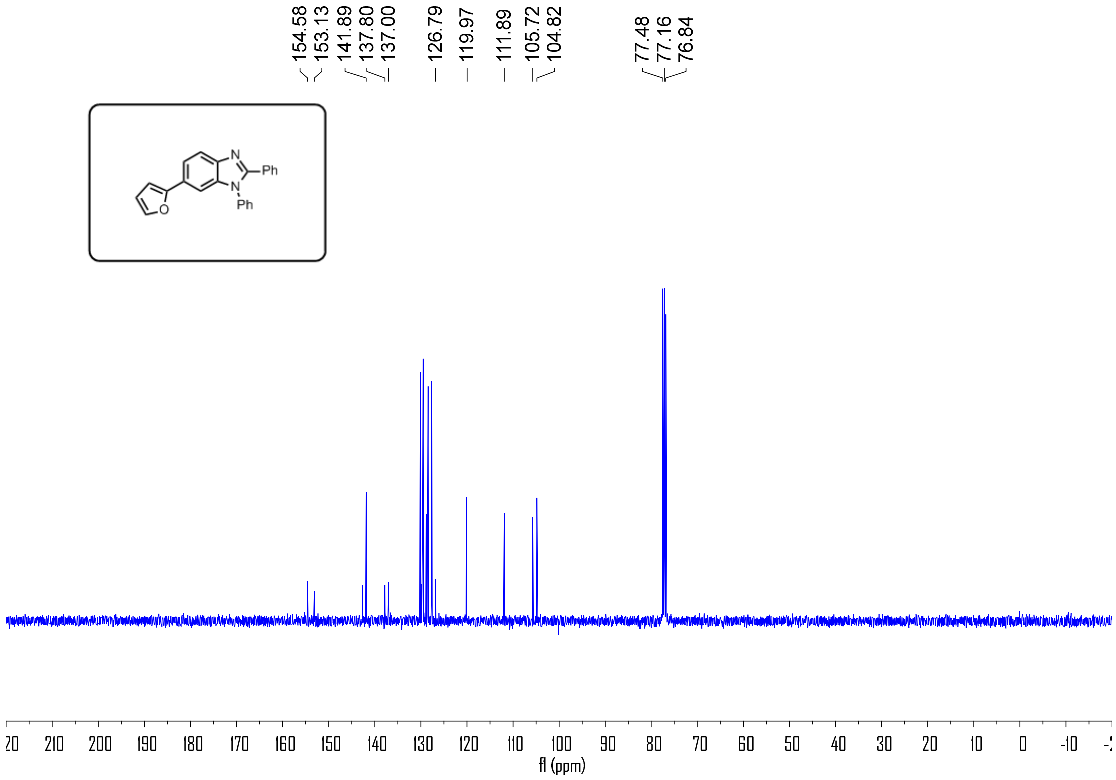

Supplementary Figure 12. ^{1}H and ^{13}C NMR spectra of **1h** in $CDCl_3$.

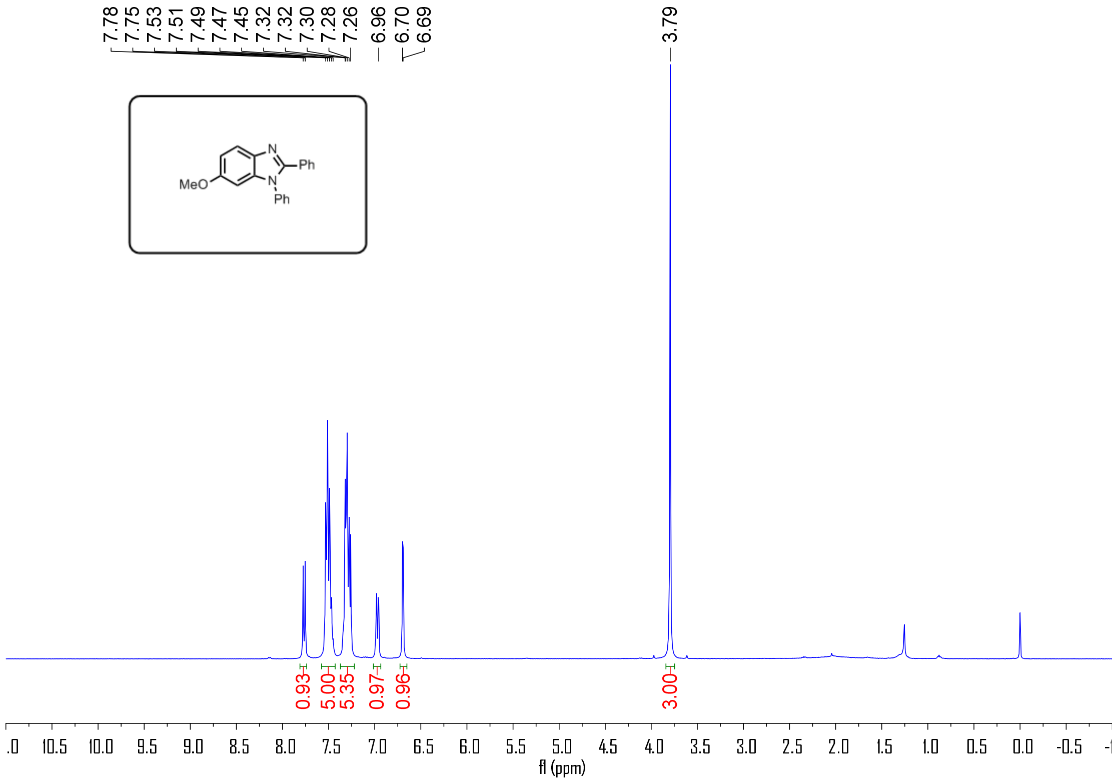

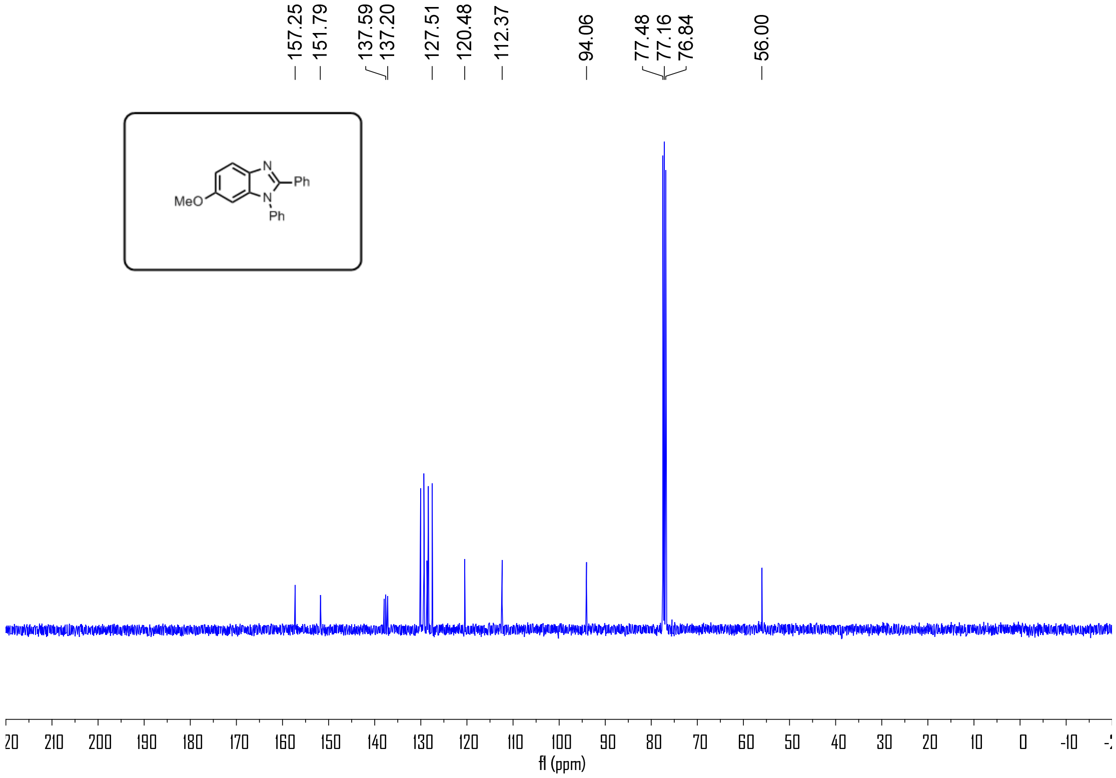

Supplementary Figure 13. ^{1}H and ^{13}C NMR spectra of **1i** in $CDCl_3$.

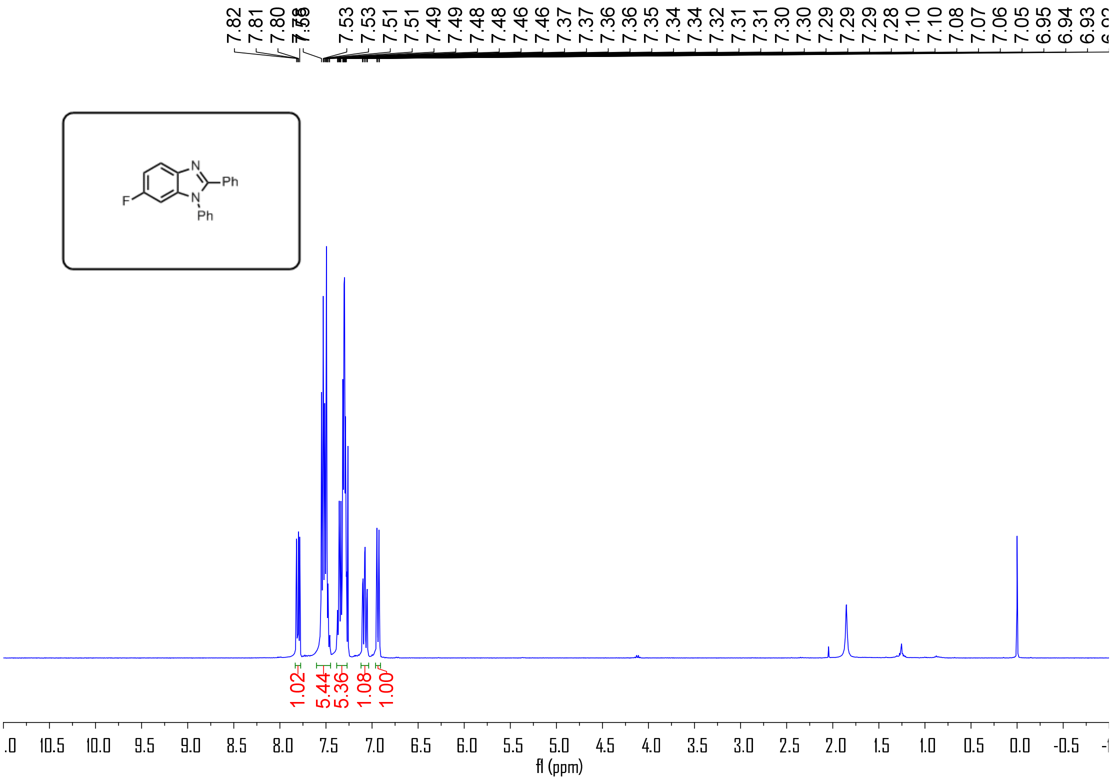

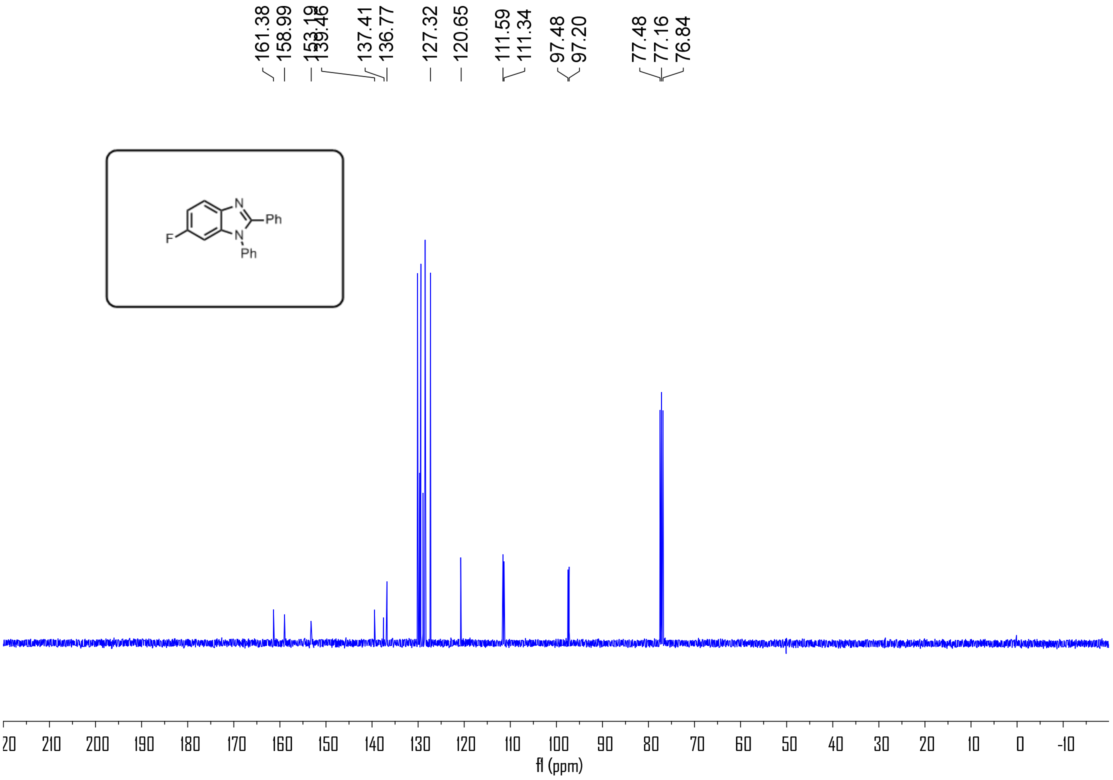

Supplementary Figure 14. ^{1}H and ^{13}C NMR spectra of **1j** in $CDCl_3$.

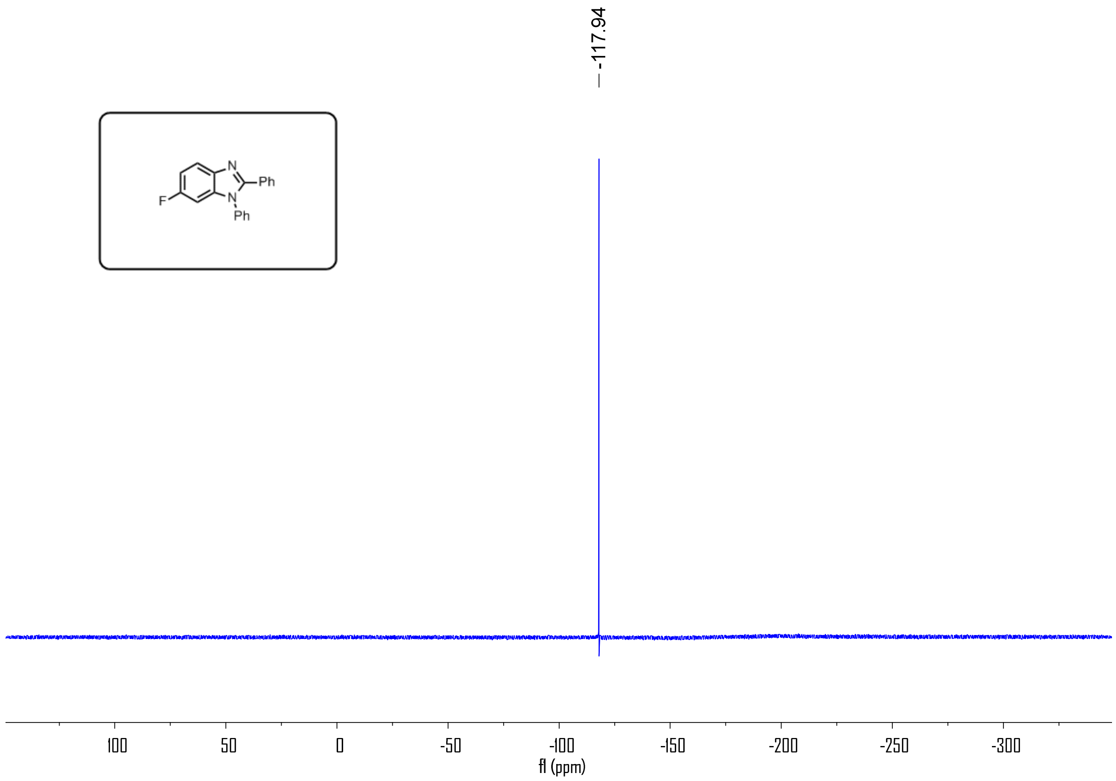

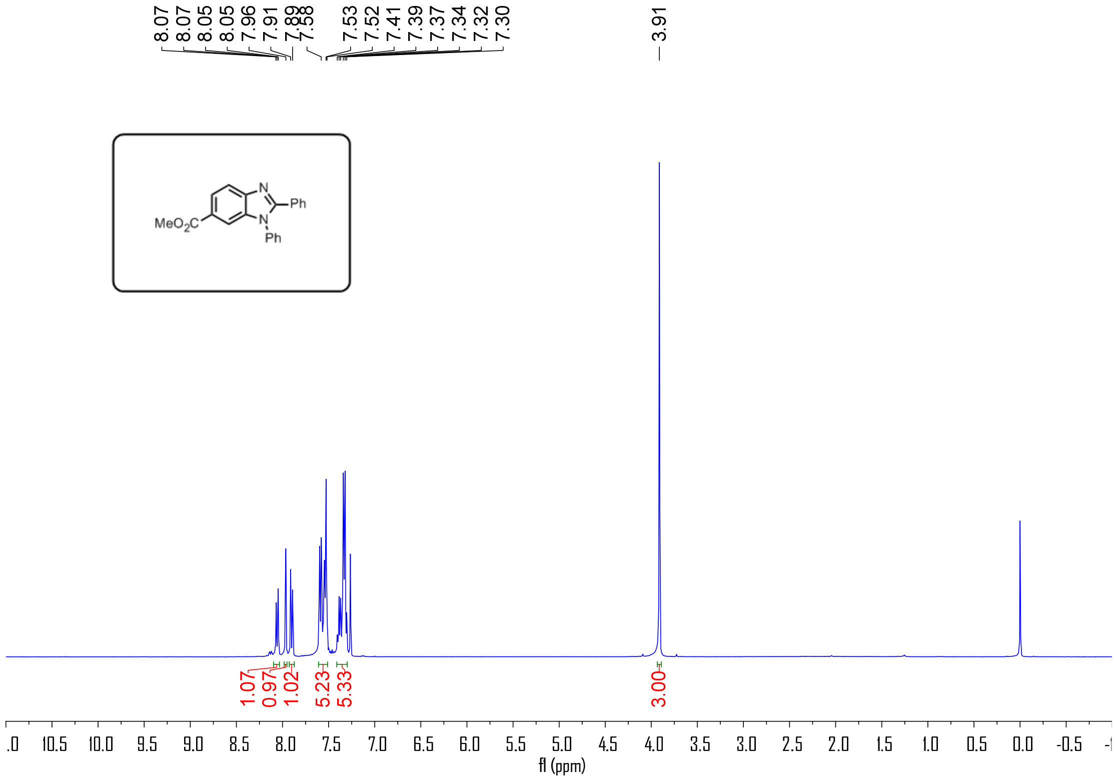

Supplementary Figure 15. ^{19}F (**1j**) and ^{1}H (**1k**) NMR spectra in $CDCl_3$.

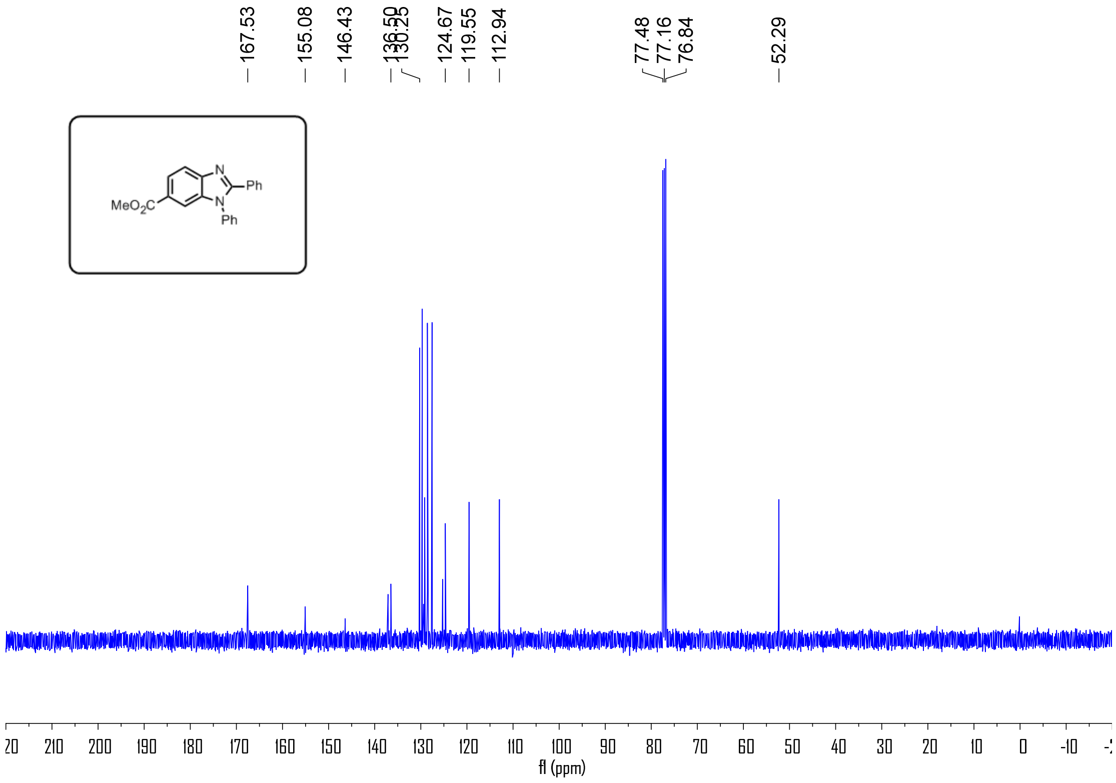

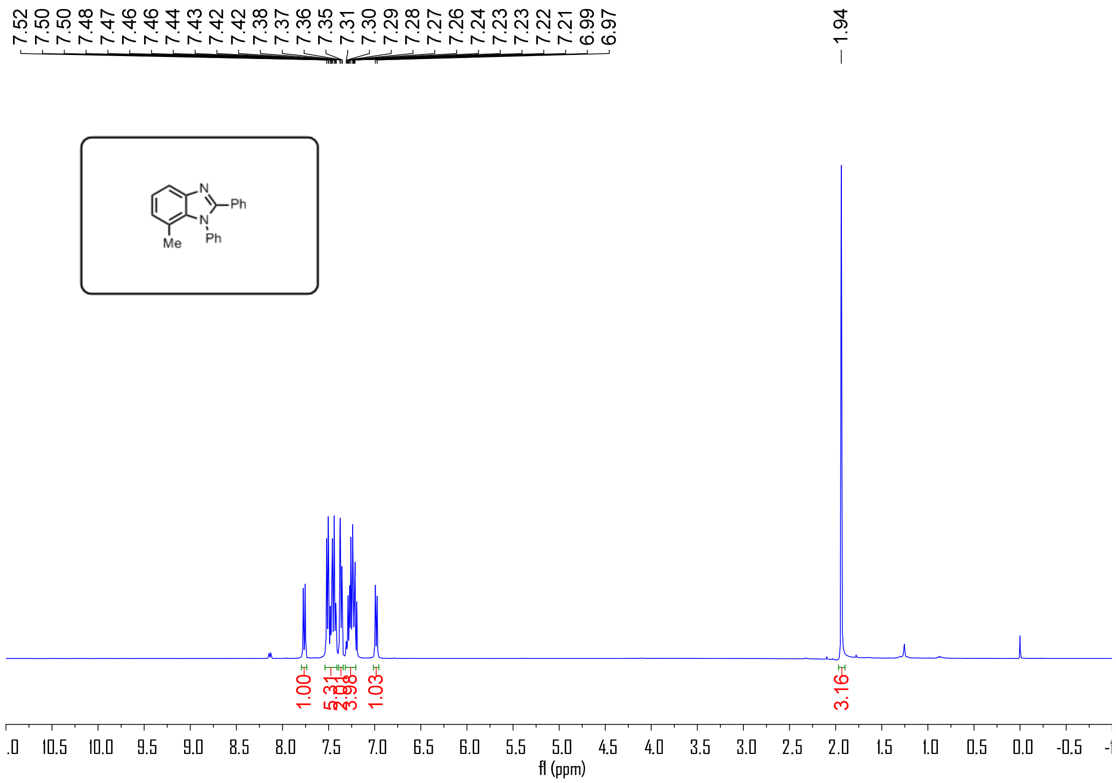

Supplementary Figure 16. ^{13}C (**1k**) and ^{1}H (**1l**) NMR spectra in $CDCl_3$.

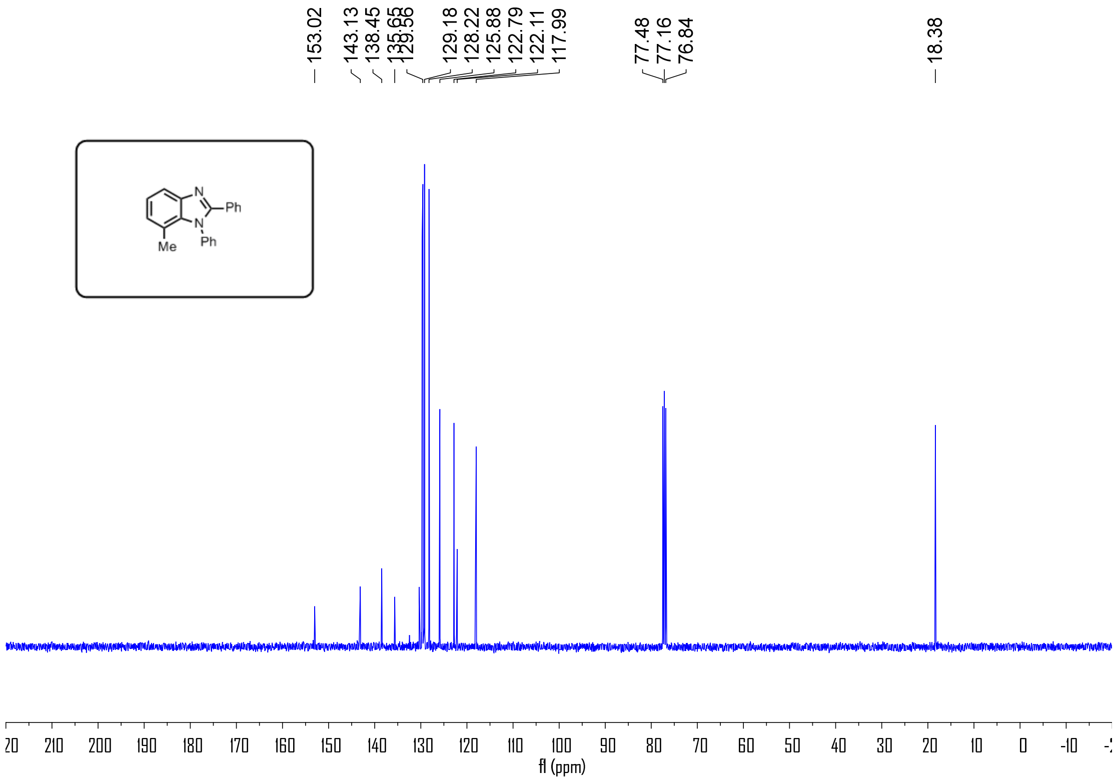

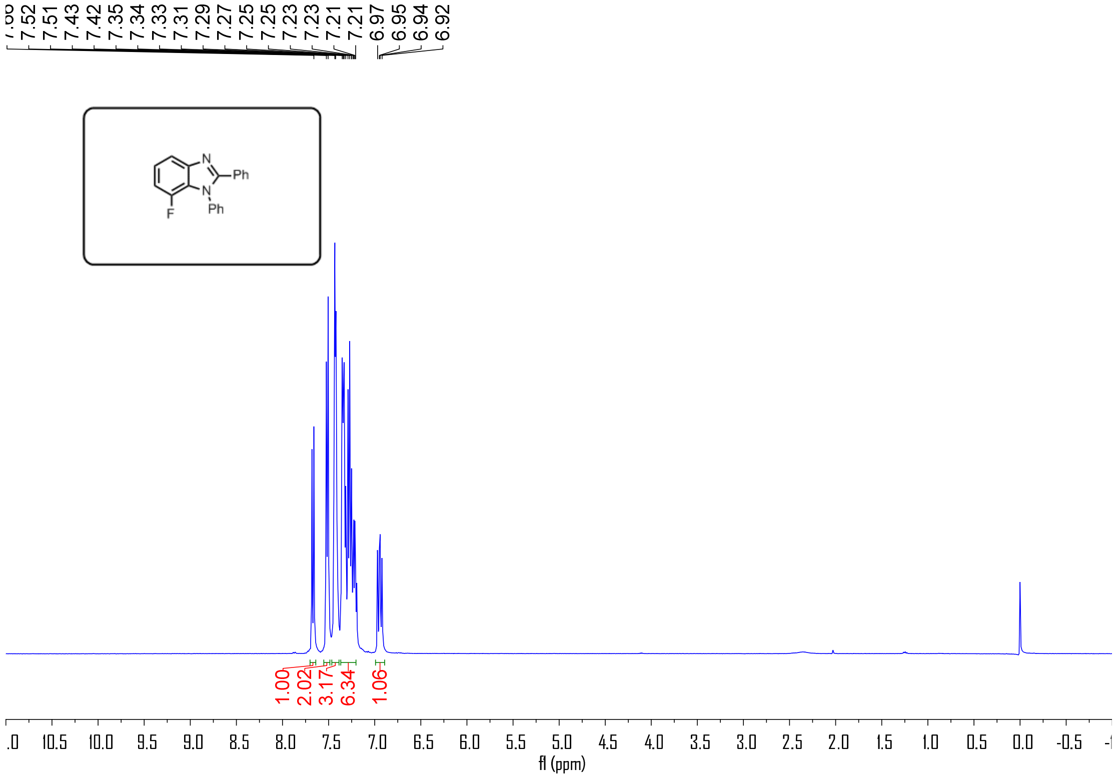

Supplementary Figure 17. ^{13}C (**1l**) and ^{1}H (**1m**) NMR spectra in $CDCl_3$.

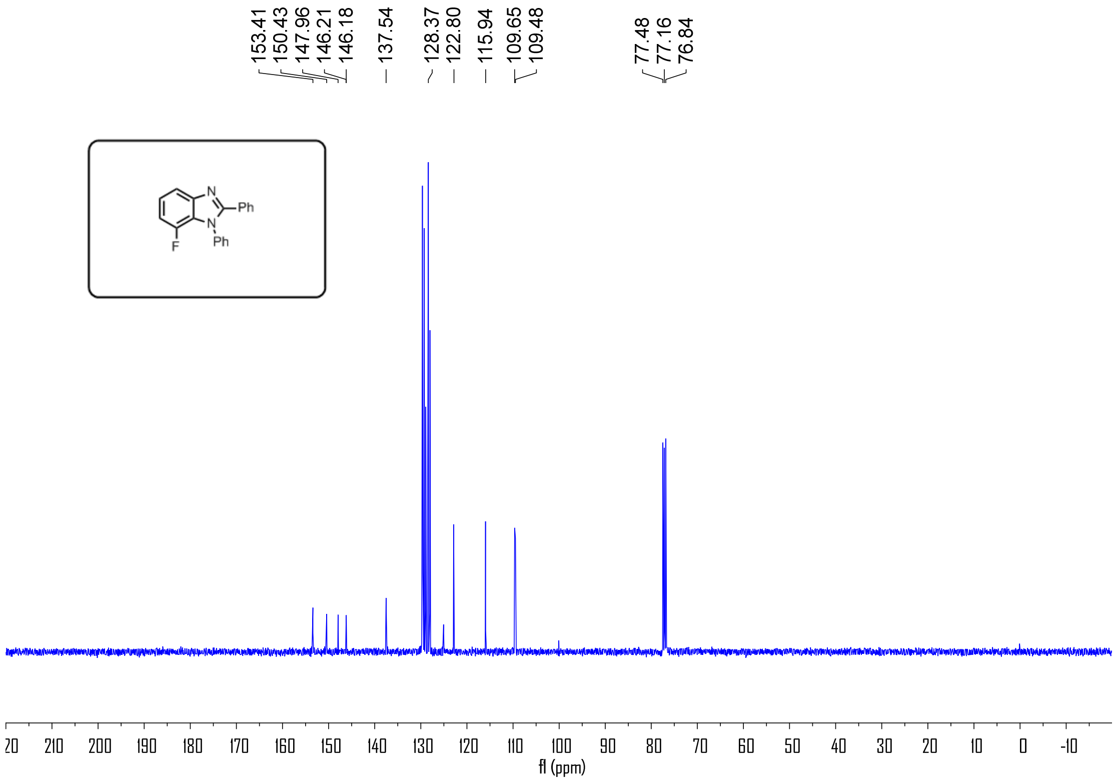

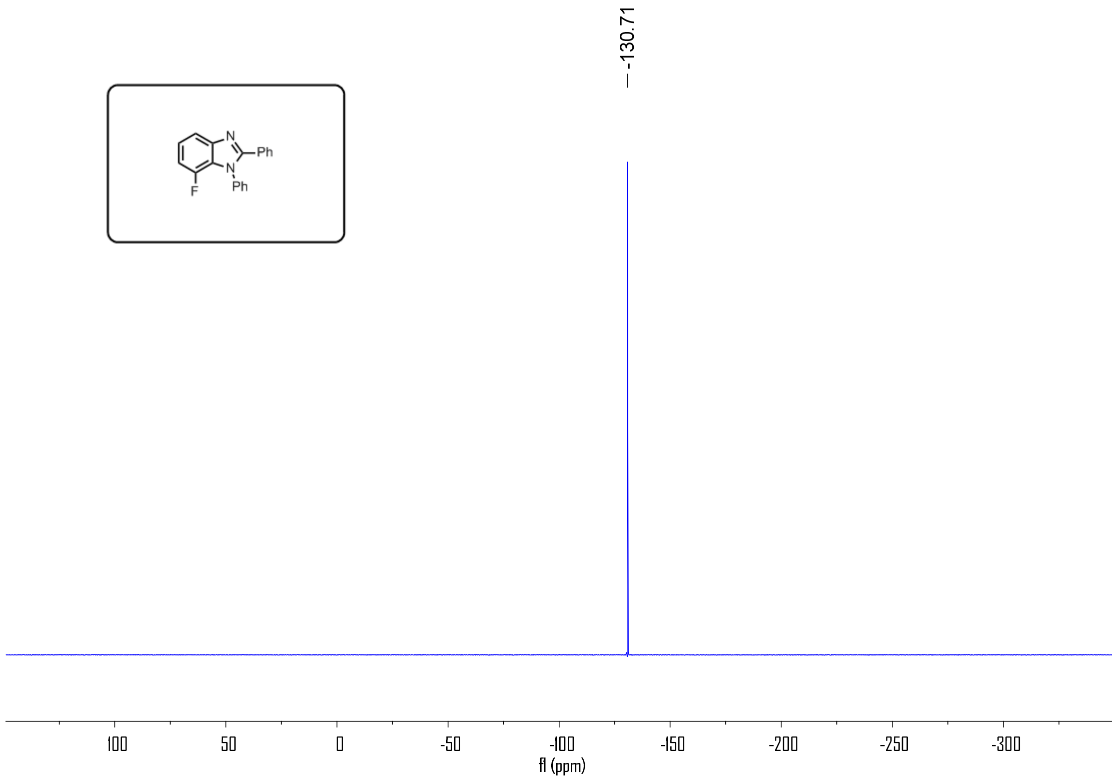

Supplementary Figure 18. ^{13}C and ^{19}F NMR spectra of **1m** in $CDCl_3$.

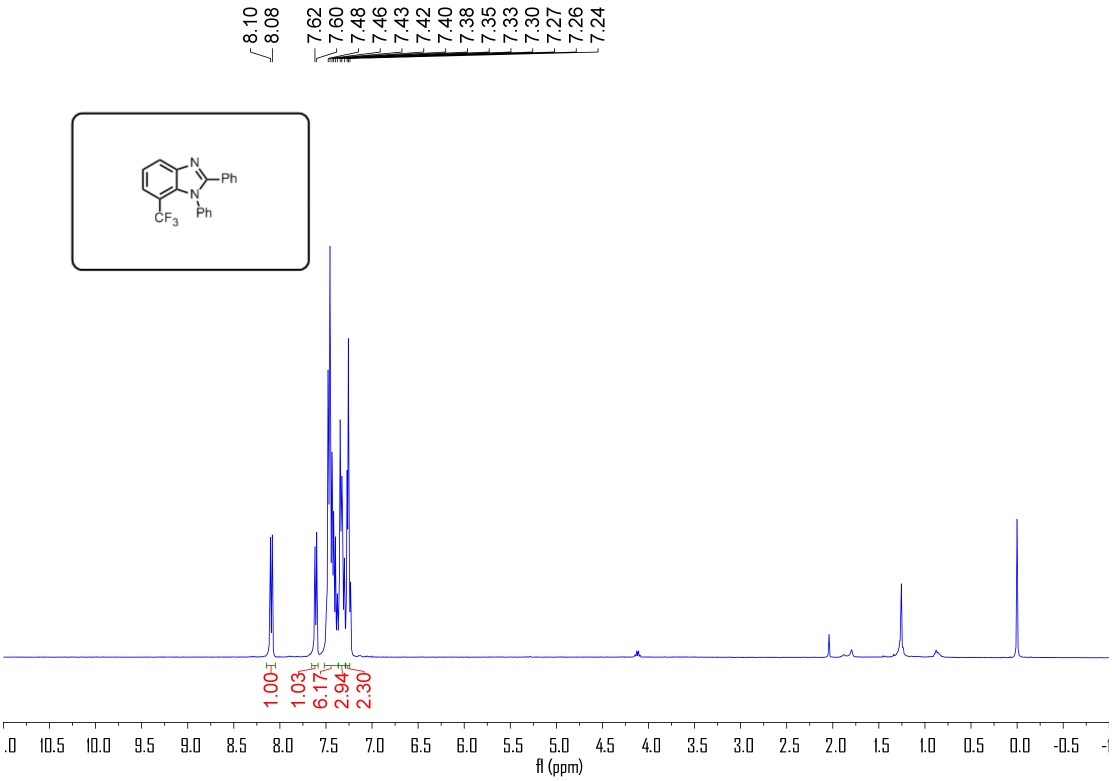

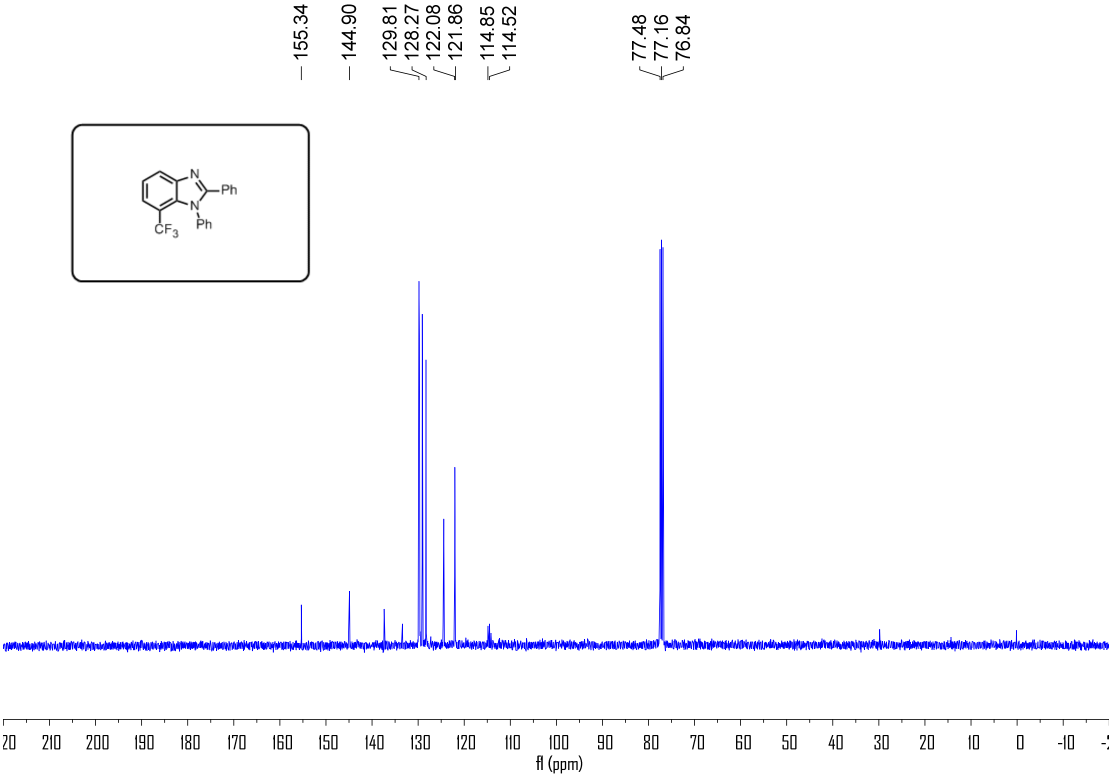

Supplementary Figure 19. ^{1}H and ^{13}C NMR spectra of **1n** in $CDCl_3$.

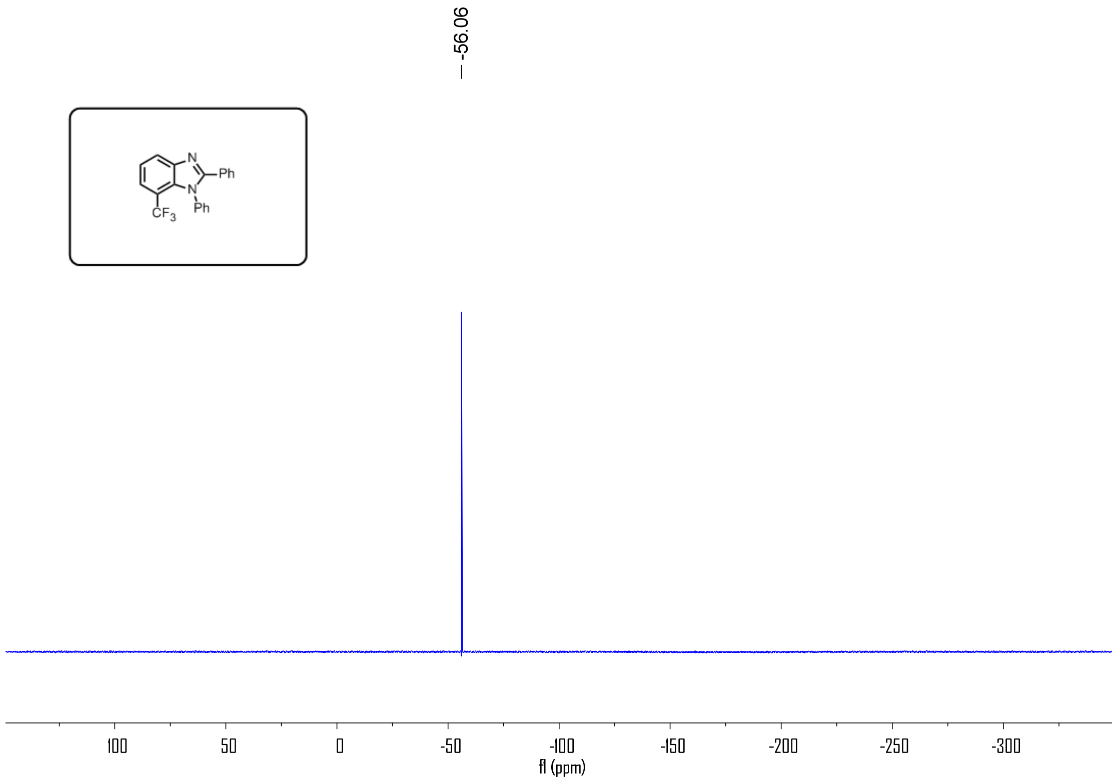

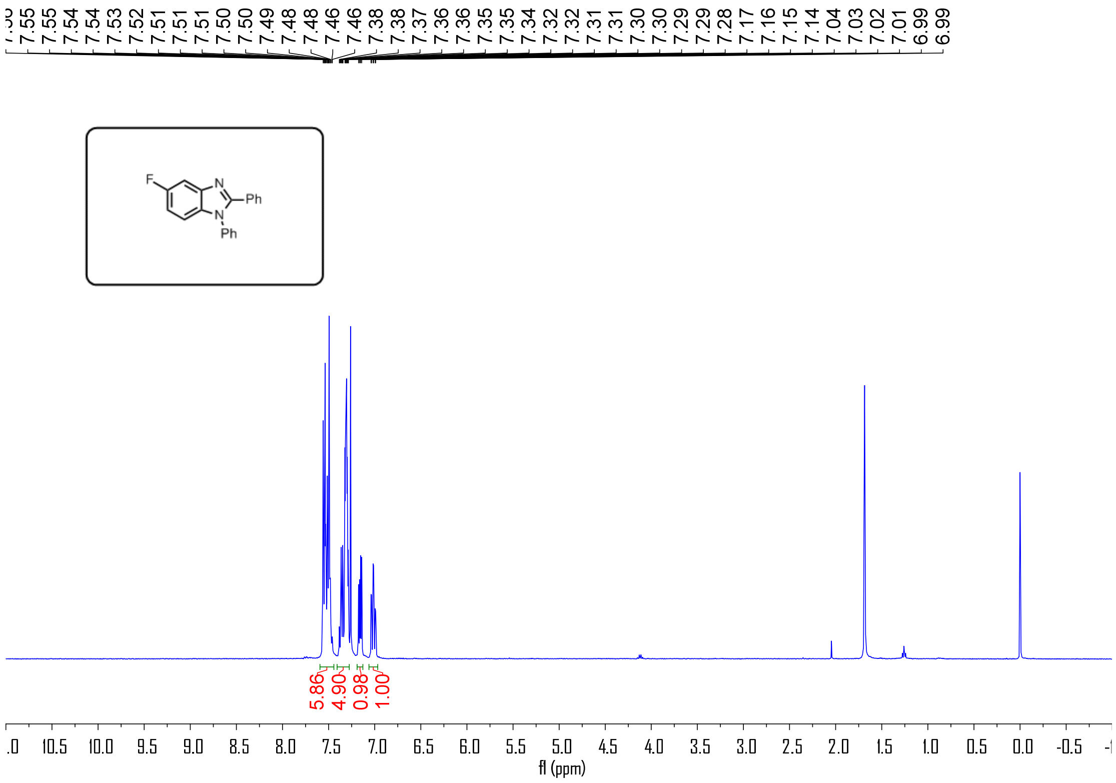

Supplementary Figure 20. ^{19}F (**1n**) and ^{1}H (**1o**) NMR spectra in $CDCl_3$.

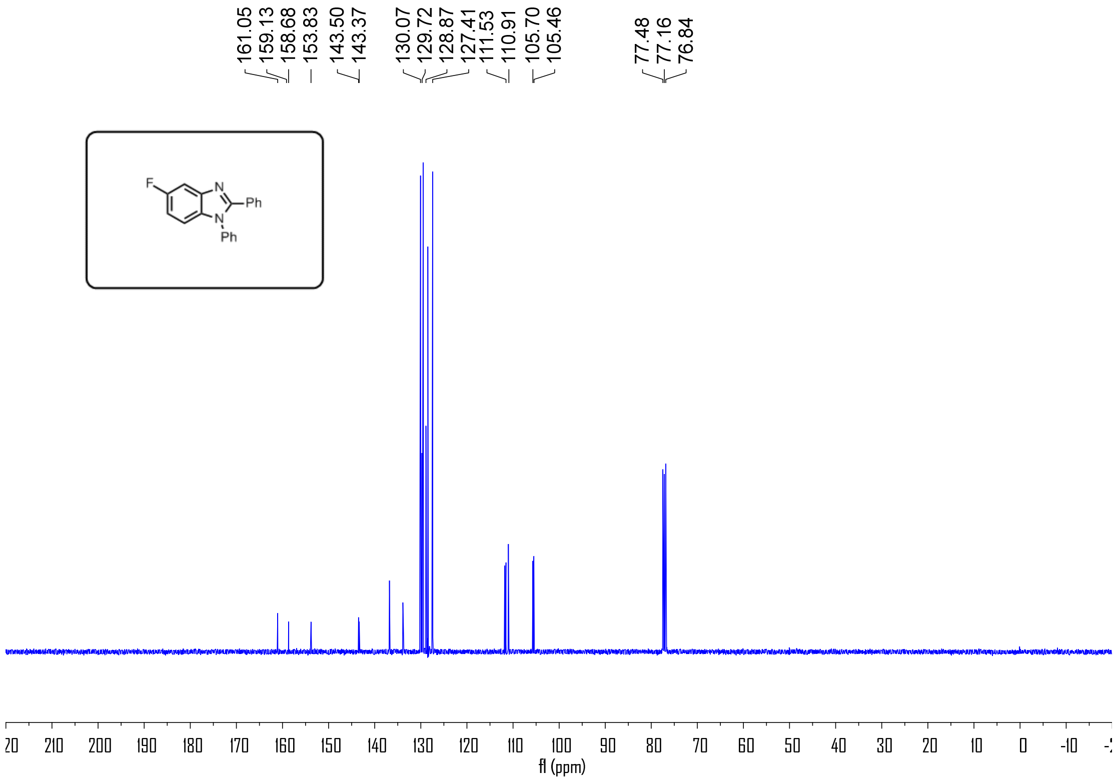

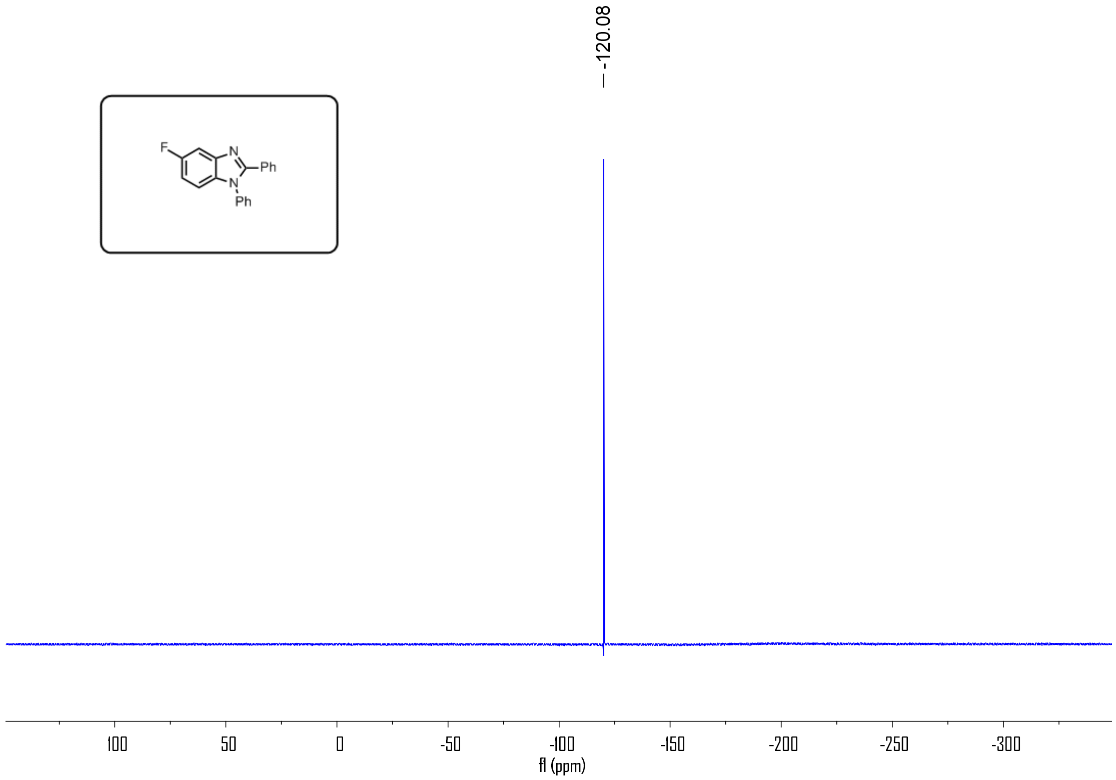

Supplementary Figure 21. ^{13}C and ^{19}F NMR spectra of **1o** in $CDCl_3$.

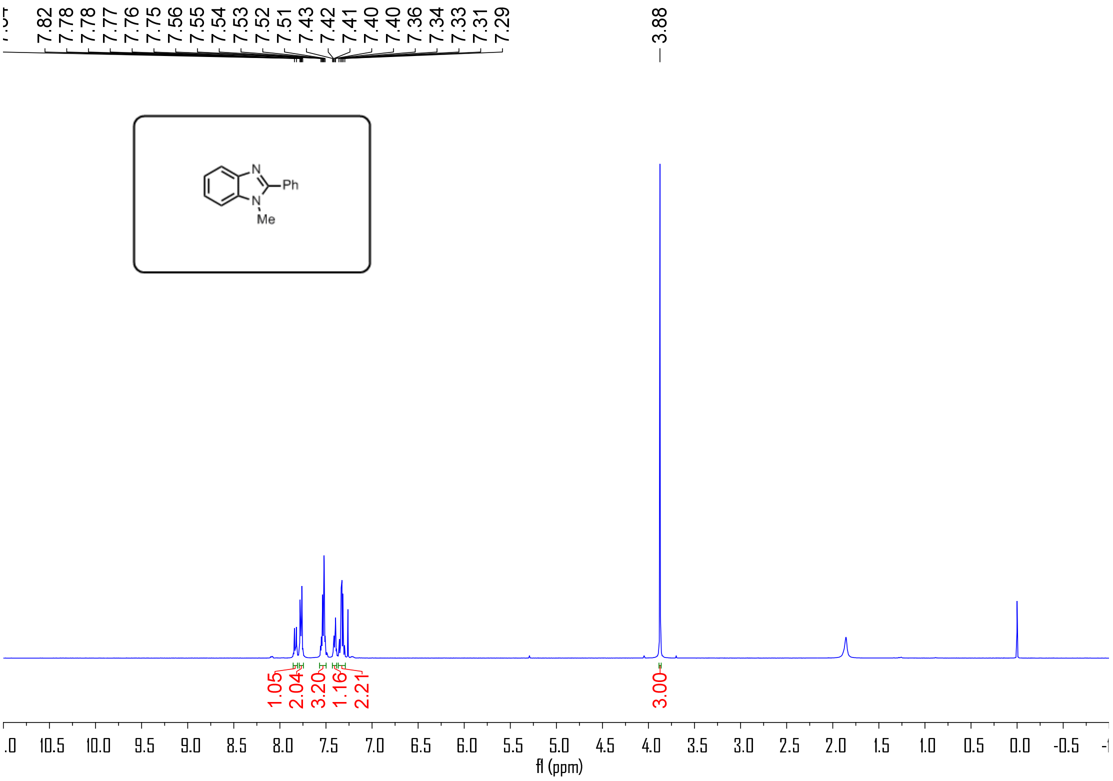

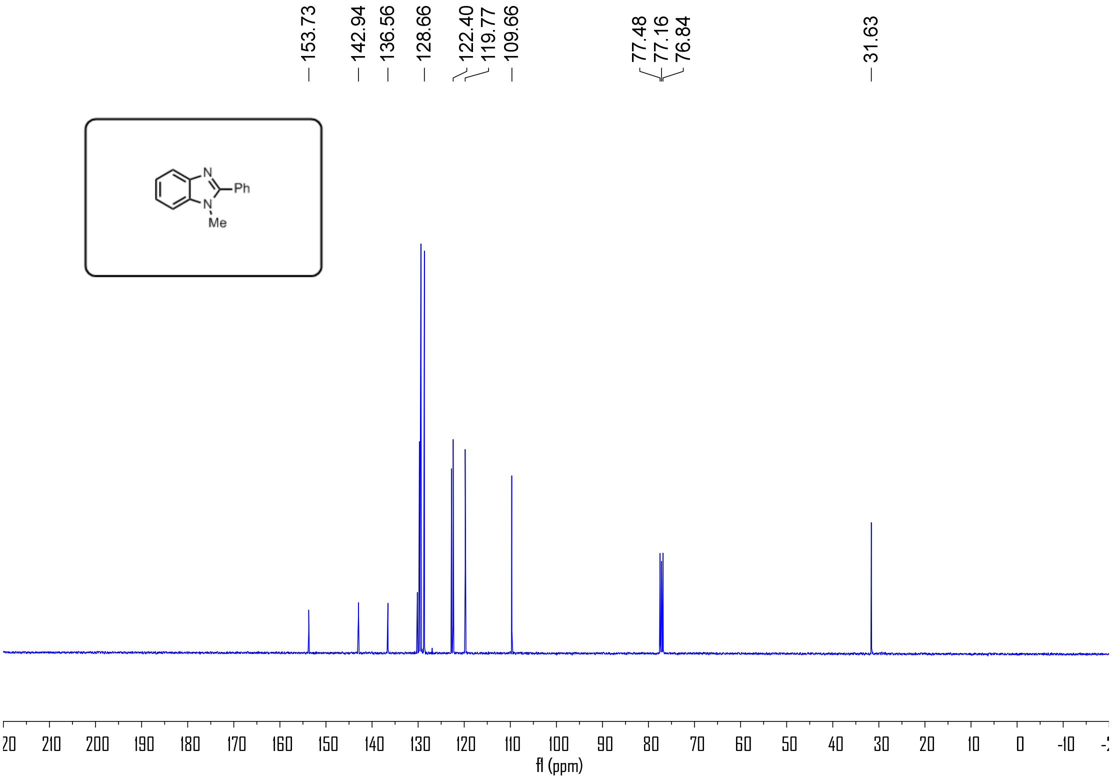

Supplementary Figure 22. ^{1}H and ^{13}C NMR spectra of **1p** in $CDCl_3$.

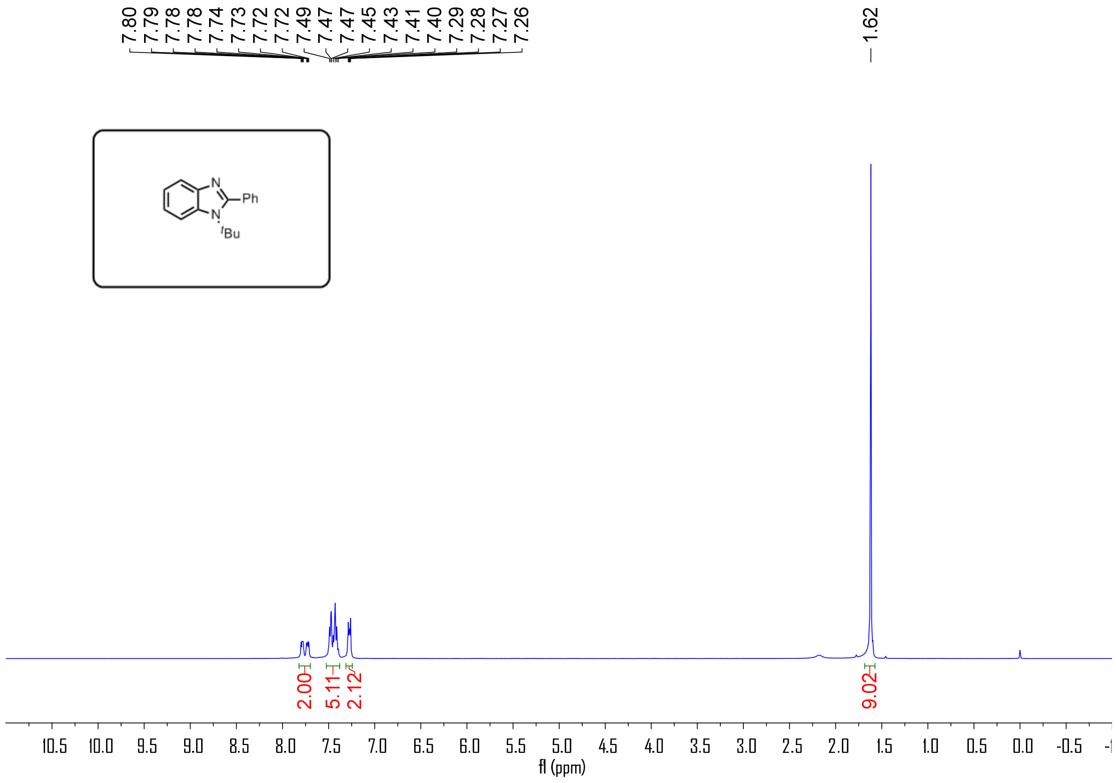

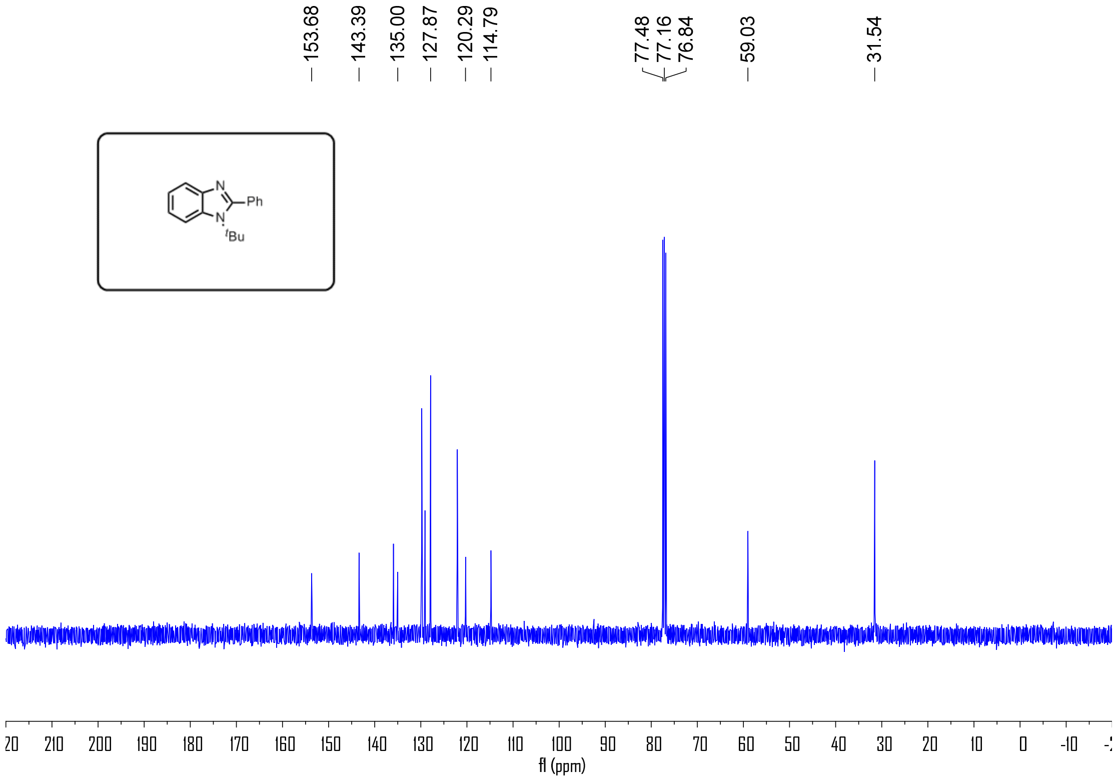

Supplementary Figure 23. ^{1}H and ^{13}C NMR spectra of **1q** in $CDCl_3$.

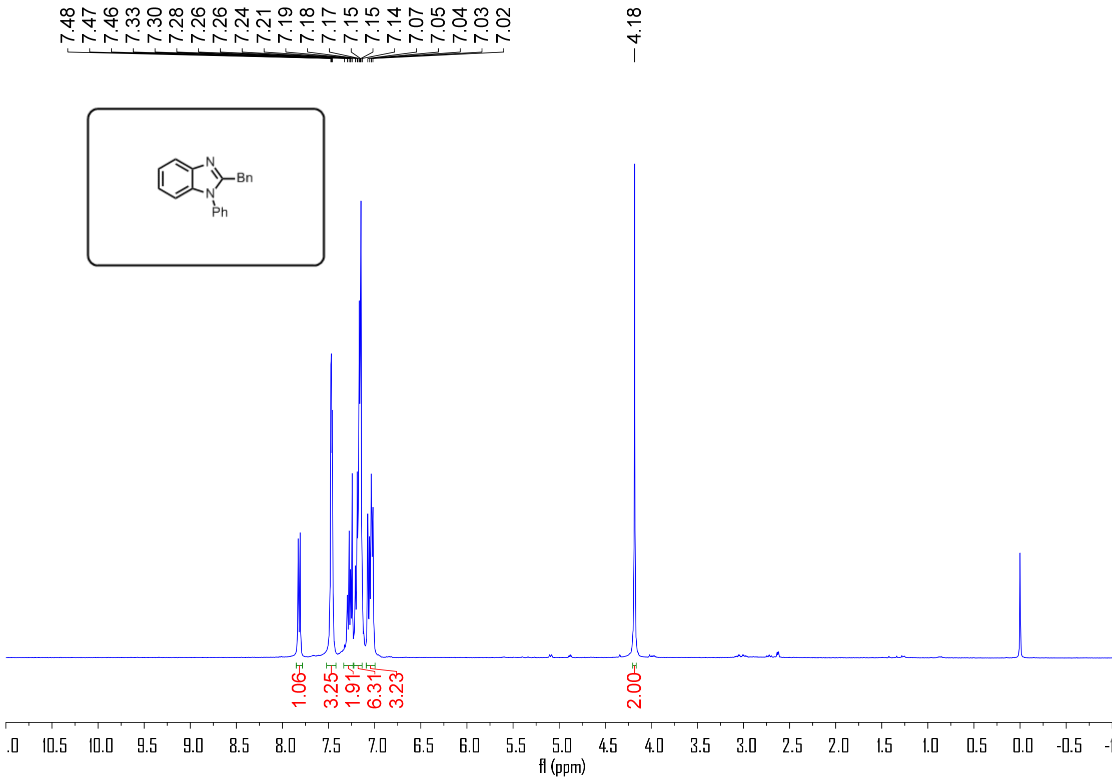

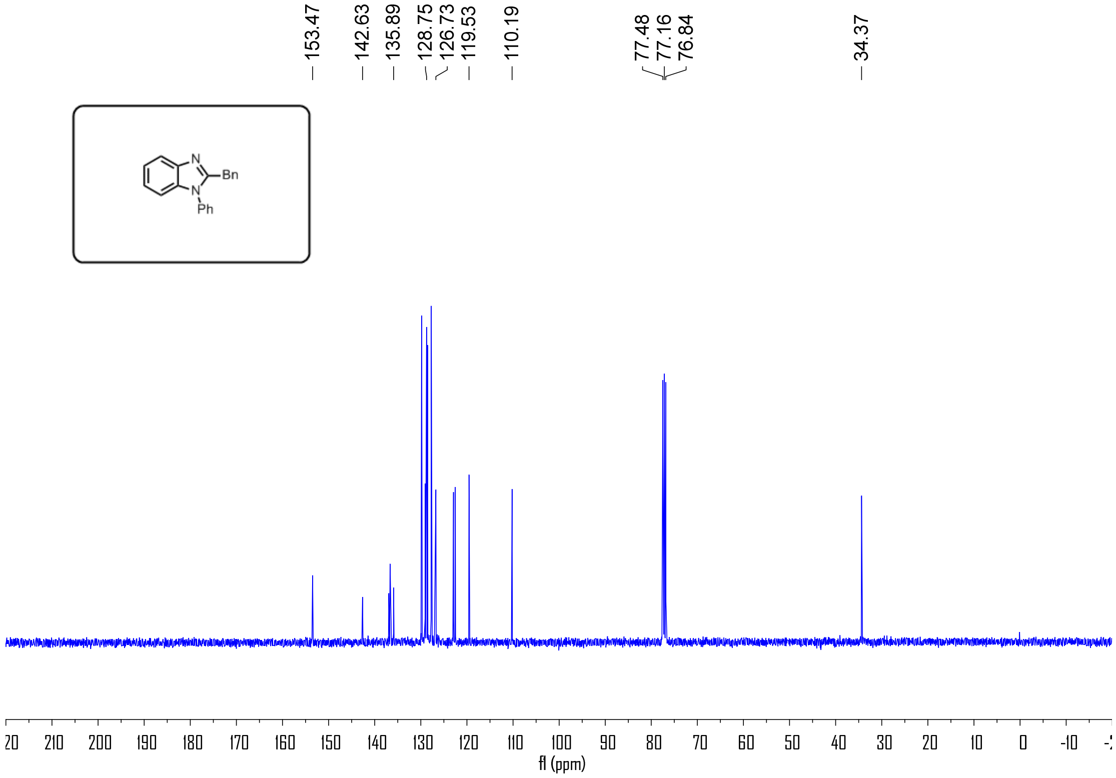

Supplementary Figure 24. ^{1}H and ^{13}C NMR spectra of **1s** in $CDCl_3$.

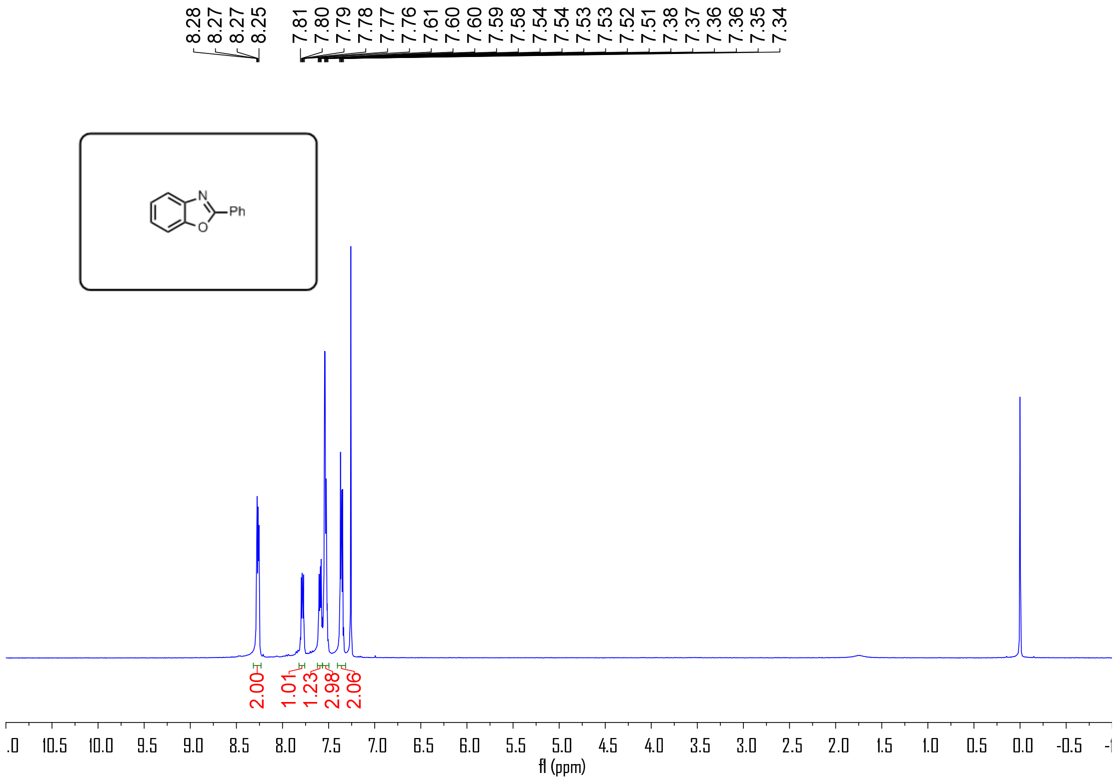

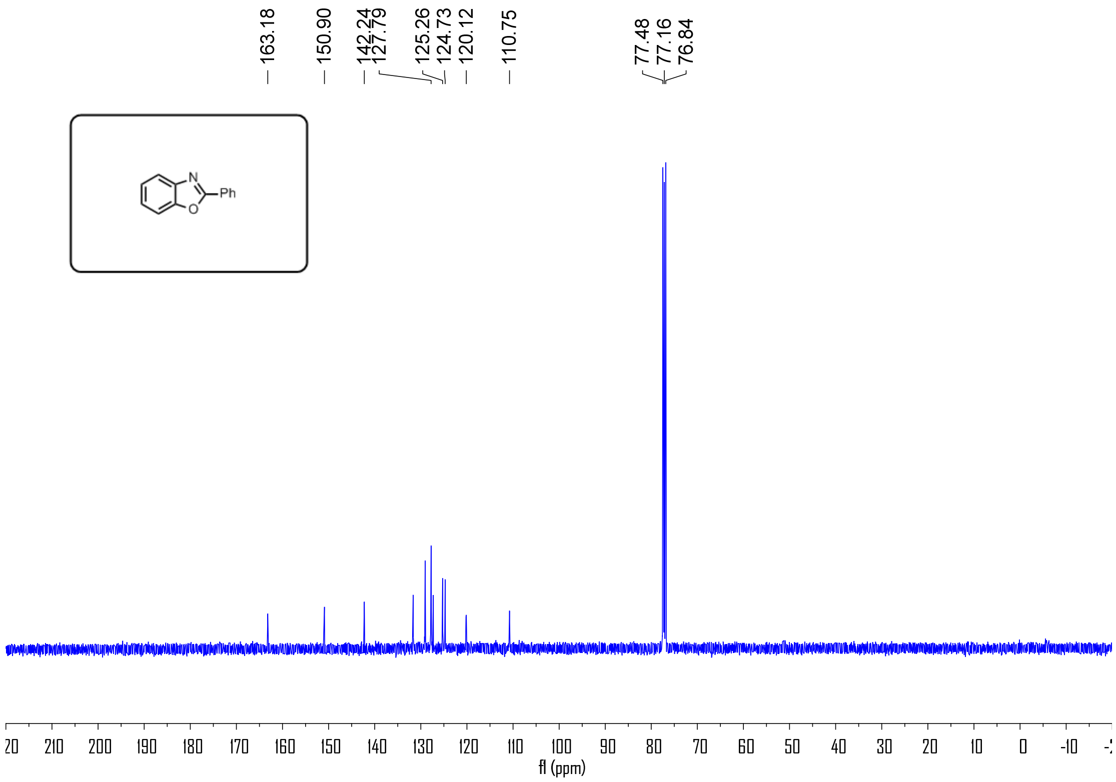

Supplementary Figure 25. ^{1}H and ^{13}C NMR spectra of **1u** in $CDCl_3$.

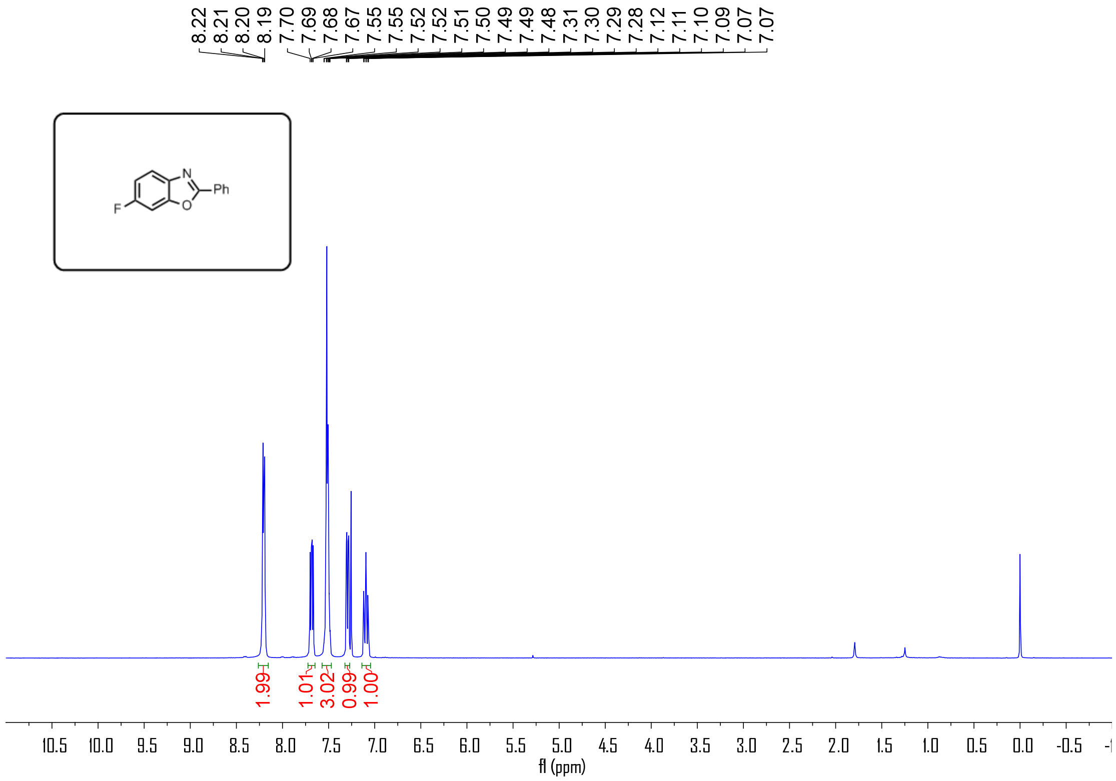

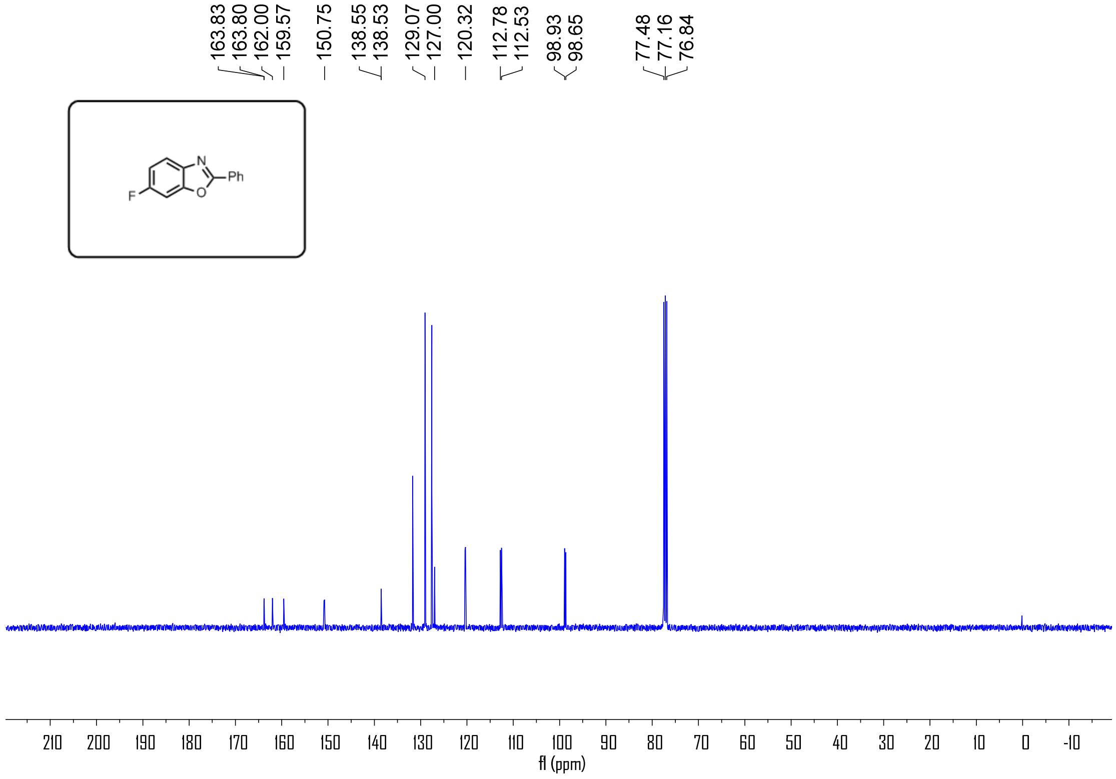

Supplementary Figure 26. ^{1}H and ^{13}C NMR spectra of **1v** in $CDCl_3$.

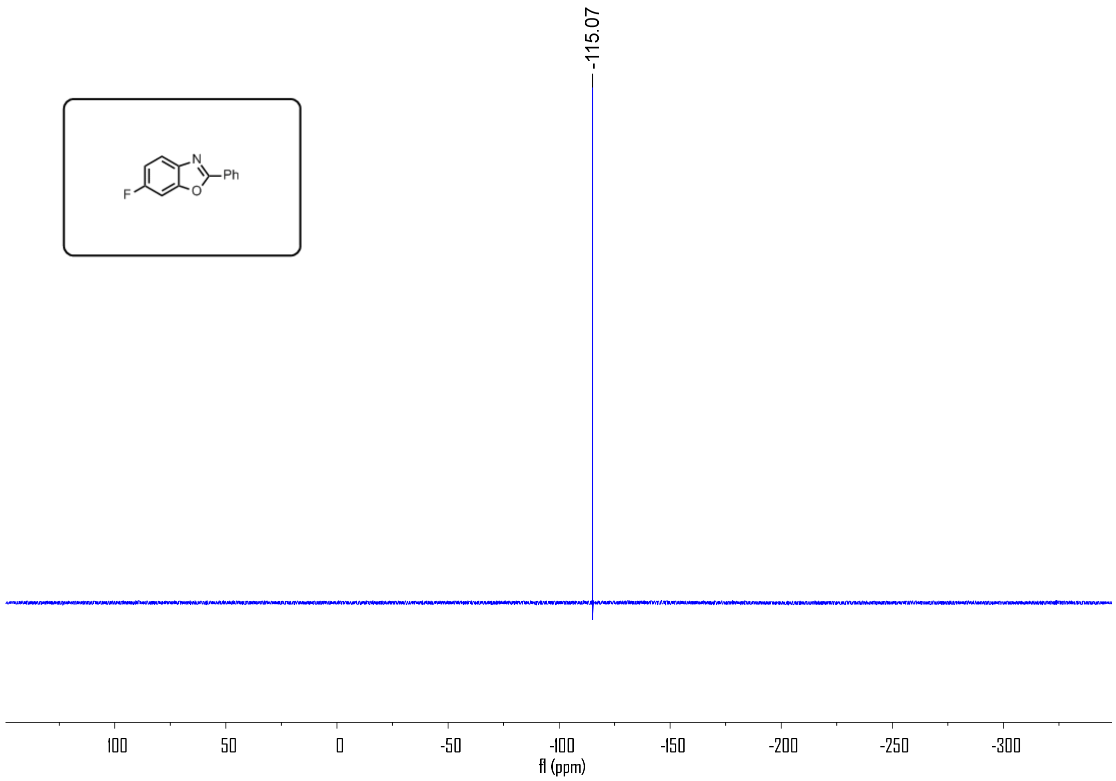

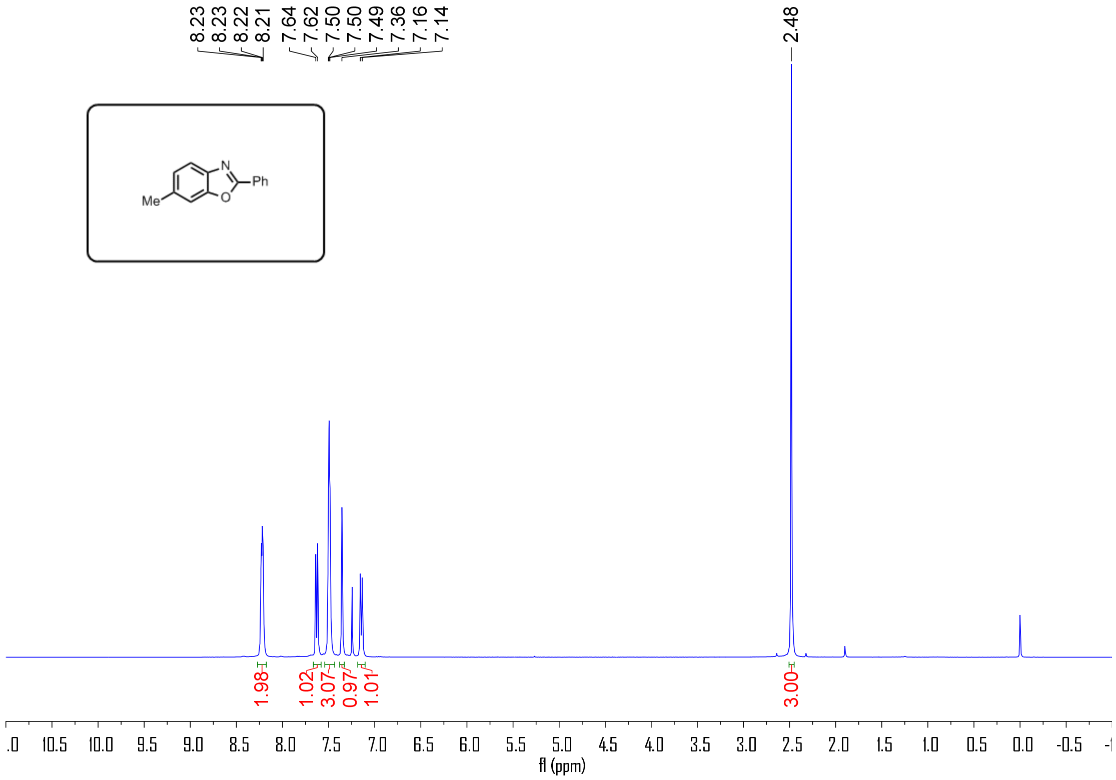

Supplementary Figure 27. ^{19}F (**1v**) and ^{1}H (**1w**) NMR spectra in $CDCl_3$.

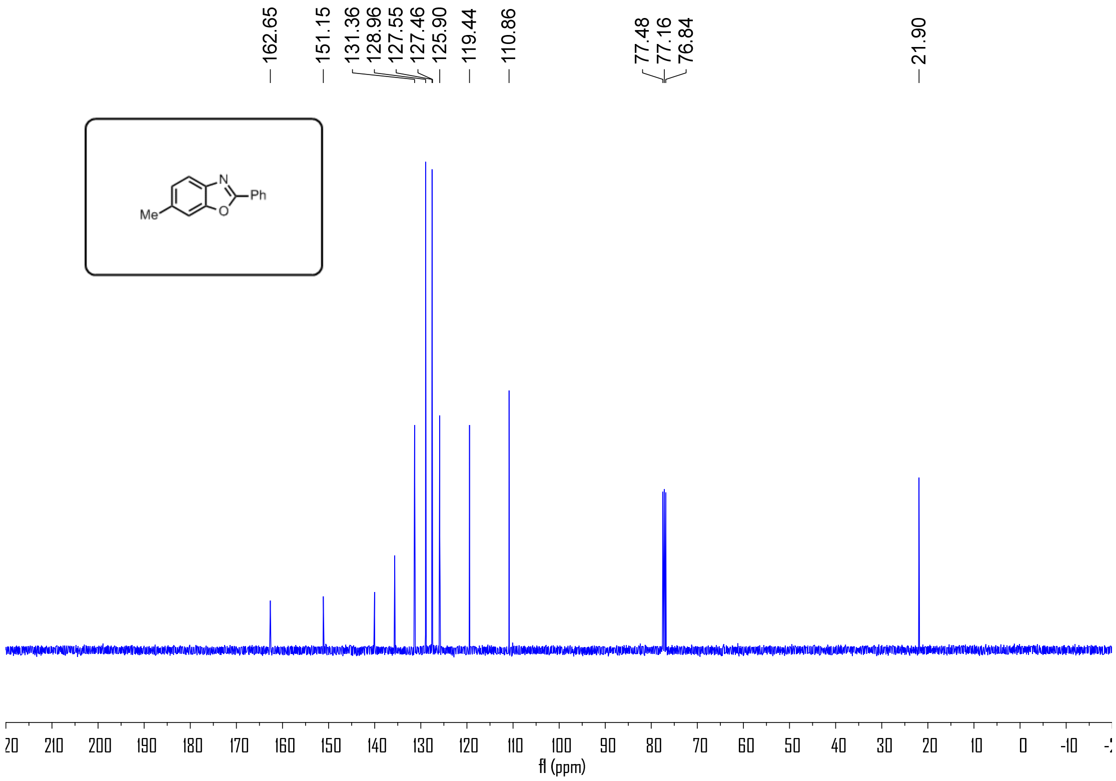

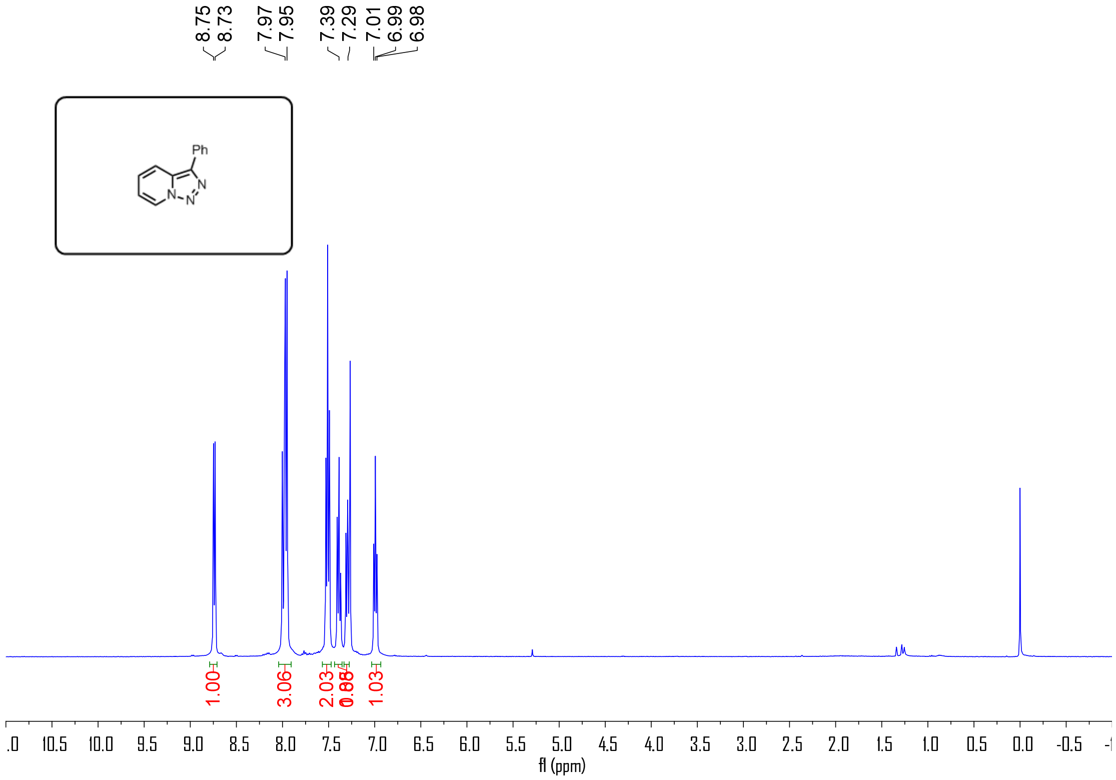

Supplementary Figure 28. ^{13}C (**1w**) and ^{1}H (**1x**) NMR spectra in $CDCl_3$.

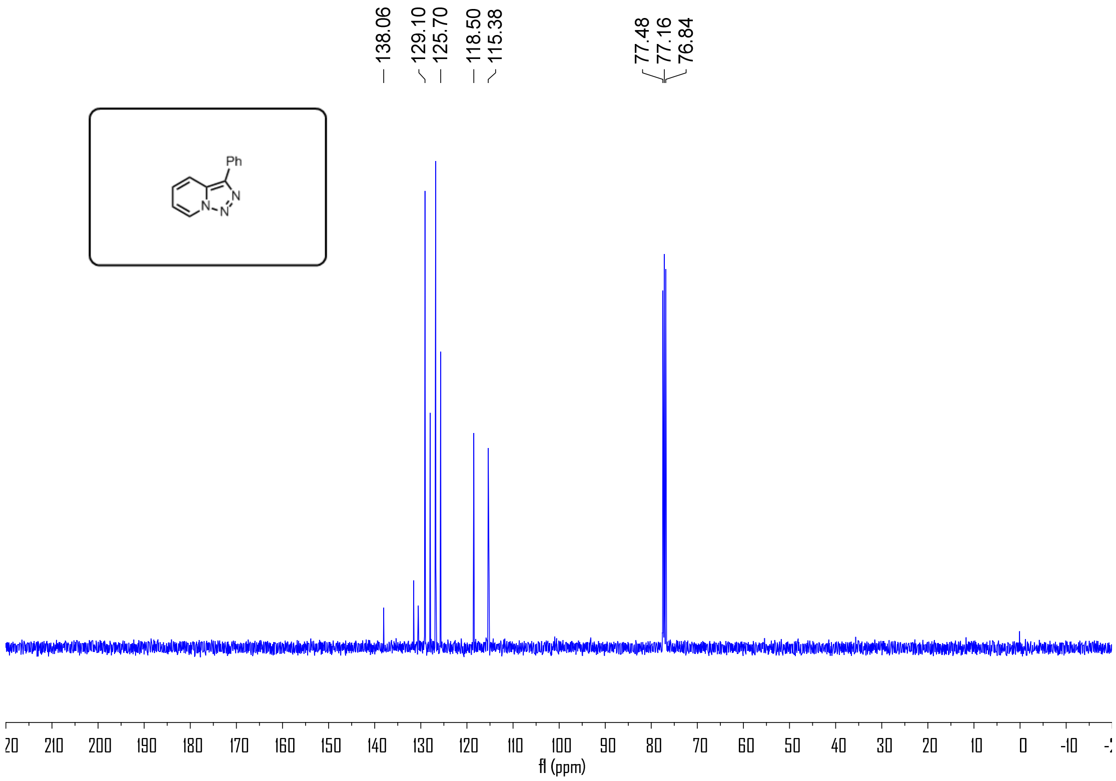

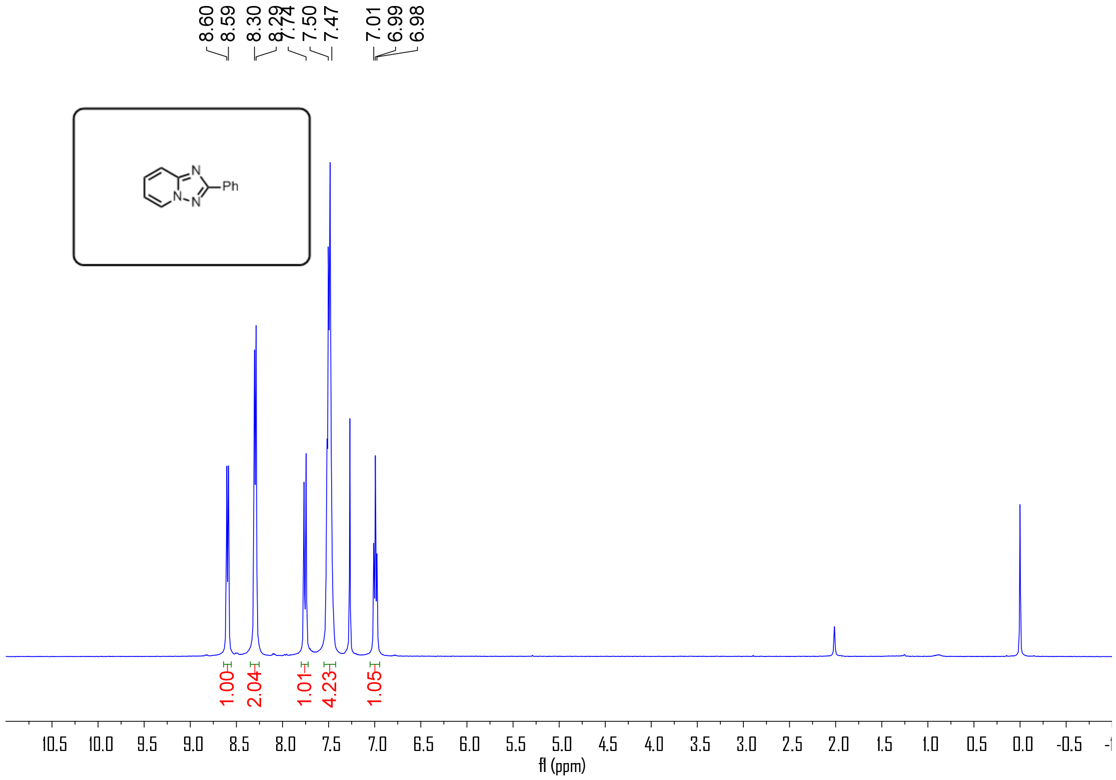

Supplementary Figure 29. ^{13}C (**1x**) and ^{1}H (**1y**) NMR spectra in $CDCl_3$.

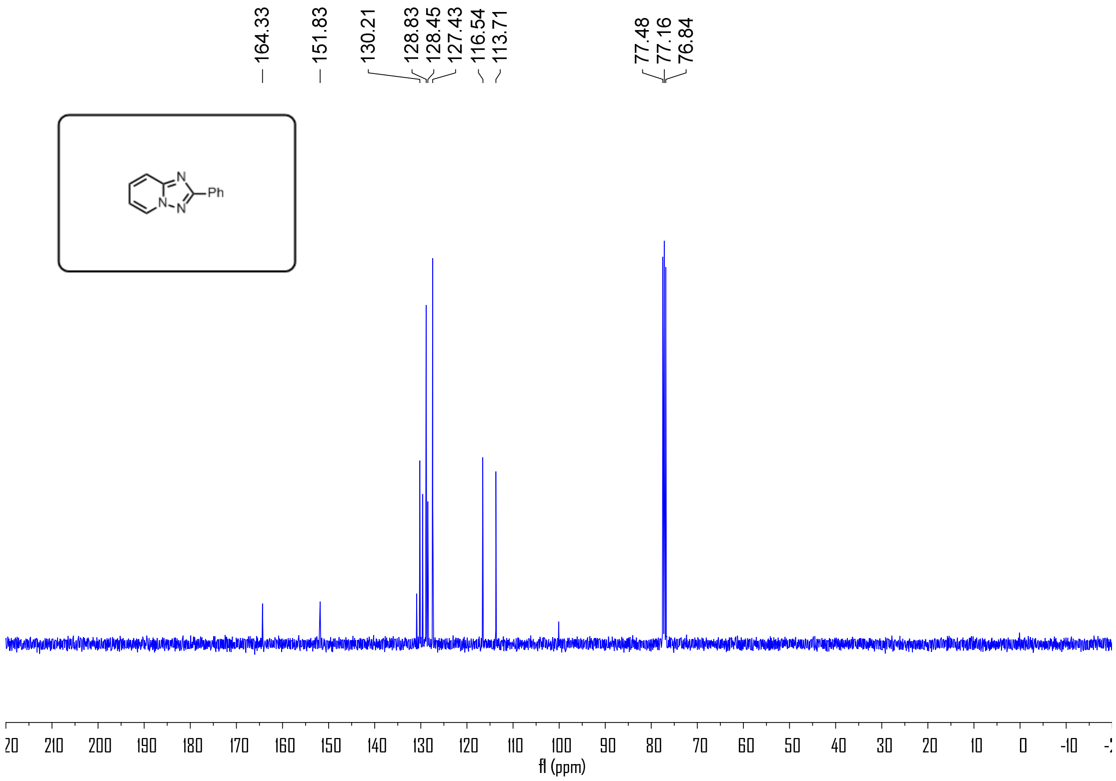

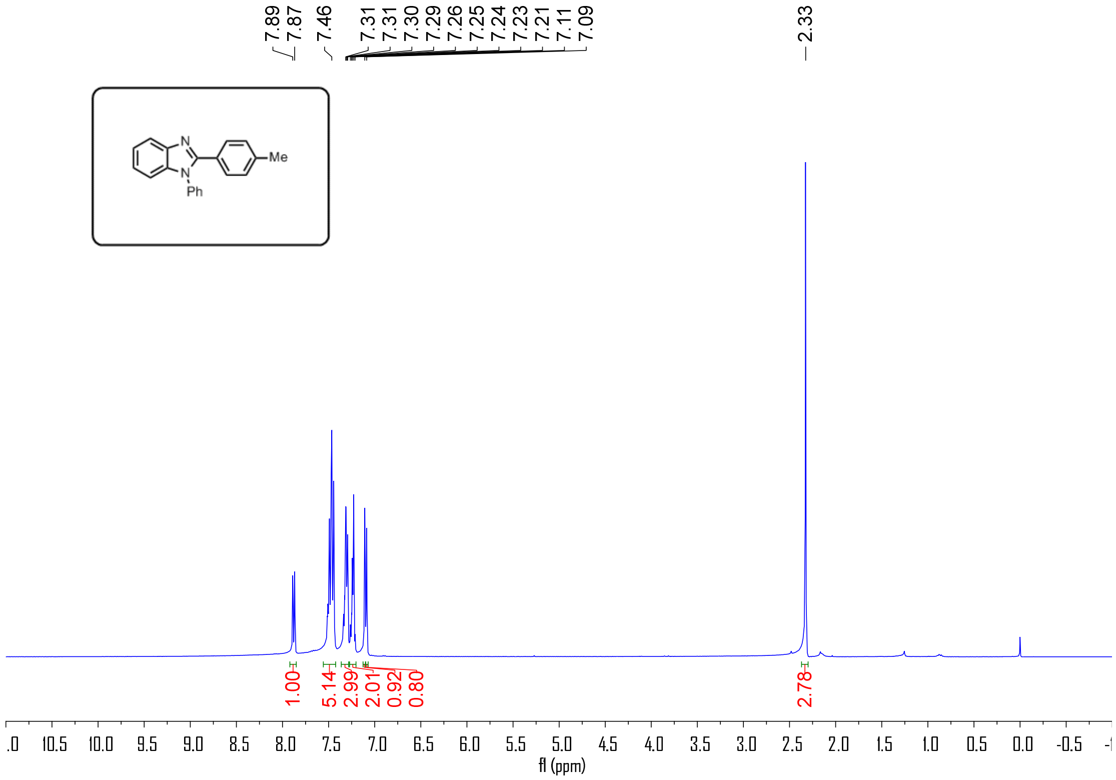

Supplementary Figure 30. ^{13}C (**1y**) and ^{1}H (**1aa**) NMR spectra in $CDCl_3$.

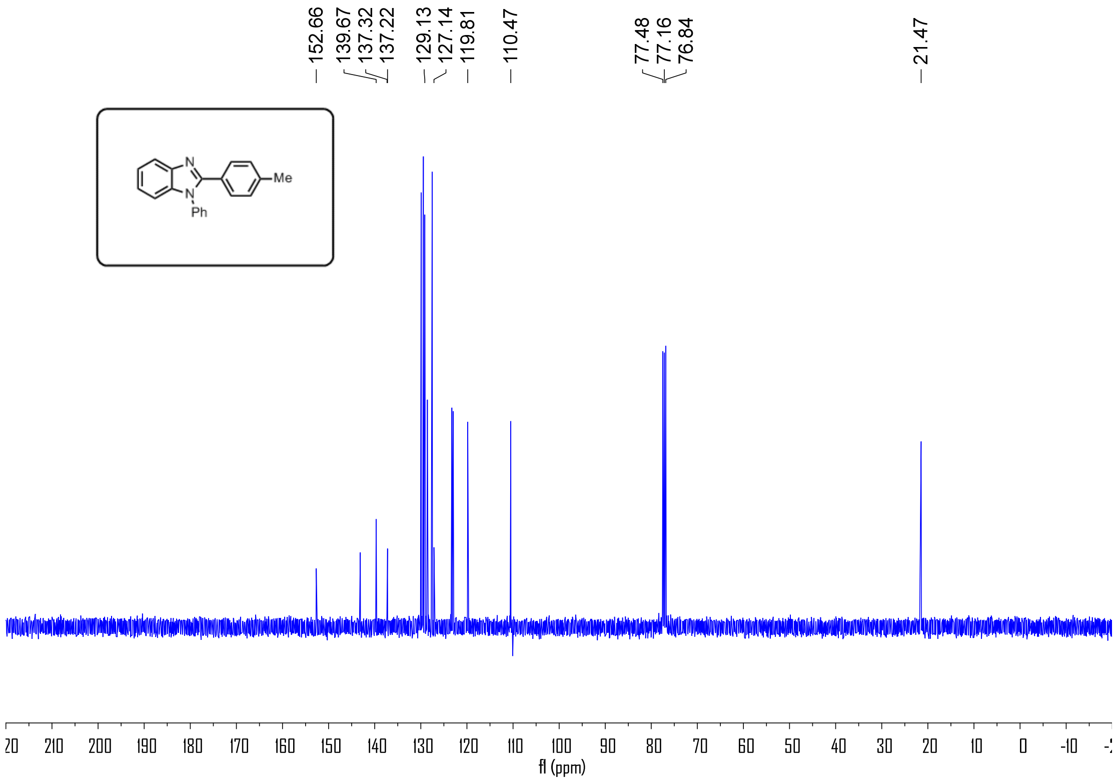

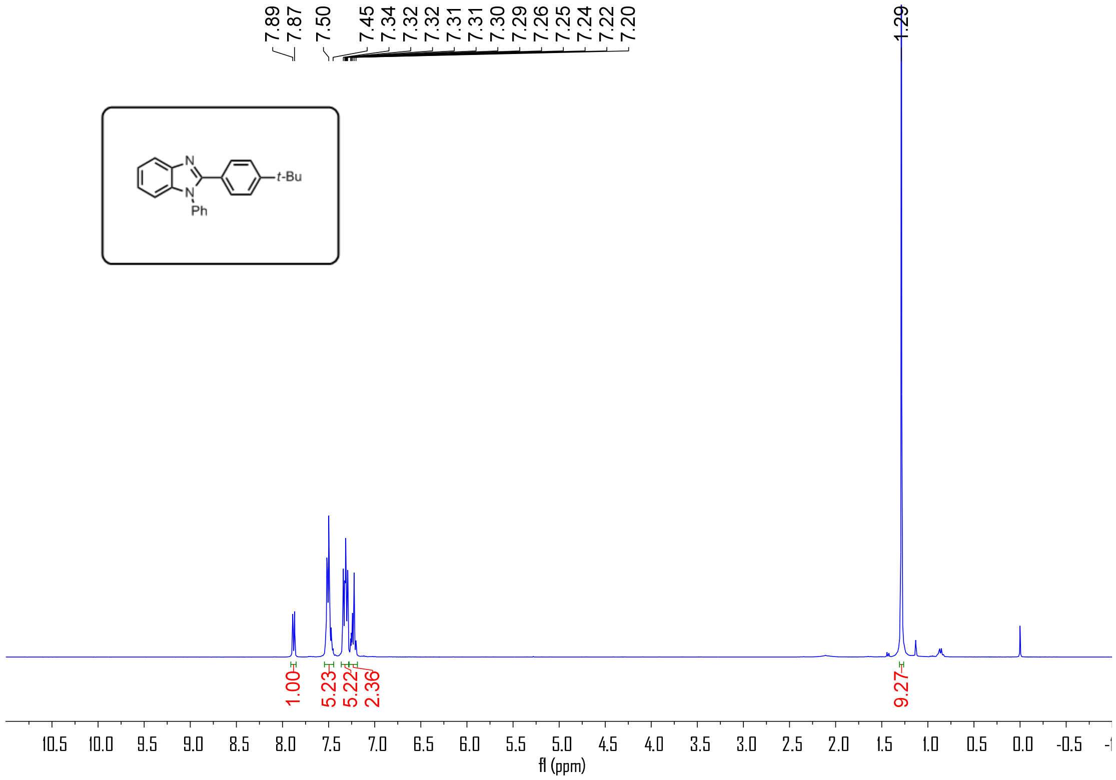

Supplementary Figure 31. ^{13}C (**1aa**) and ^{1}H (**1ab**) NMR spectra in $CDCl_3$.

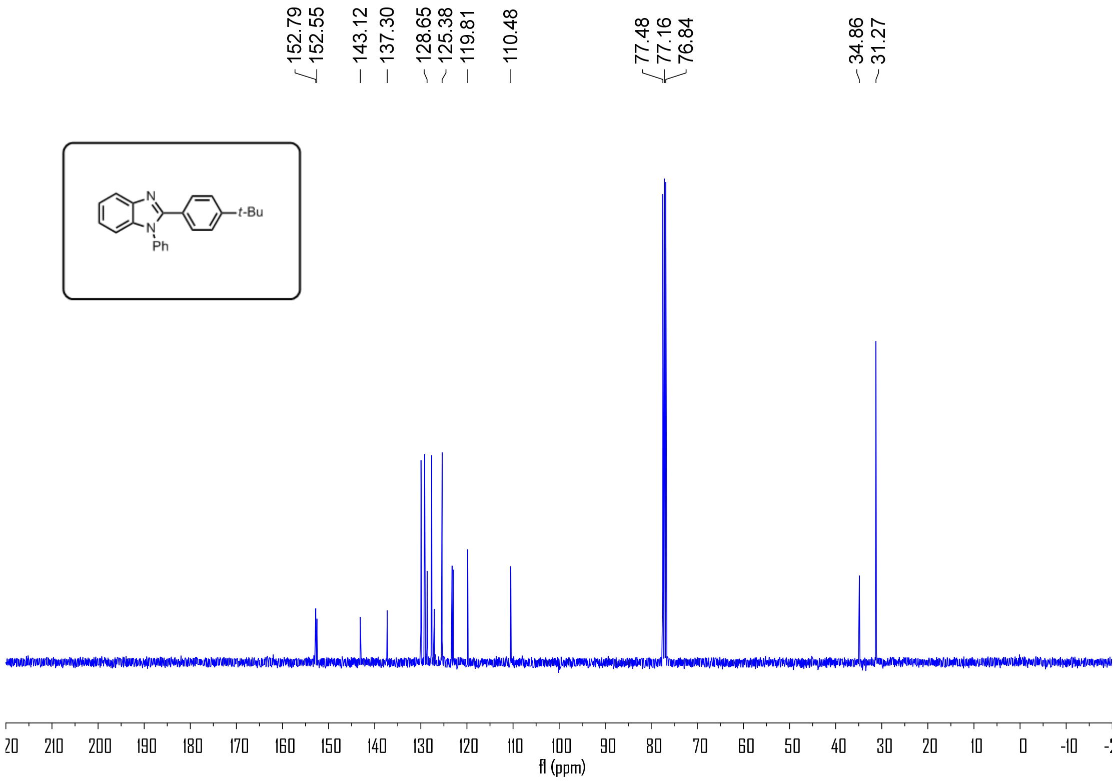

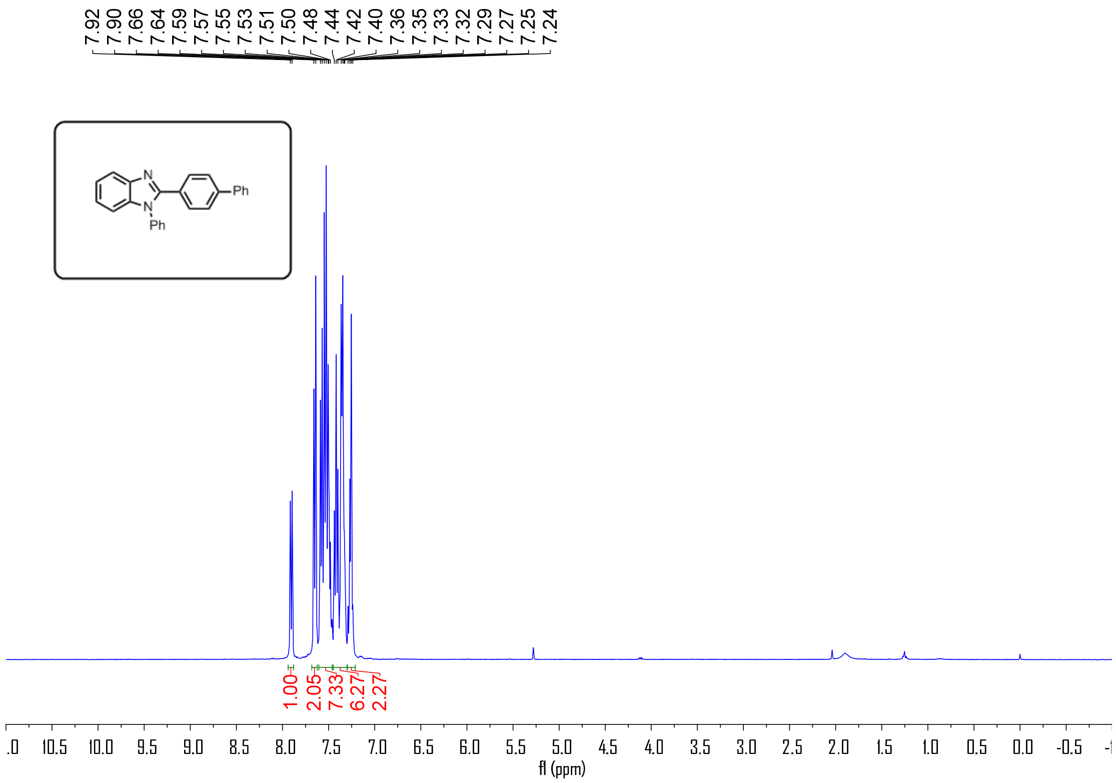

Supplementary Figure 32. ^{13}C (**1ab**) and ^{1}H (**1ac**) NMR spectra in $CDCl_3$.

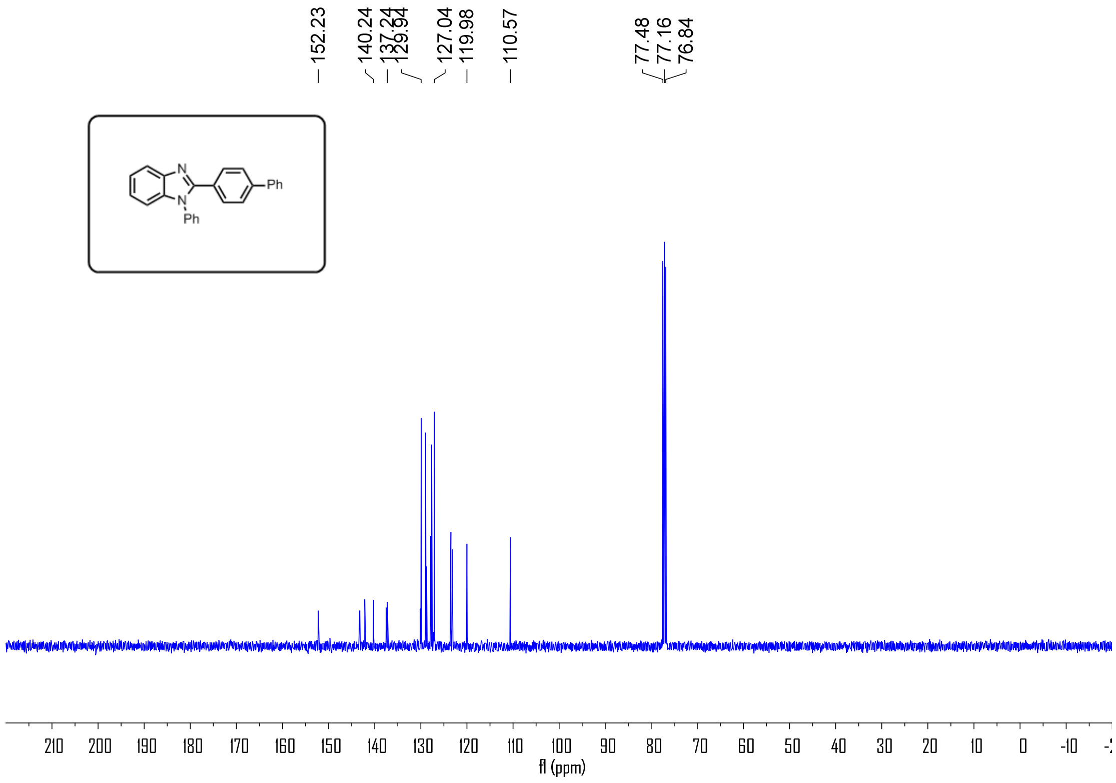

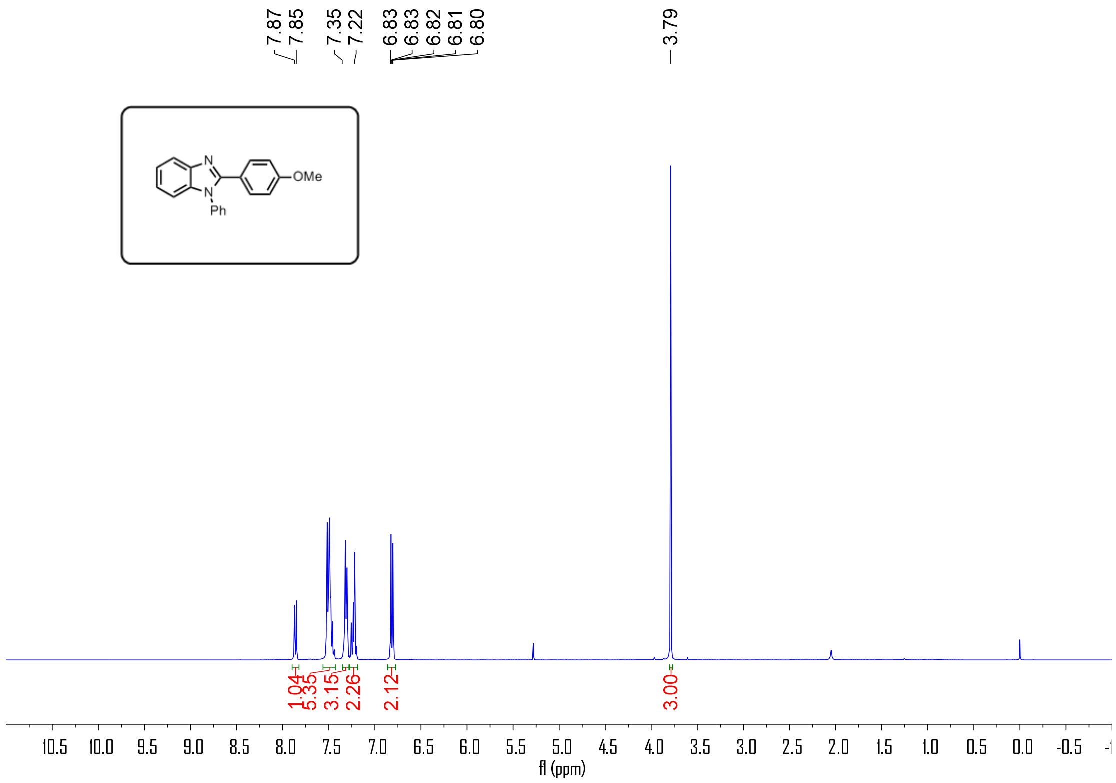

Supplementary Figure 33. ^{13}C (**1ac**) and ^{1}H (**1ad**) NMR spectra in $CDCl_3$.

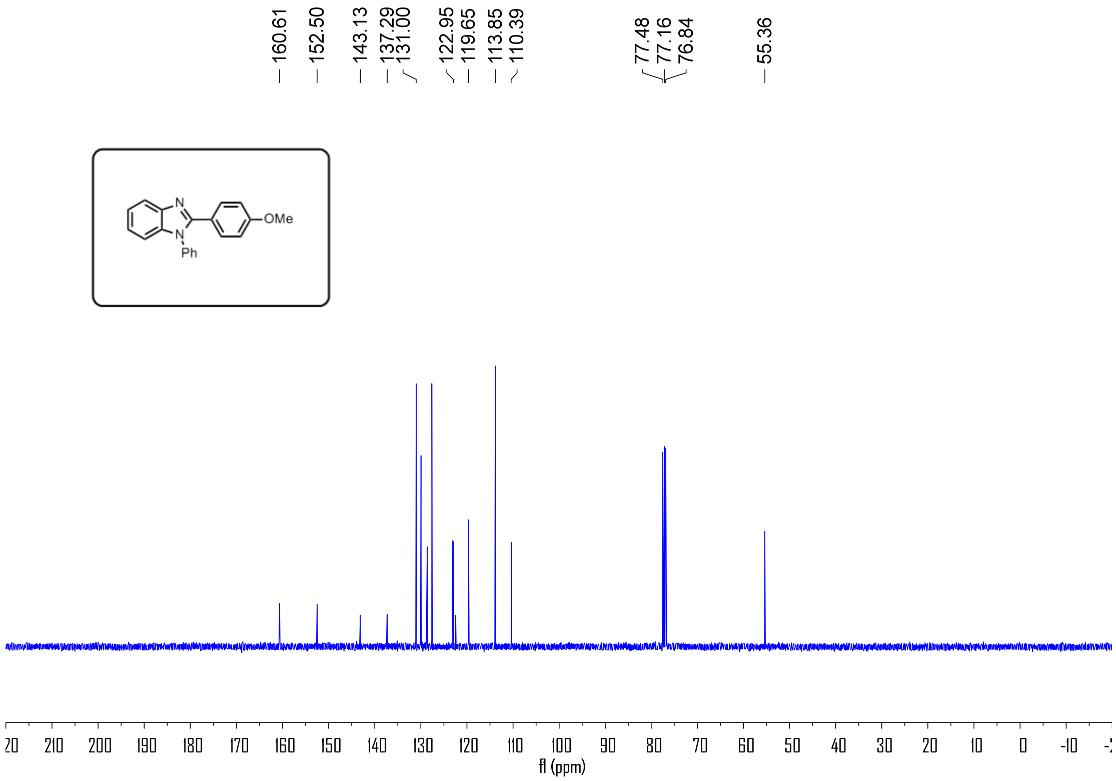

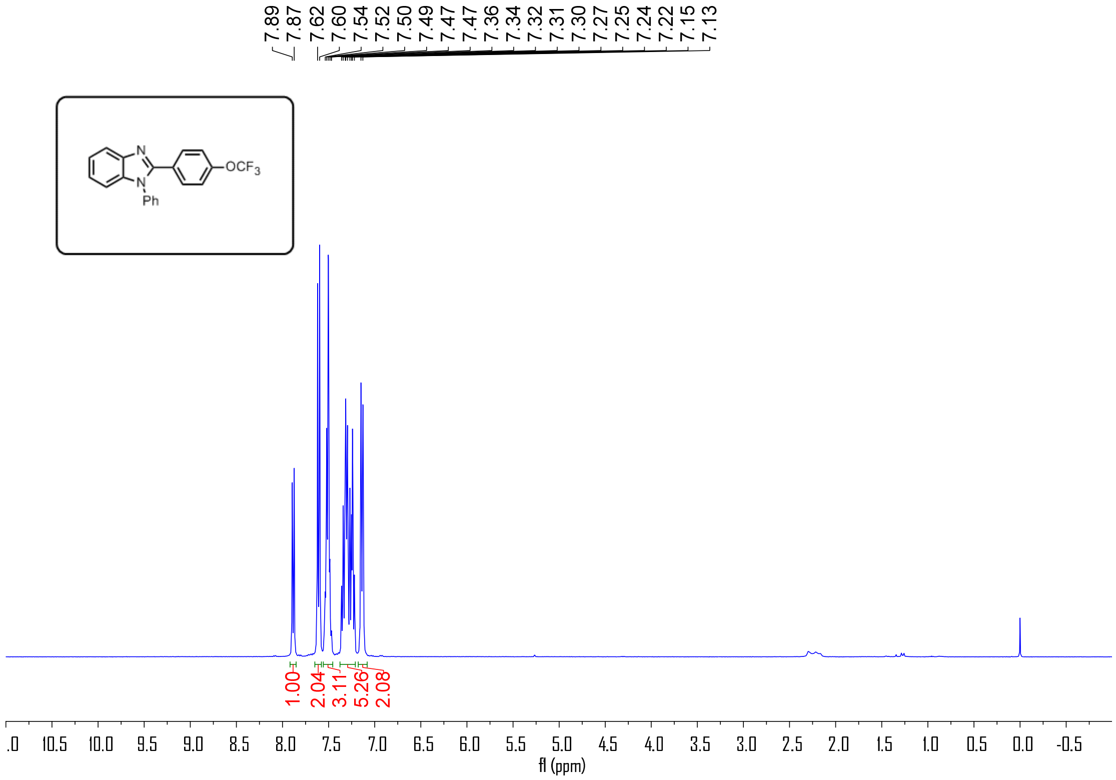

Supplementary Figure 34. ^{13}C (**1ad**) and ^{1}H (**1ae**) NMR spectra in $CDCl_3$.

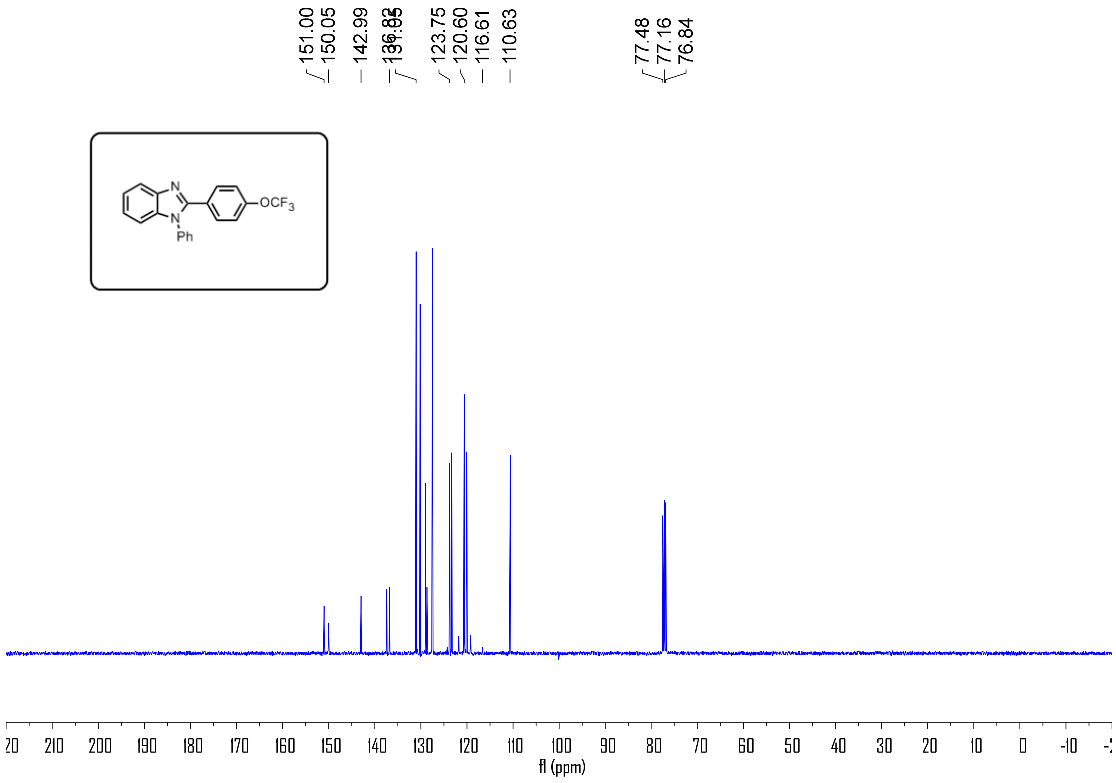

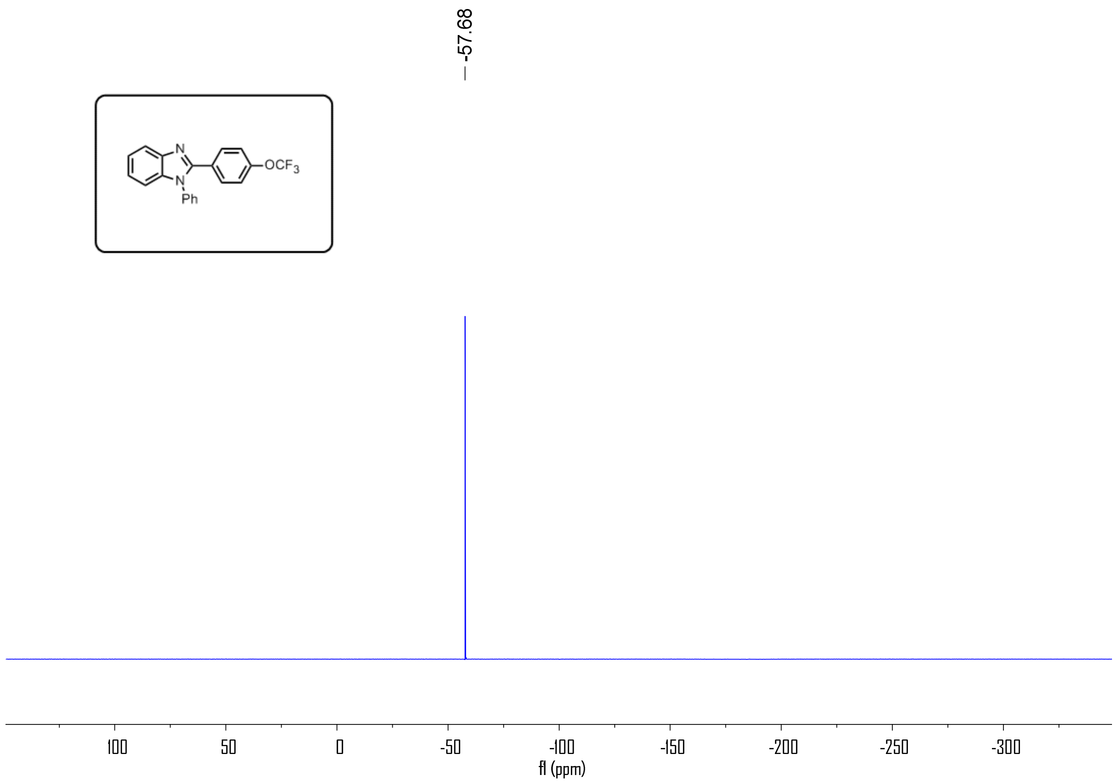

Supplementary Figure 35. ^{13}C and ^{19}F NMR spectra of **1ae** in $CDCl_3$.

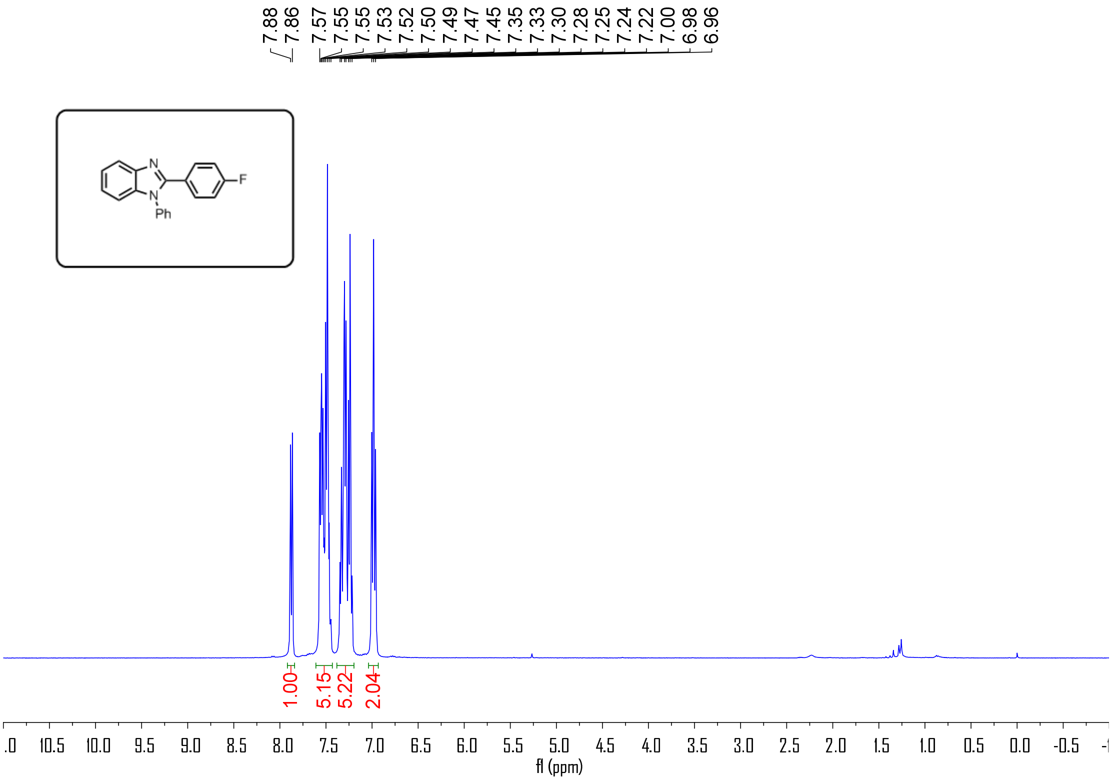

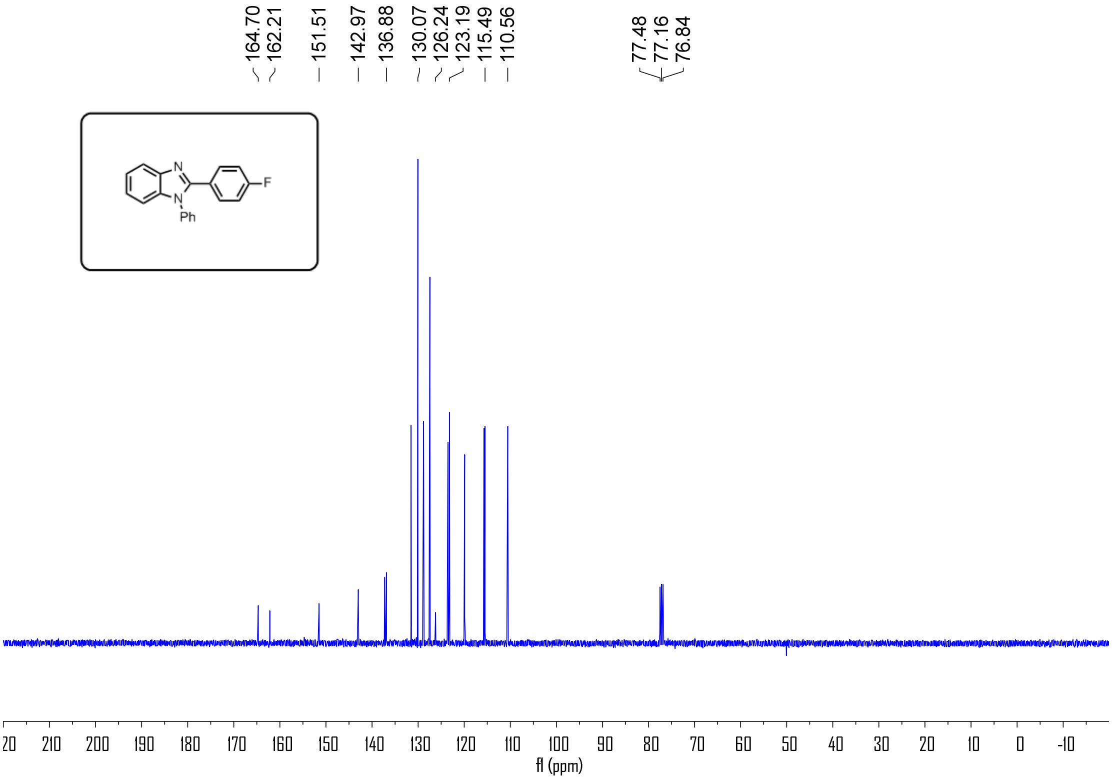

Supplementary Figure 36. ^{1}H and ^{13}C NMR spectra of **1af** in $CDCl_3$.

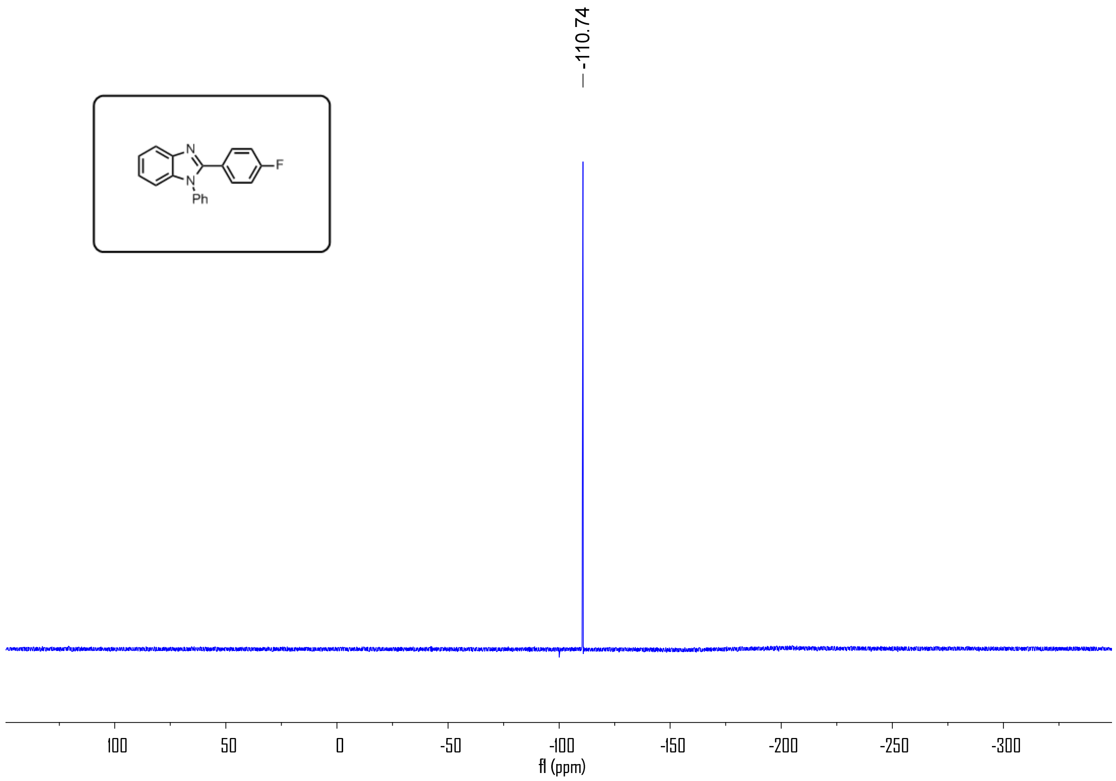

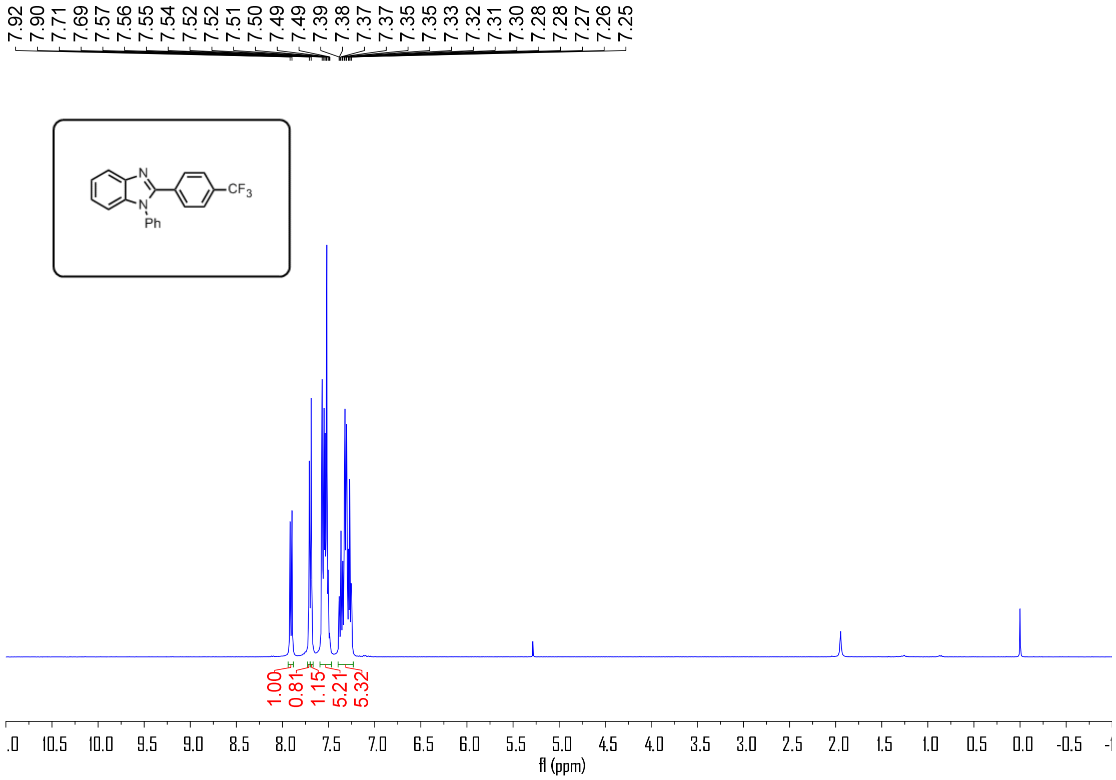

Supplementary Figure 37. ^{19}F (**1af**) and ^{1}H (**1ag**) NMR spectra in $CDCl_3$.

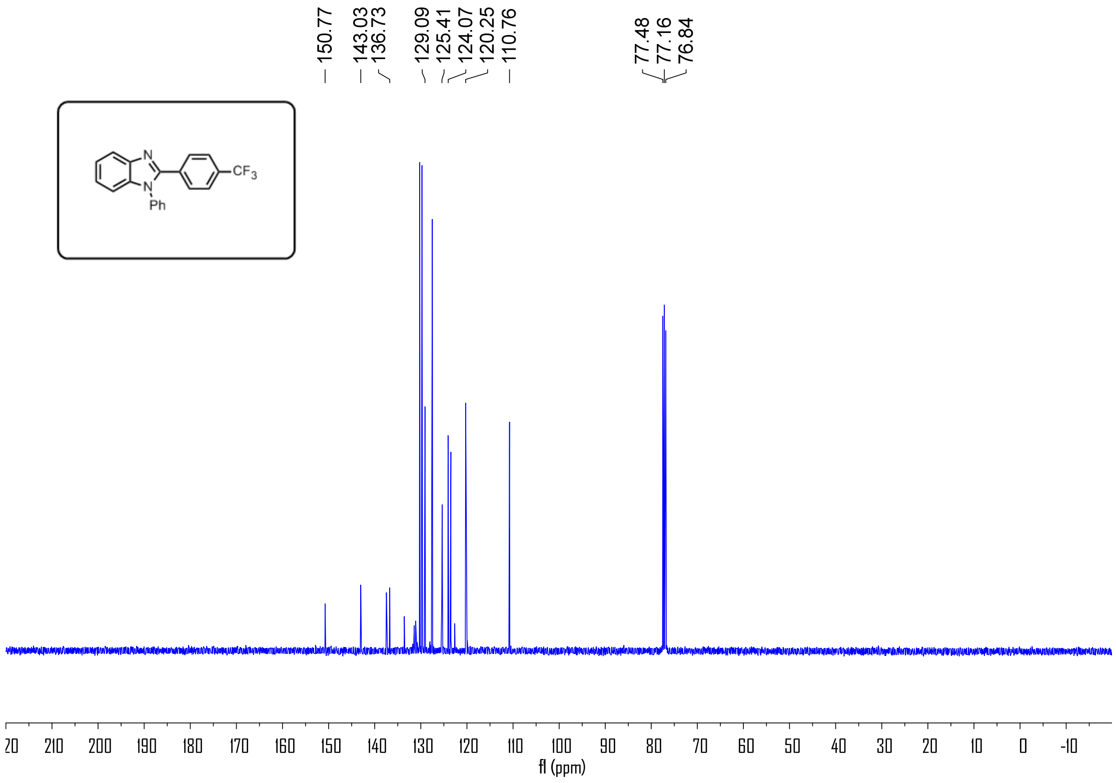

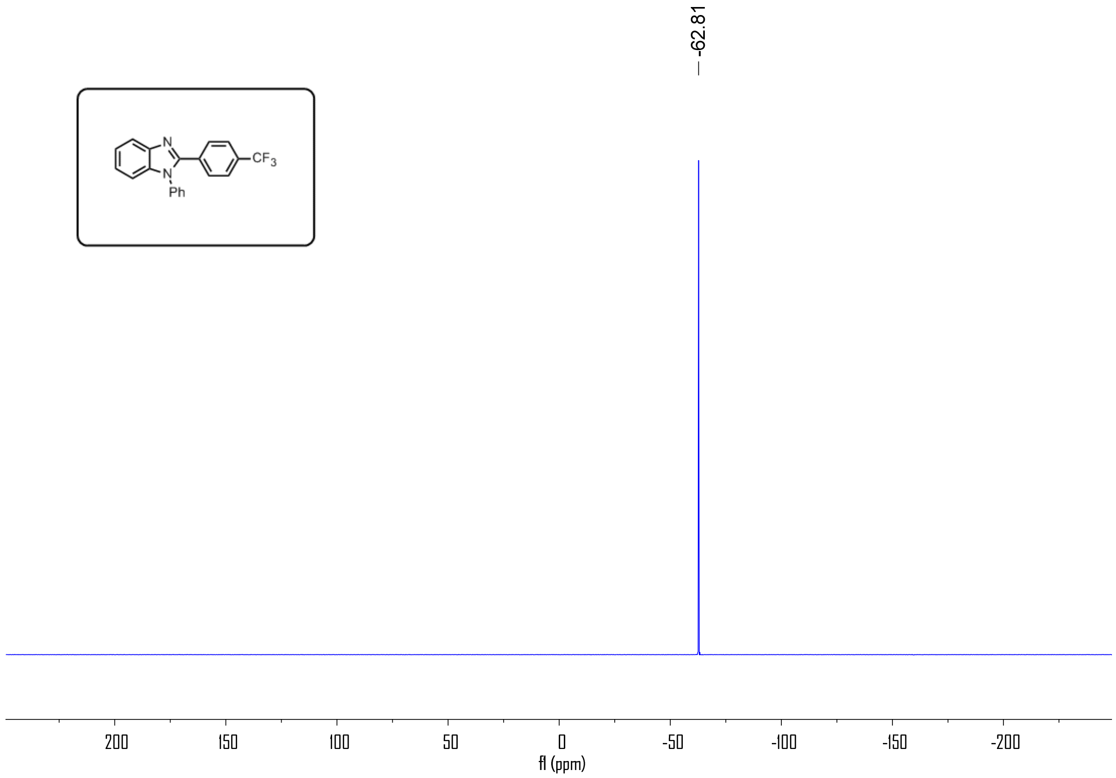

Supplementary Figure 38. ^{13}C and ^{19}F NMR spectra of **1ag** in $CDCl_3$.

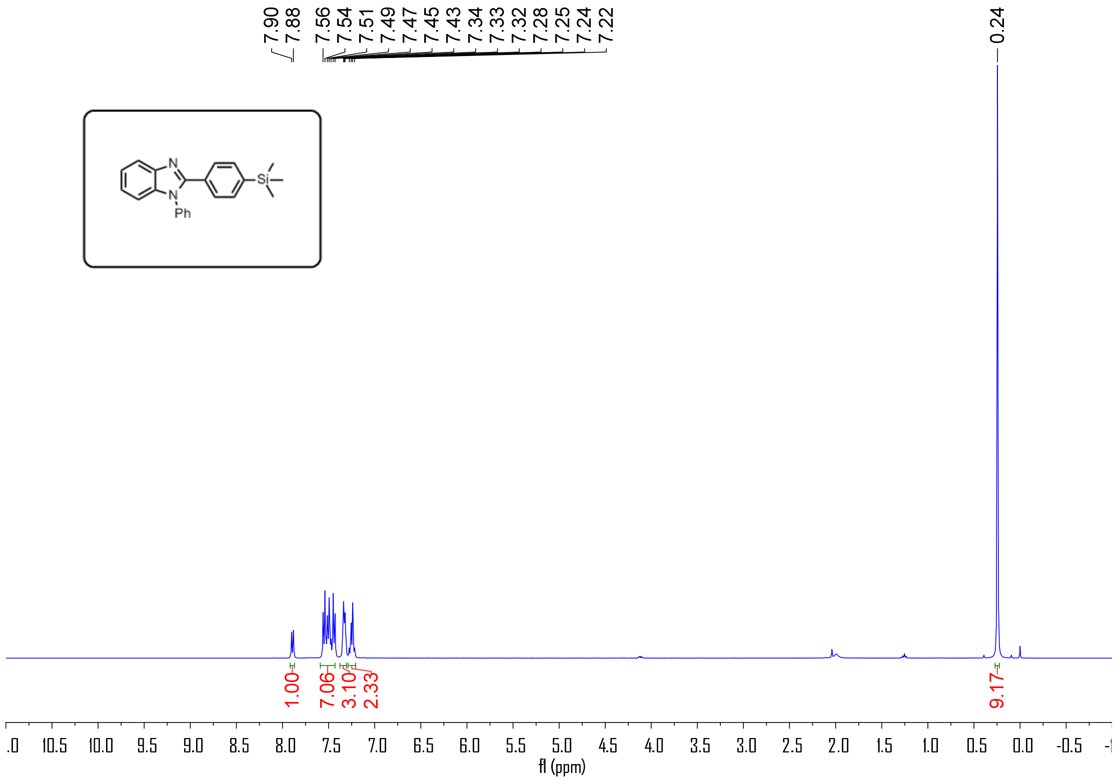

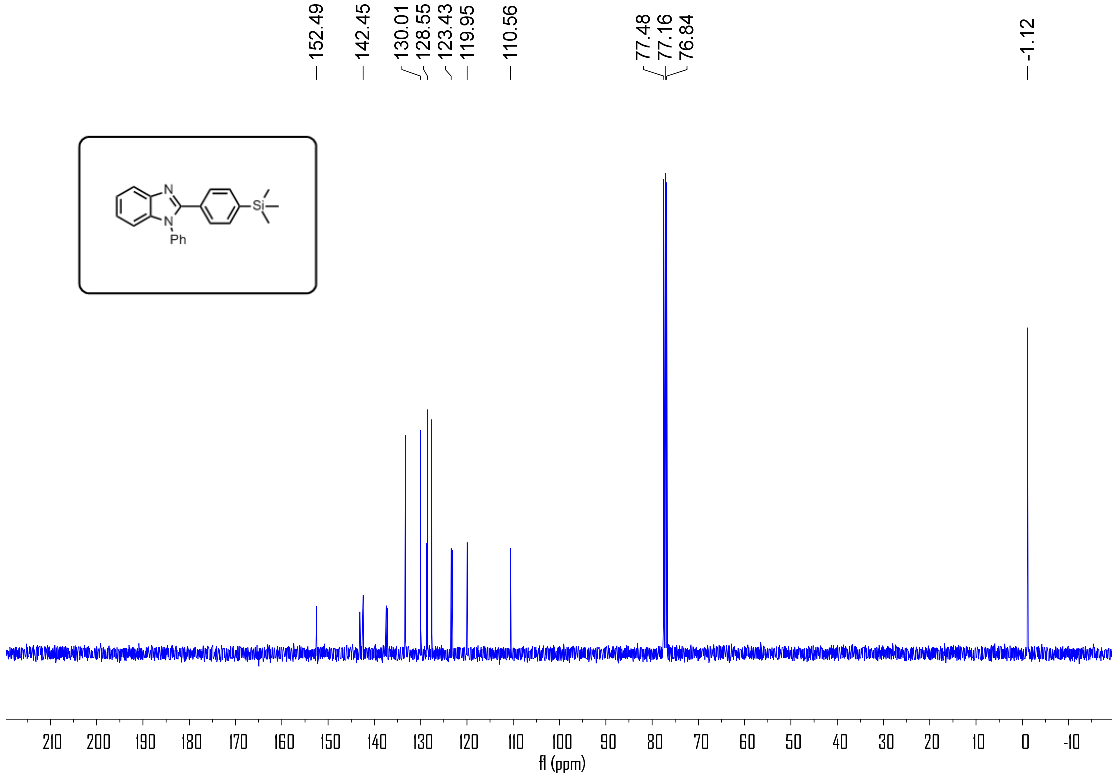

Supplementary Figure 39. ^{1}H and ^{13}C NMR spectra of **1ah** in $CDCl_3$.

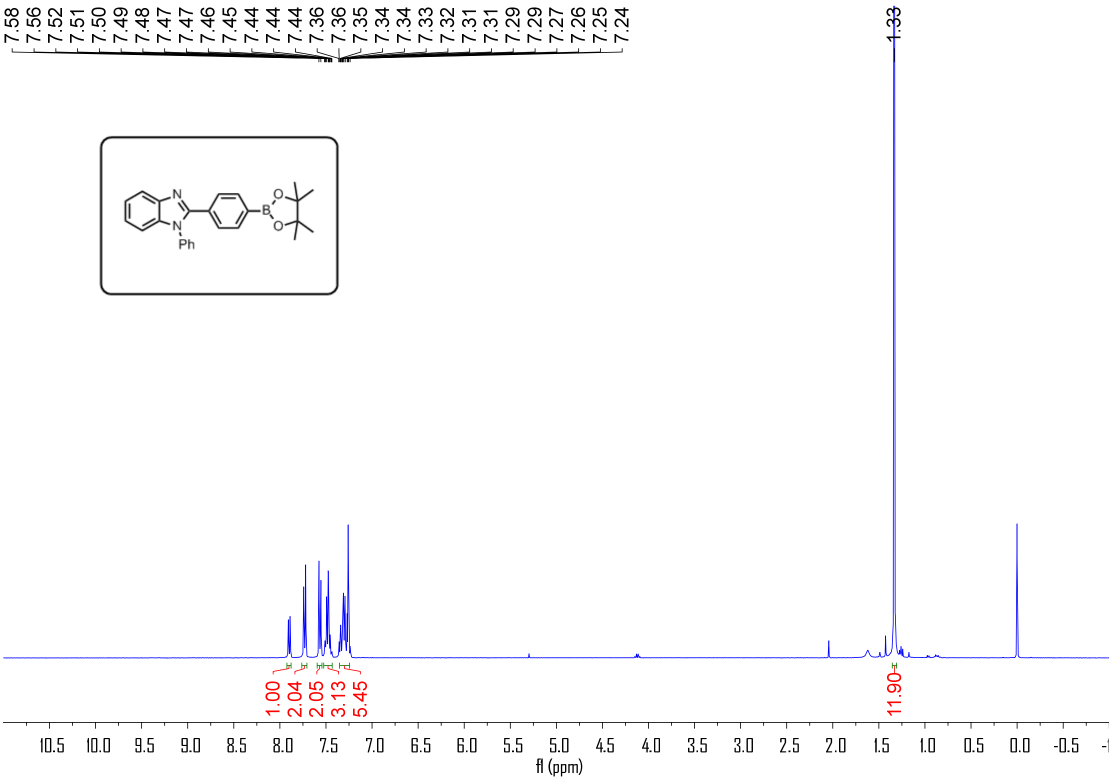

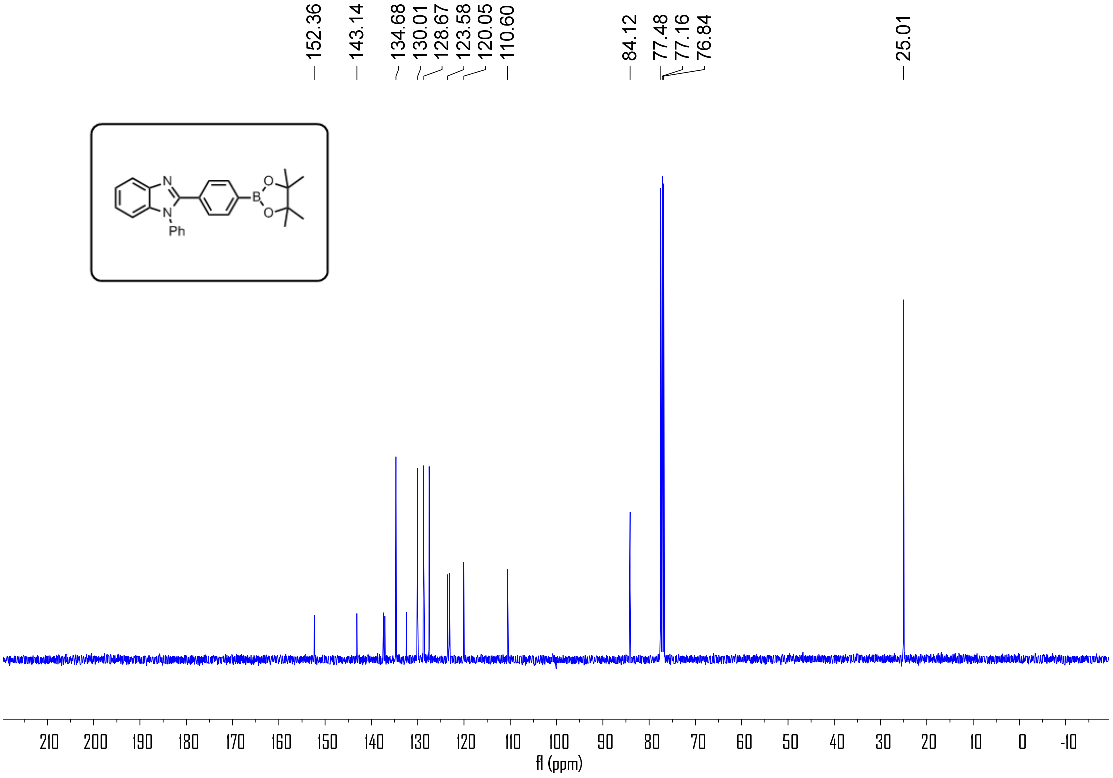

Supplementary Figure 40. ^{1}H and ^{13}C NMR spectra of **1ai** in $CDCl_3$.

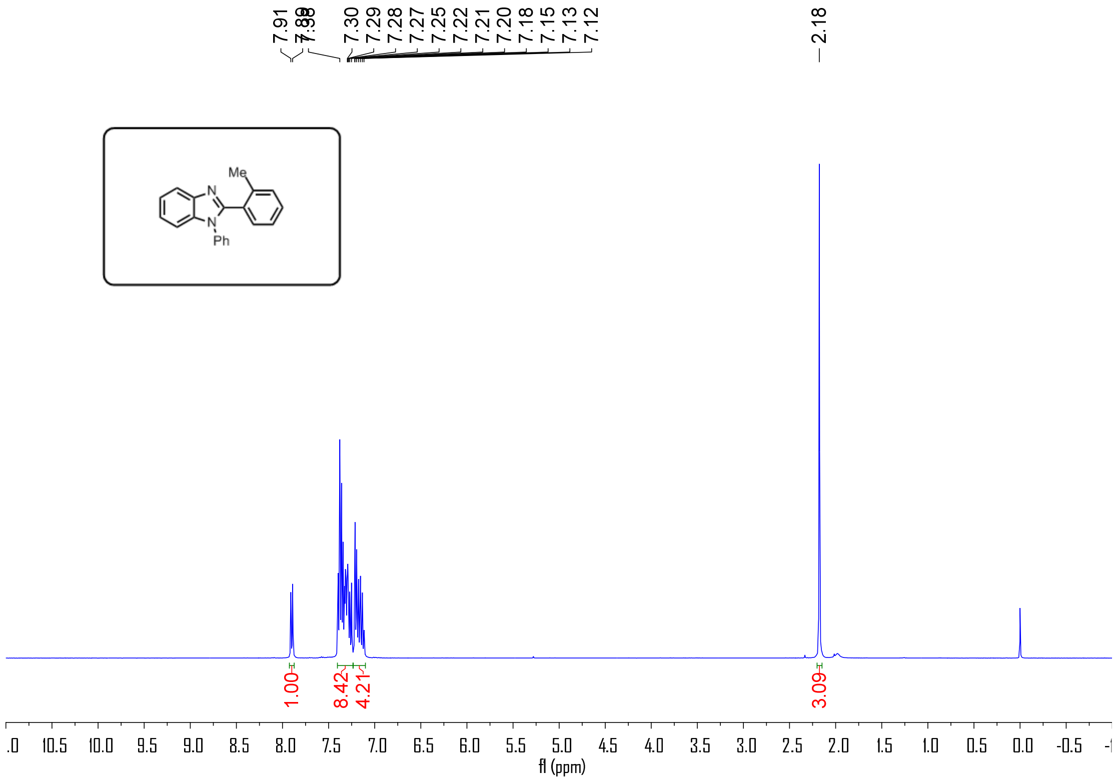

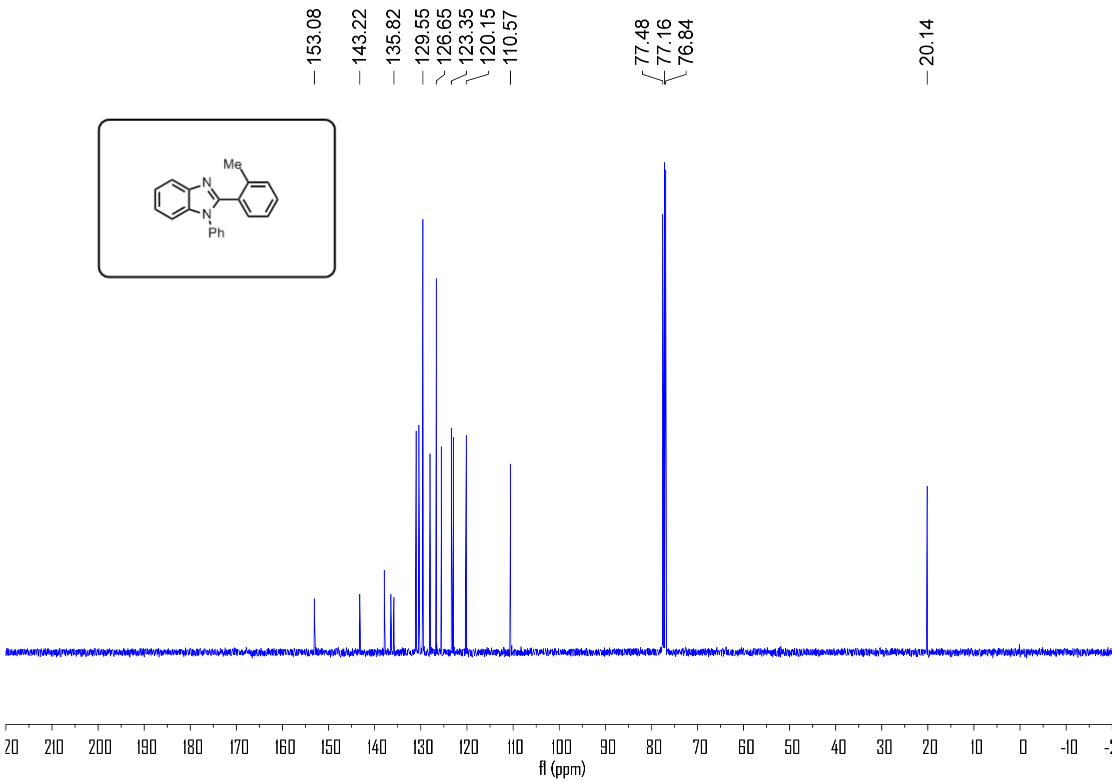

Supplementary Figure 41. ^{1}H and ^{13}C NMR spectra of **1aj** in $CDCl_3$.

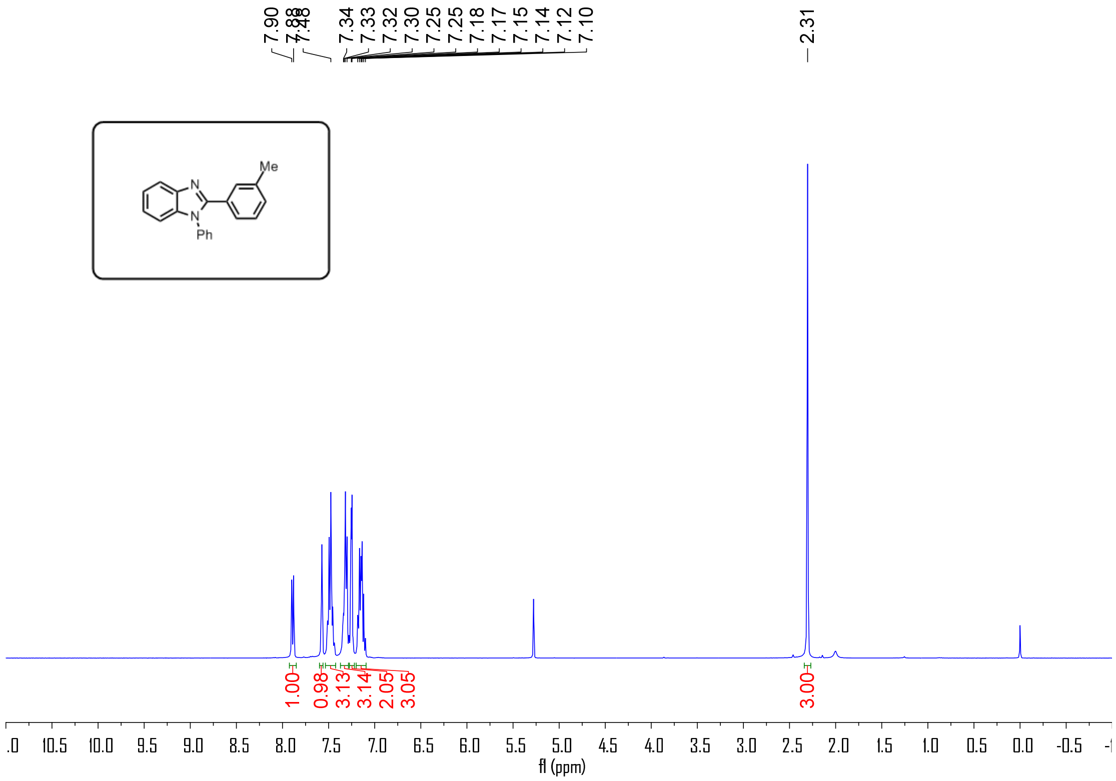

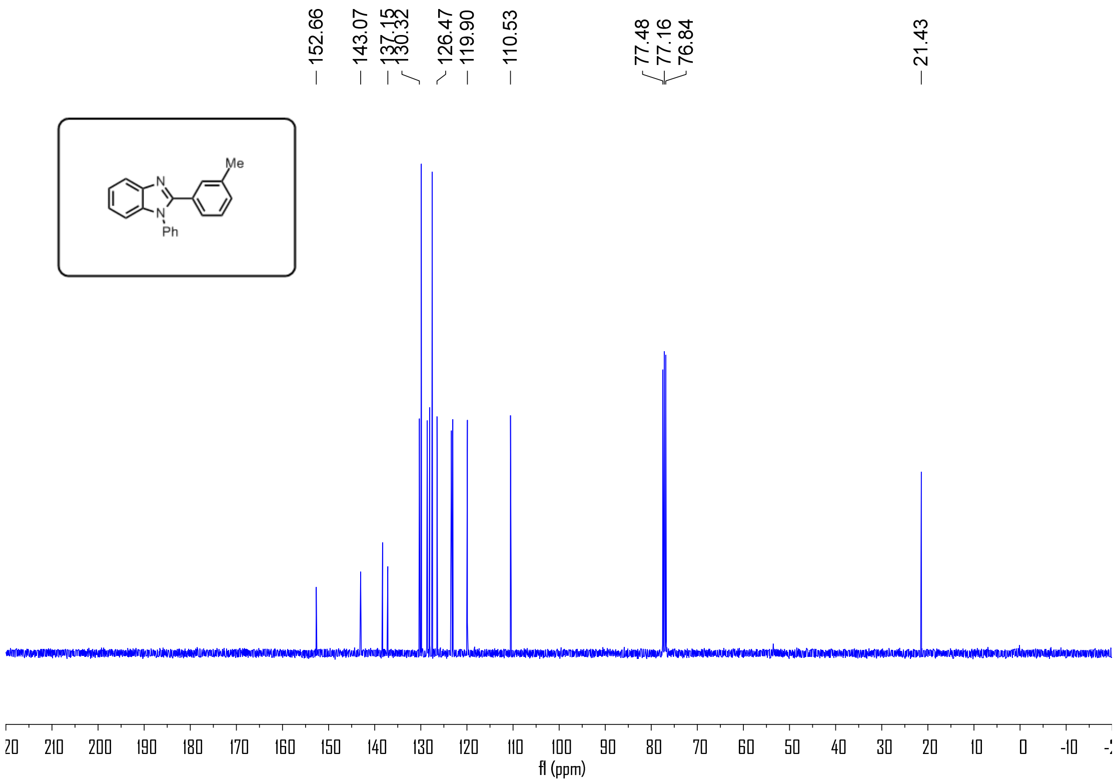

Supplementary Figure 42. ^{1}H and ^{13}C NMR spectra of **1ak** in $CDCl_3$.

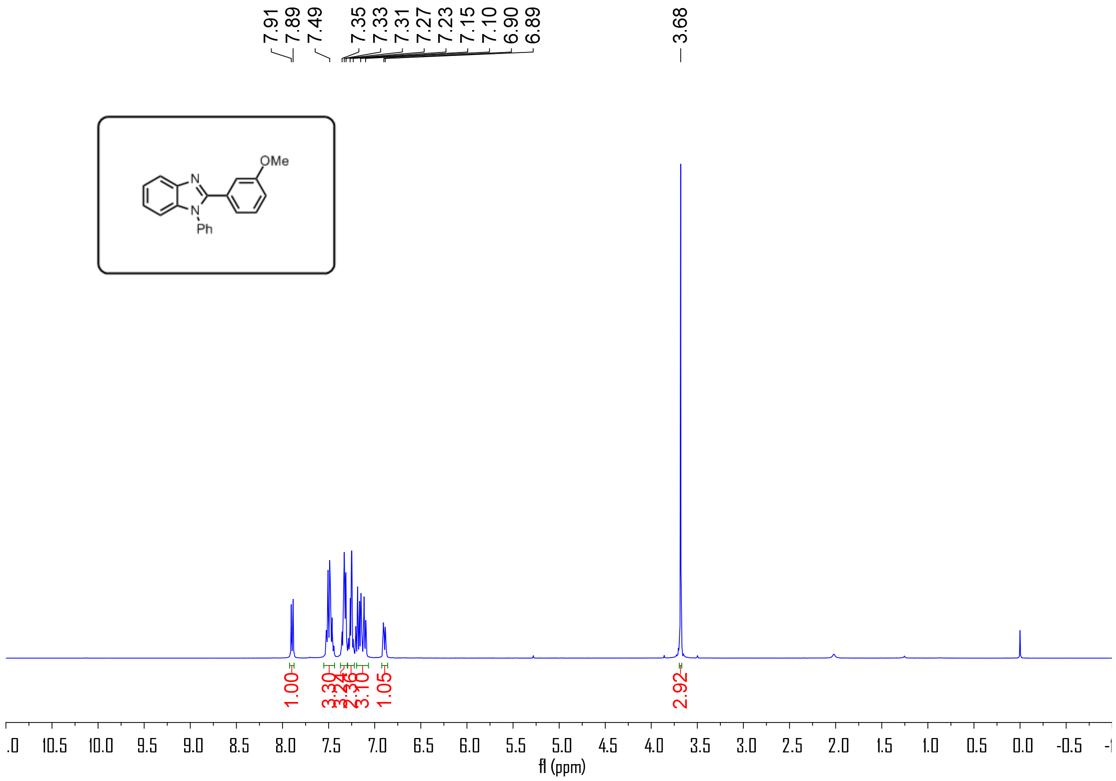

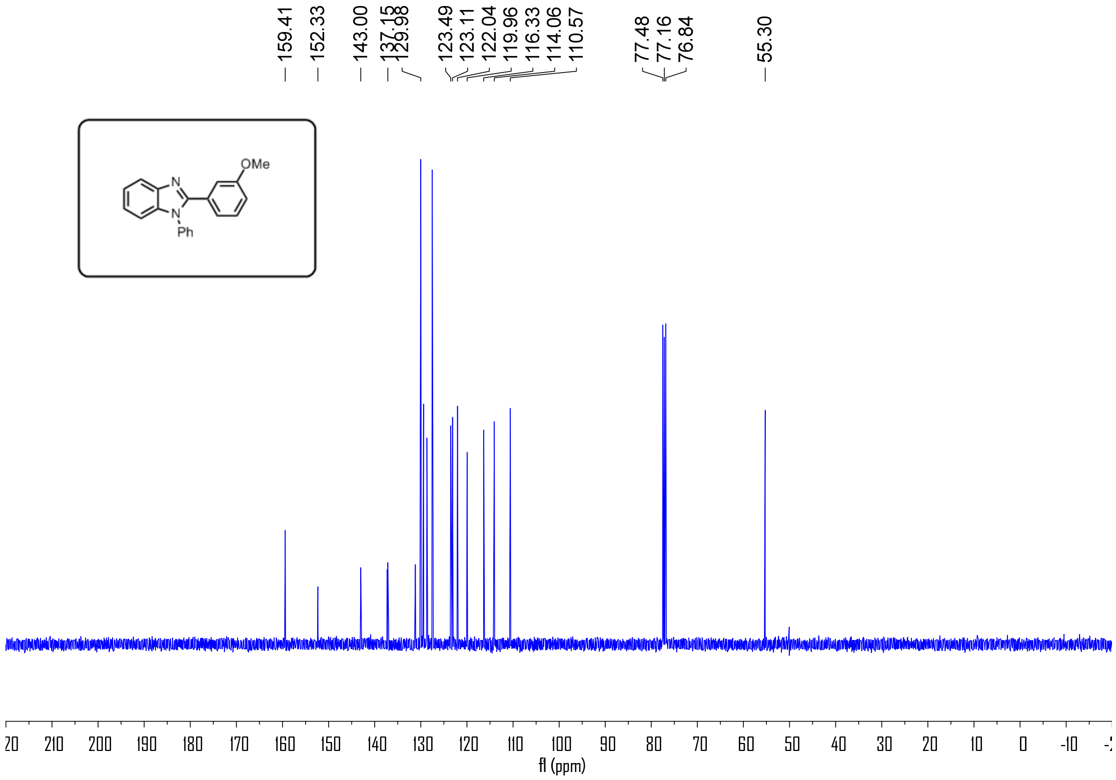

Supplementary Figure 43. ^{1}H and ^{13}C NMR spectra of **1al** in $CDCl_3$.

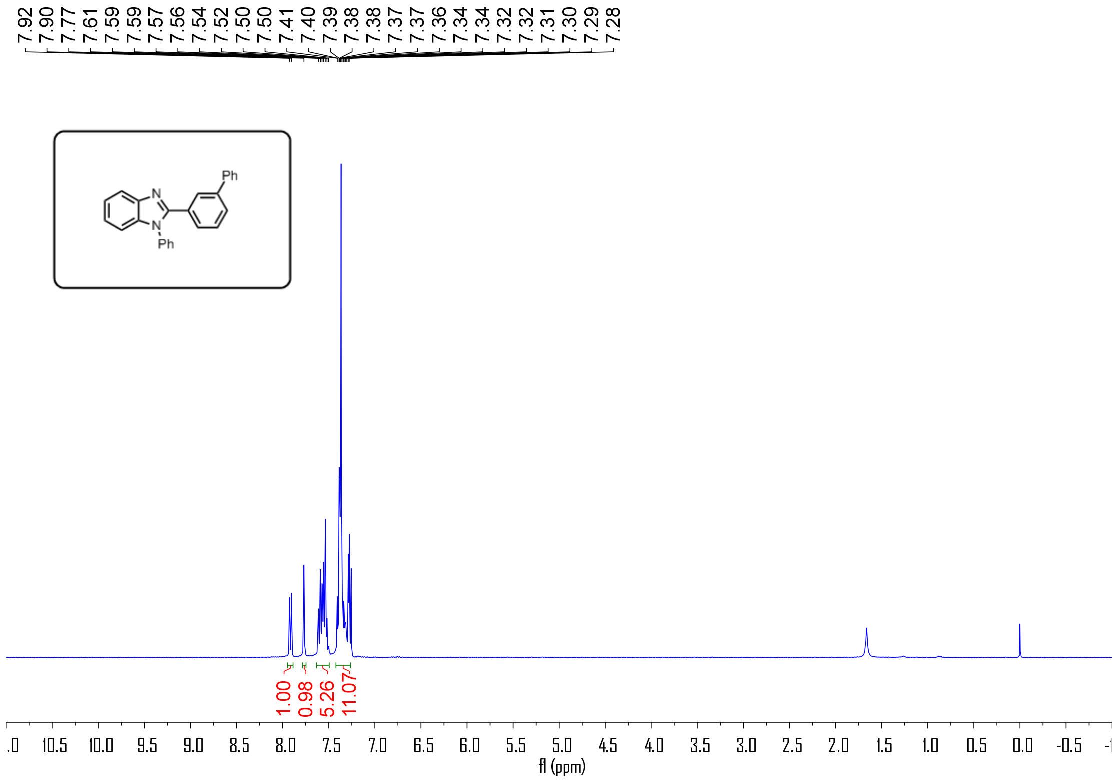

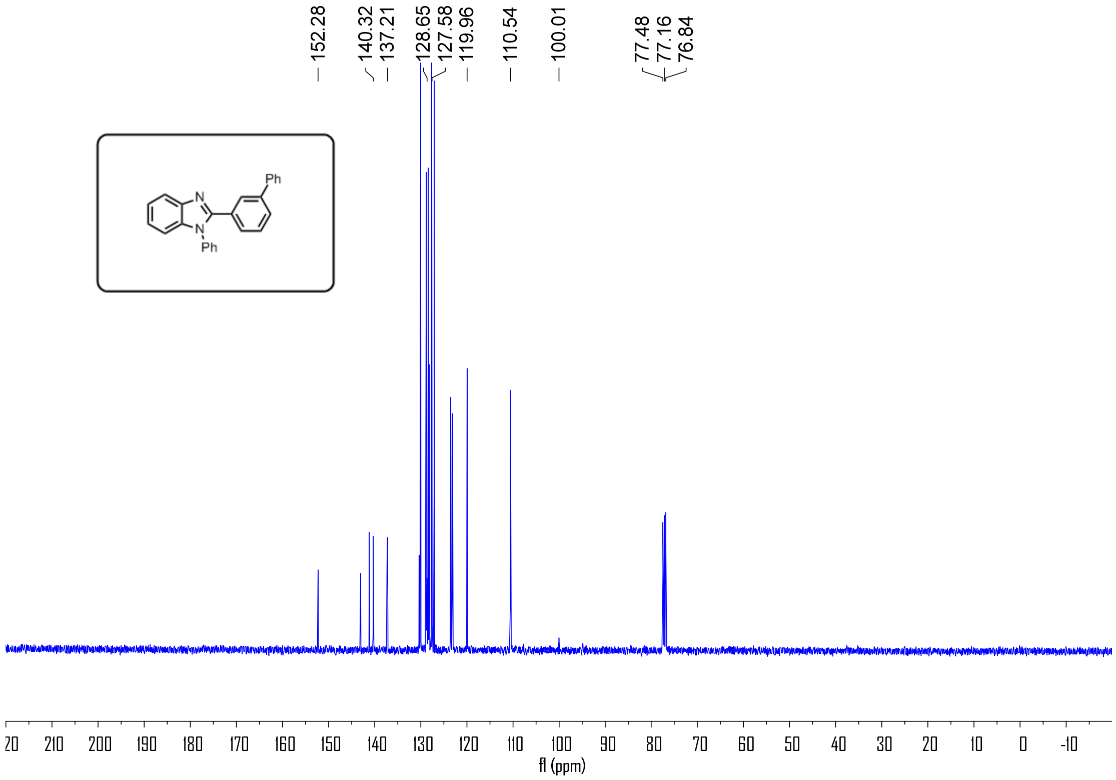

Supplementary Figure 44. ^{1}H and ^{13}C NMR spectra of **1am** in $CDCl_3$.

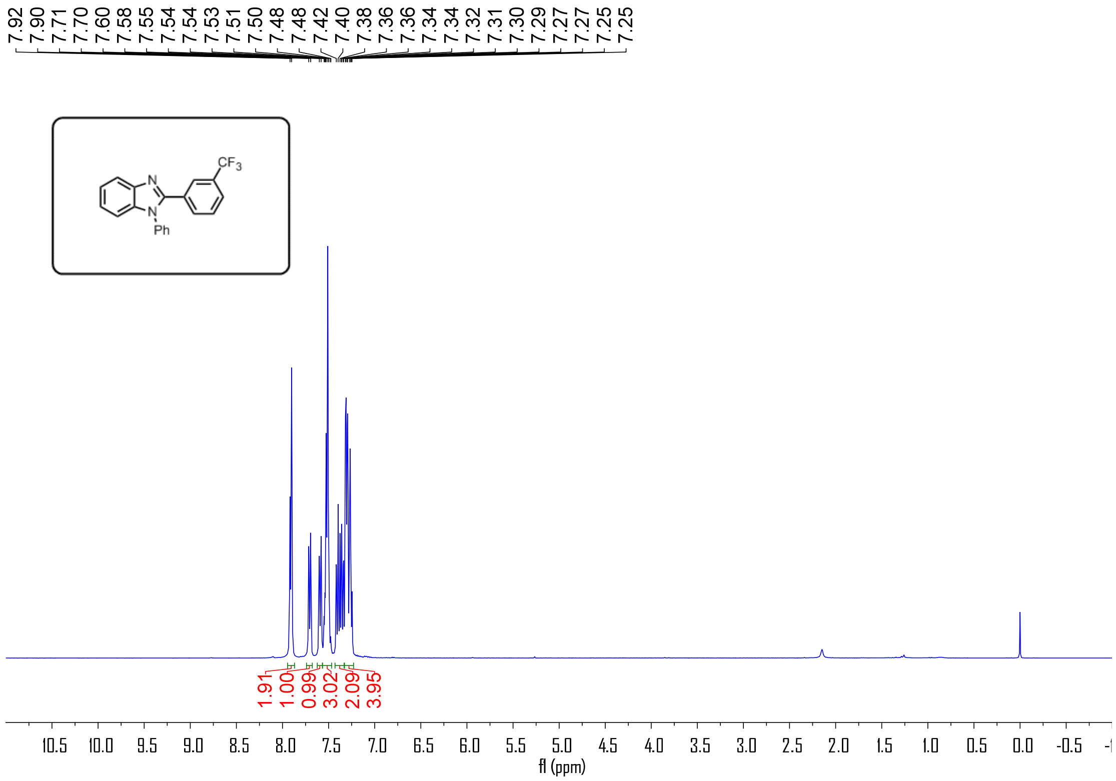

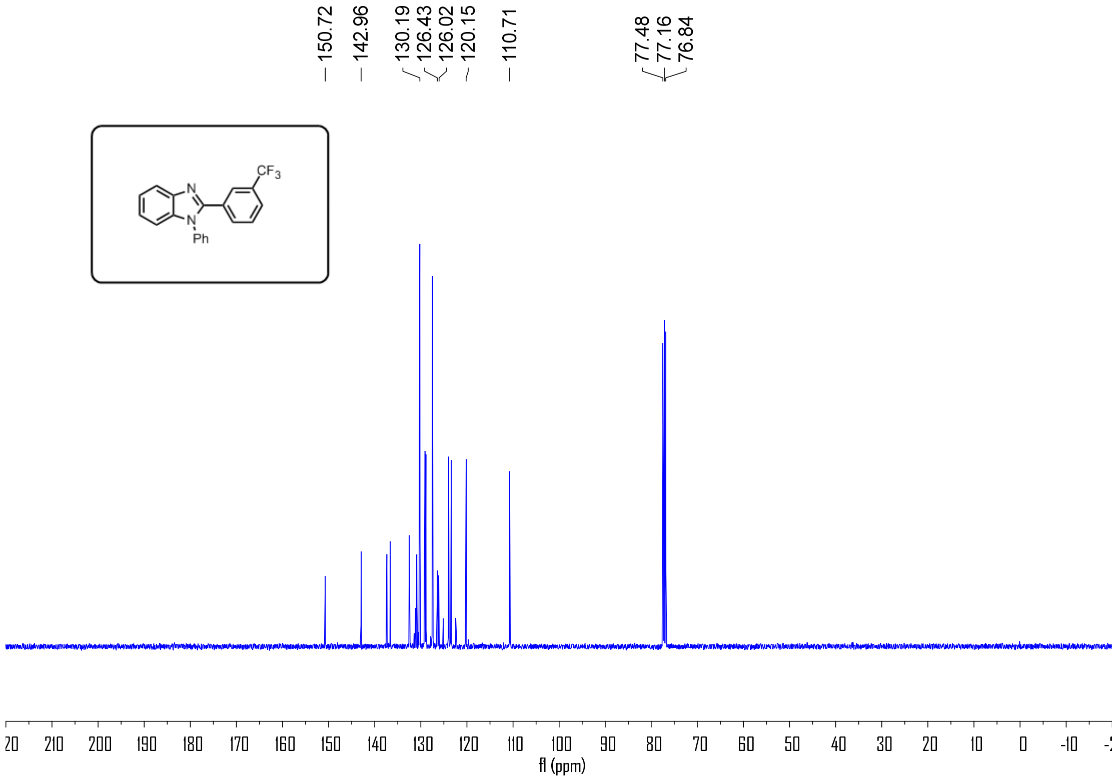

Supplementary Figure 45. ^{1}H and ^{13}C NMR spectra of **1an** in $CDCl_3$.

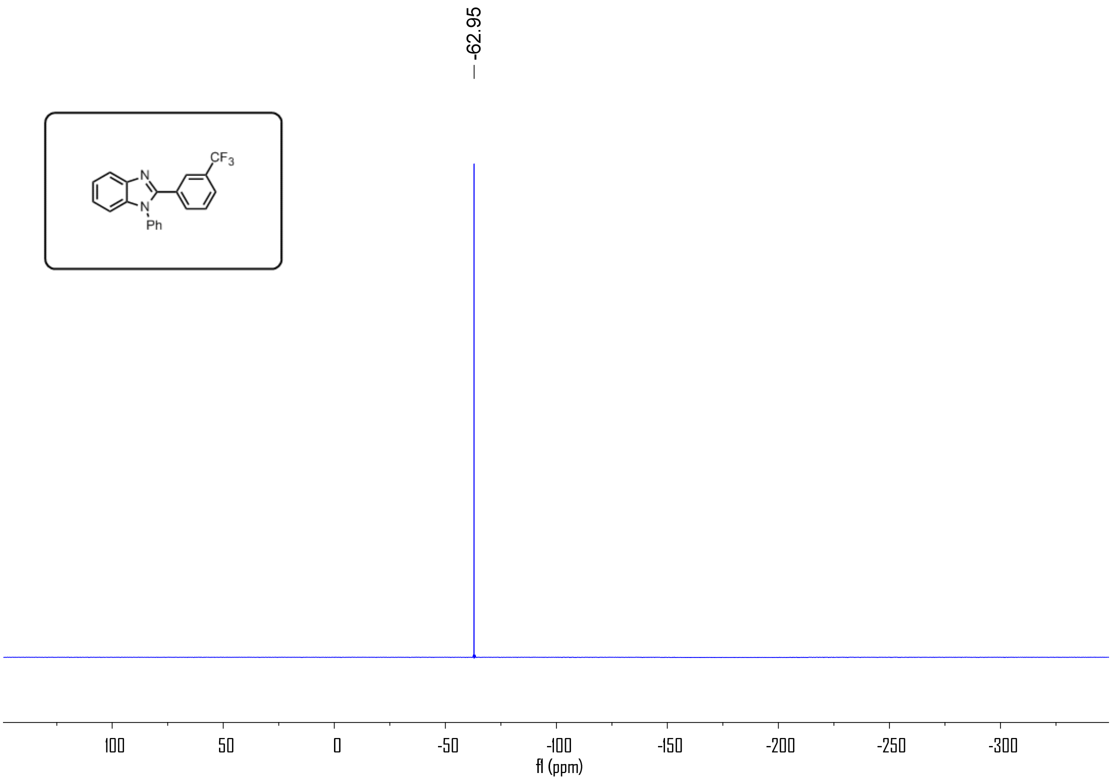

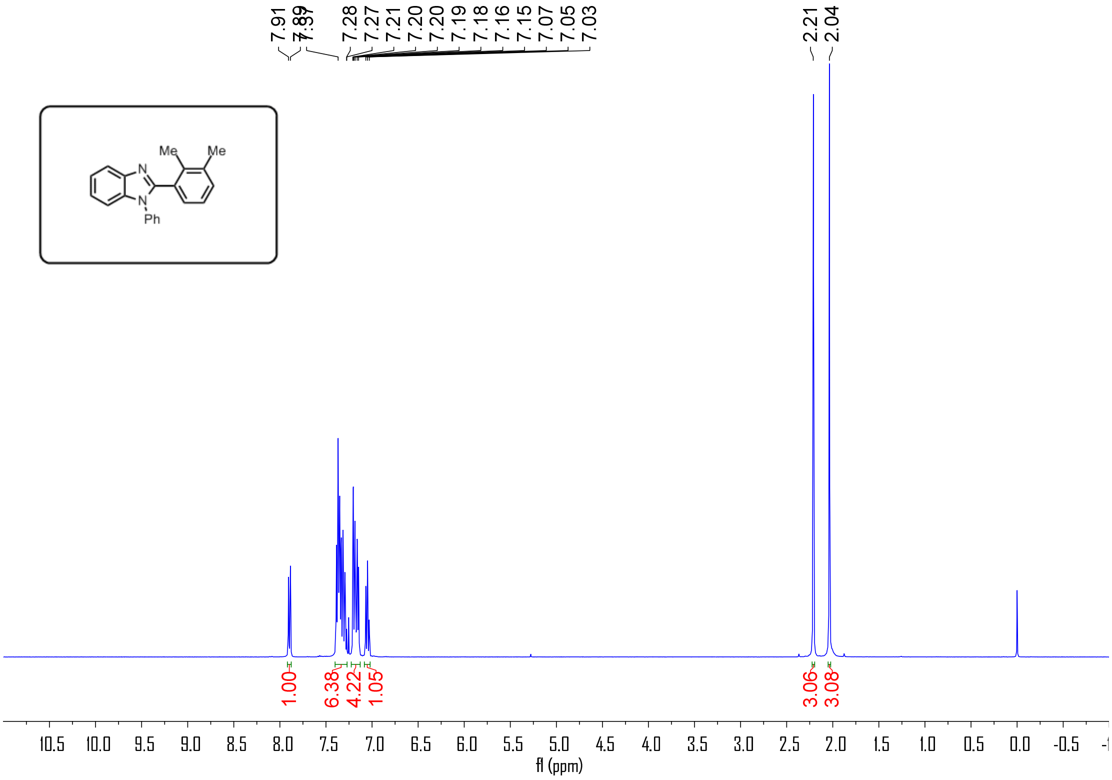

Supplementary Figure 46. ^{19}F (**1an**) and ^{1}H (**1ao**) NMR spectra in $CDCl_3$.

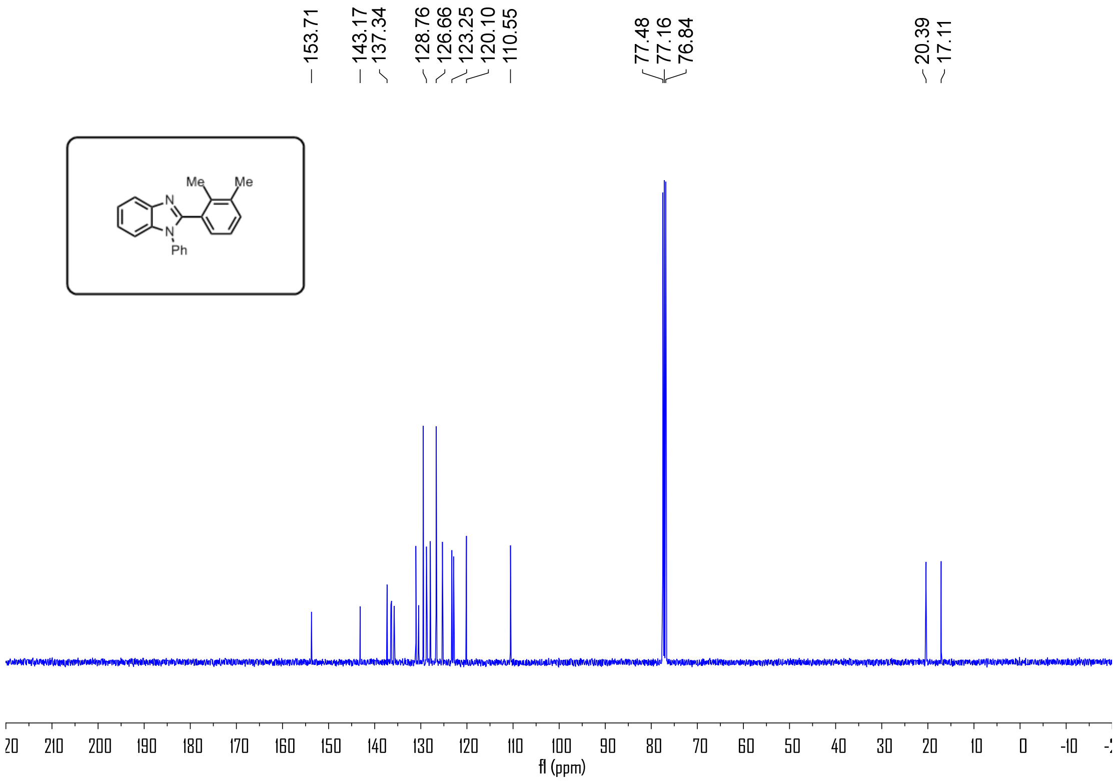

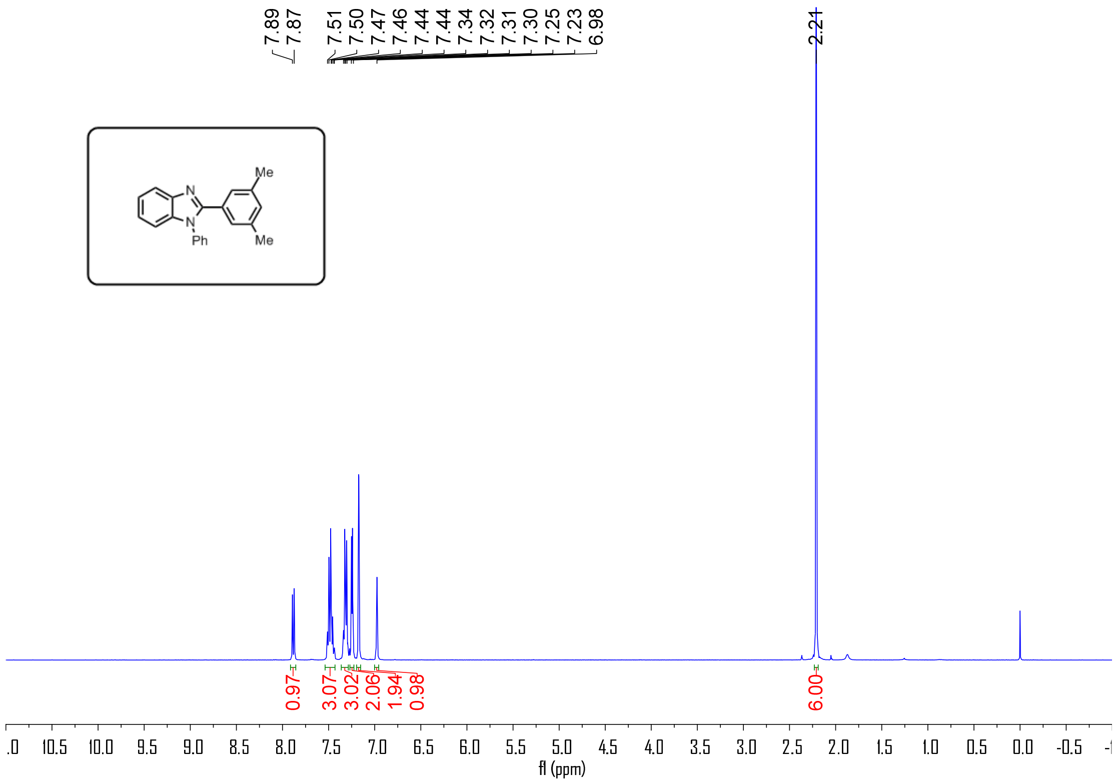

Supplementary Figure 47. ^{13}C (**1ao**) and ^{1}H (**1ap**) NMR spectra in $CDCl_3$.

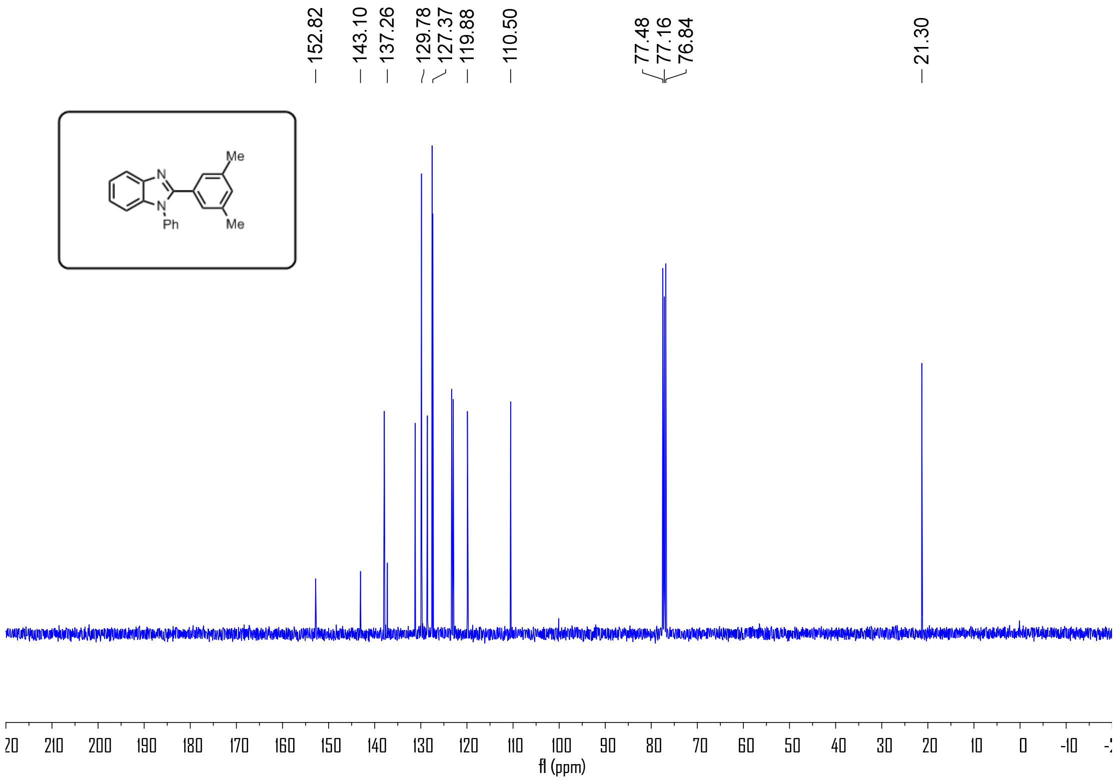

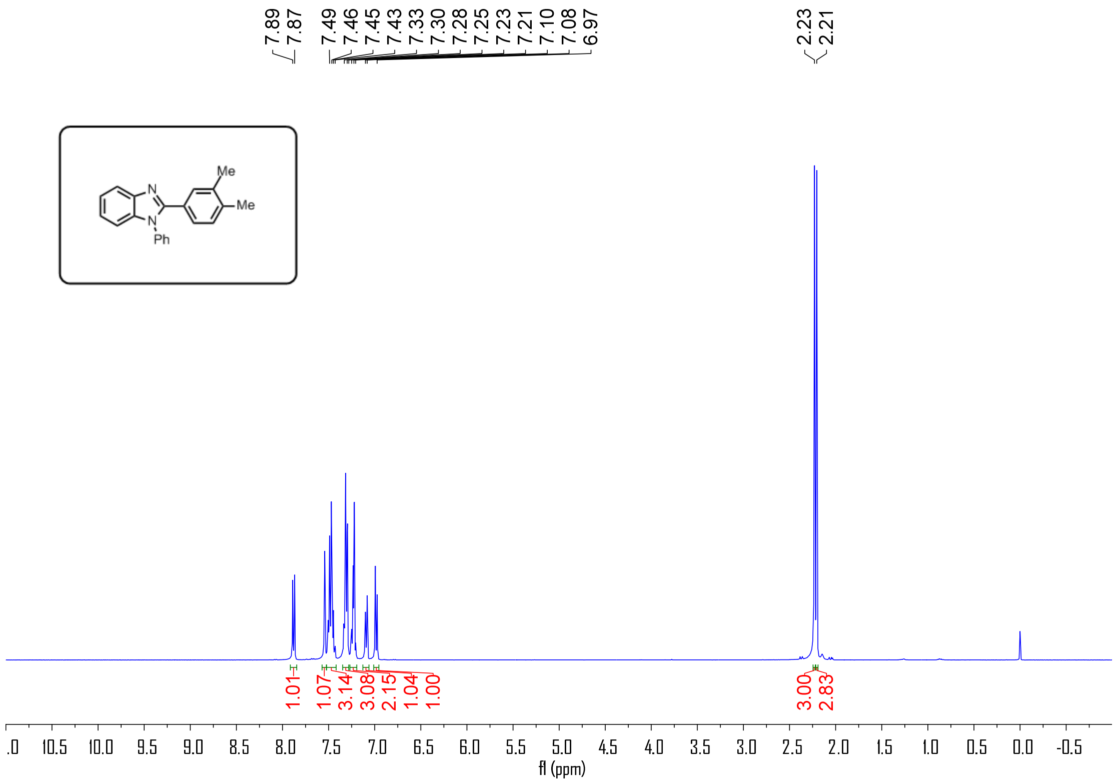

Supplementary Figure 48. ^{13}C (**1ap**) and ^{1}H (**1aq**) NMR spectra in $CDCl_3$.

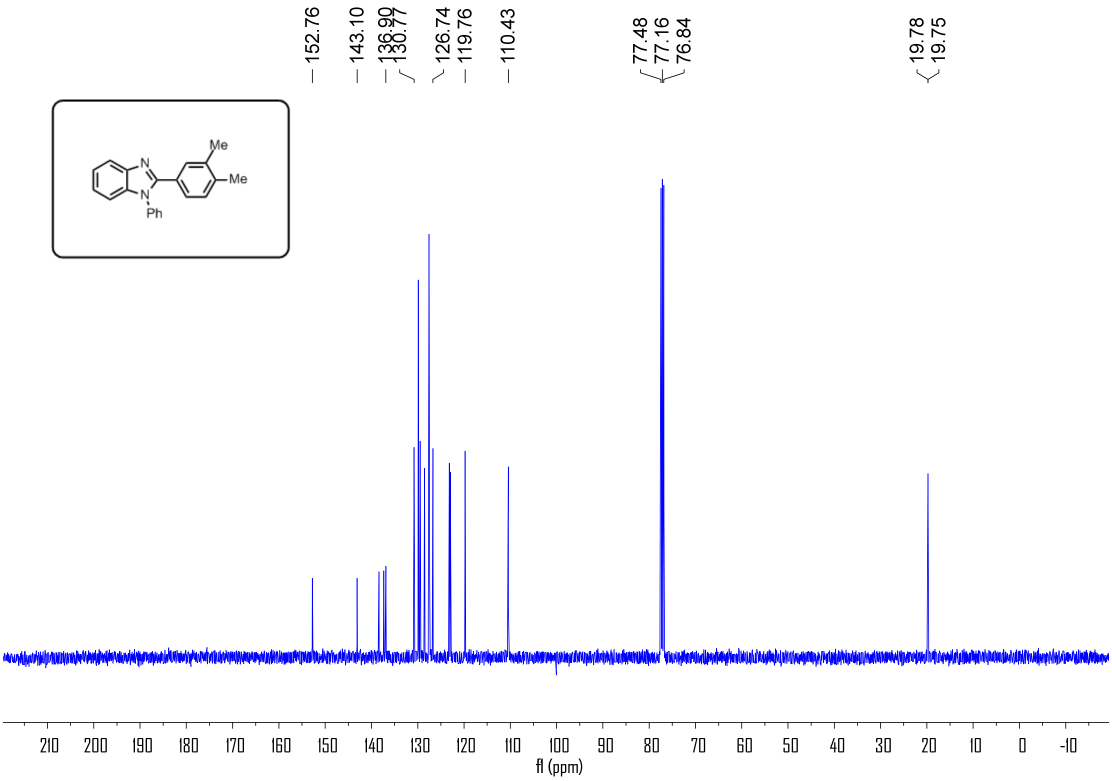

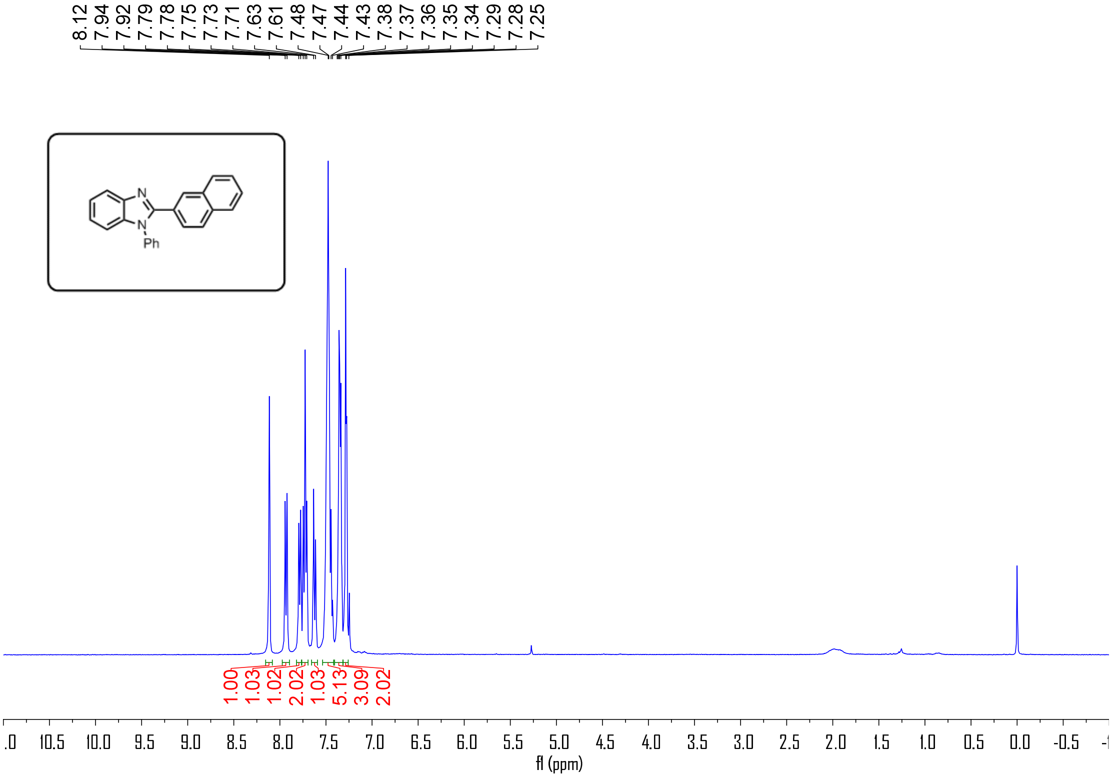

Supplementary Figure 49. ^{13}C (**1aq**) and ^{1}H (**1ar**) NMR spectra in $CDCl_3$.

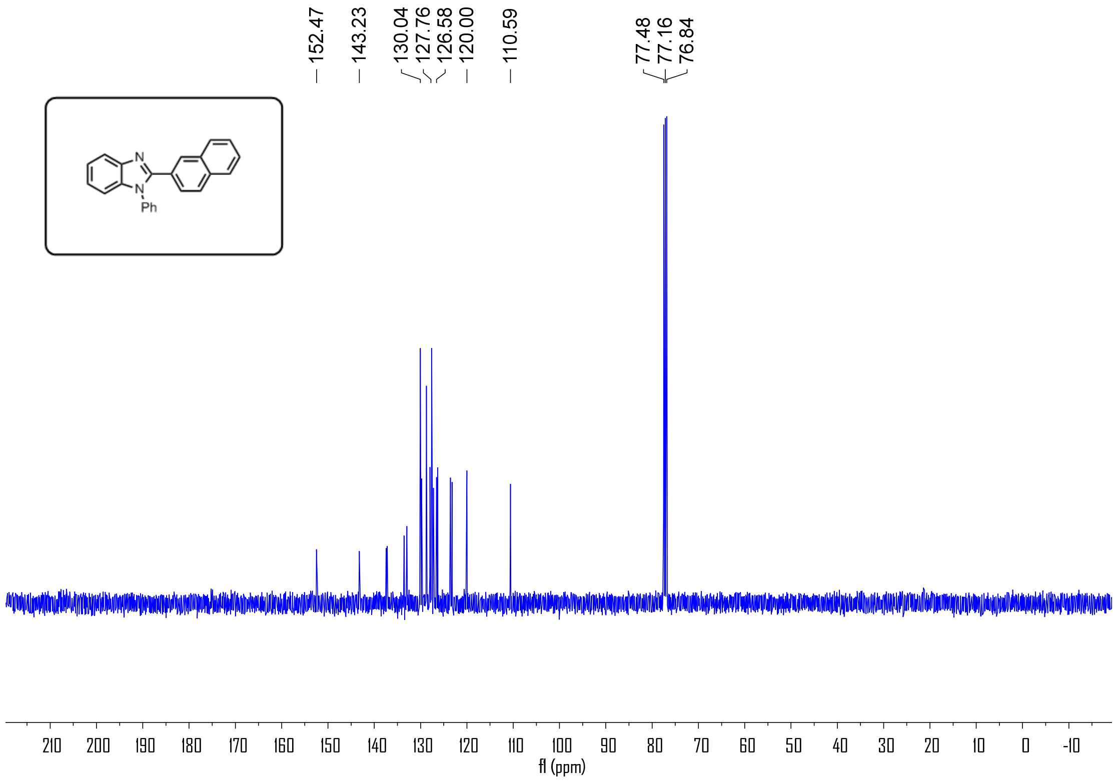

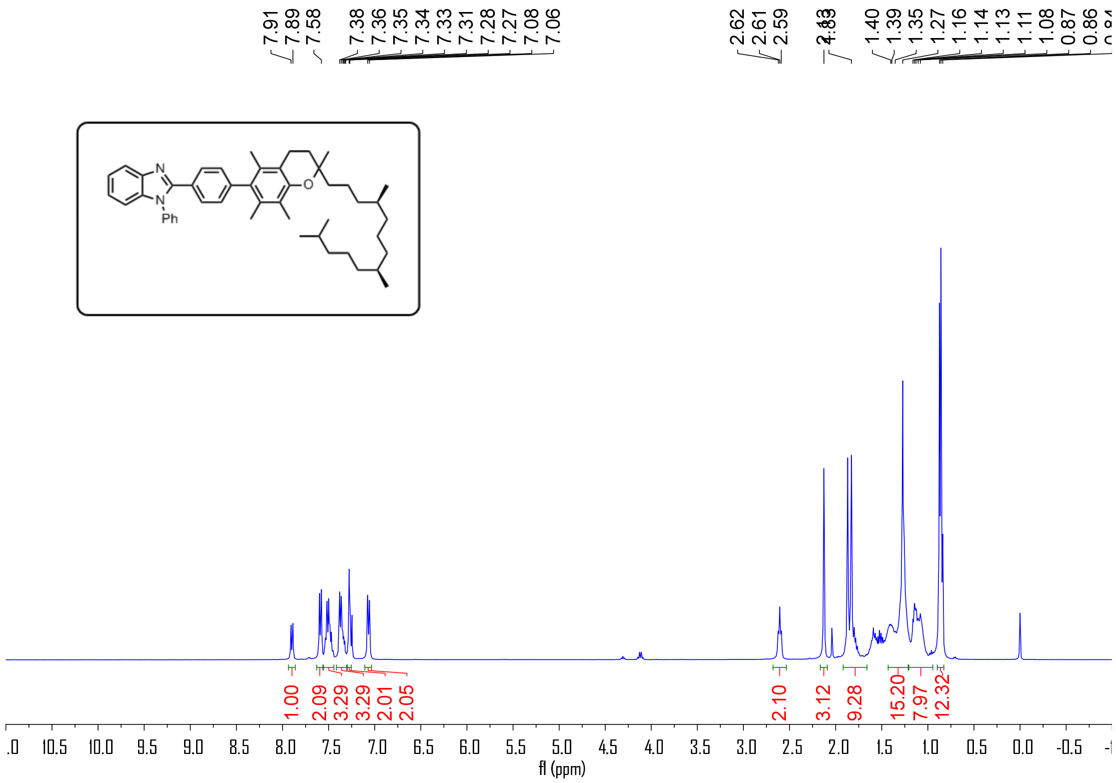

Supplementary Figure 50. ^{13}C (**1ar**) and ^{1}H (**S10**) NMR spectra in $CDCl_3$.

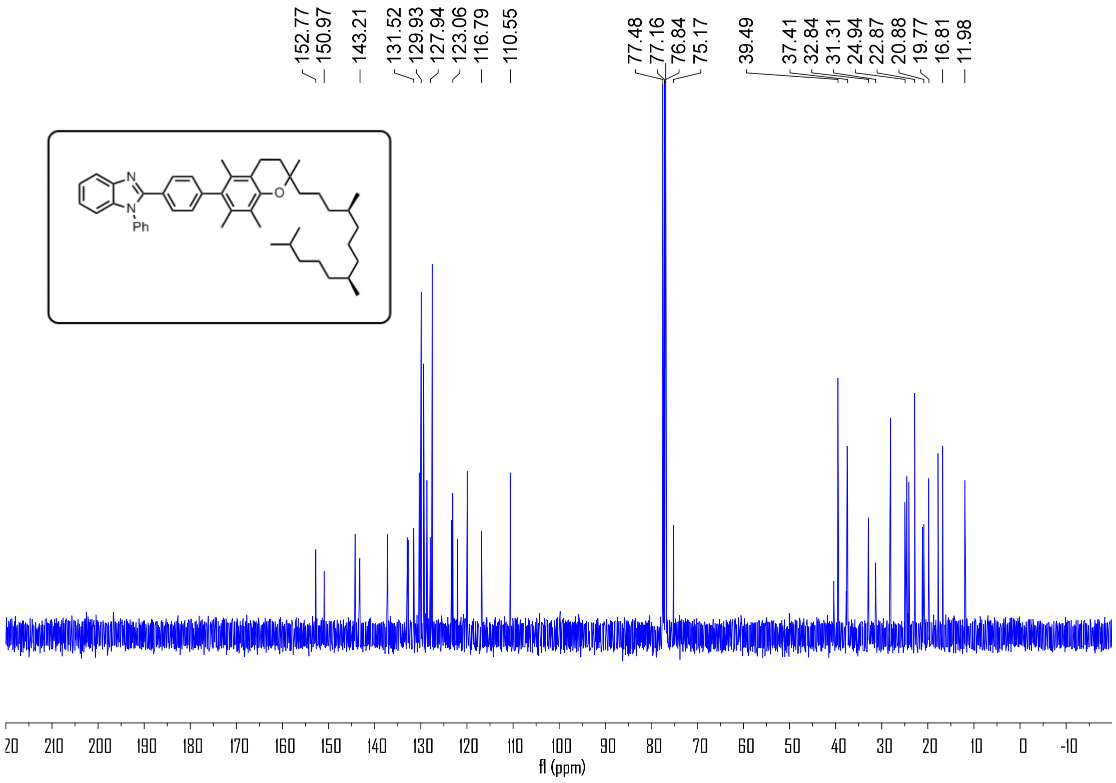

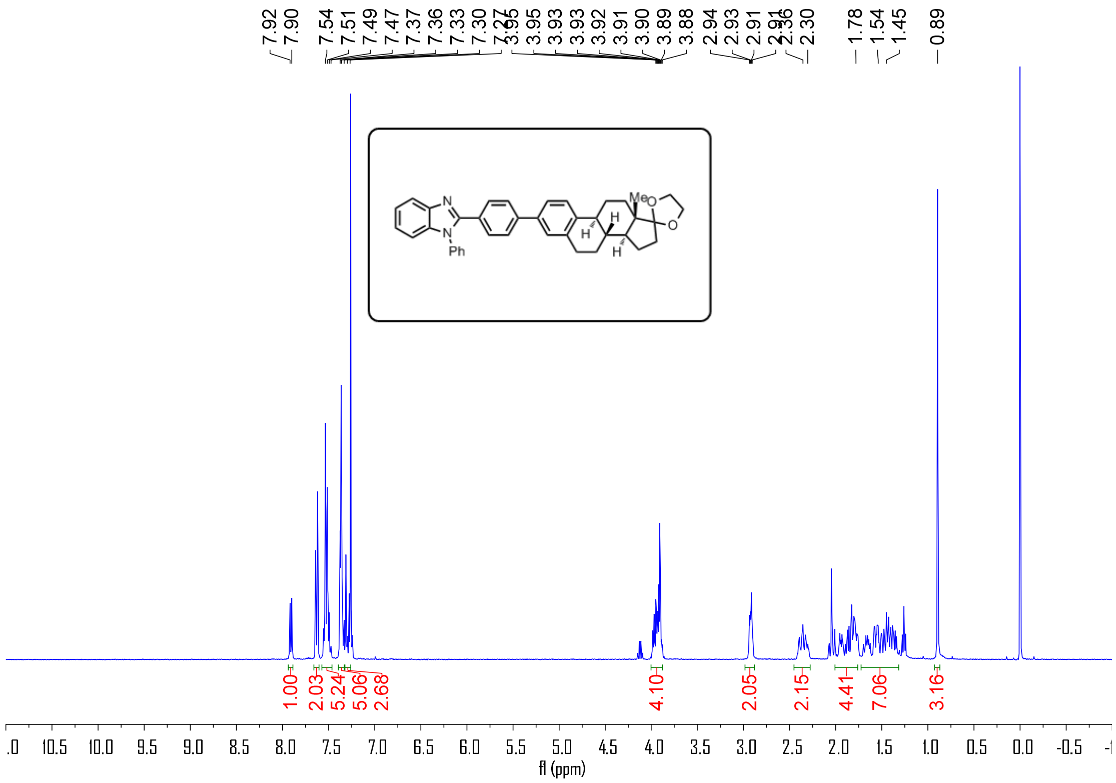

Supplementary Figure 51. ^{13}C (**S10**) and ^{1}H (**S11**) NMR spectra in $CDCl_3$.

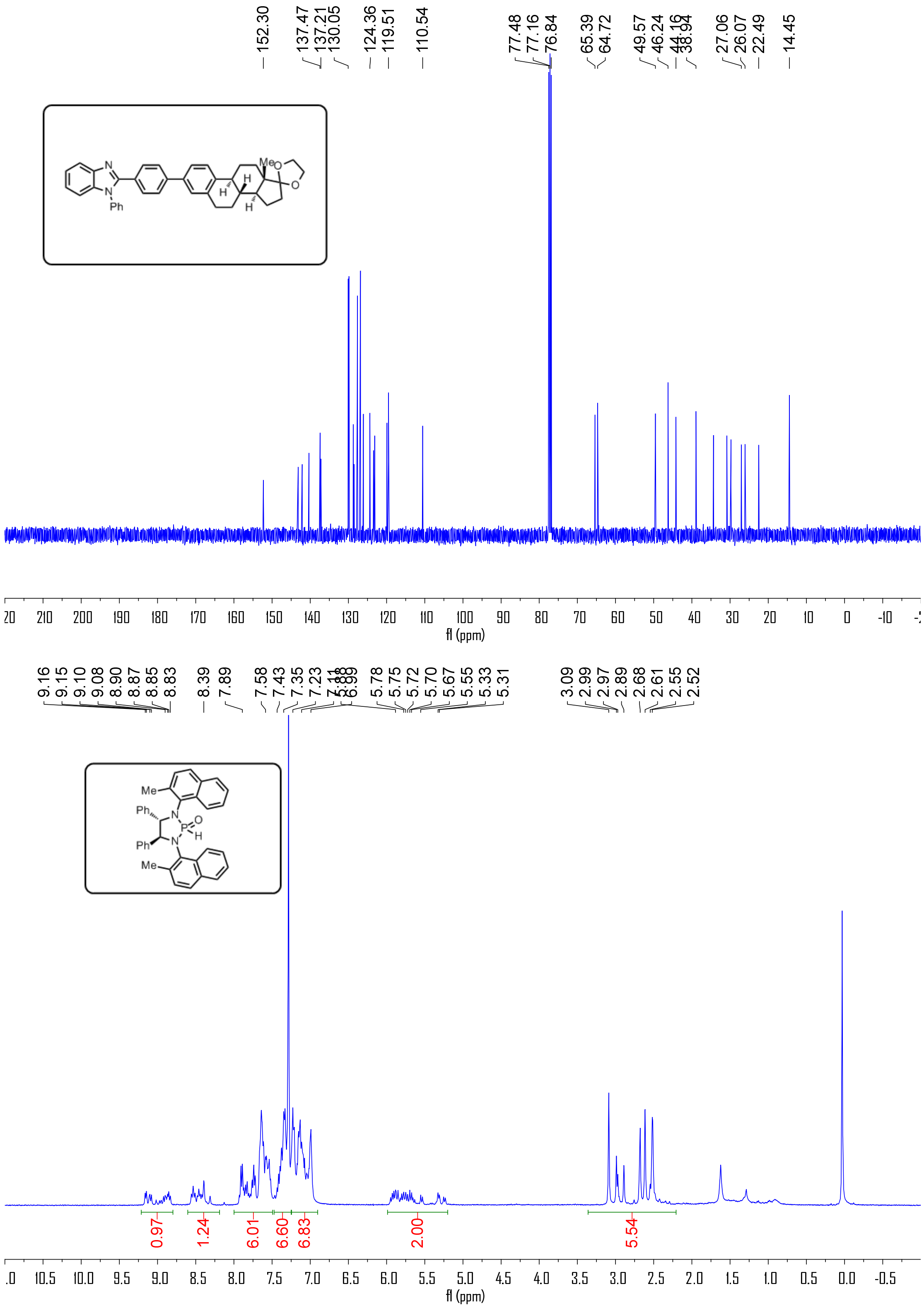

Supplementary Figure 52. ^{13}C (**S11**) and ^{1}H (**PO-6**) NMR spectra in $CDCl_3$.

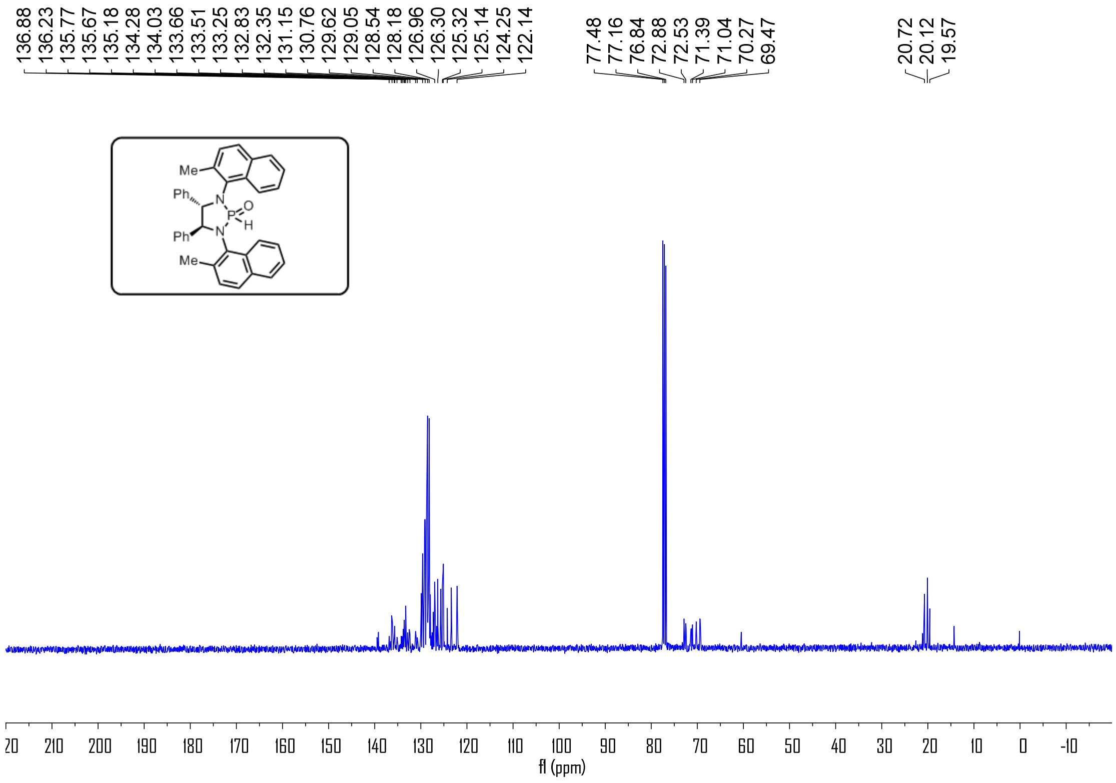

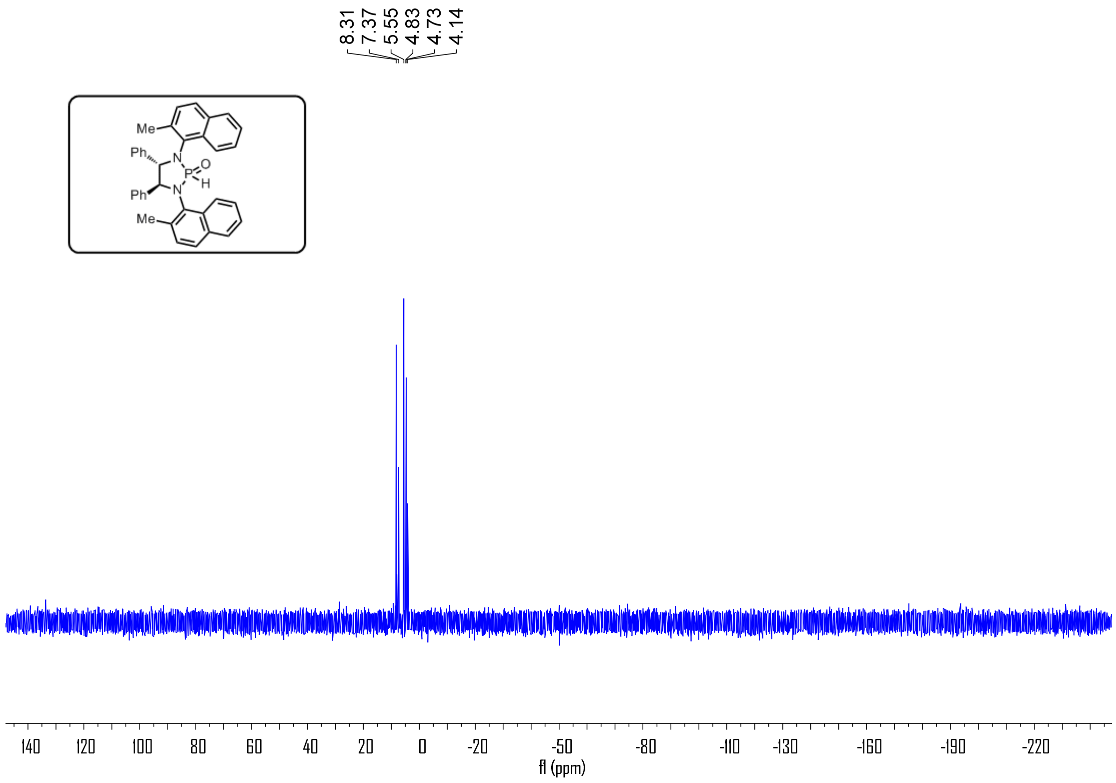

Supplementary Figure 53. ^{13}C and ^{31}P NMR spectra of **PO-6** in $CDCl_3$.

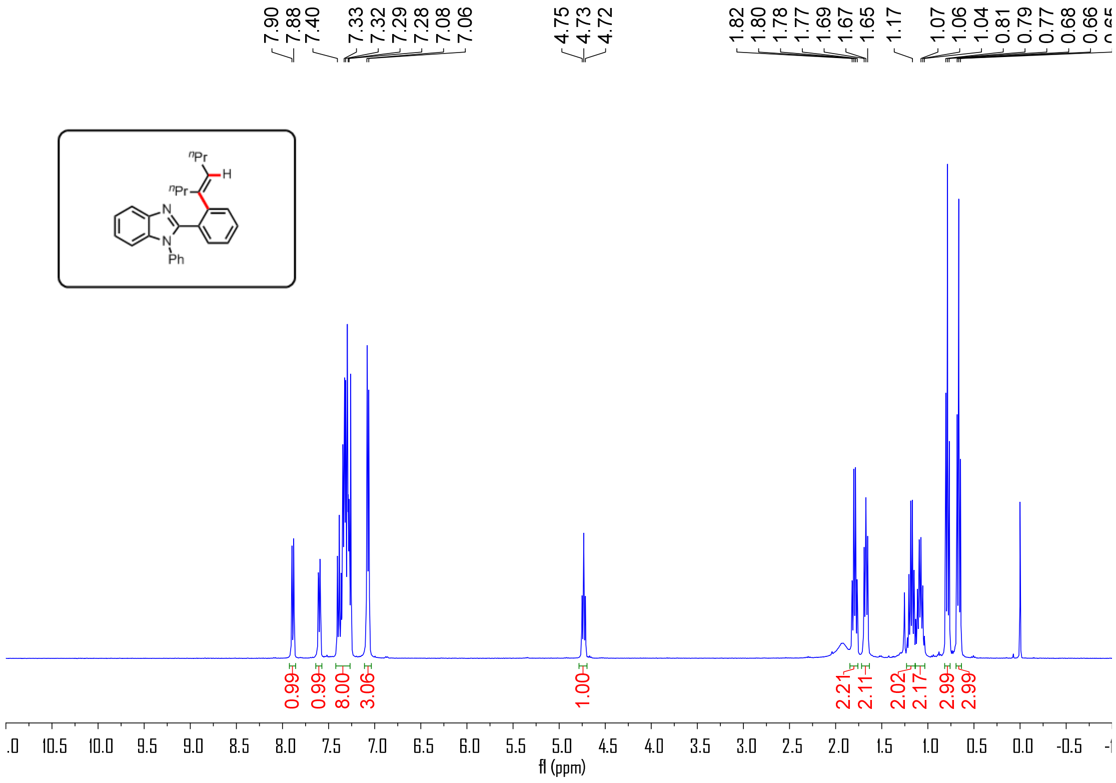

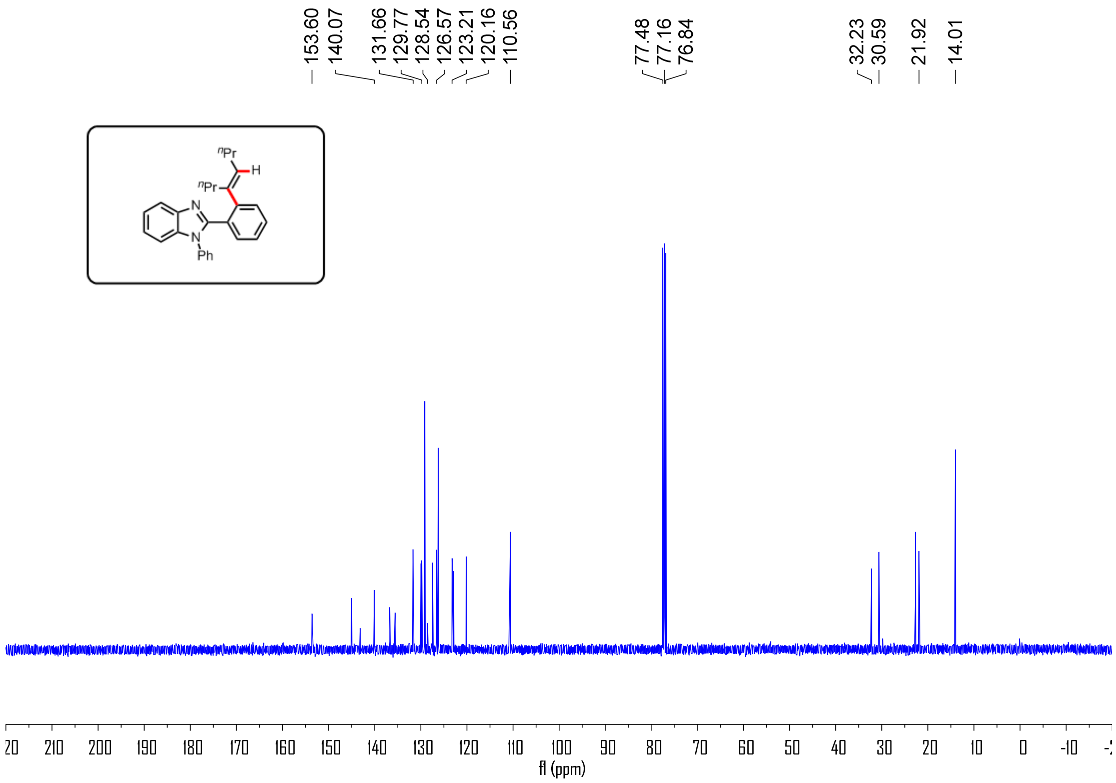

Supplementary Figure 54. ^{1}H and ^{13}C NMR spectra of **3a′** in $CDCl_3$.

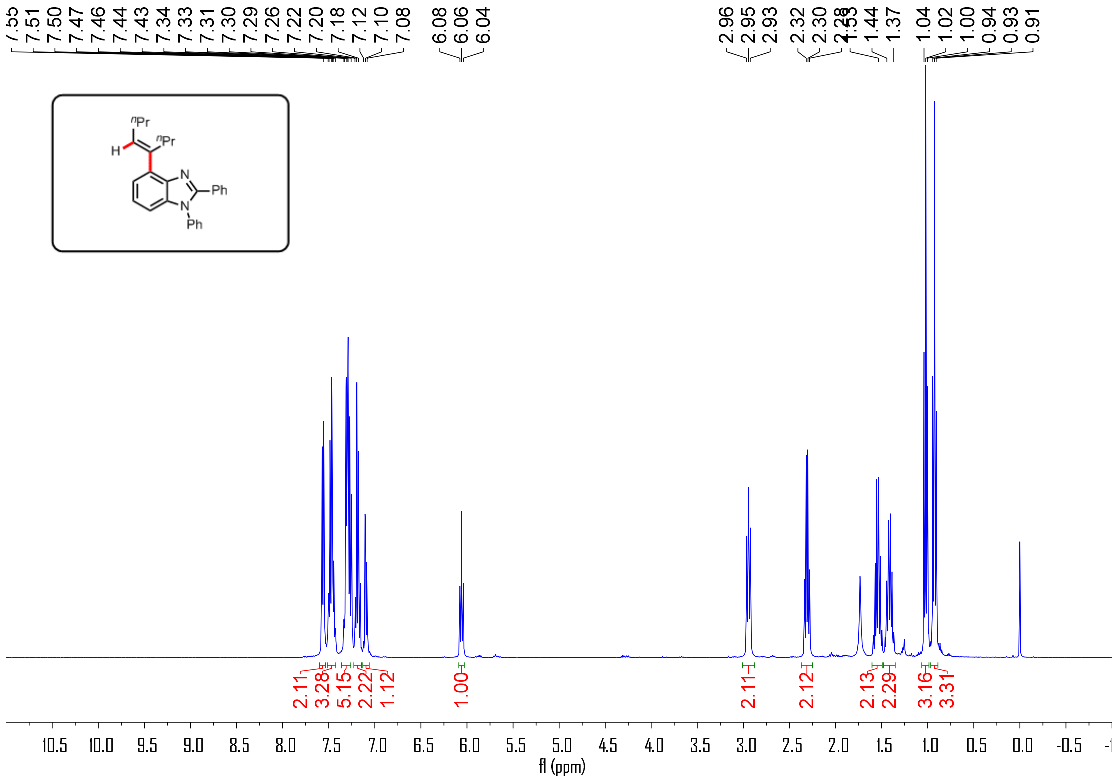

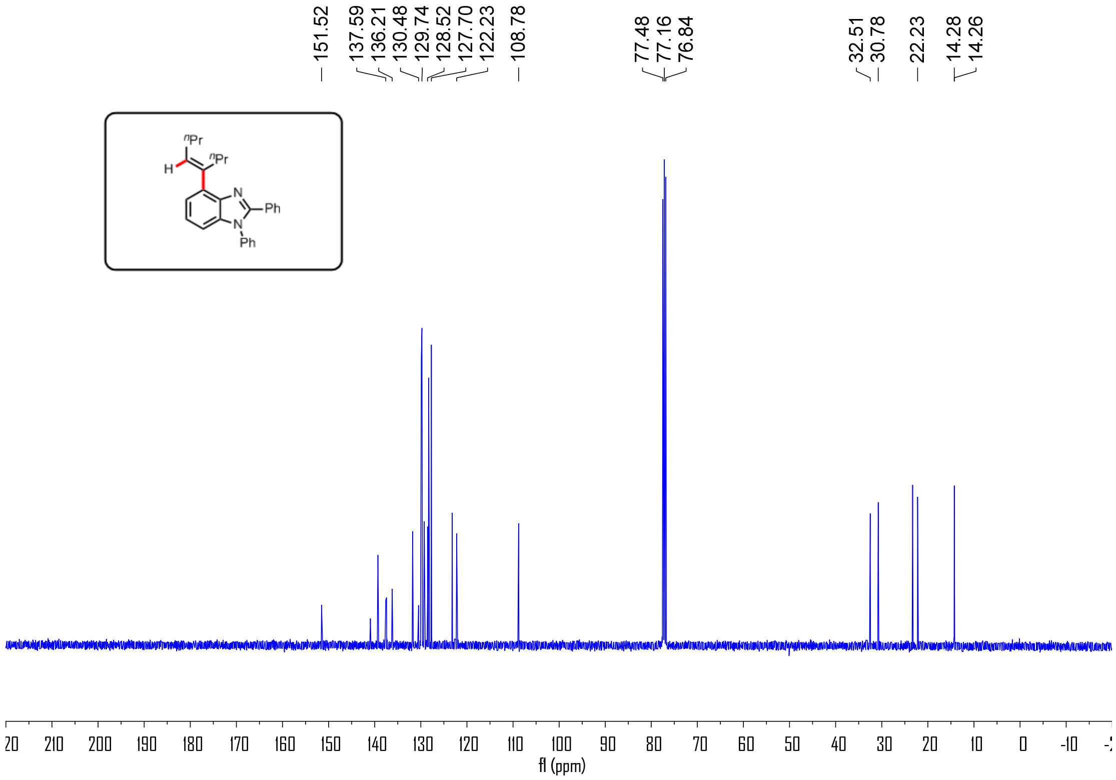

Supplementary Figure 55. ^{1}H (**3a**) and ^{13}C (**3a**) NMR spectra in $CDCl_3$.

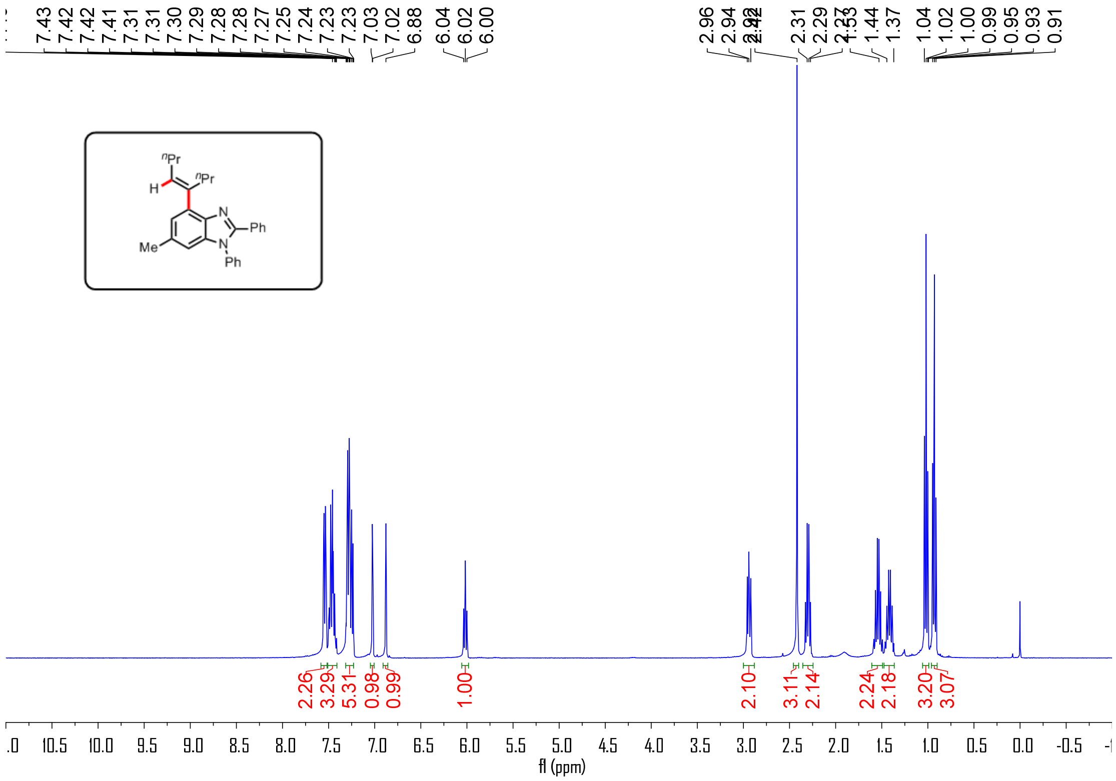

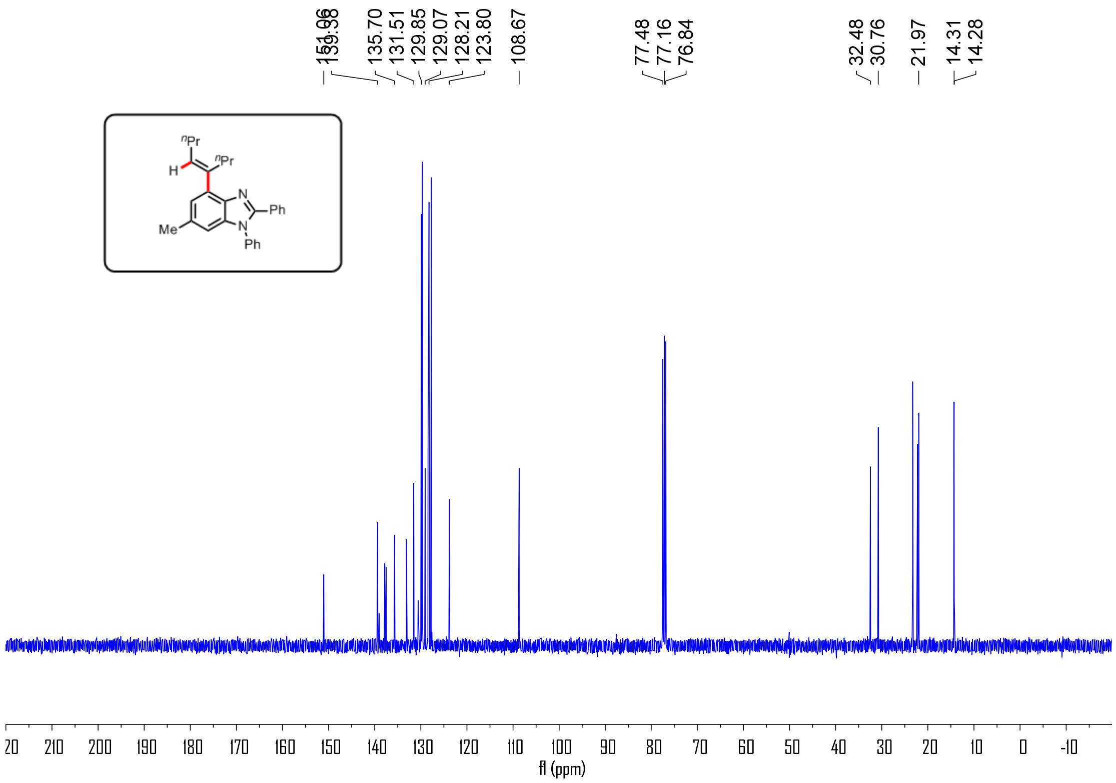

Supplementary Figure 56. ^{1}H (**3b**) and ^{13}C (**3b**) NMR spectra in $CDCl_3$.

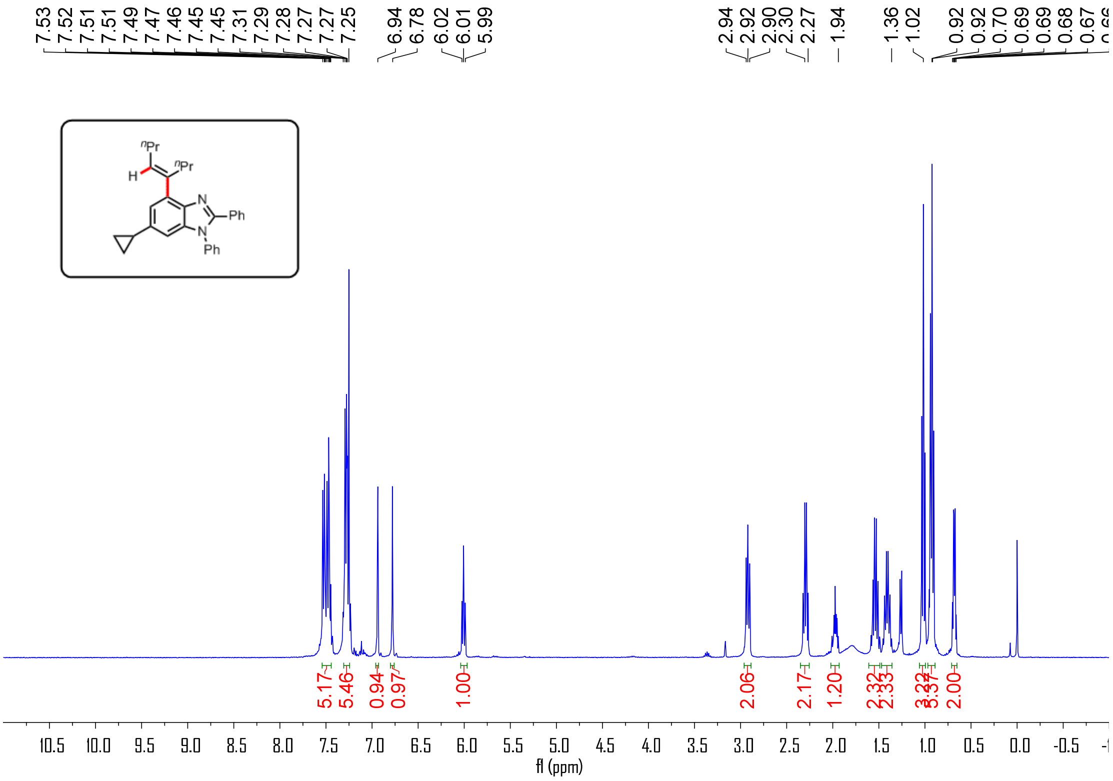

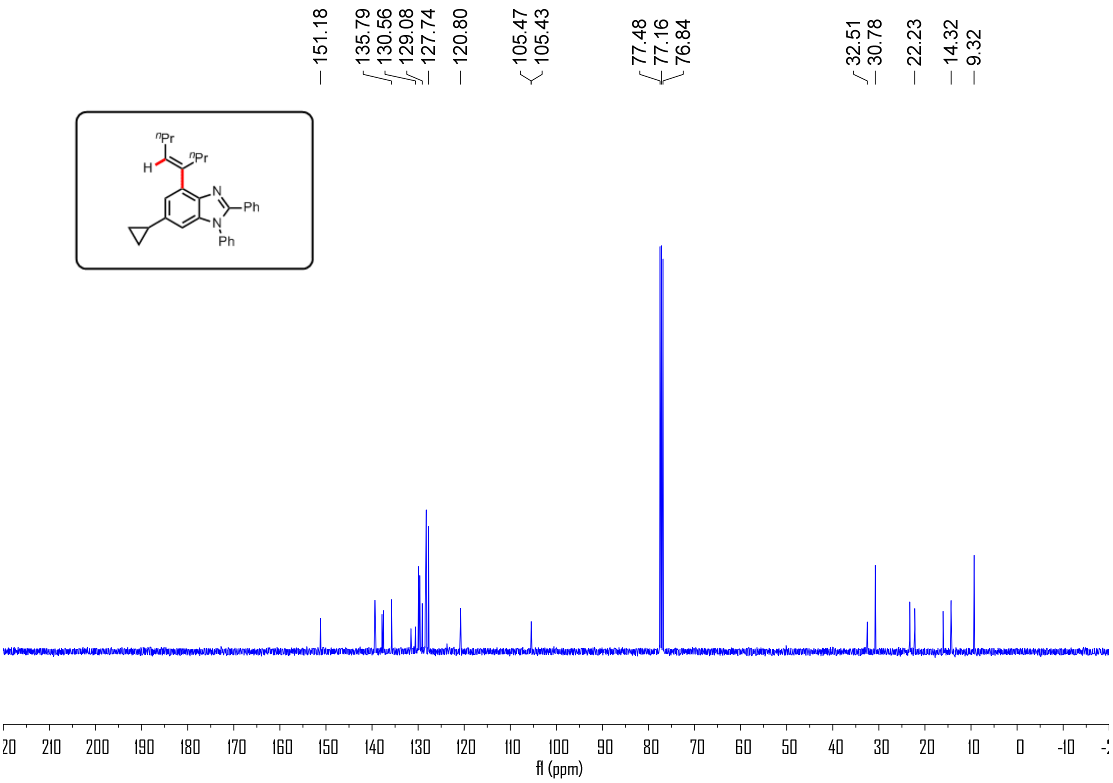

Supplementary Figure 57. ^{1}H (**3c**) and ^{13}C (**3c**) NMR spectra in $CDCl_3$.

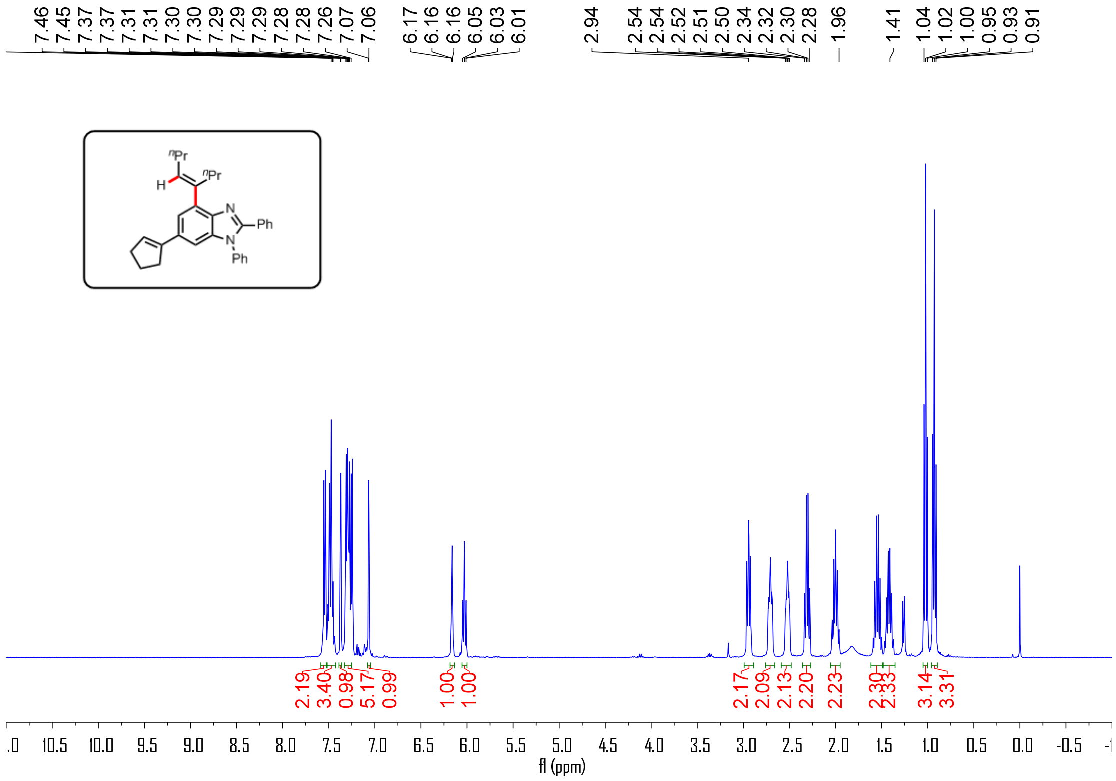

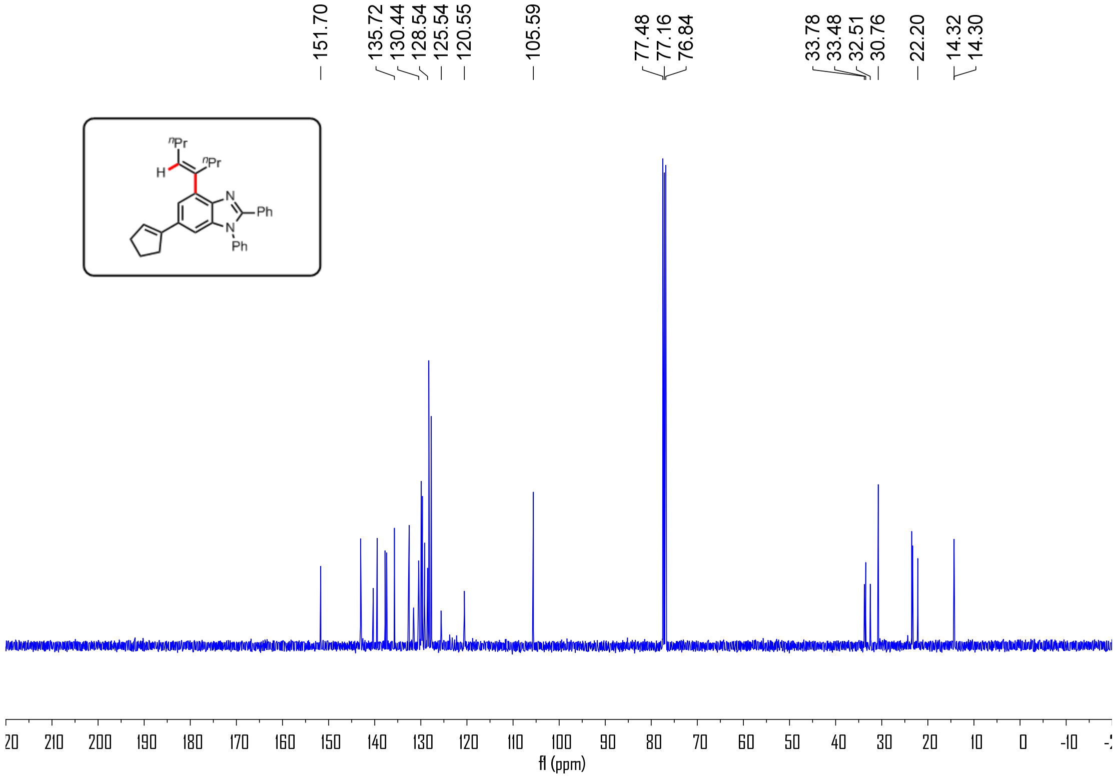

Supplementary Figure 58. ^{1}H (**3d**) and ^{13}C (**3d**) NMR spectra in $CDCl_3$.

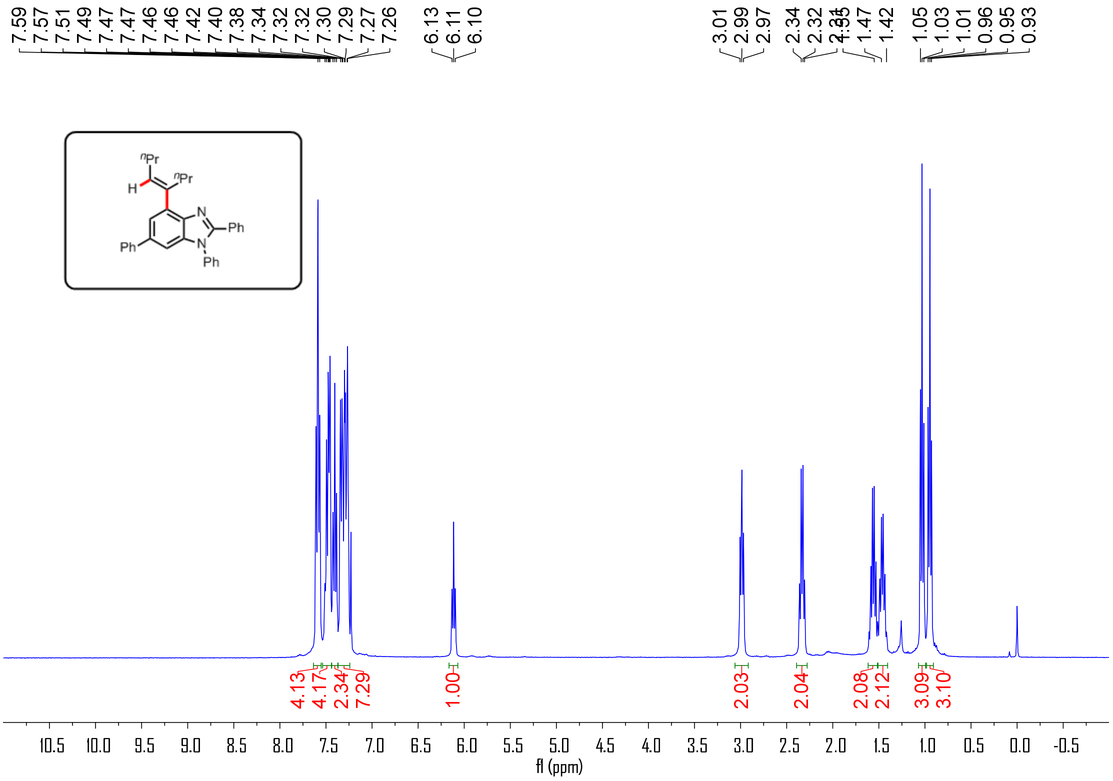

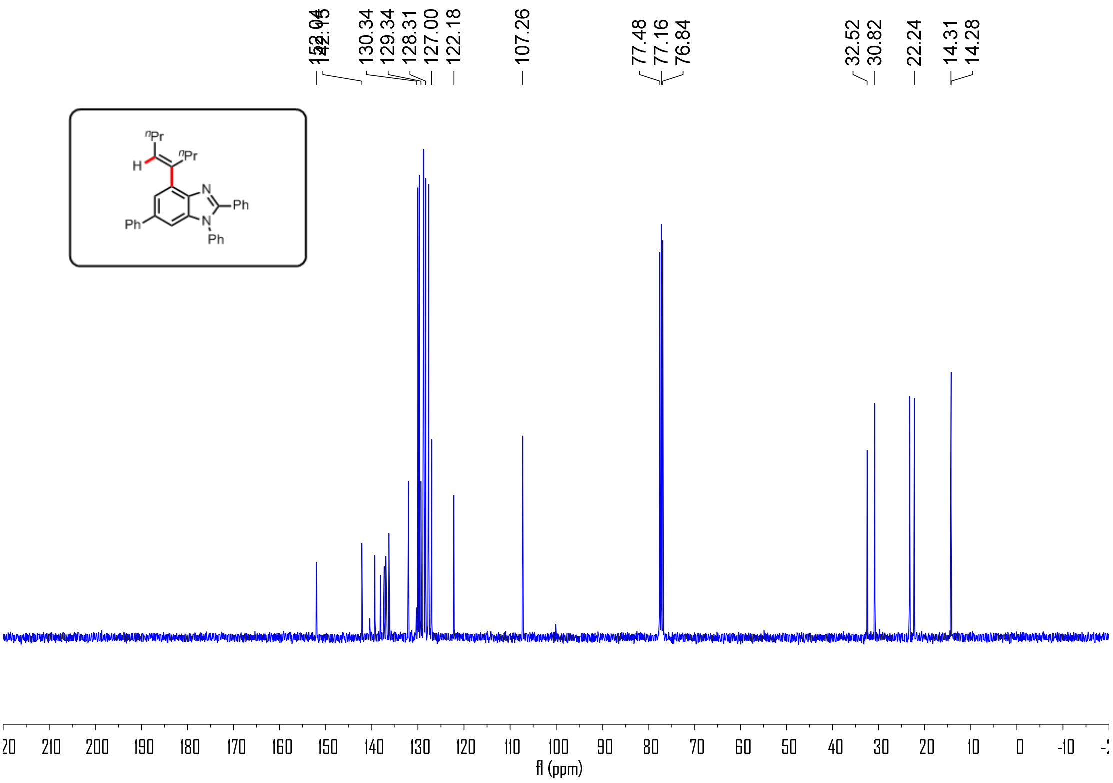

Supplementary Figure 59. ^{1}H (**3e**) and ^{13}C (**3e**) NMR spectra in $CDCl_3$.

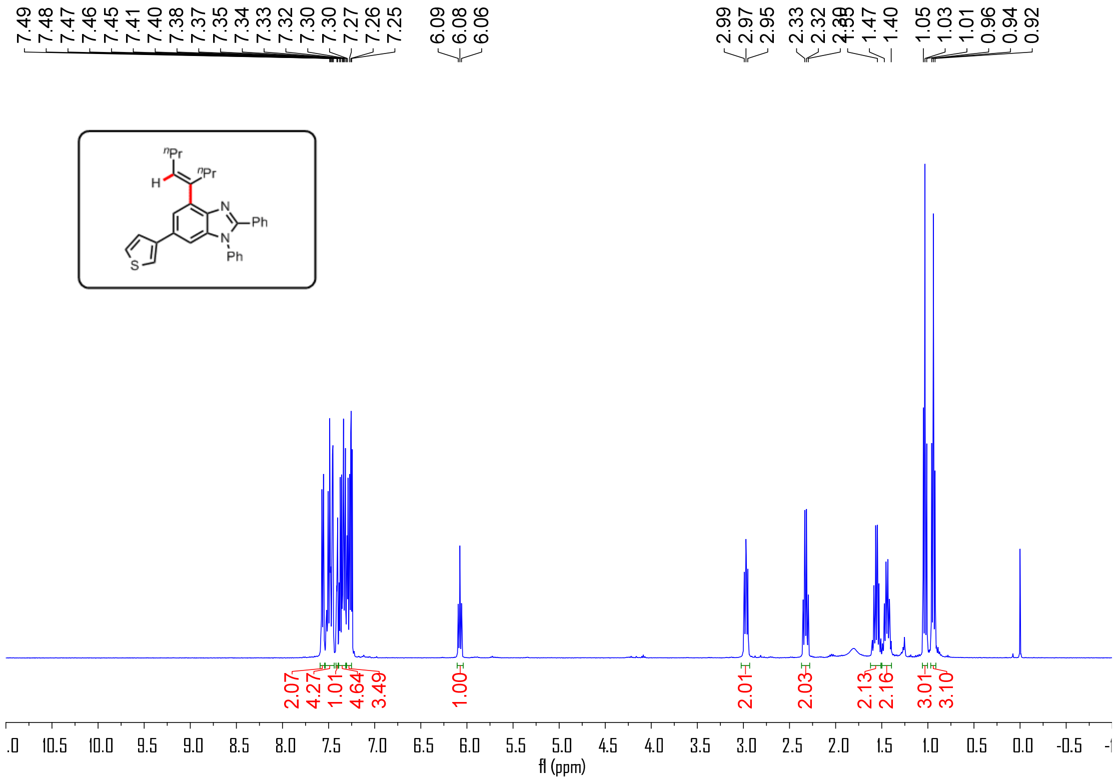

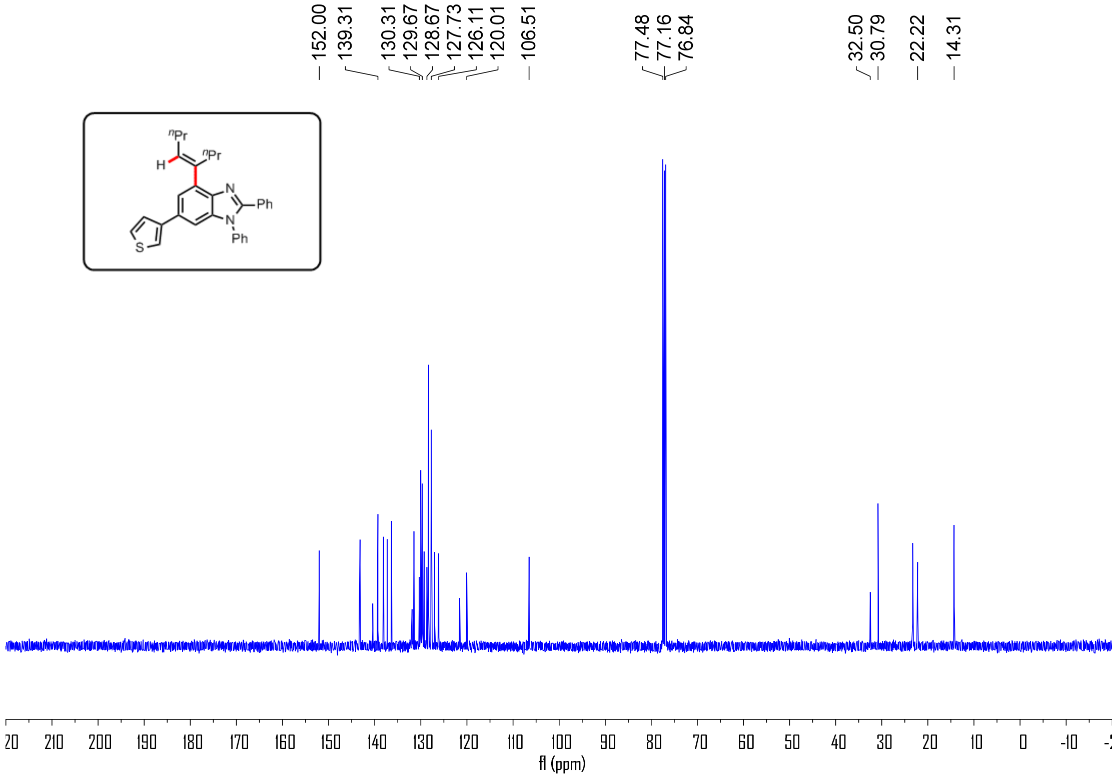

Supplementary Figure 60. ^{1}H (**3f**) and ^{13}C (**3f**) NMR spectra in $CDCl_3$.

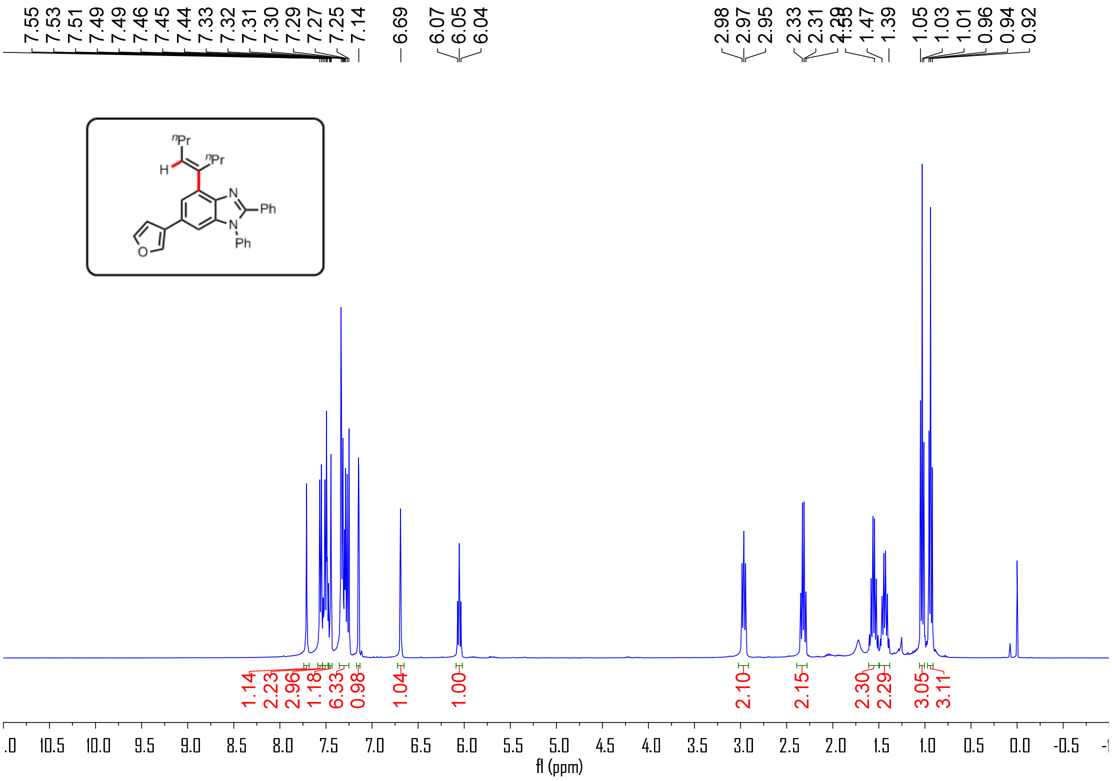

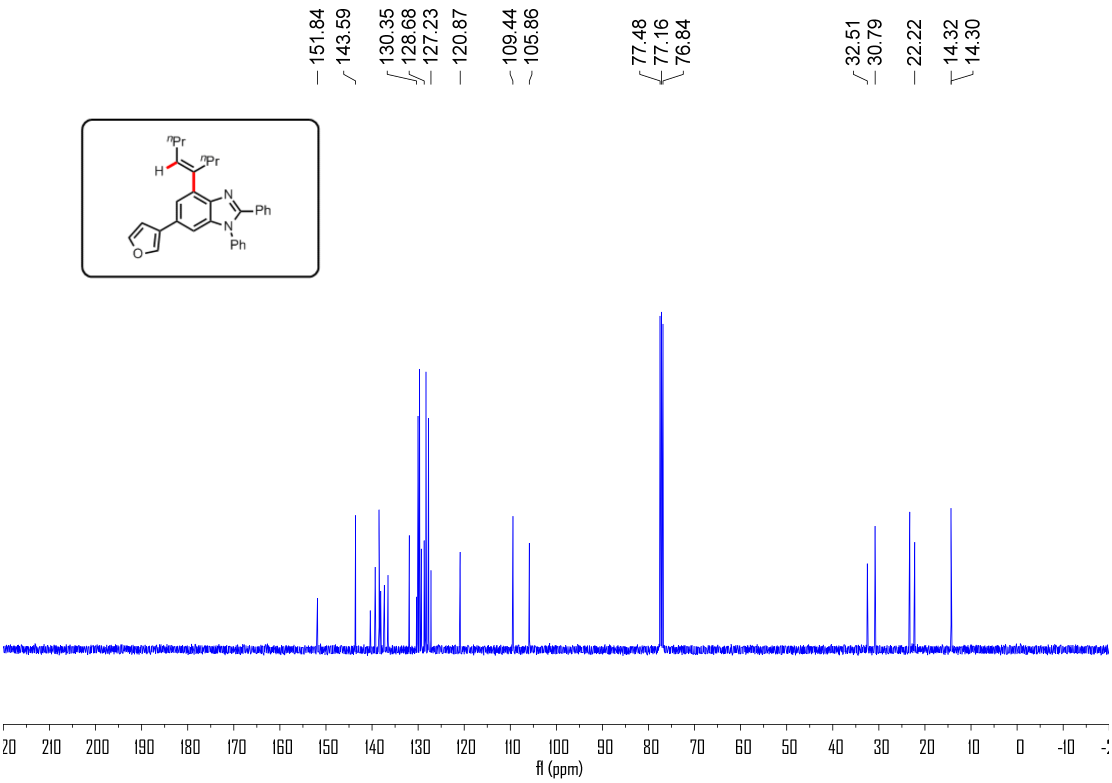

Supplementary Figure 61. ^{1}H (**3f**) and ^{13}C (**3f**) NMR spectra in $CDCl_3$.

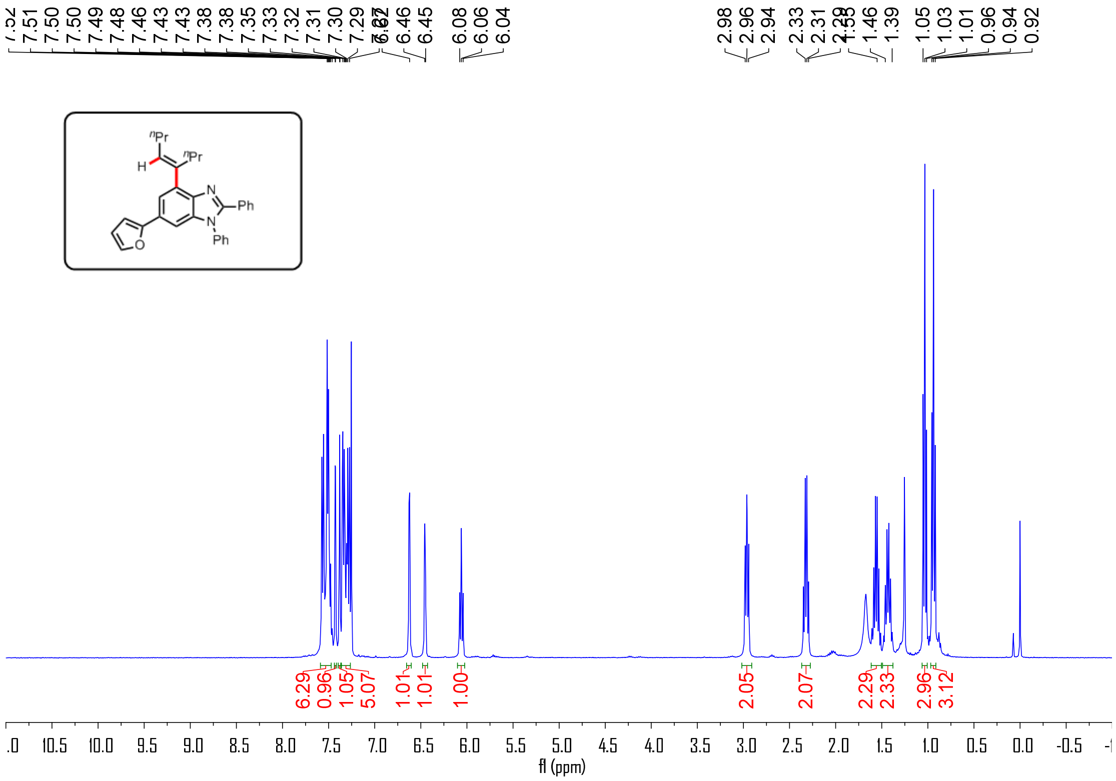

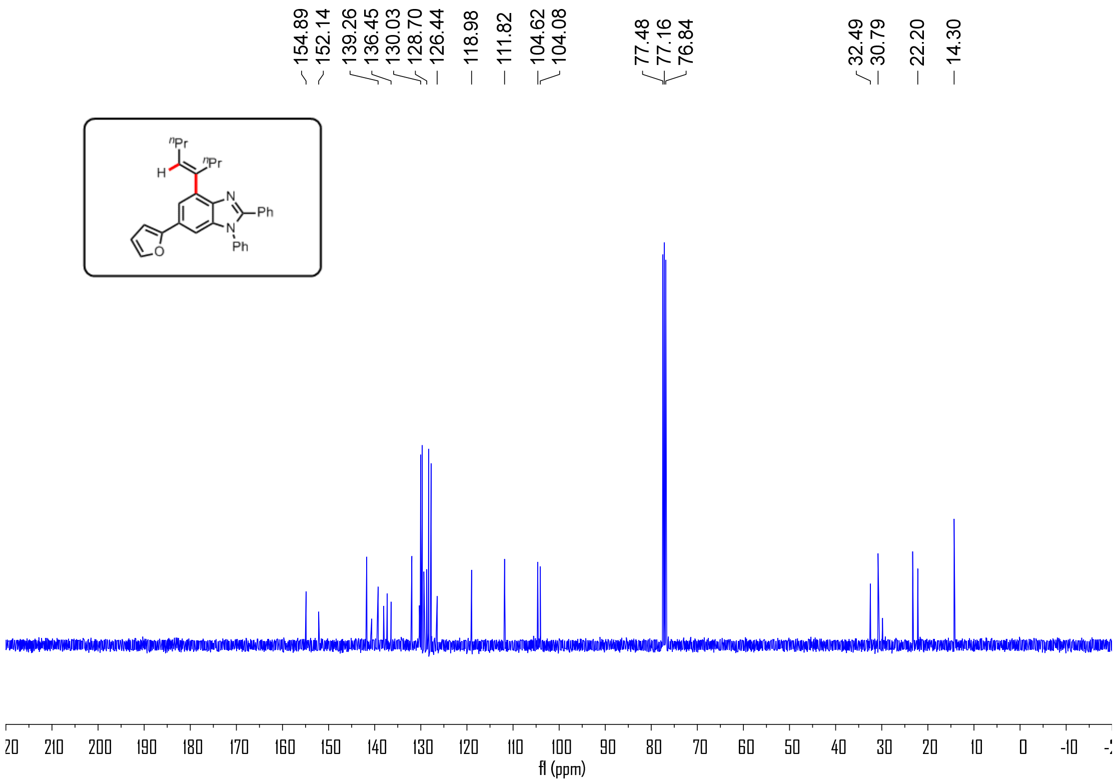

Supplementary Figure 62. ^{1}H (**3h**) and ^{13}C (**3h**) NMR spectra in $CDCl_3$.

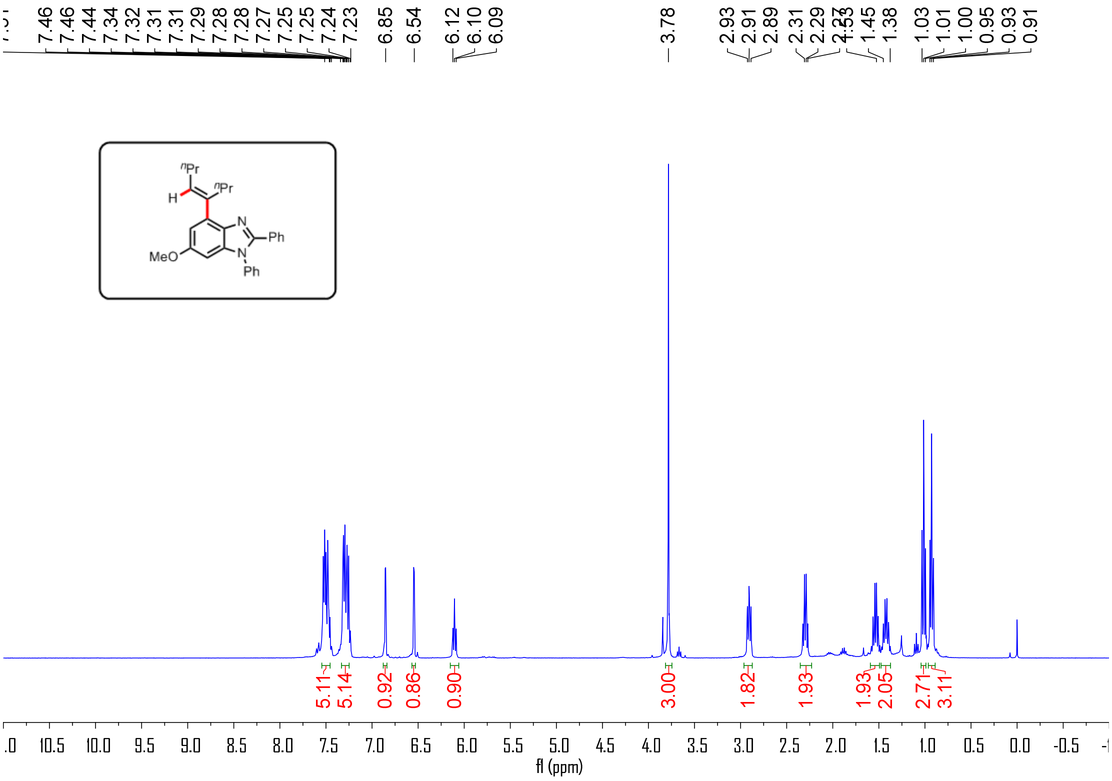

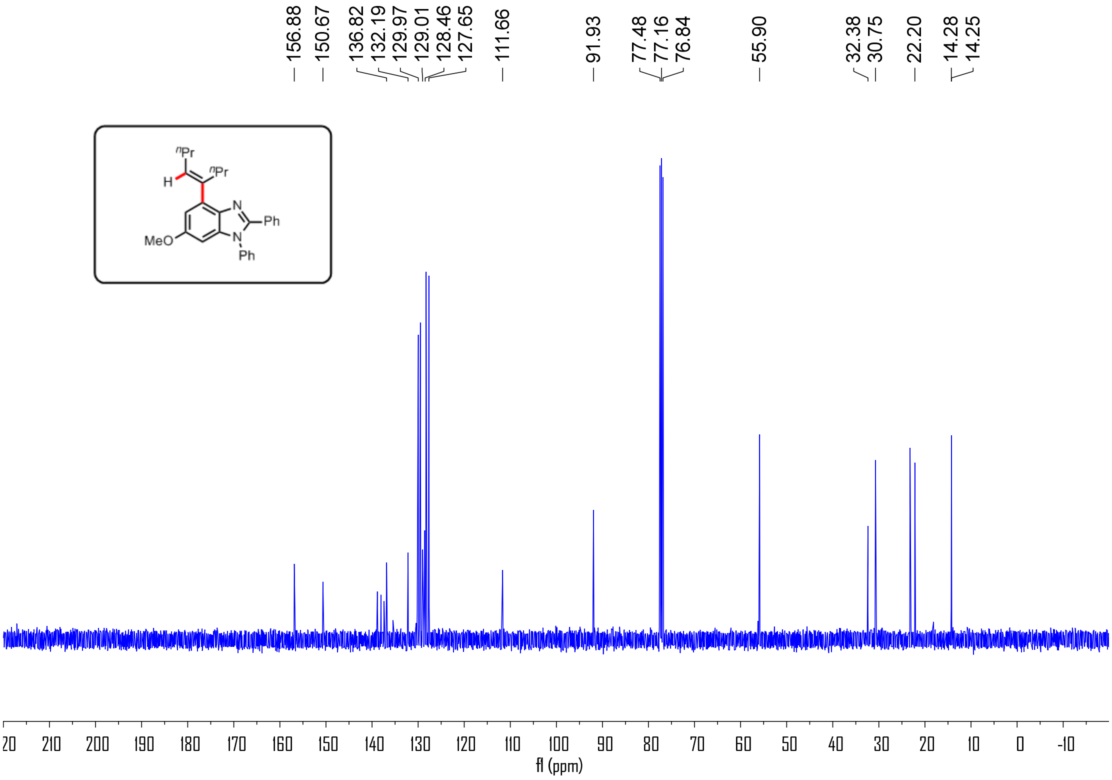

Supplementary Figure 63. ^{1}H (**3i**) and ^{13}C (**3i**) NMR spectra in $CDCl_3$.

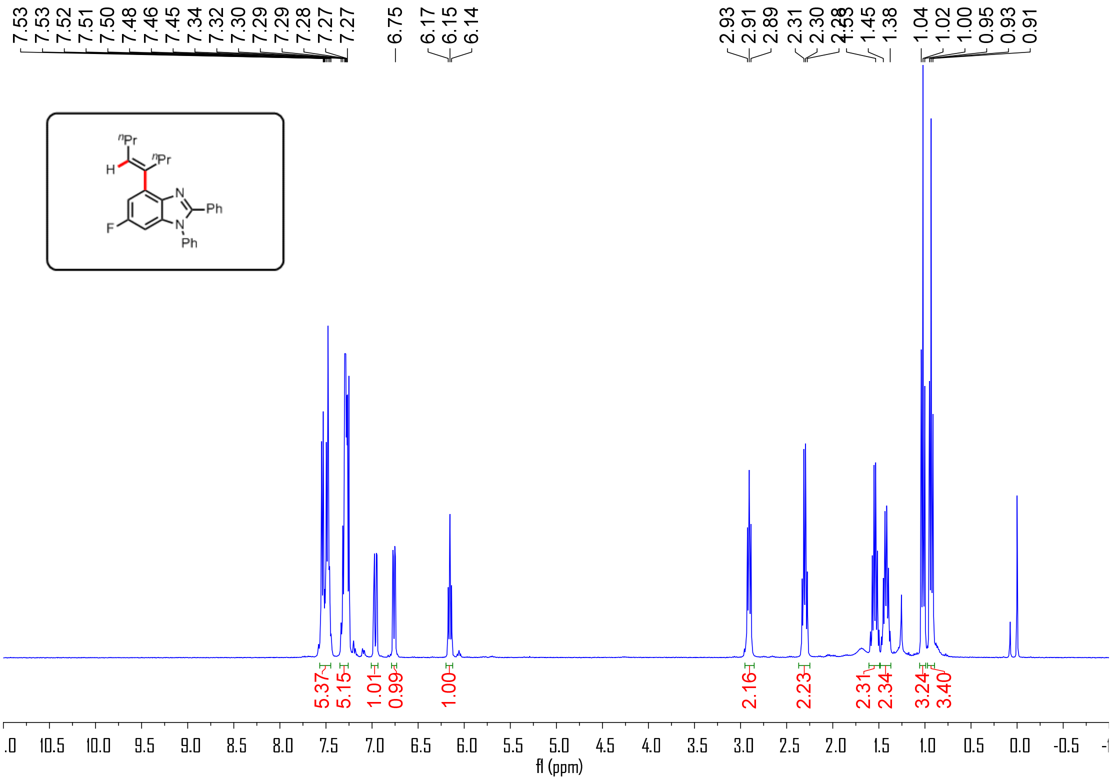

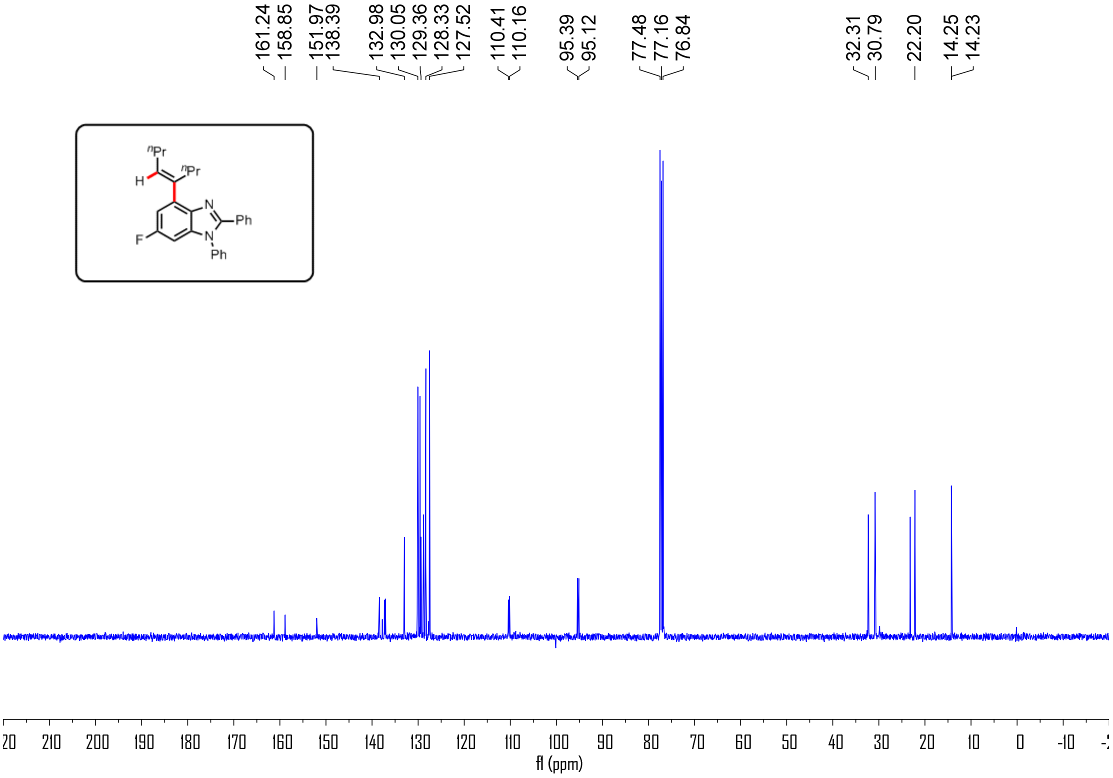

Supplementary Figure 64. ^{1}H (**3j**) and ^{13}C (**3j**) NMR spectra in $CDCl_3$.

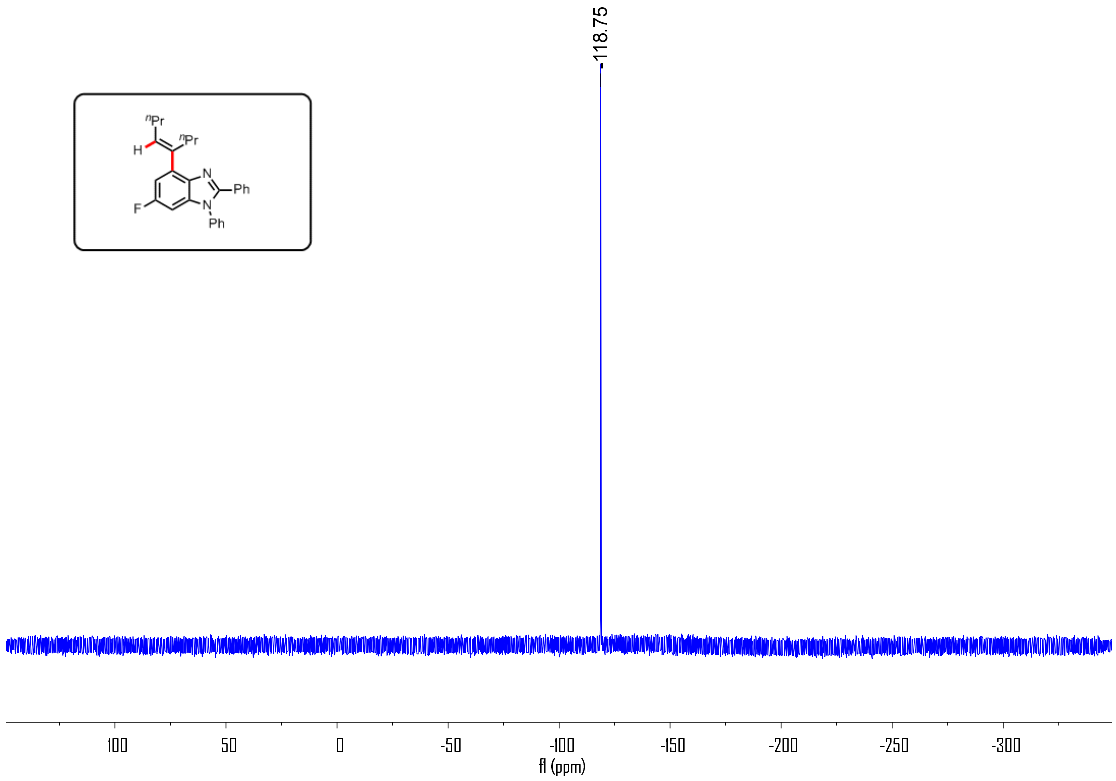

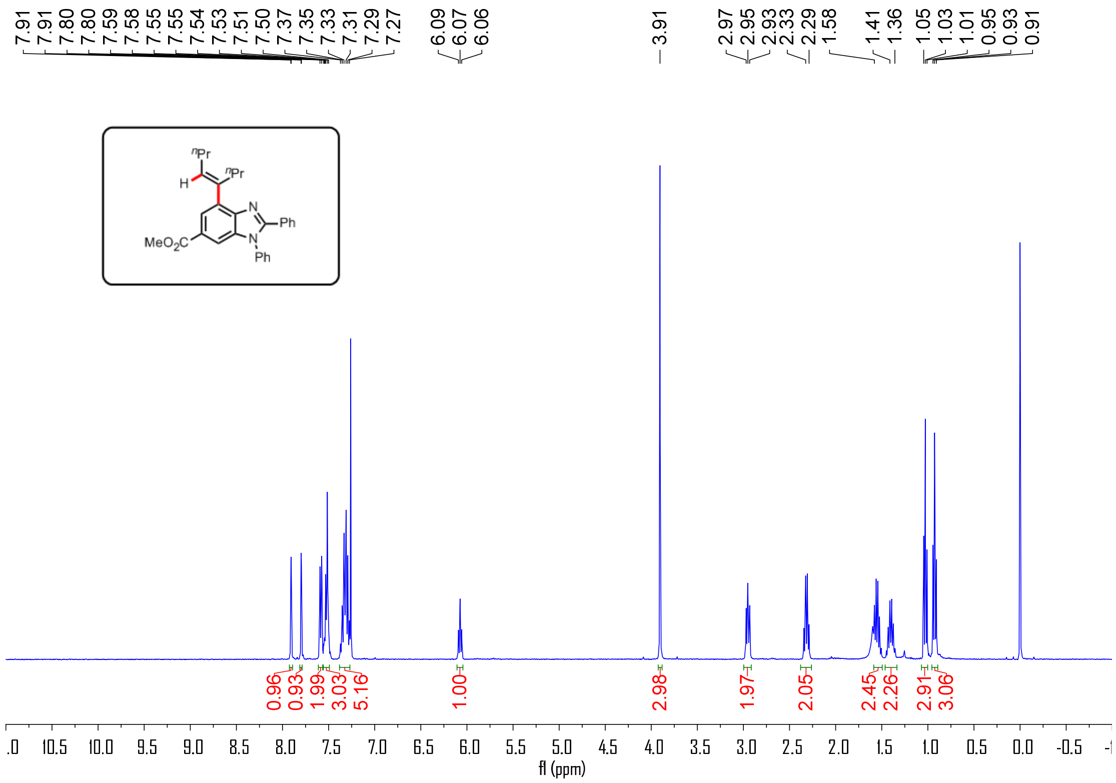

Supplementary Figure 65. ^{19}F (**3j**) and ^{1}H (**3k**) NMR spectra in $CDCl_3$.

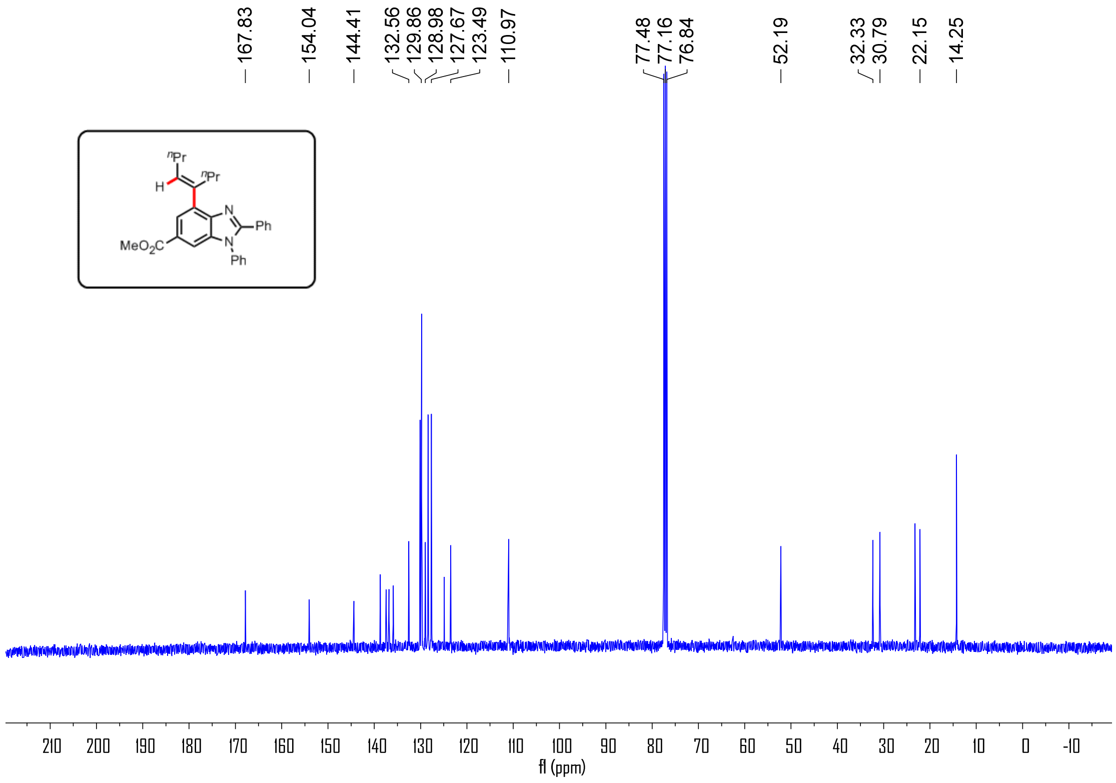

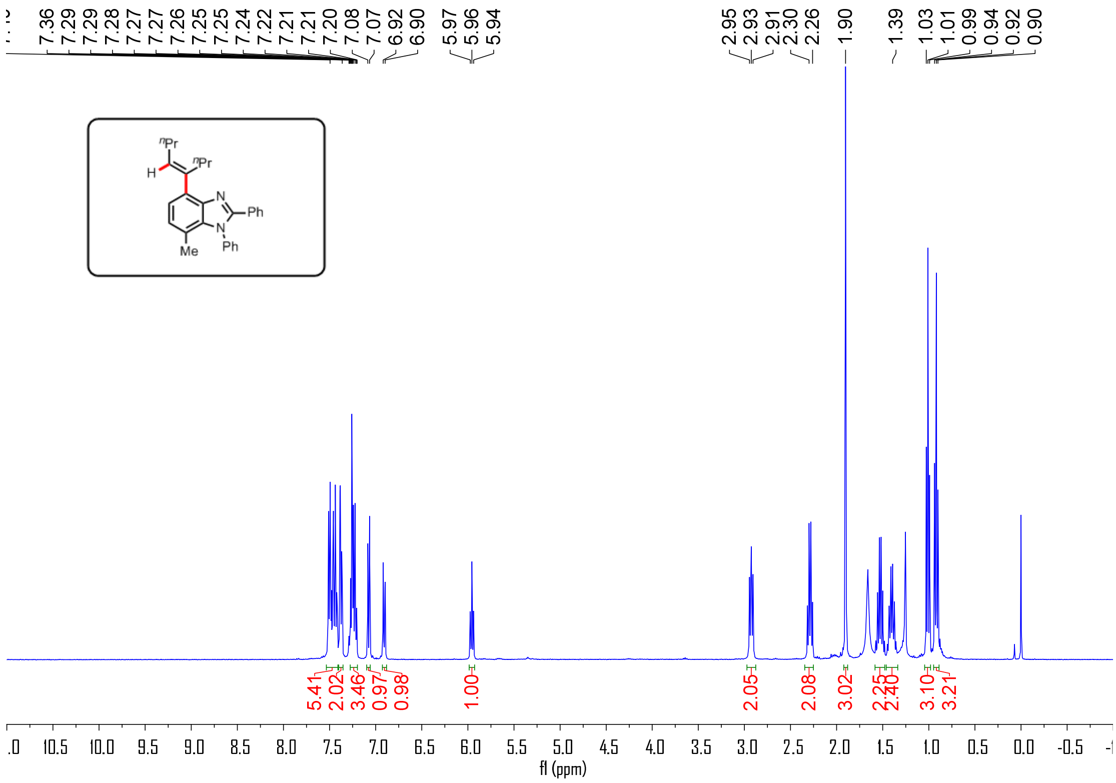

Supplementary Figure 66. ^{13}C (**3k**) and ^{1}H (**3l**) NMR spectra in $CDCl_3$.

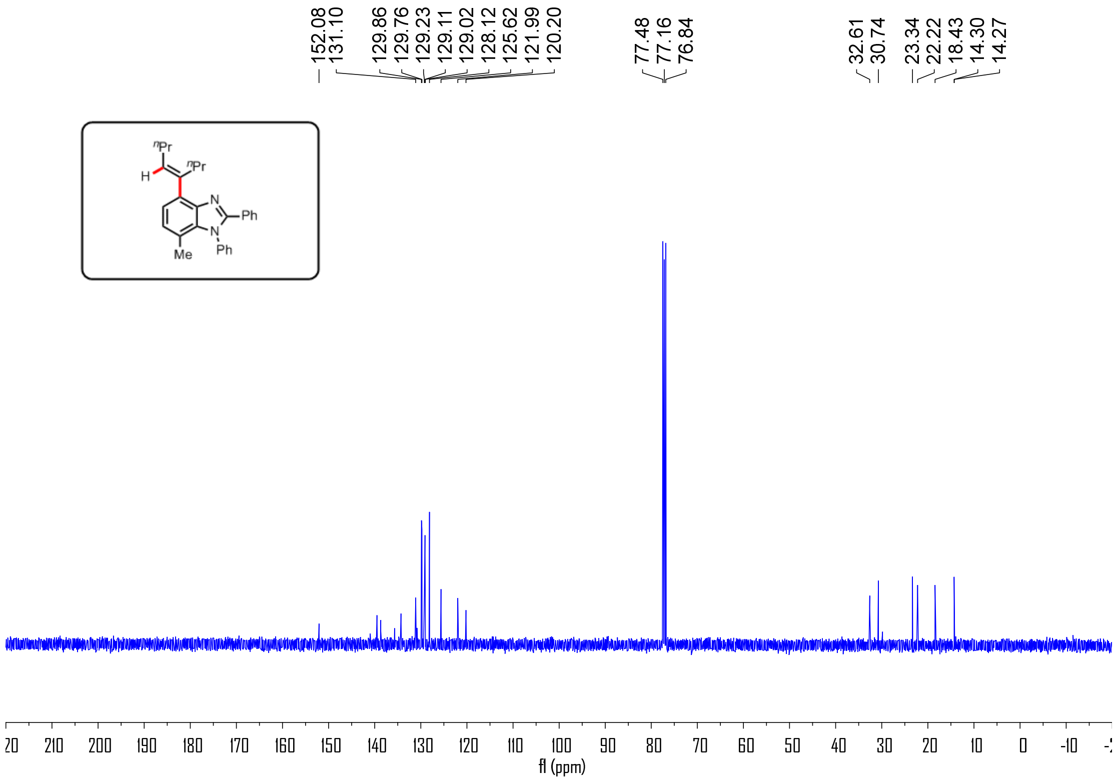

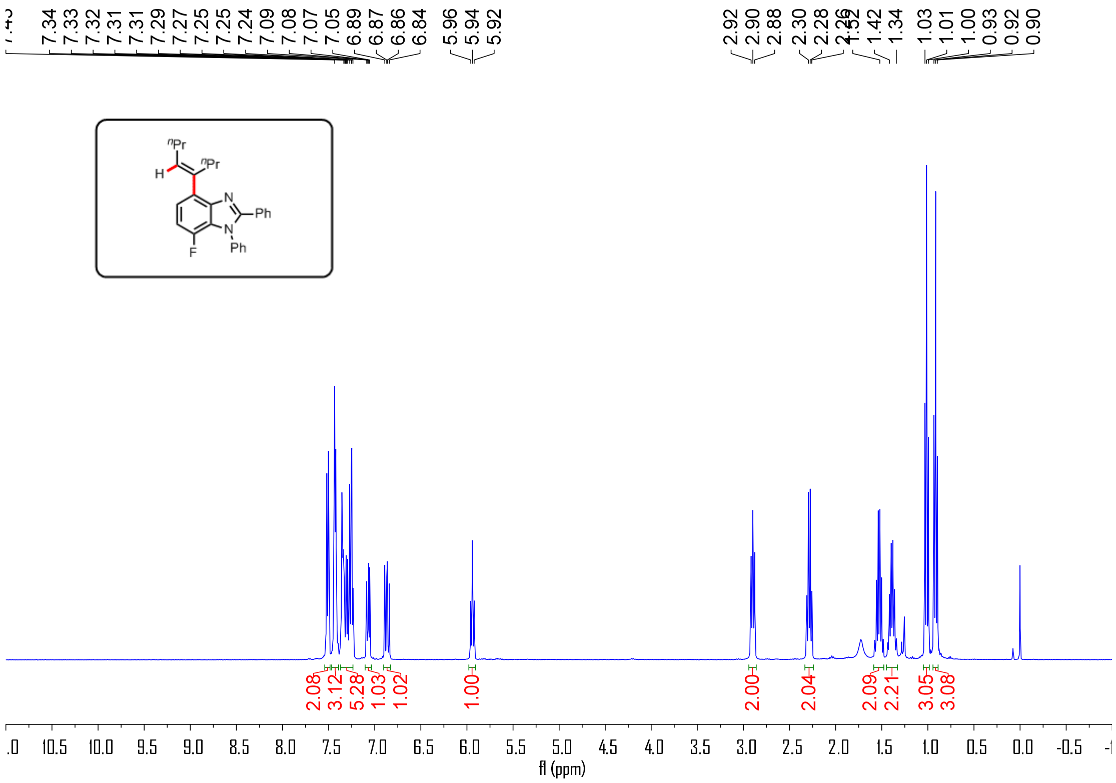

Supplementary Figure 67. ^{13}C (**3l**) and ^{1}H (**3m**) NMR spectra in $CDCl_3$.

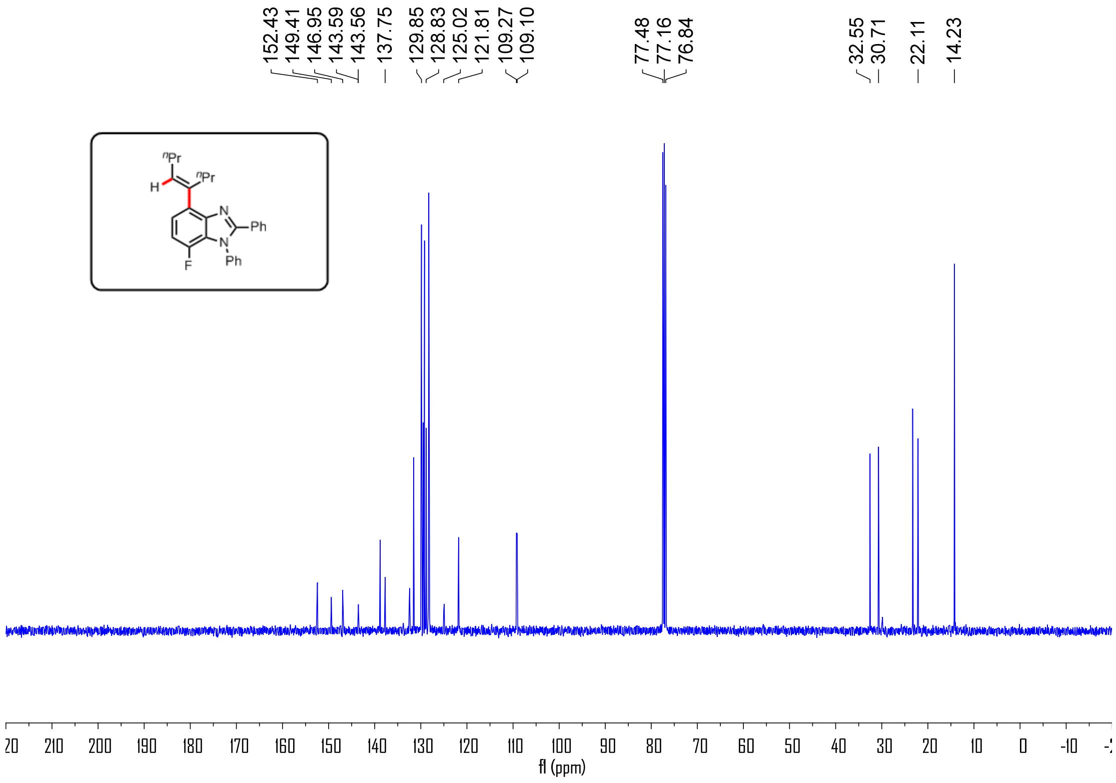

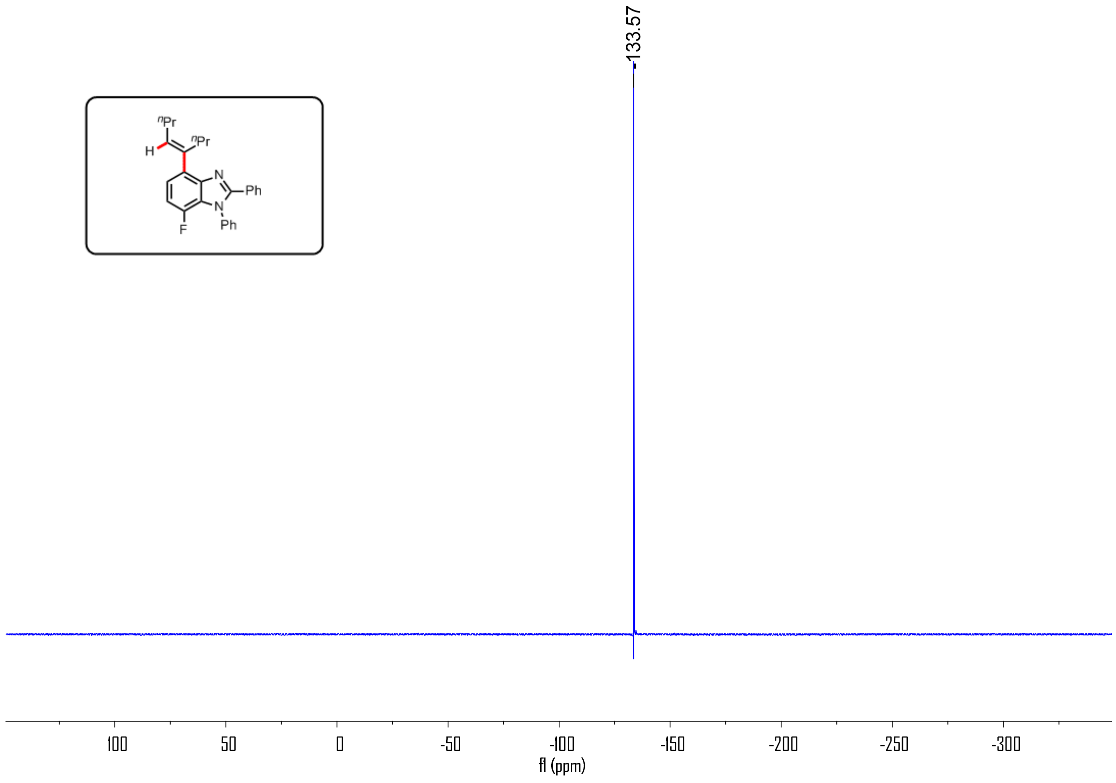

Supplementary Figure 68. ^{13}C (**3m**) and ^{19}F (**3m**) NMR spectra in $CDCl_3$.

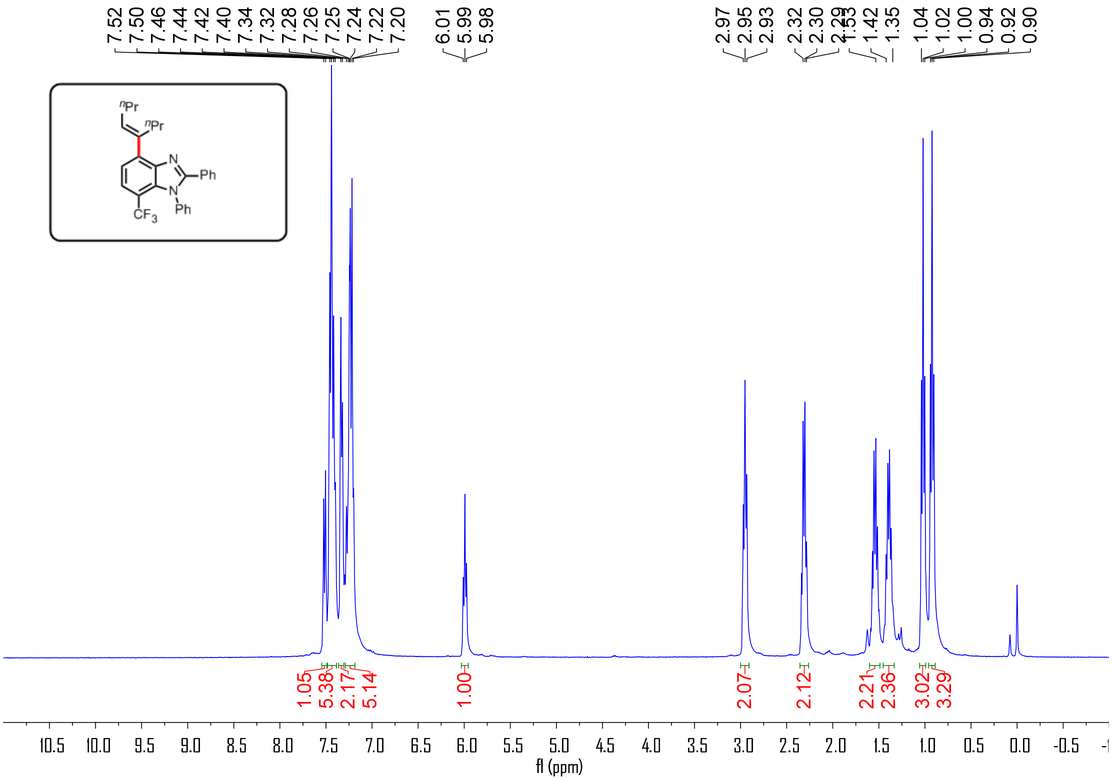

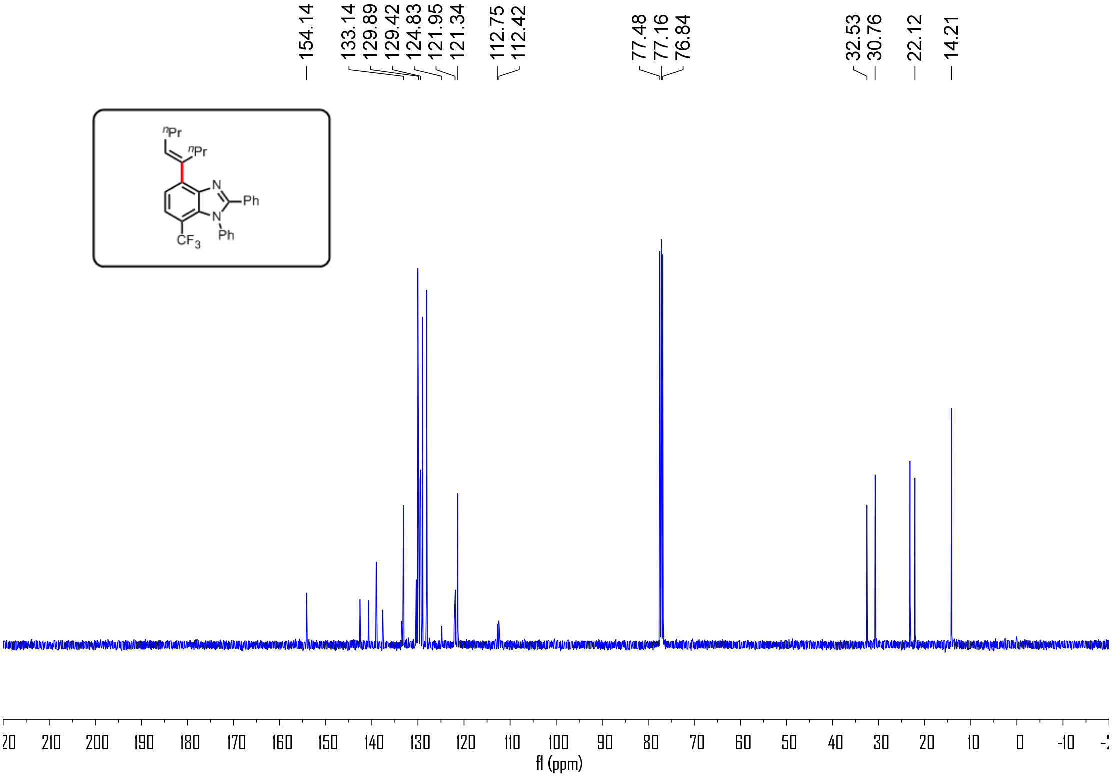

Supplementary Figure 69. ^{1}H (**3n**) and ^{13}C (**3n**) NMR spectra in $CDCl_3$.

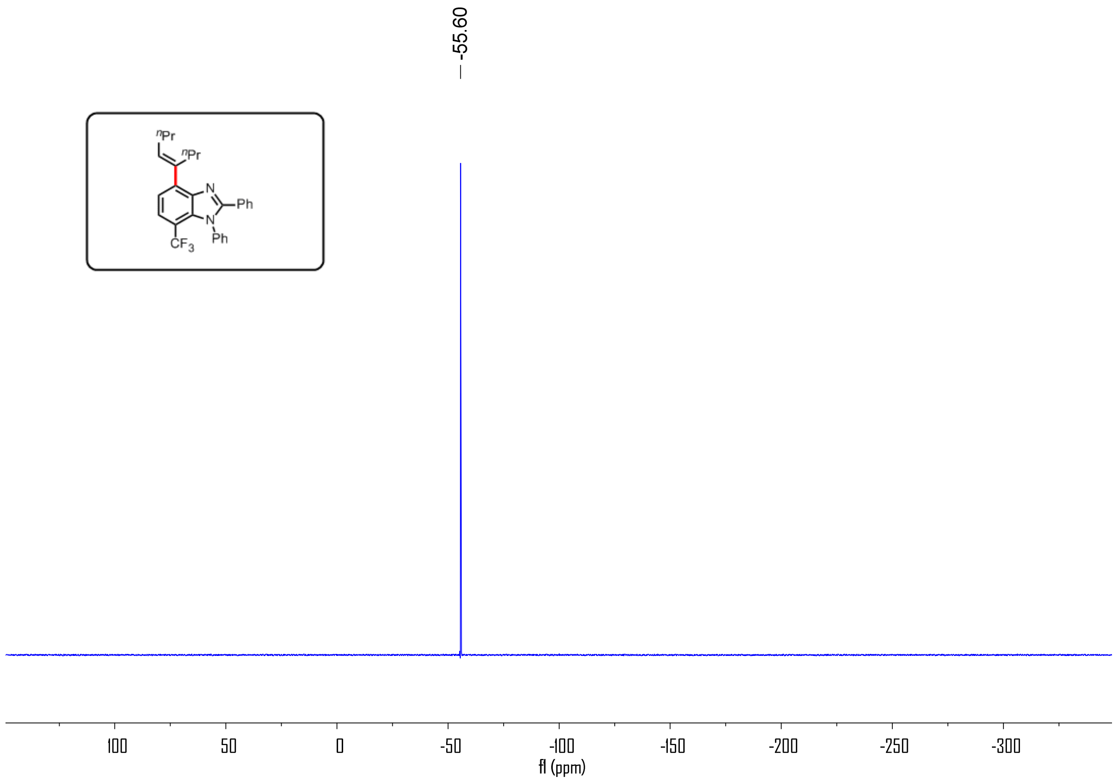

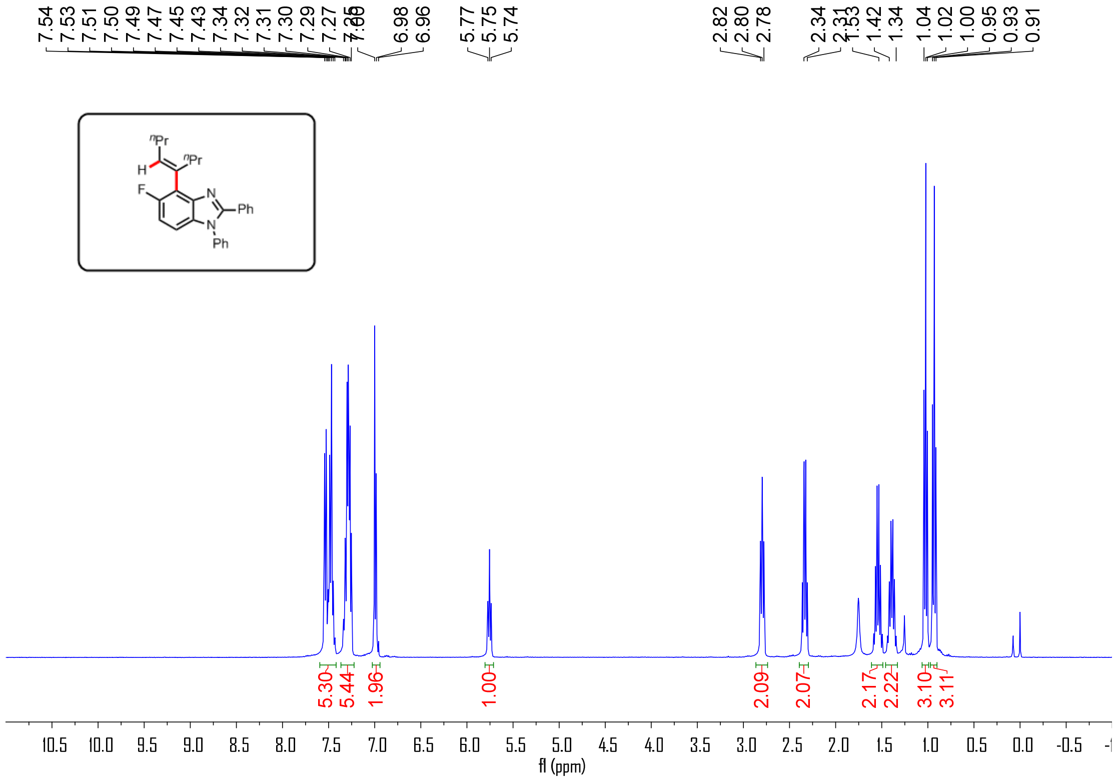

Supplementary Figure 70. ^{19}F (**3n**) and ^{1}H (**3o**) NMR spectra in $CDCl_3$.

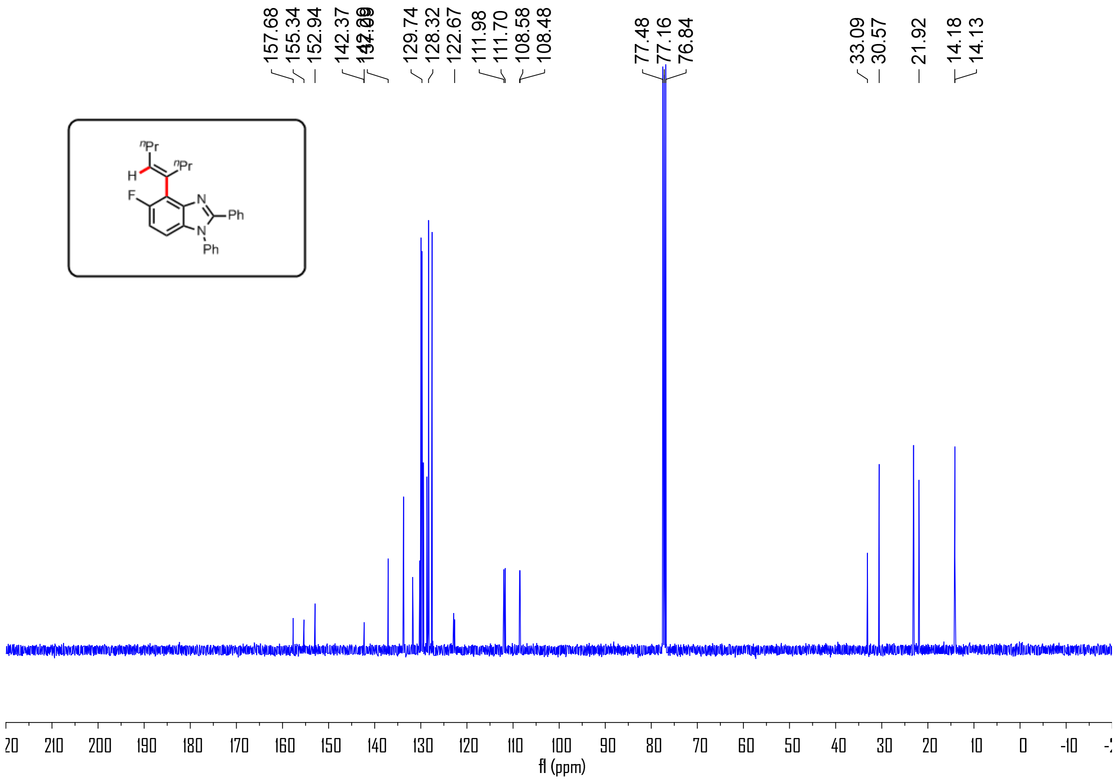

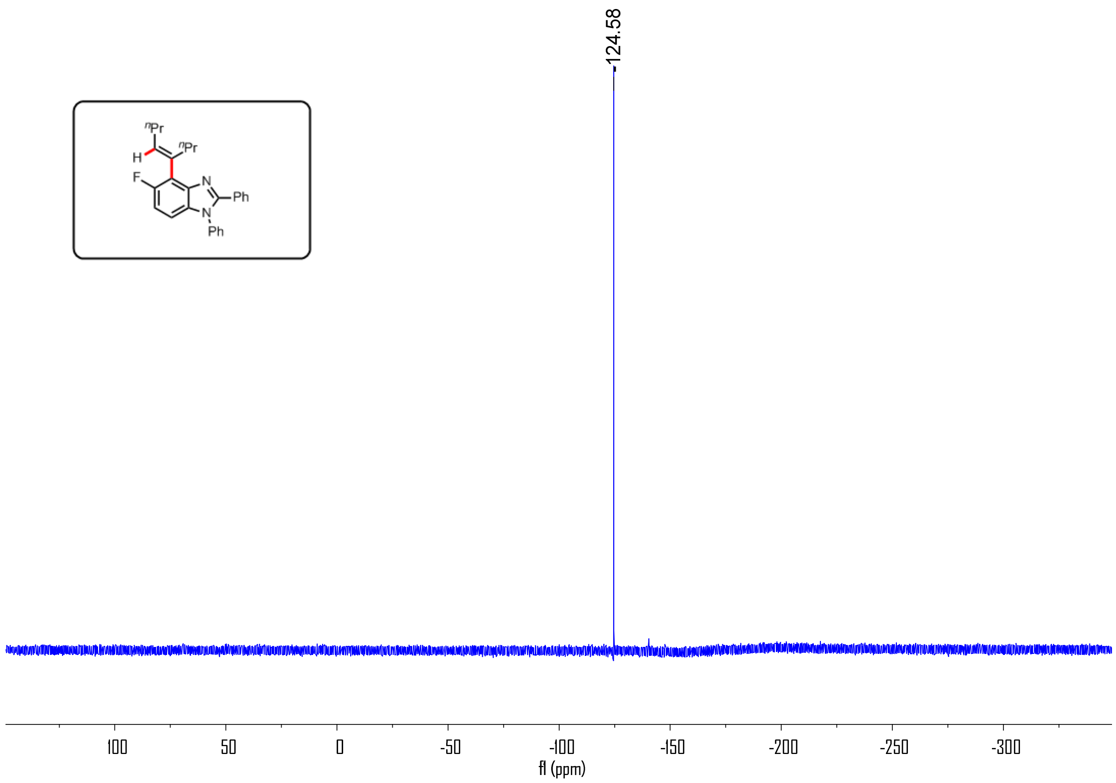

Supplementary Figure 71. ^{13}C (3o) and ^{19}F (3o) NMR spectra in $CDCl_3$.

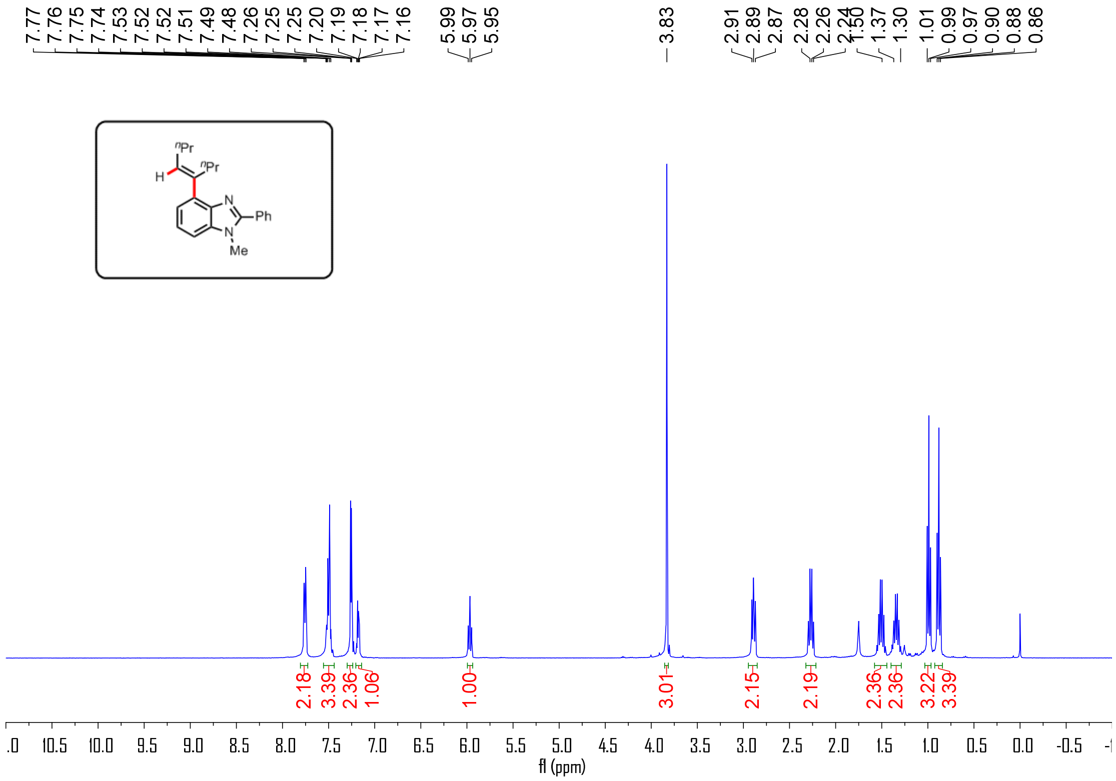

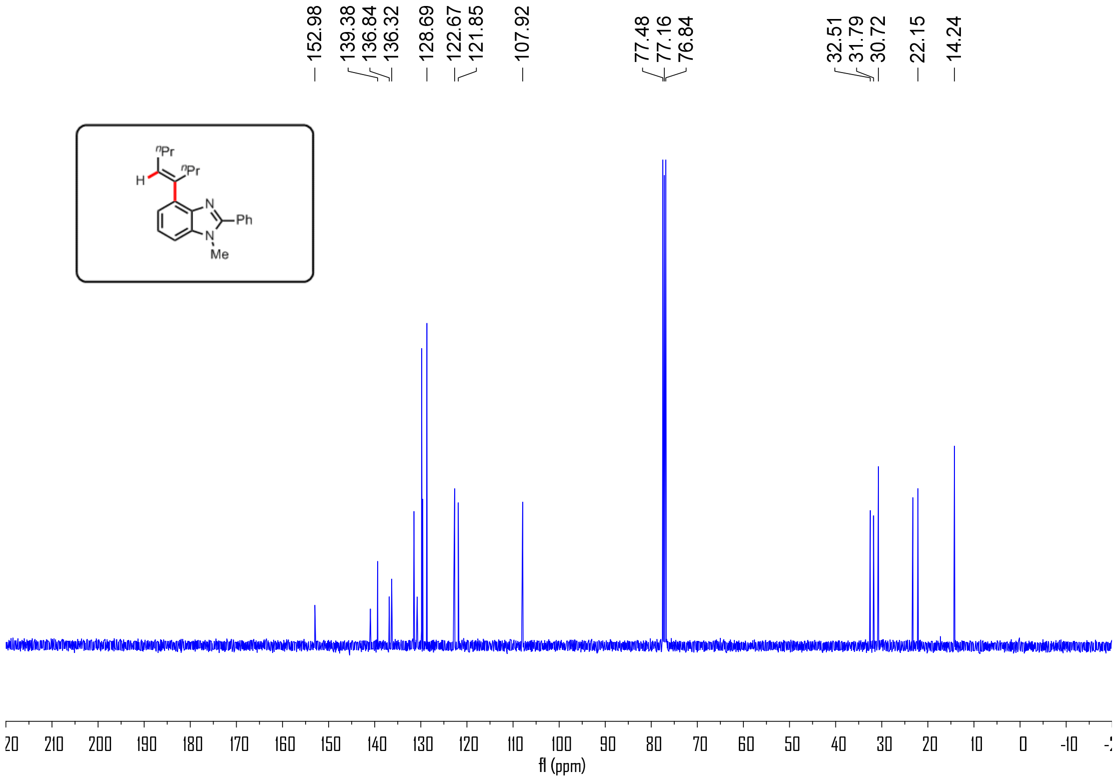

Supplementary Figure 72. ^{1}H (**3p**) and ^{13}C (**3p**) NMR spectra in $CDCl_3$.

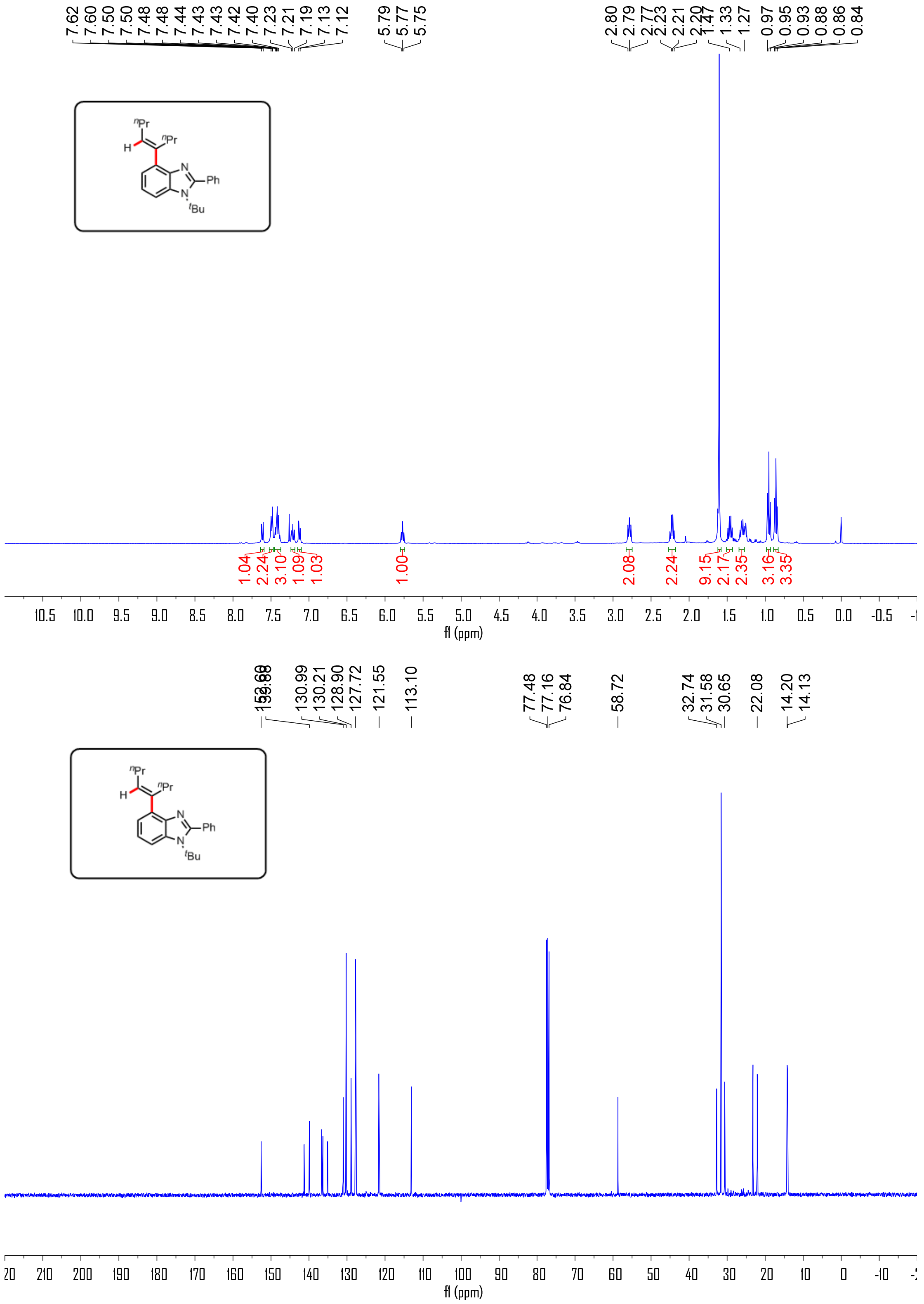

Supplementary Figure 73. ^{1}H (**3q**) and ^{13}C (**3q**) NMR spectra in $CDCl_3$.

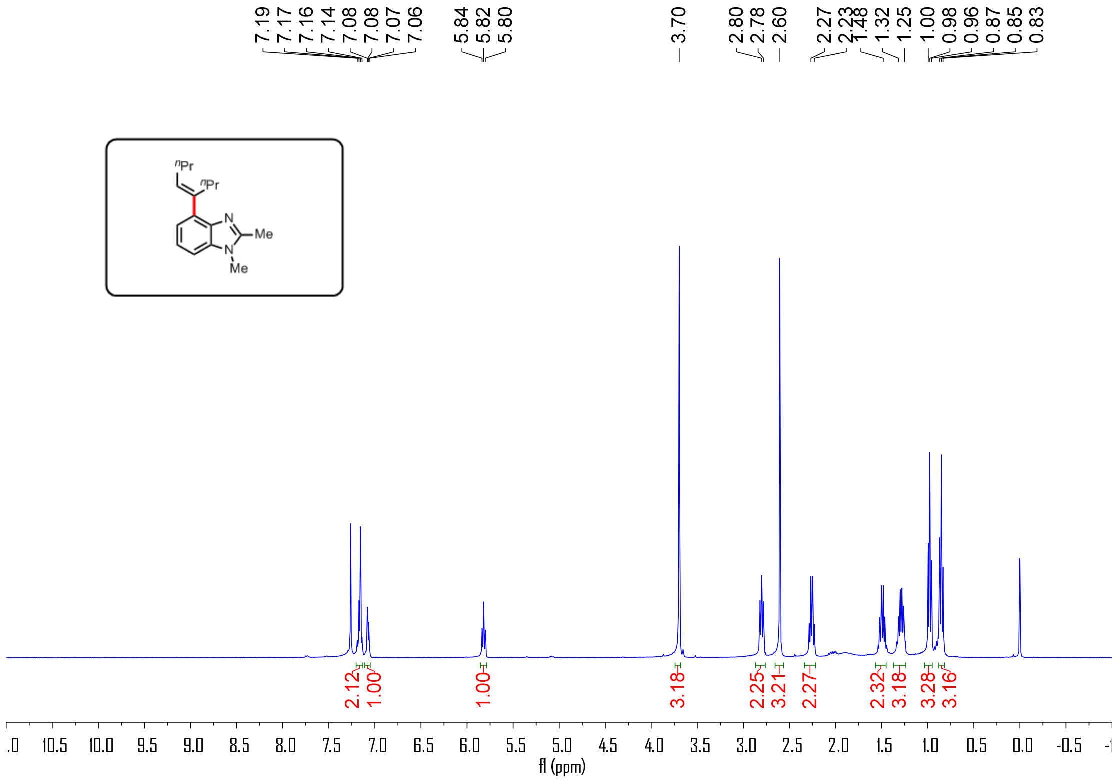

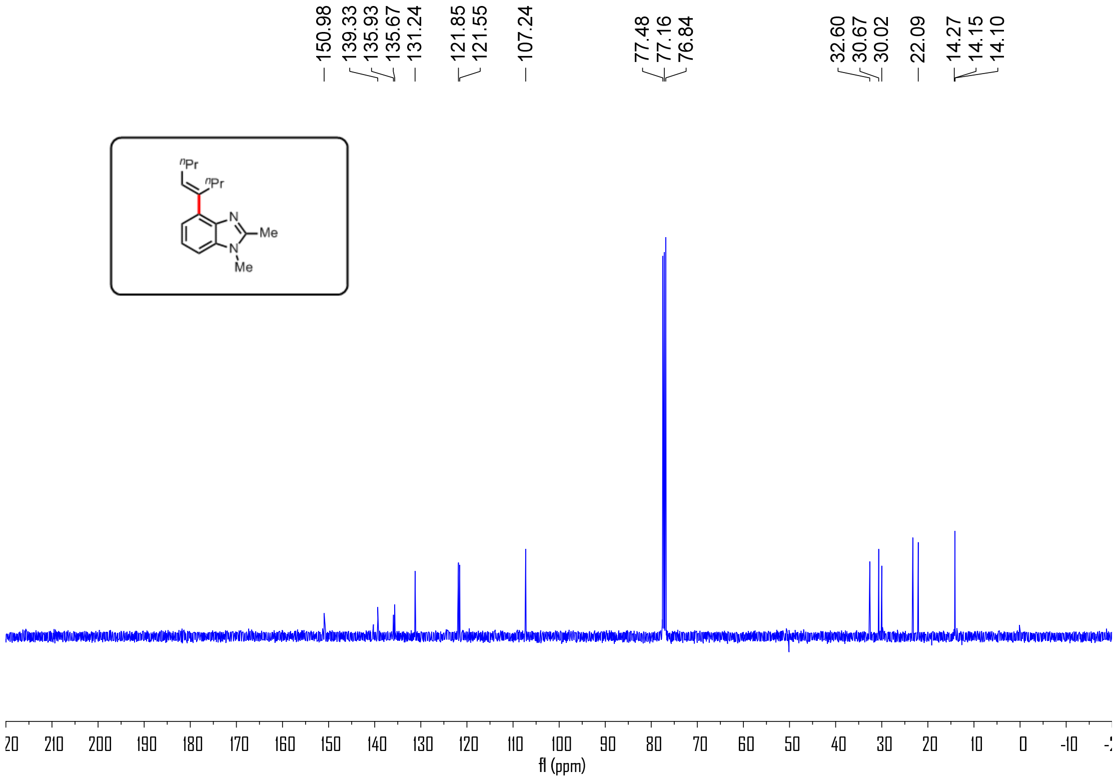

Supplementary Figure 74. ^{1}H (**3r**) and ^{13}C (**3r**) NMR spectra in $CDCl_3$.

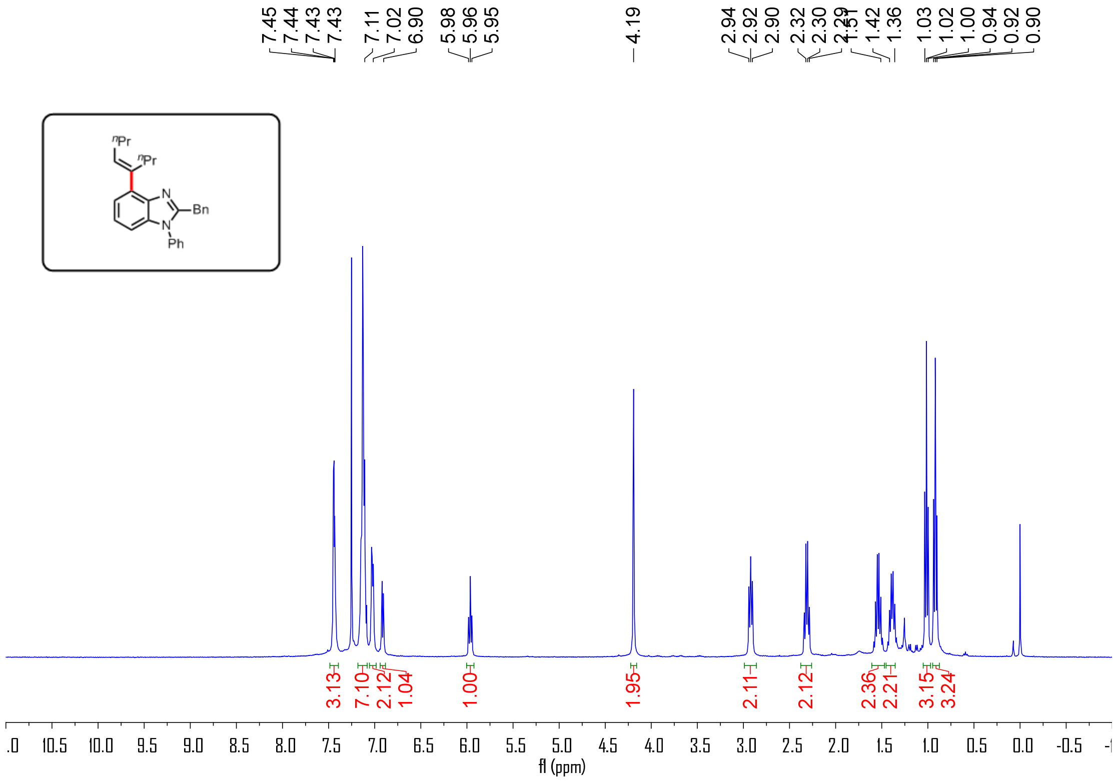

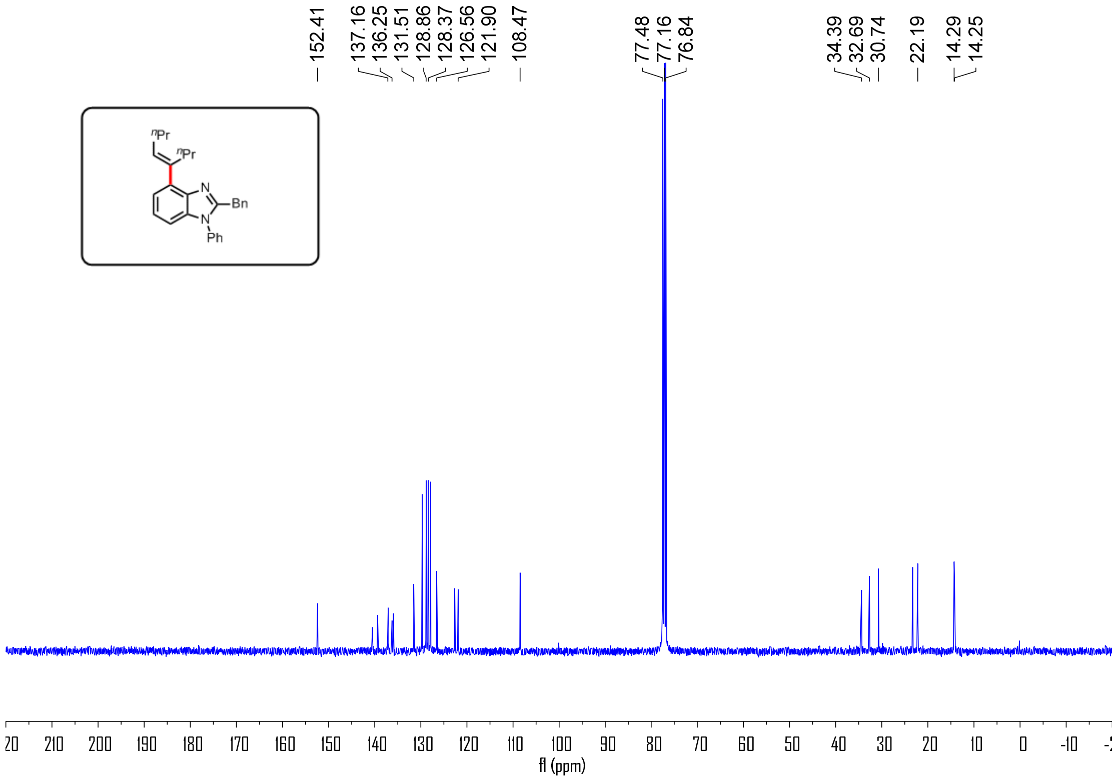

Supplementary Figure 75. ^{1}H (**3s**) and ^{13}C (**3s**) NMR spectra in $CDCl_3$.

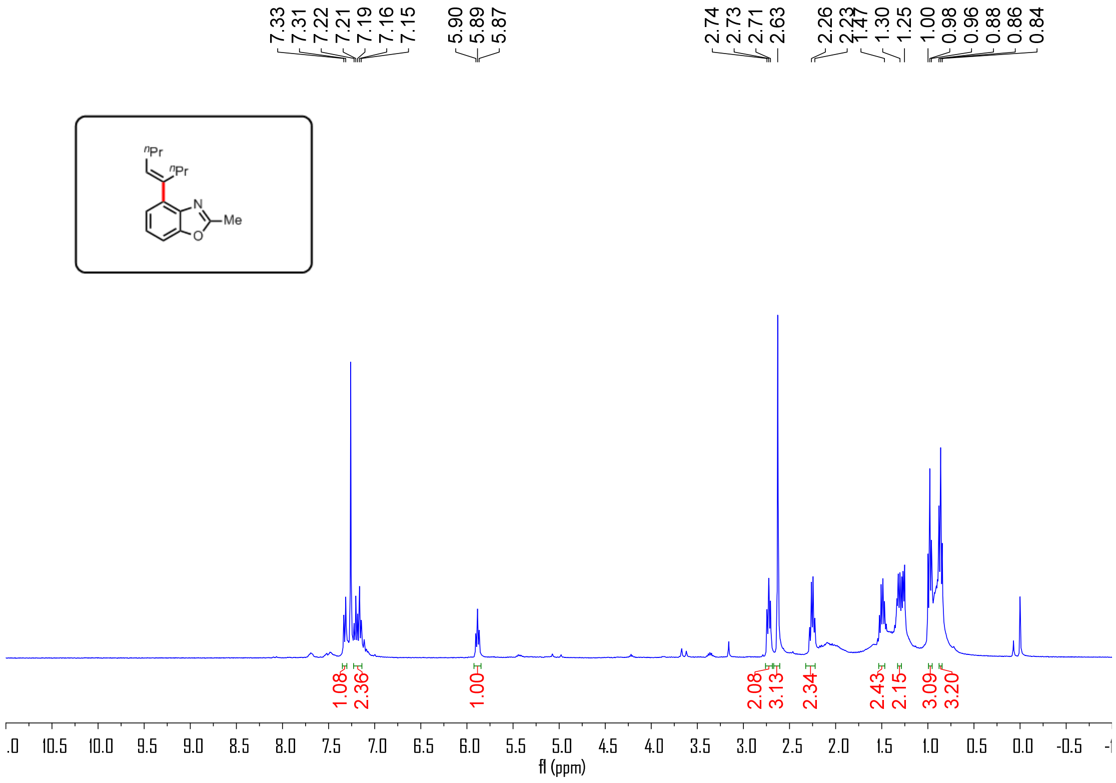

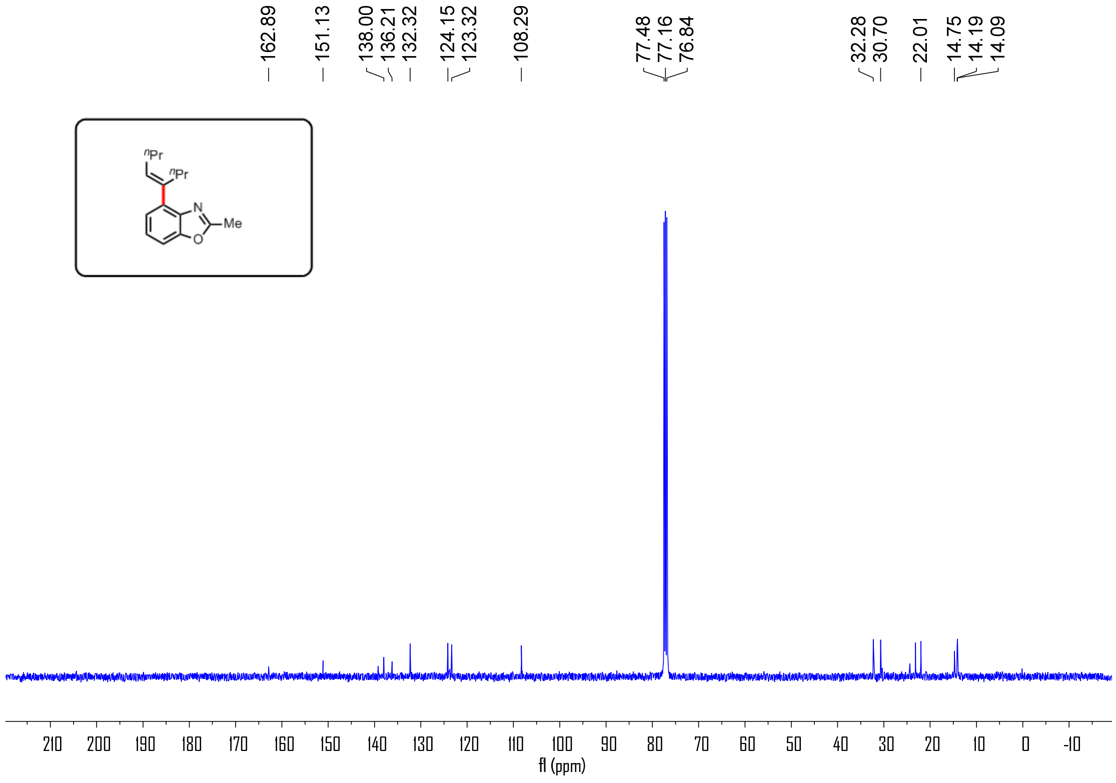

Supplementary Figure 76. ^1H (**3t**) and ^{13}C (**3t**) NMR spectra in $CDCl_3$.

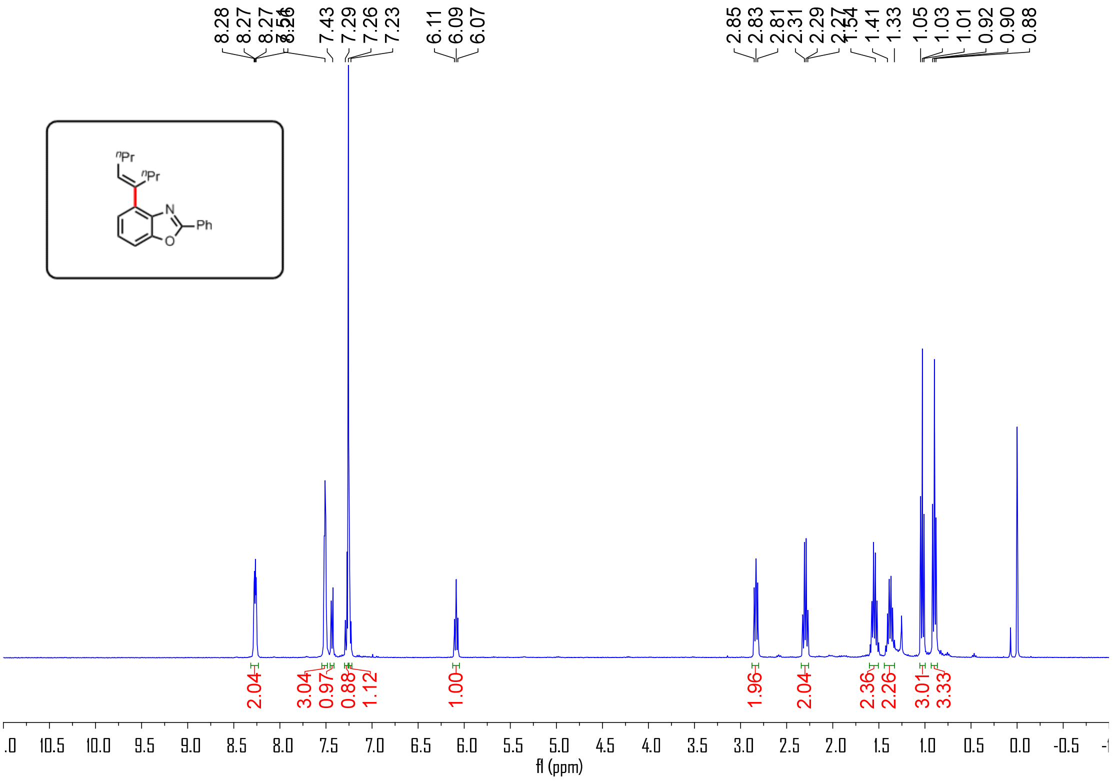

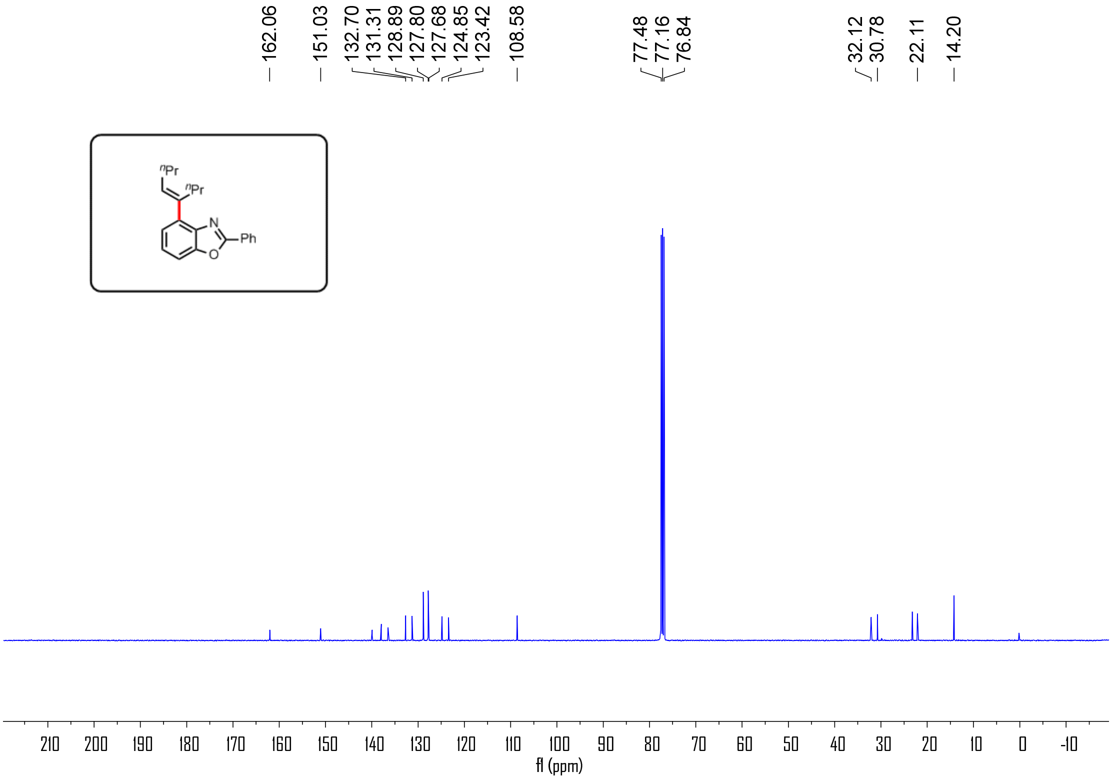

Supplementary Figure 77. ^{1}H (**3u**) and ^{13}C (**3u**) NMR spectra in $CDCl_3$.

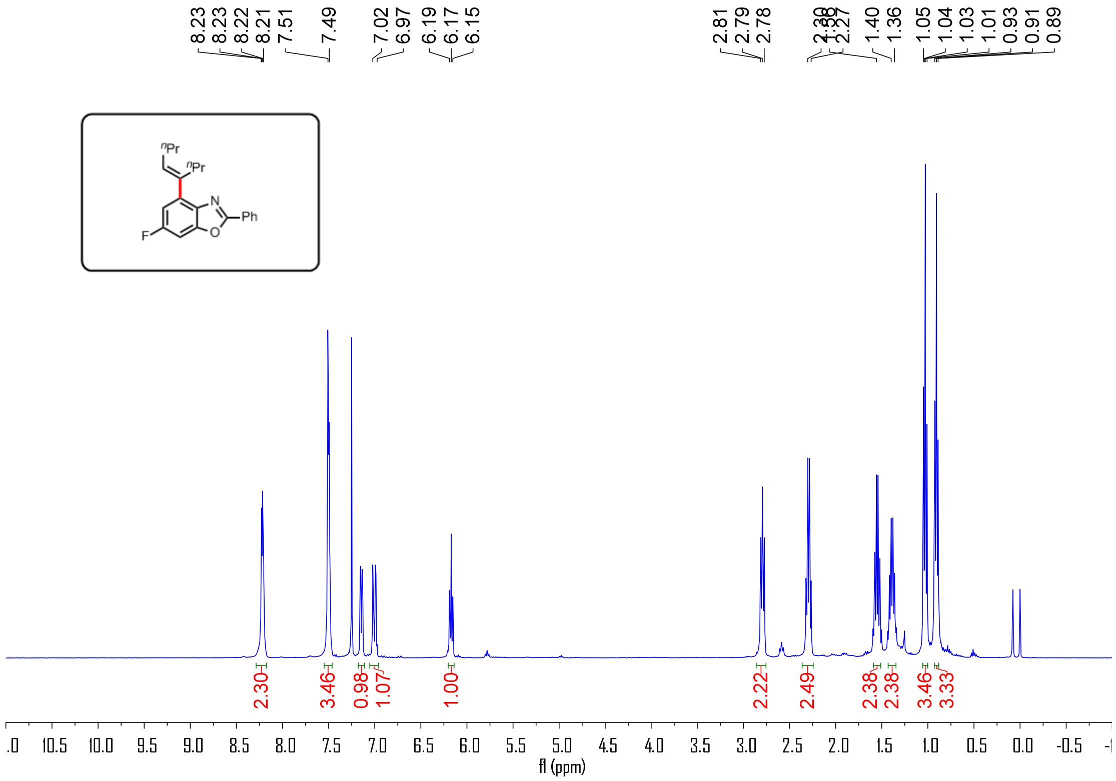

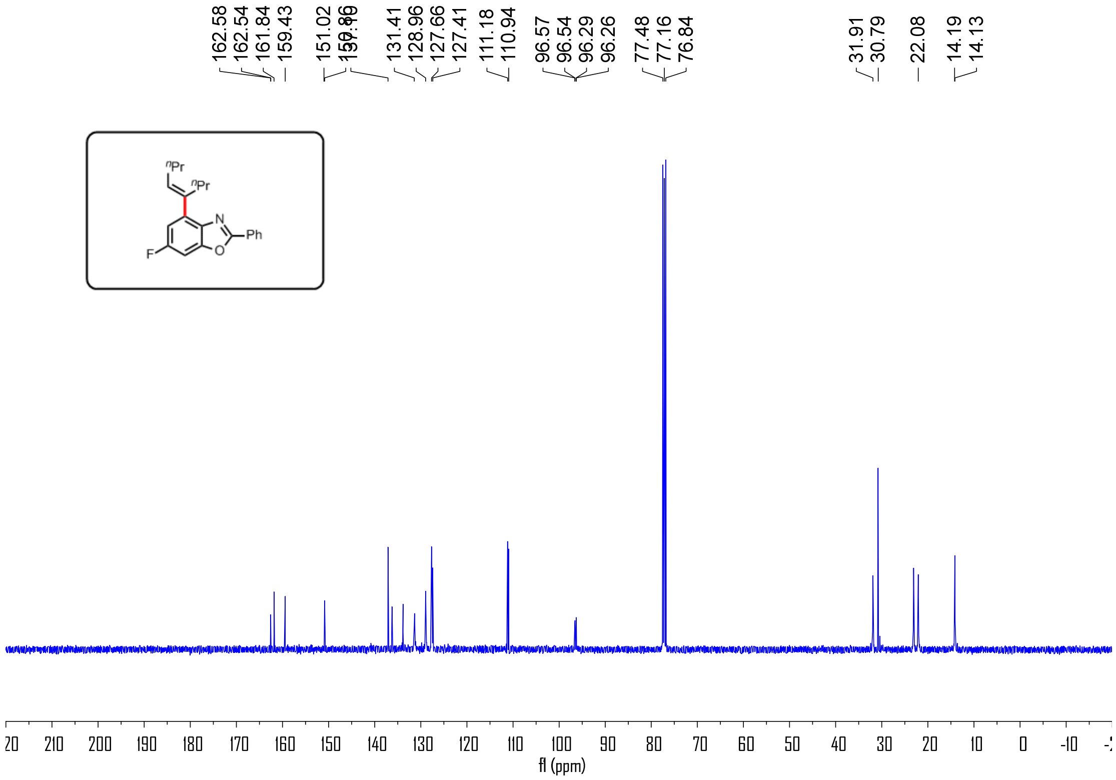

Supplementary Figure 78. ^{1}H (**3v**) and ^{13}C (**3v**) NMR spectra in $CDCl_3$.

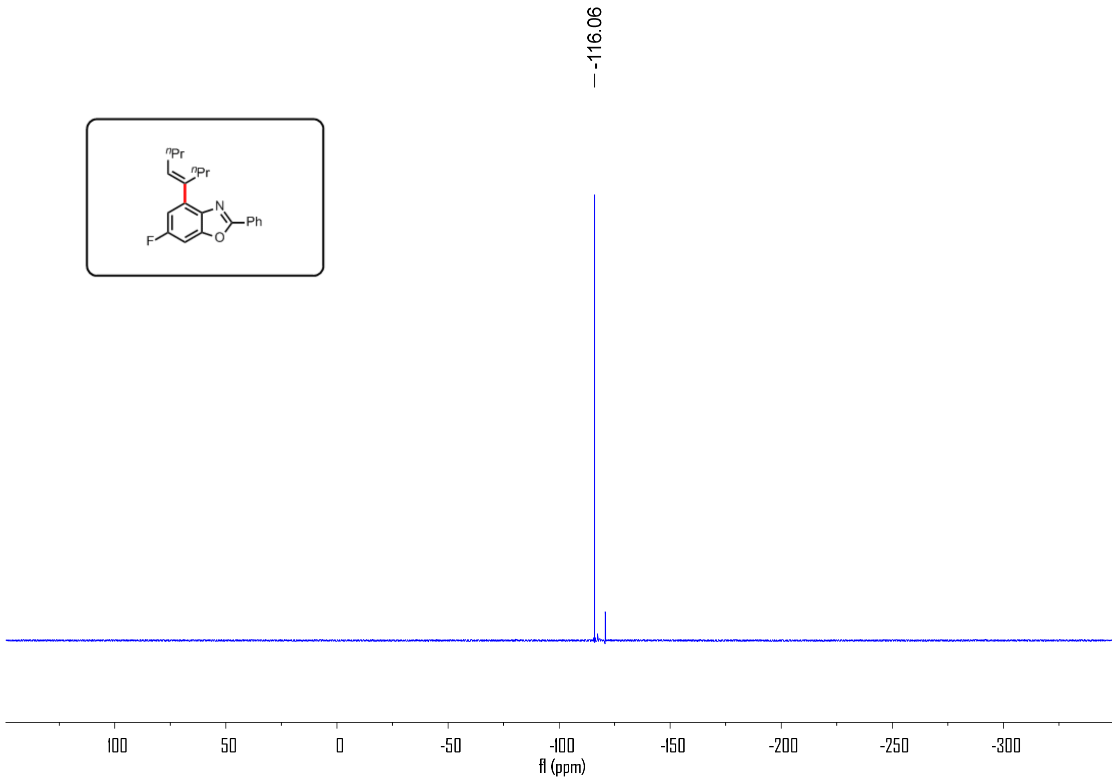

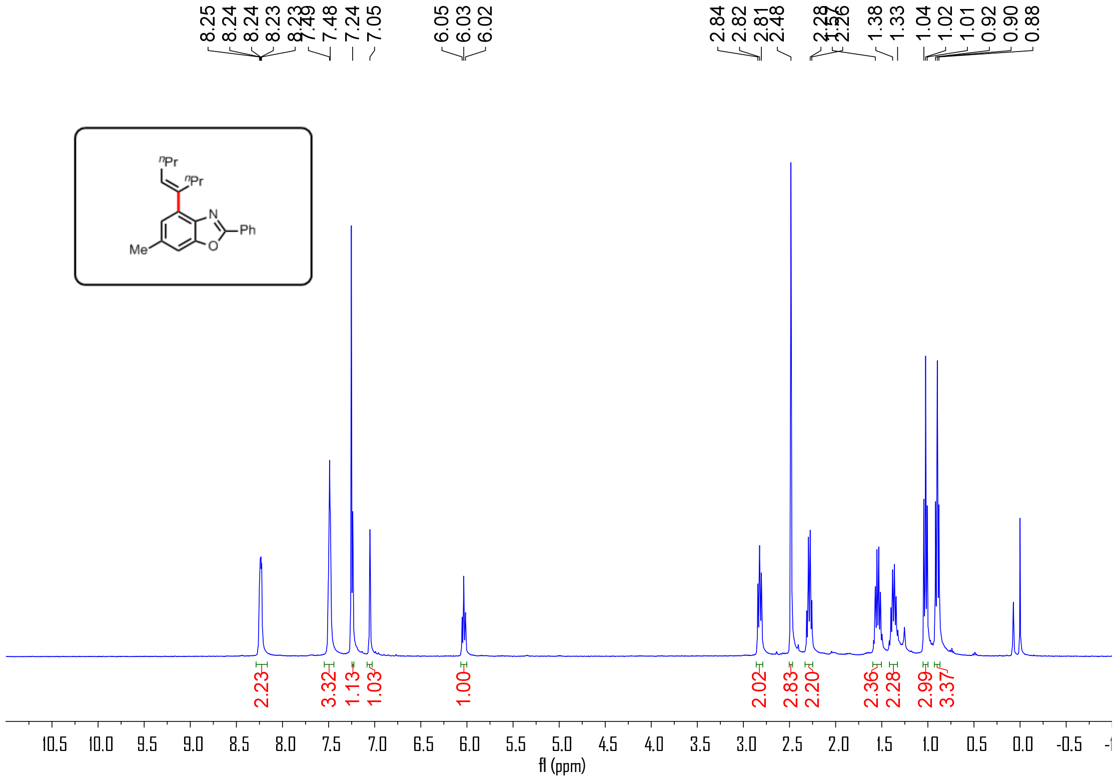

Supplementary Figure 79. ^{19}F (**3v**) and ^{1}H (**3w**) NMR spectra in $CDCl_3$.

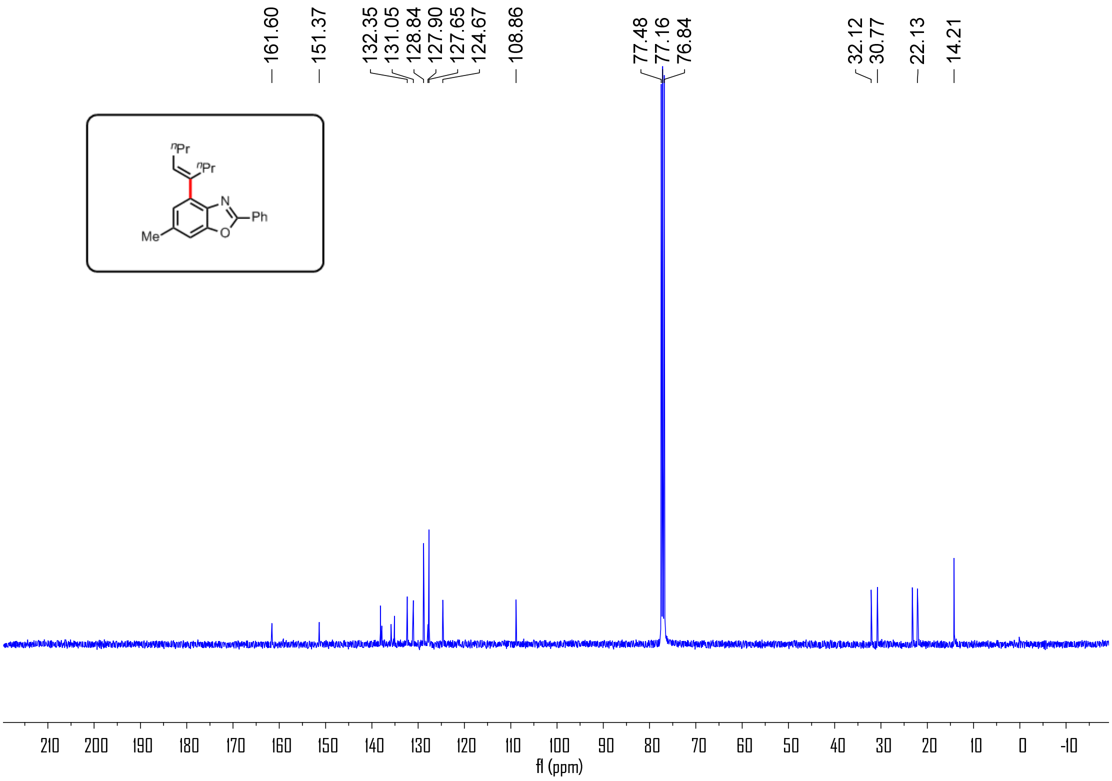

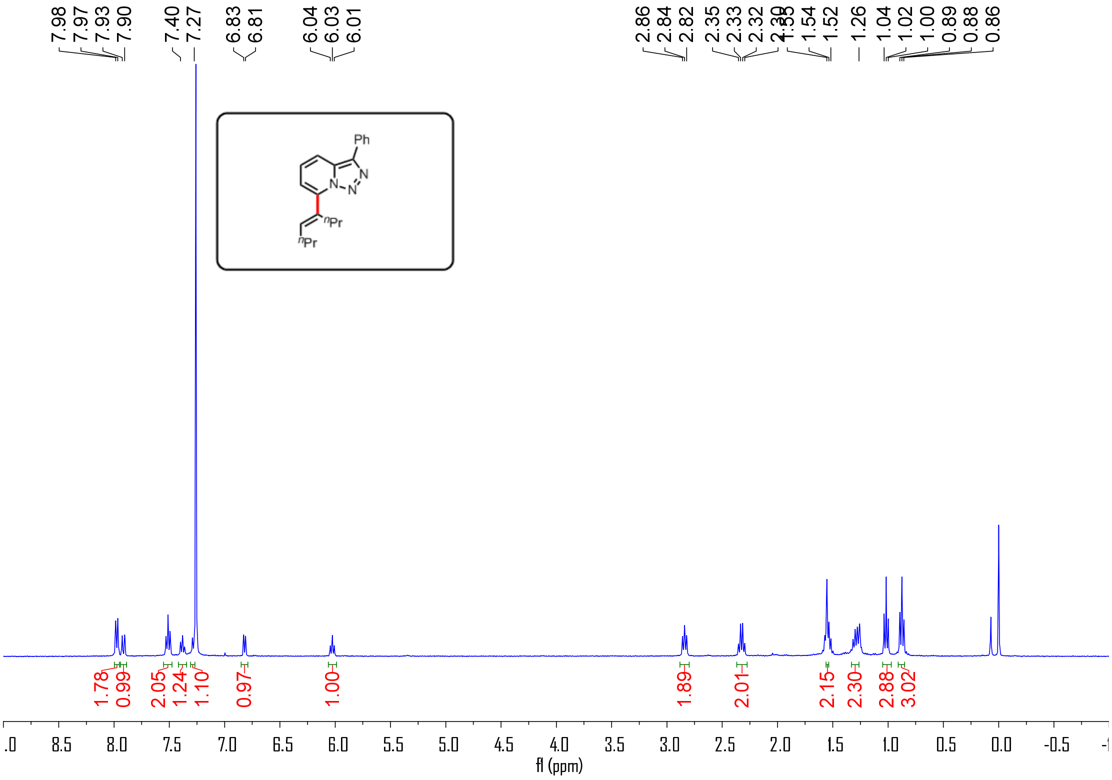

Supplementary Figure 80. ^{13}C (**3w**) and ^{1}H (**3x**) NMR spectra in $CDCl_3$.

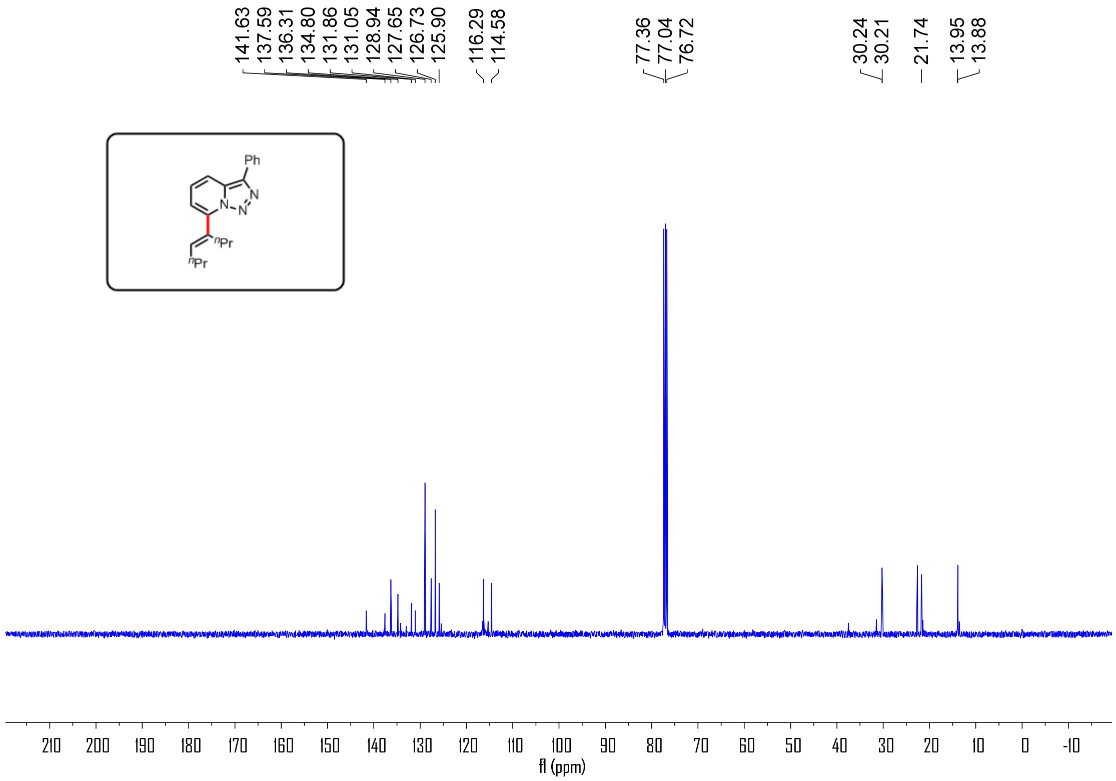

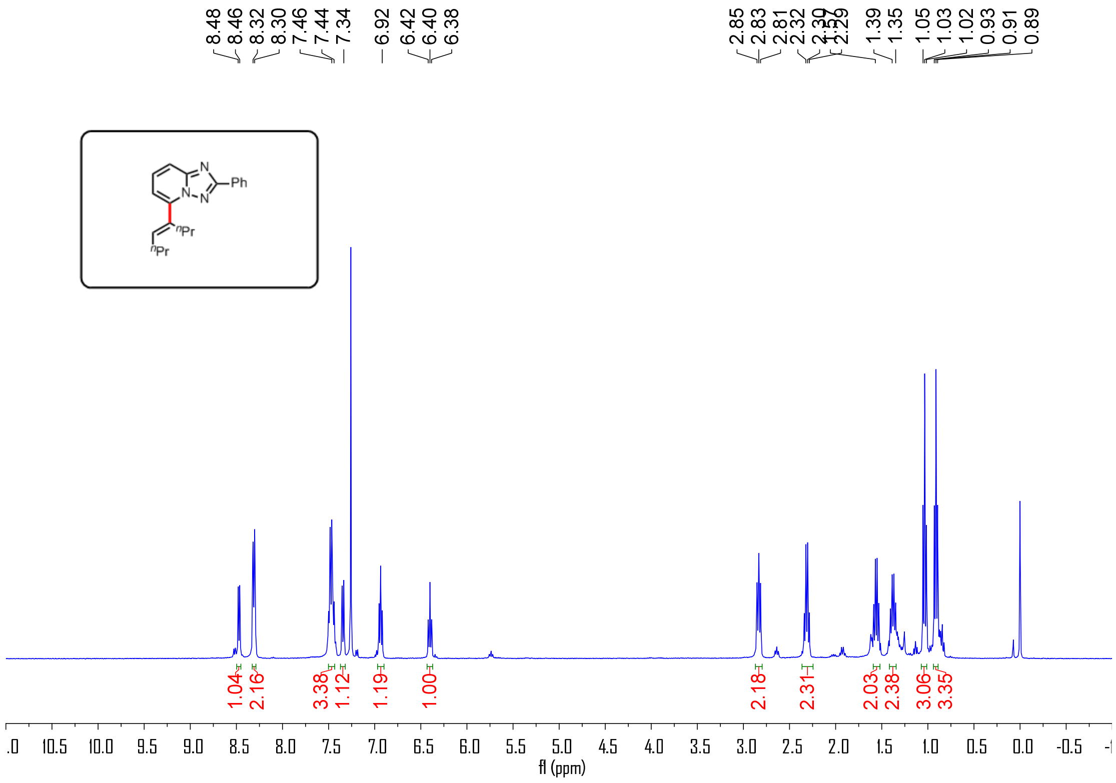

Supplementary Figure 81. ^{13}C (**3x**) and ^{1}H (**3y-mono**) NMR spectra in $CDCl_3$.

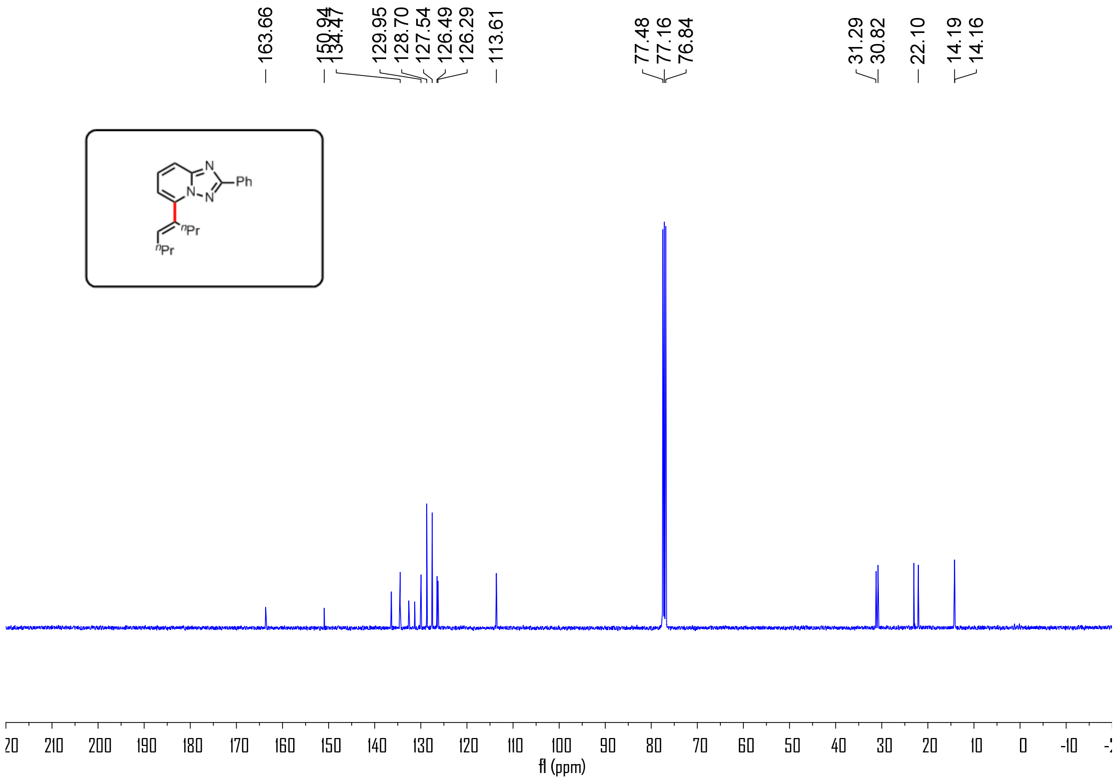

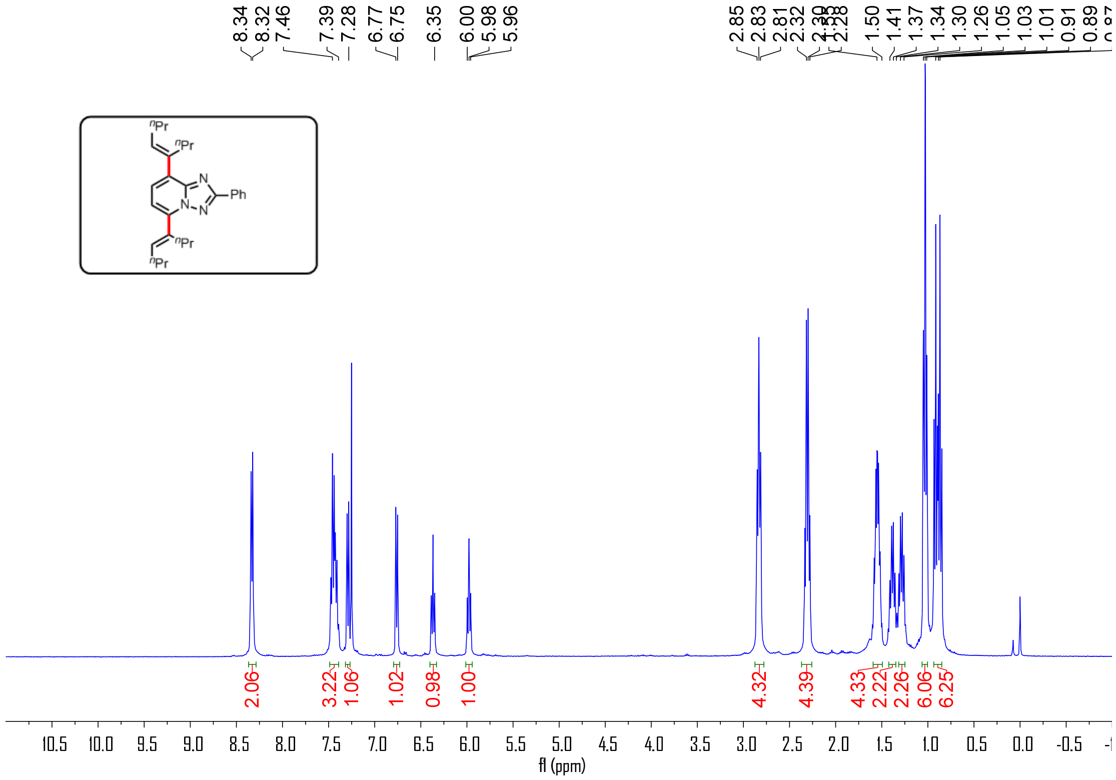

Supplementary Figure 82. ^{13}C (**3y-mono**) and ^{1}H (**3y-di**) NMR spectra in $CDCl_3$.

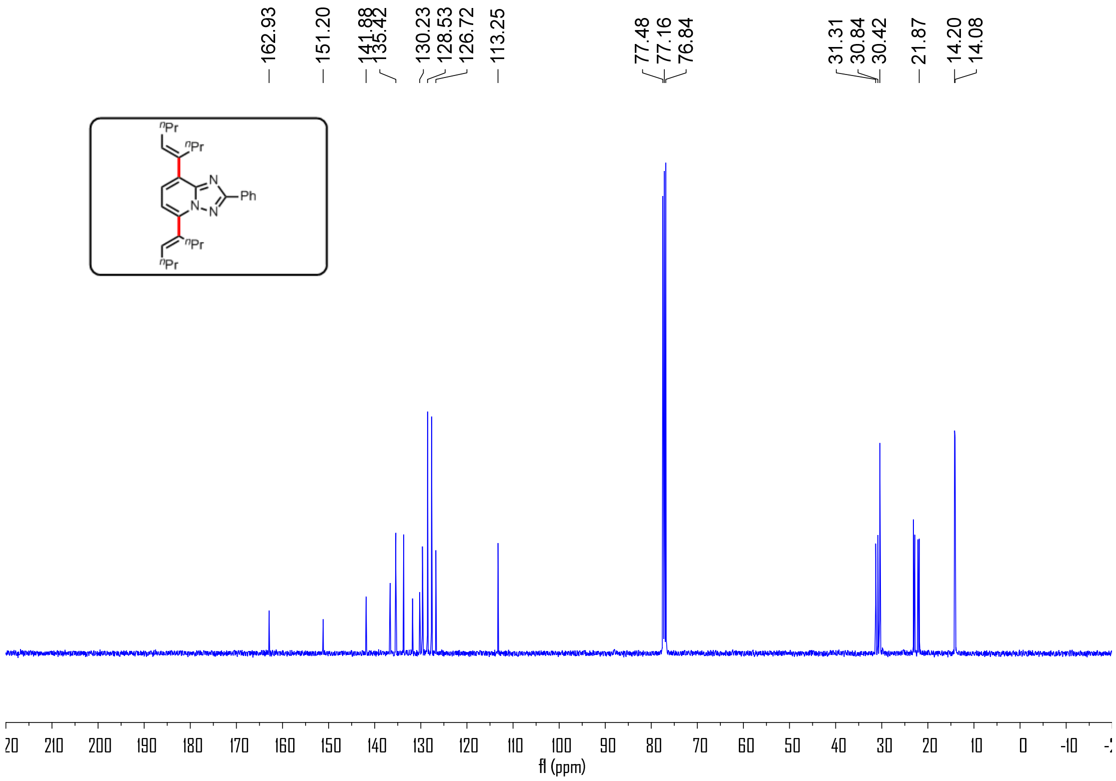

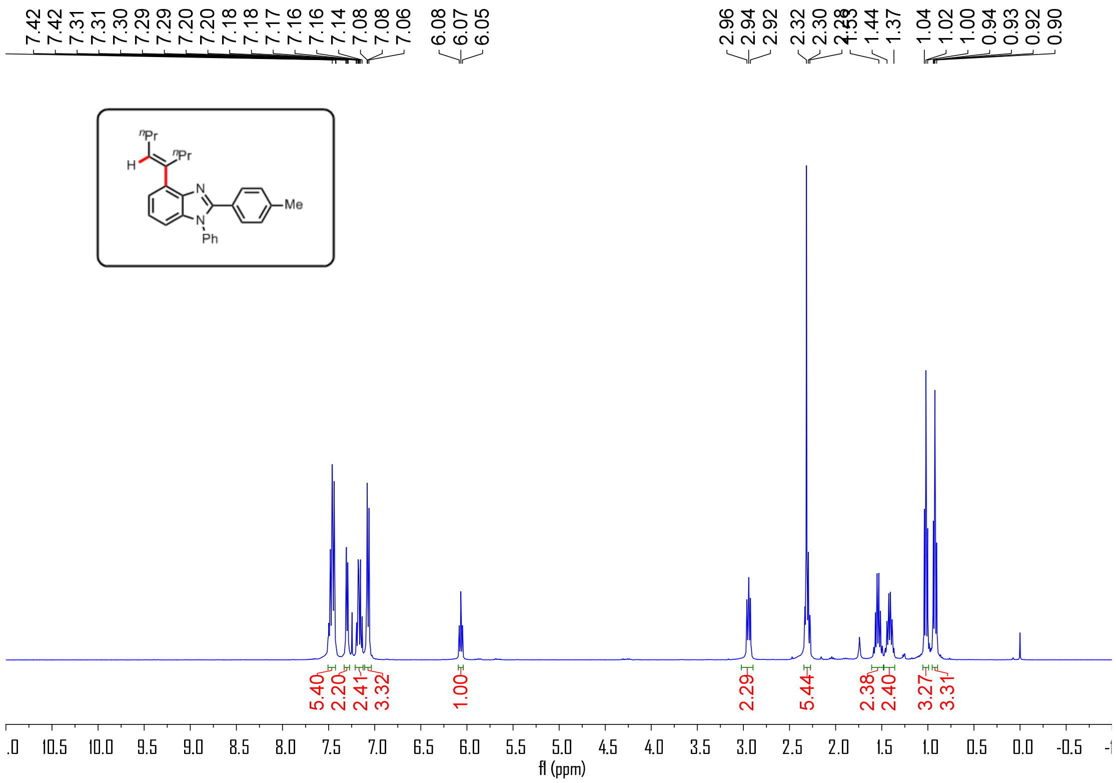

Supplementary Figure 83. ^{13}C (**3y-di**) and ^{1}H (**4a**) NMR spectra in $CDCl_3$.

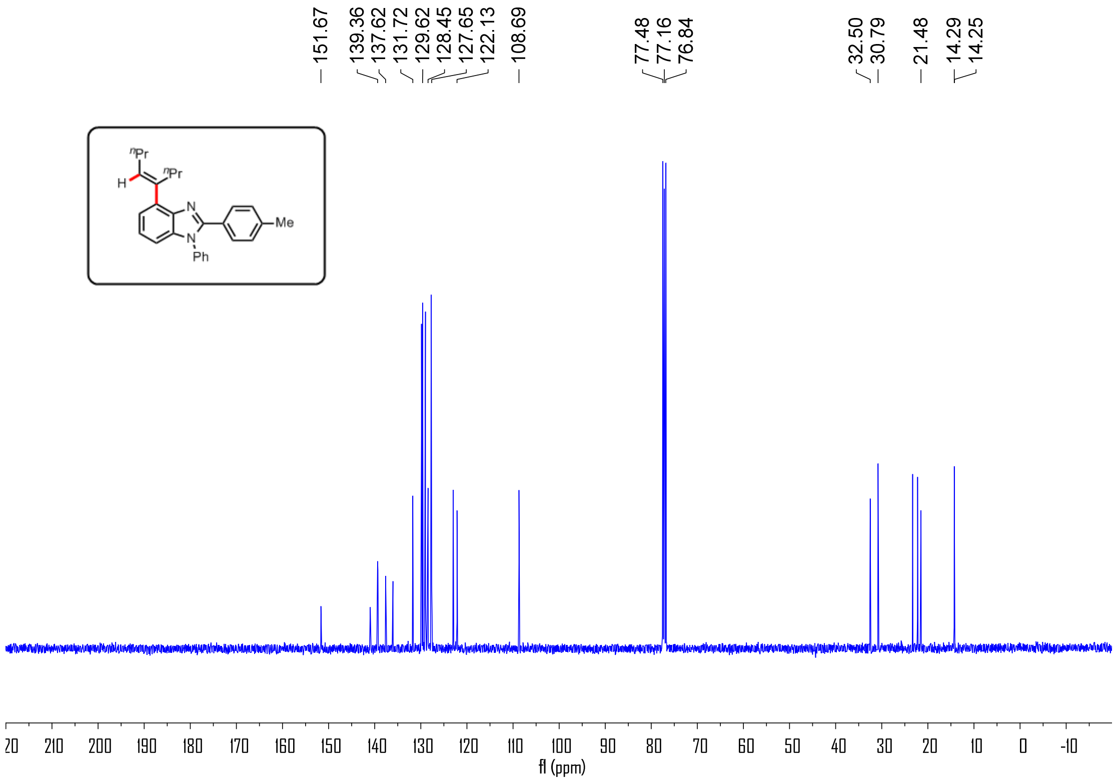

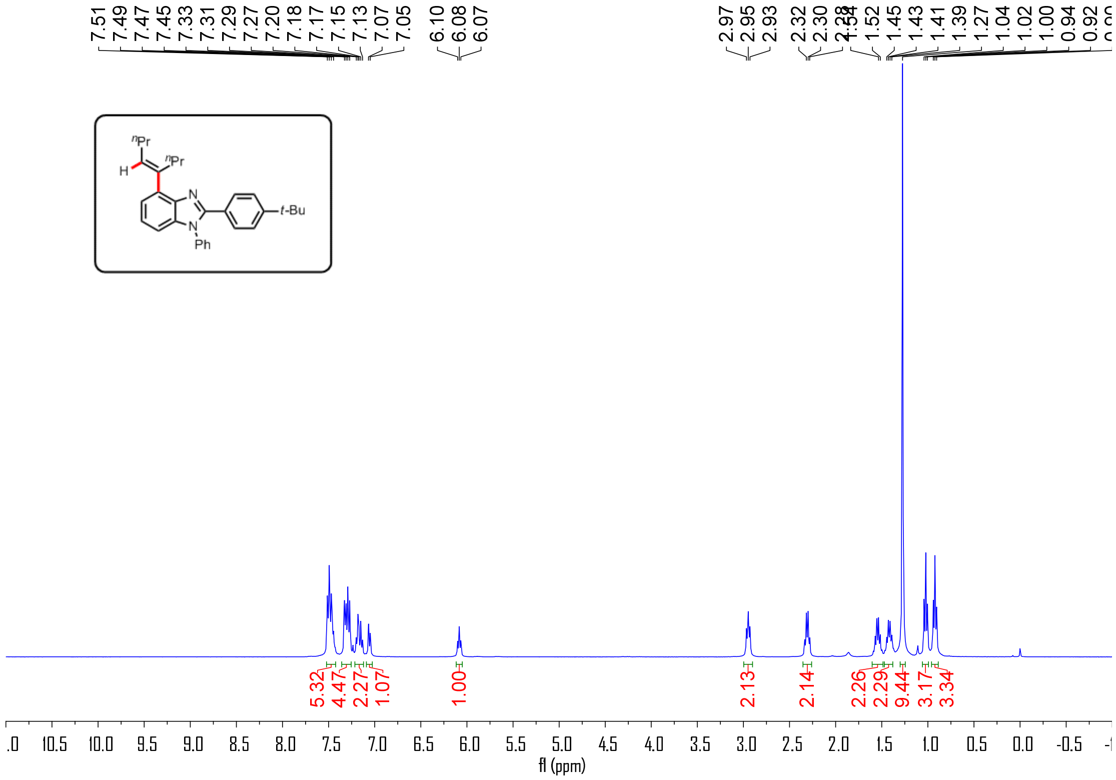

Supplementary Figure 84. ^{13}C (**4a**) and ^{1}H (**4b**) NMR spectra in $CDCl_3$.

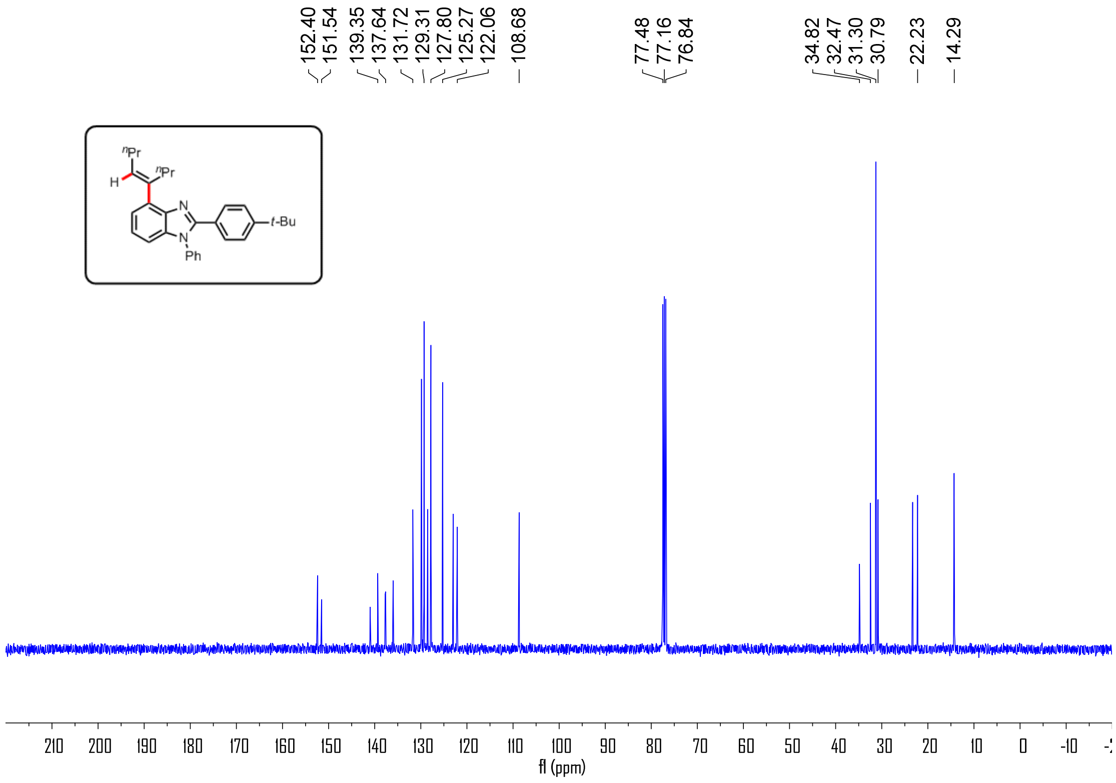

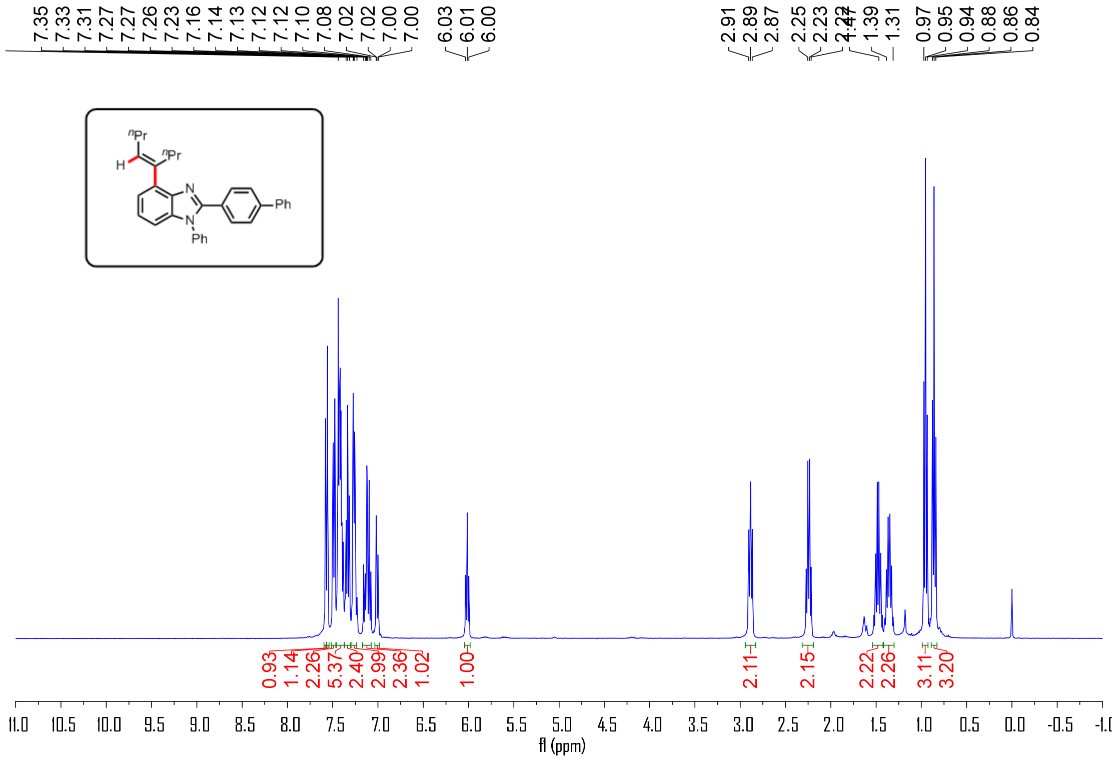

Supplementary Figure 85. ^{13}C (**4b**) and ^{1}H (**4c**) NMR spectra in $CDCl_3$.

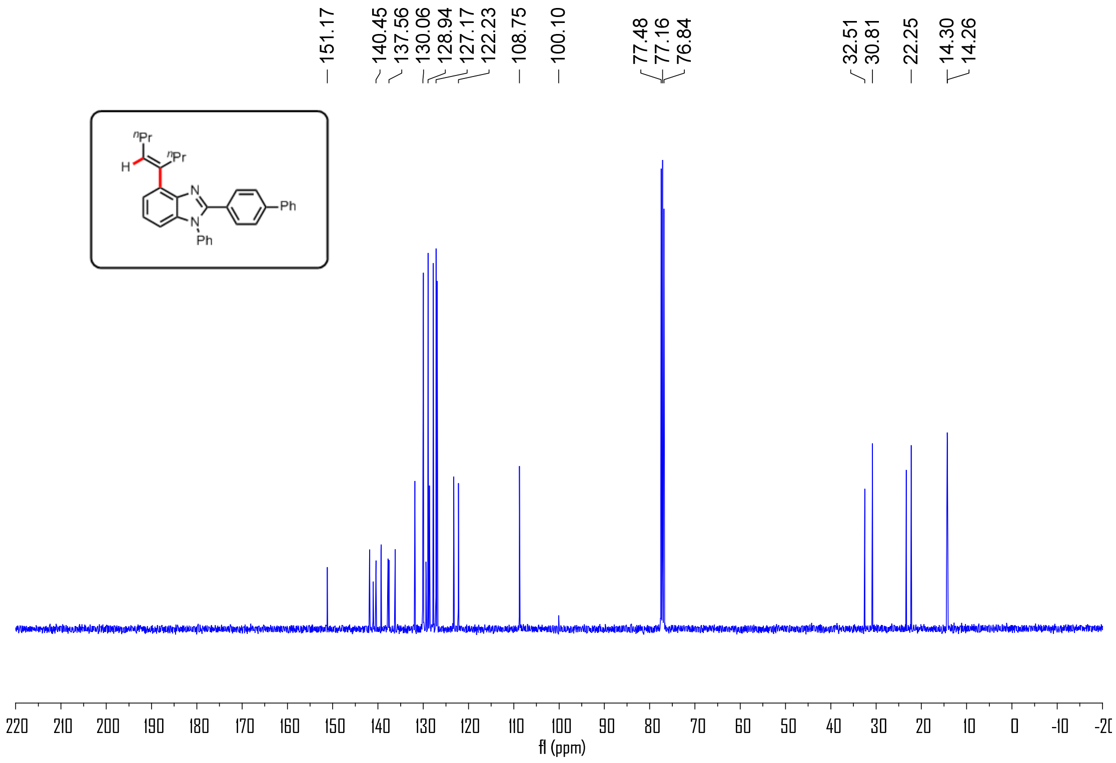

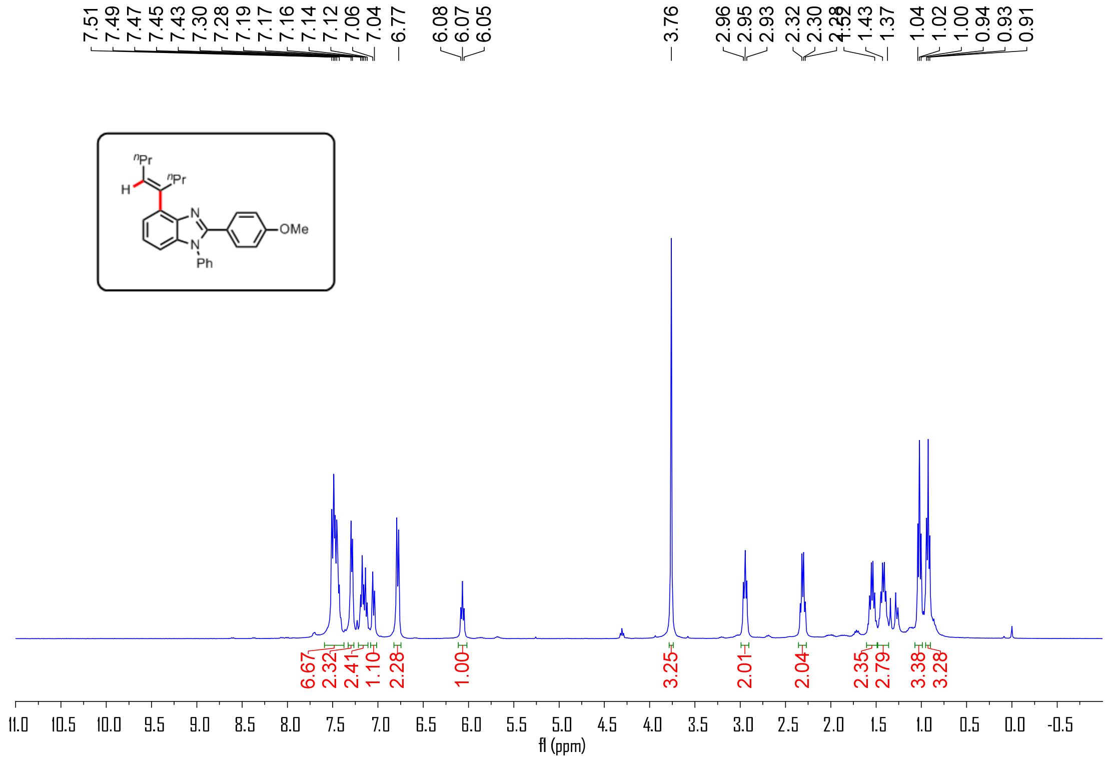

Supplementary Figure 86. ^{13}C (**4c**) and ^{1}H (**4d**) NMR spectra in $CDCl_3$.

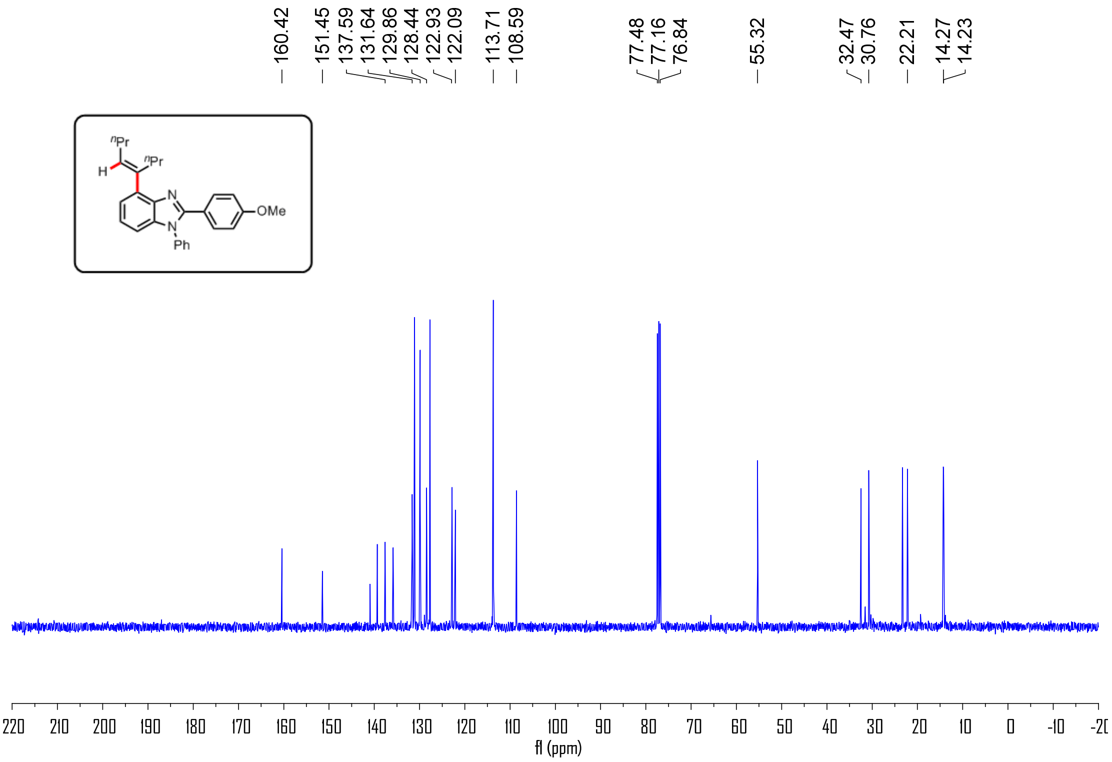

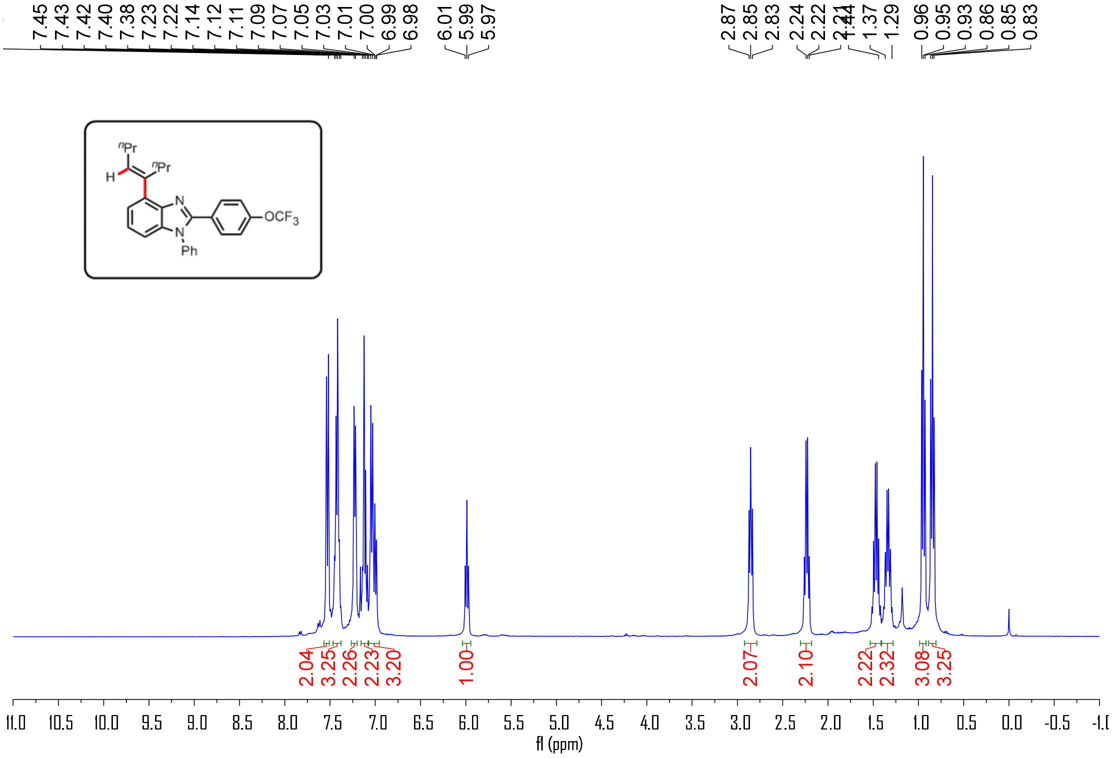

Supplementary Figure 87. ^{13}C (**4d**) and ^{1}H (**4e**) NMR spectra in $CDCl_3$.

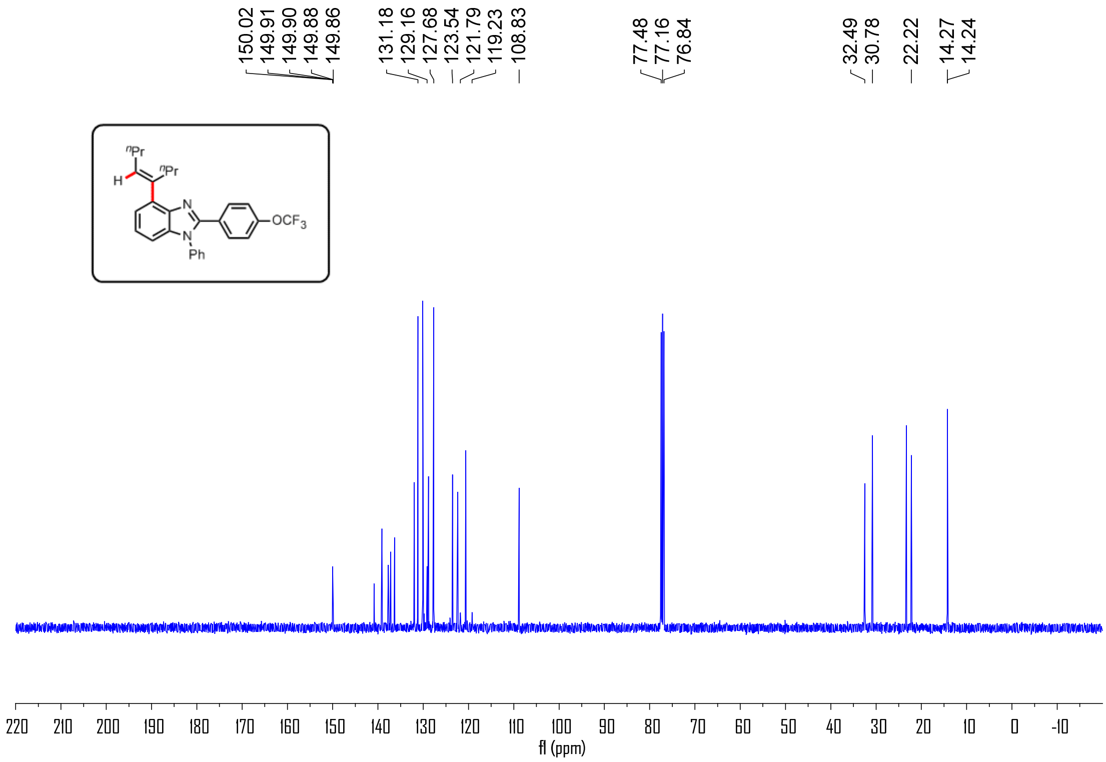

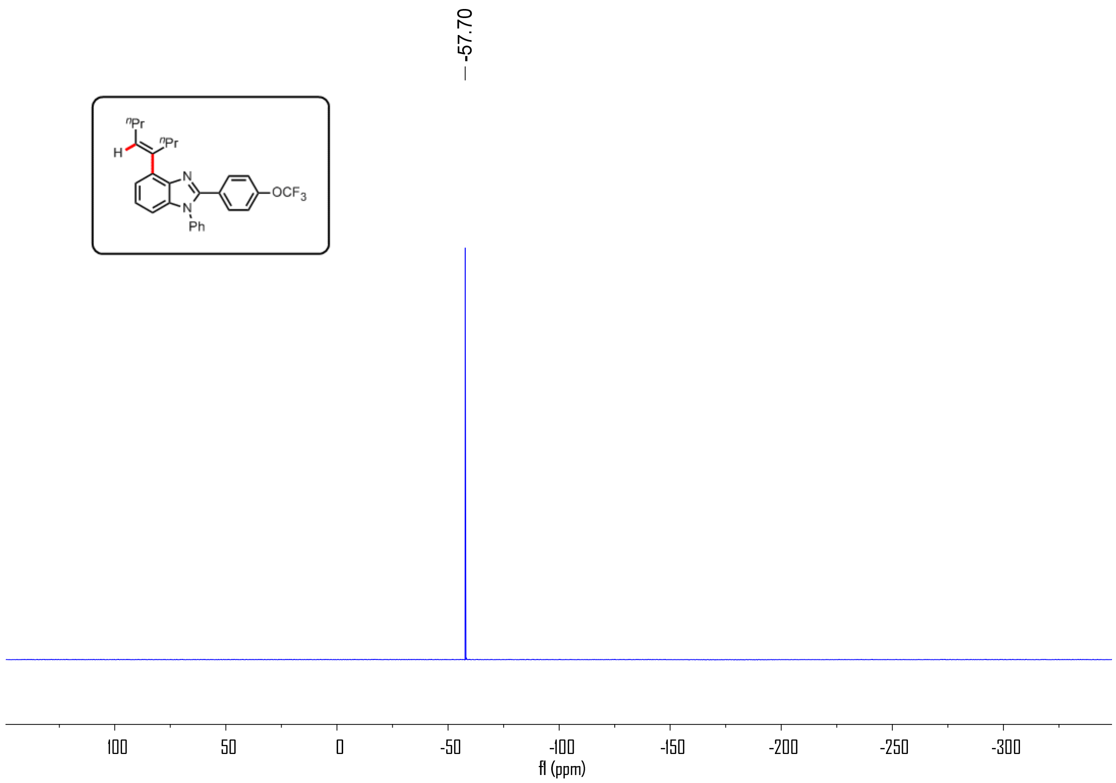

Supplementary Figure 88. ^{13}C (**4e**) and ^{19}F (**4e**) NMR spectra in $CDCl_3$.

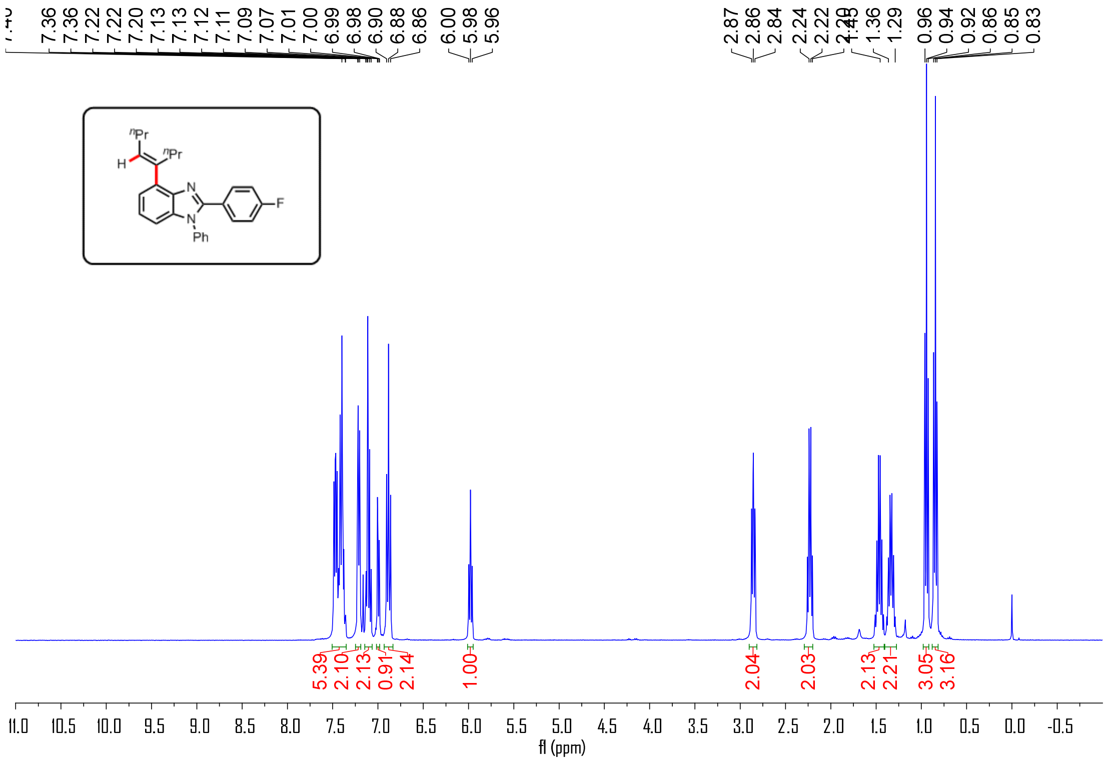

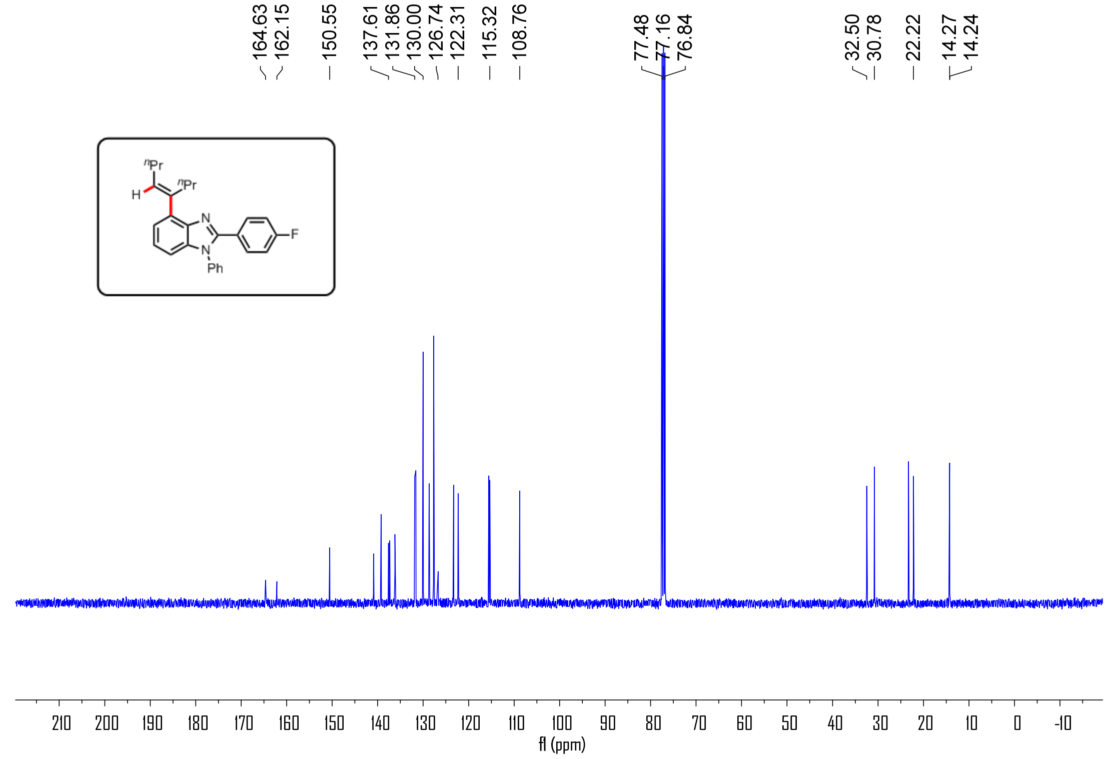

Supplementary Figure 89. ^{1}H (**4f**) and ^{13}C (**4f**) NMR spectra in $CDCl_3$.

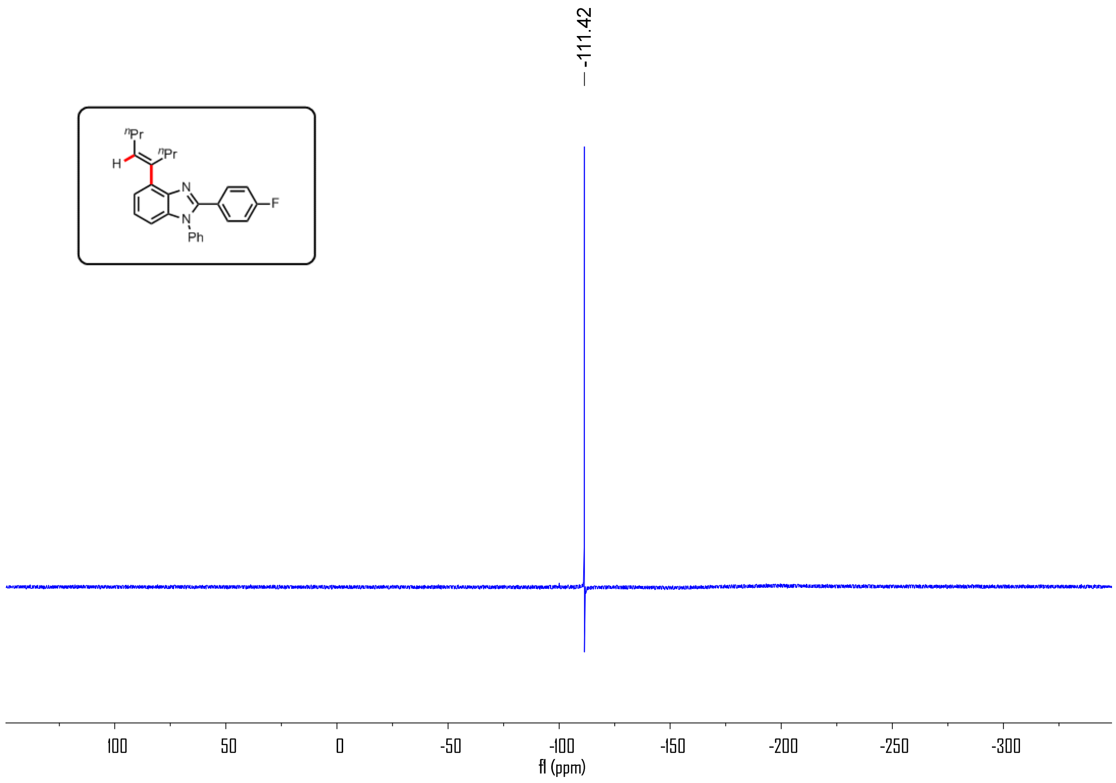

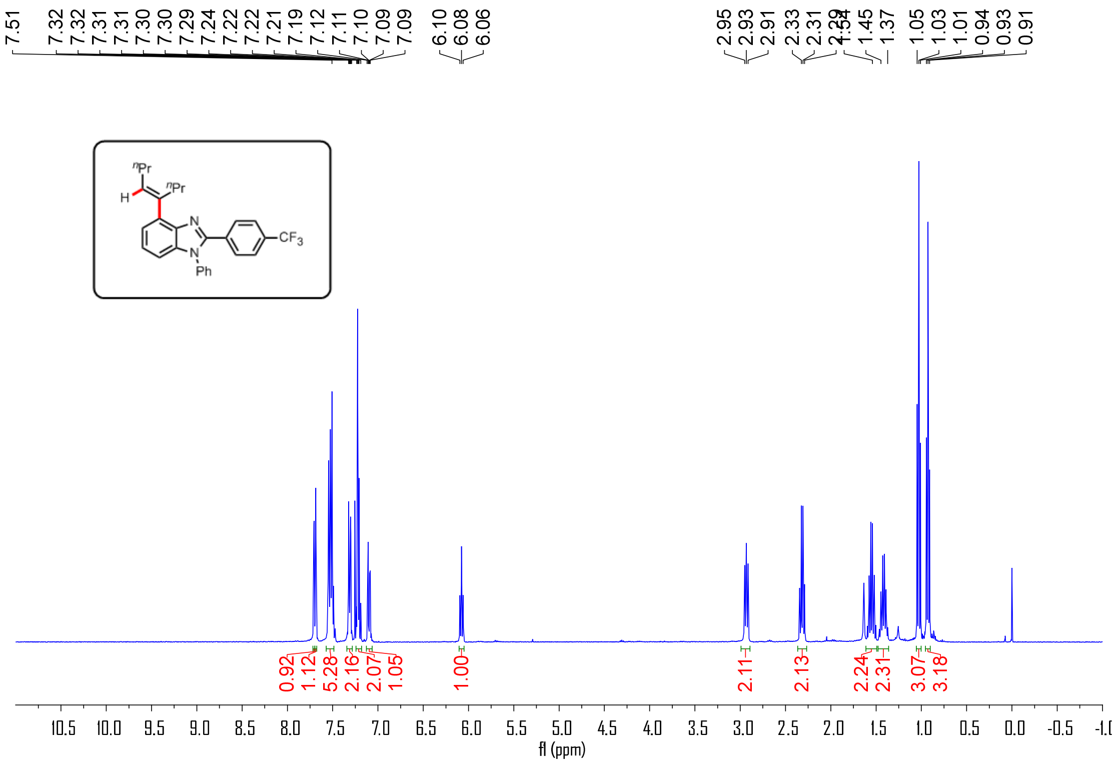

Supplementary Figure 90. ^{19}F (**4f**) and ^{1}H (**4g**) NMR spectra in $CDCl_3$.

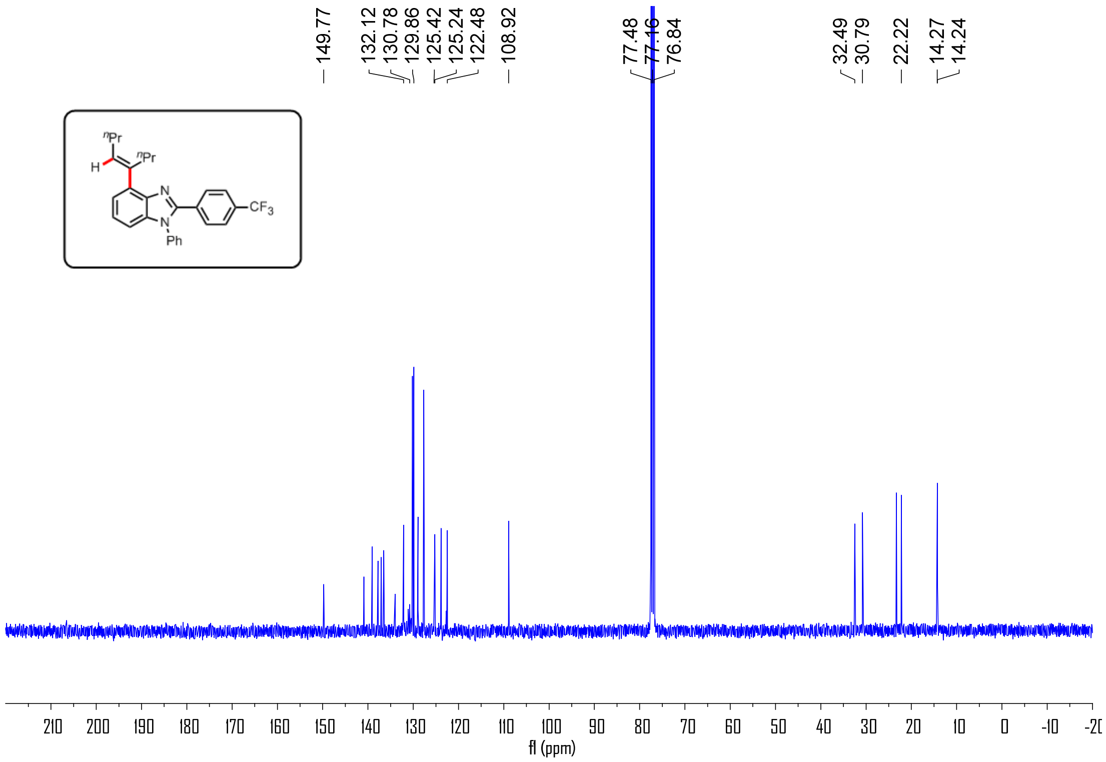

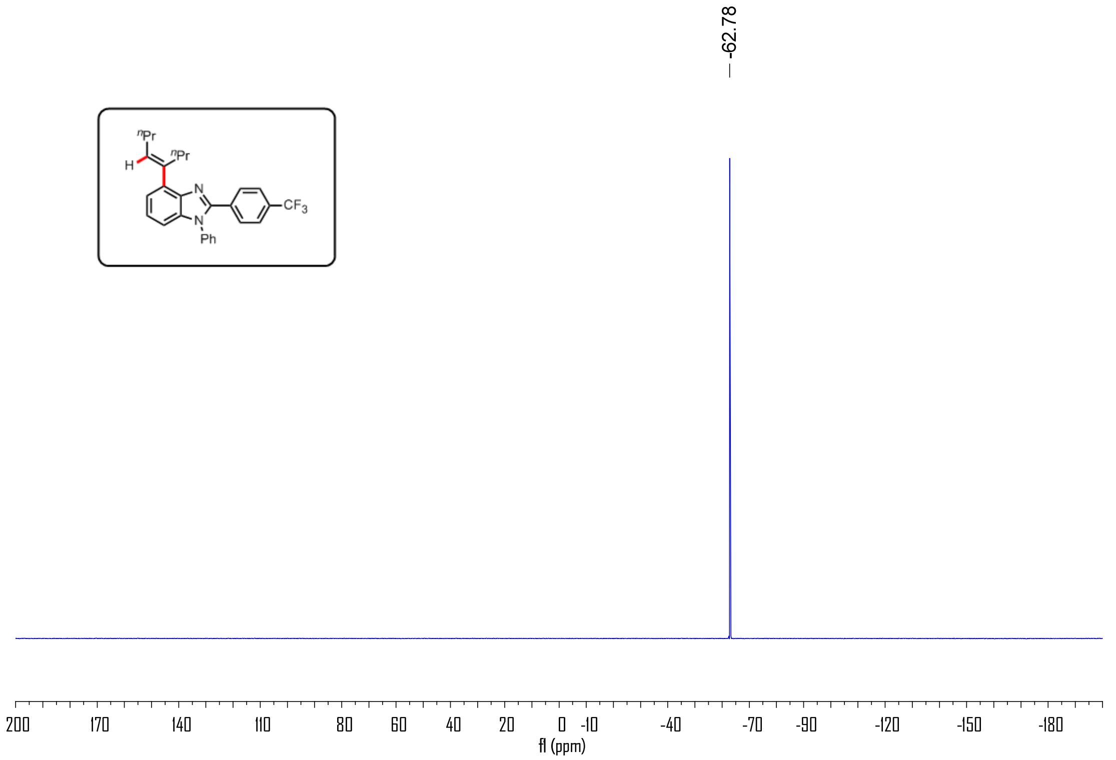

Supplementary Figure 91. ^{13}C (**4g**) and ^{19}F (**4g**) NMR spectra in $CDCl_3$.

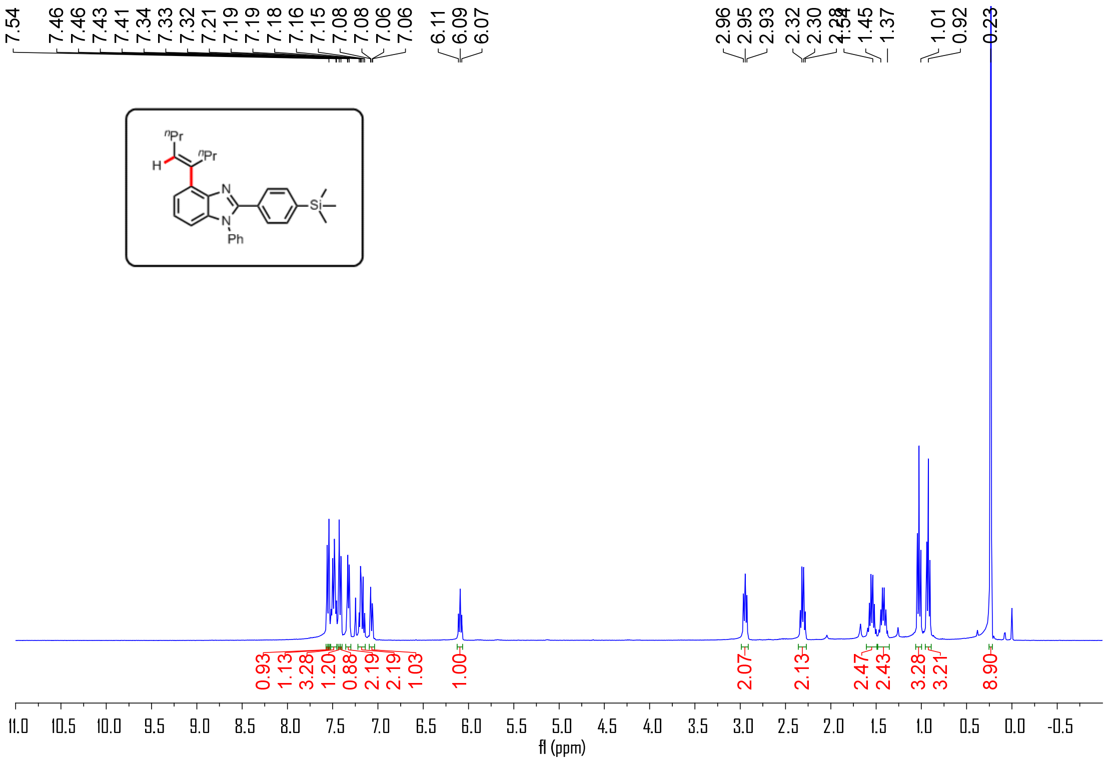

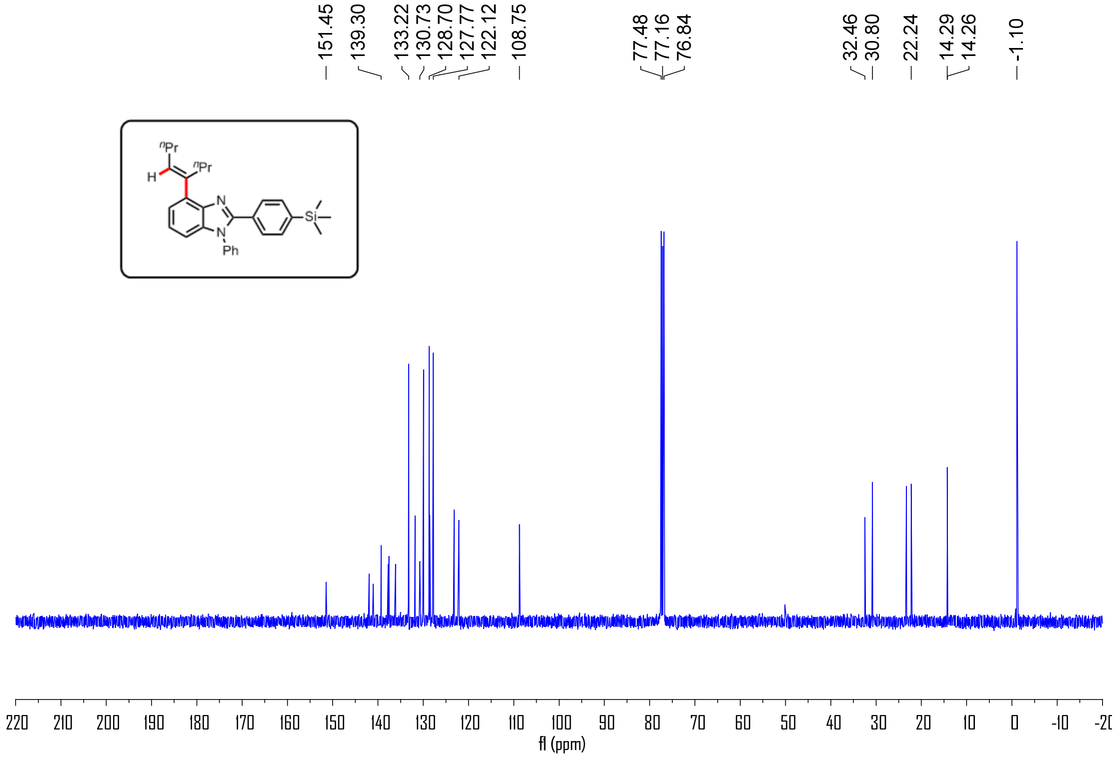

Supplementary Figure 92. ^{1}H (**4h**) and ^{13}C (**4h**) NMR spectra in $CDCl_3$.

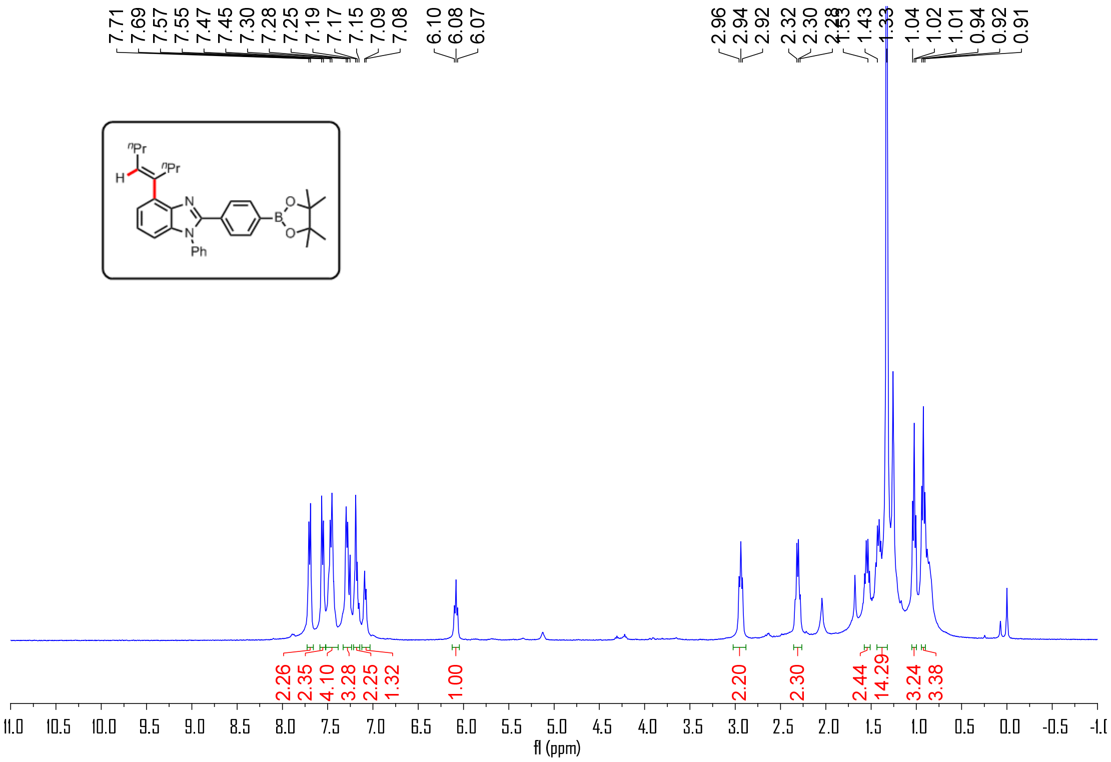

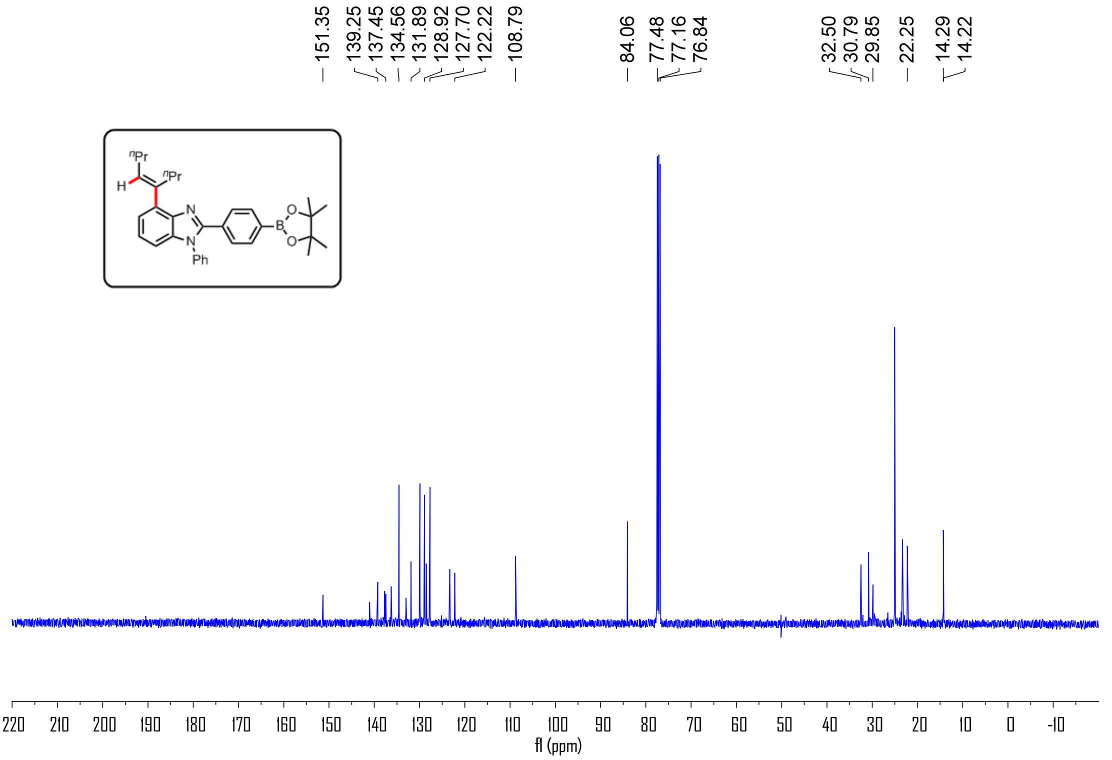

Supplementary Figure 93. ^{1}H (**4i**) and ^{13}C (**4i**) NMR spectra in $CDCl_3$.

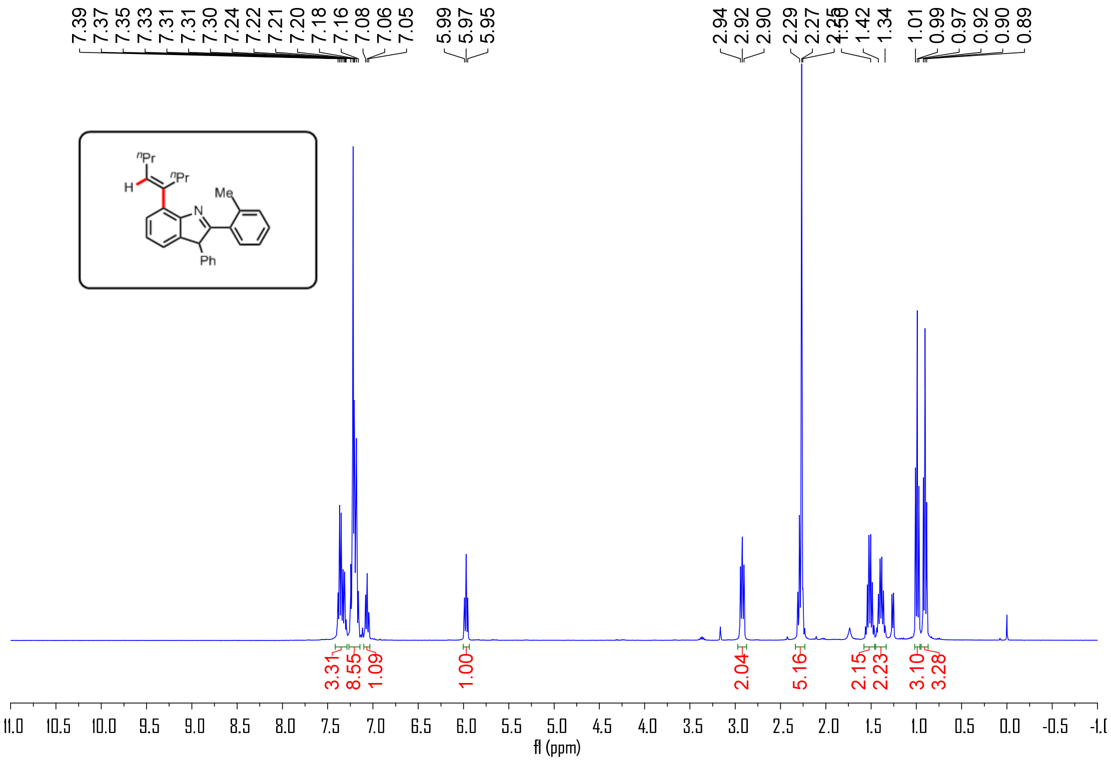

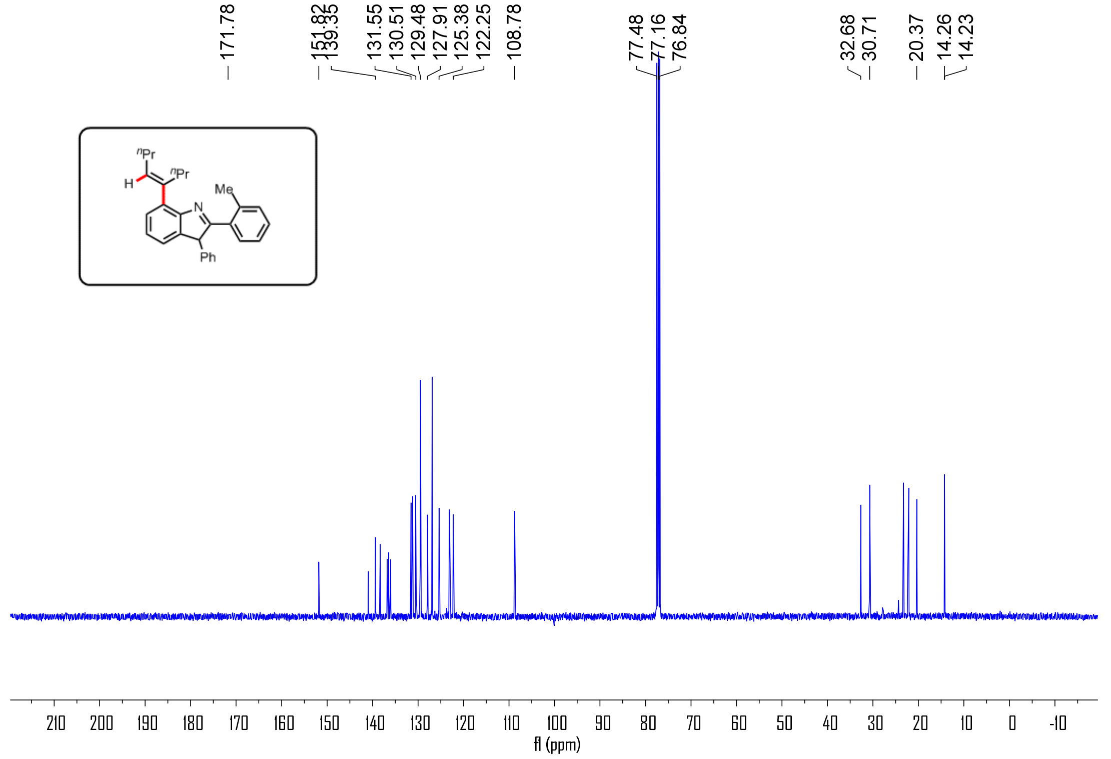

Supplementary Figure 94. ^{1}H (**4j**) and ^{13}C (**4j**) NMR spectra in $CDCl_3$.

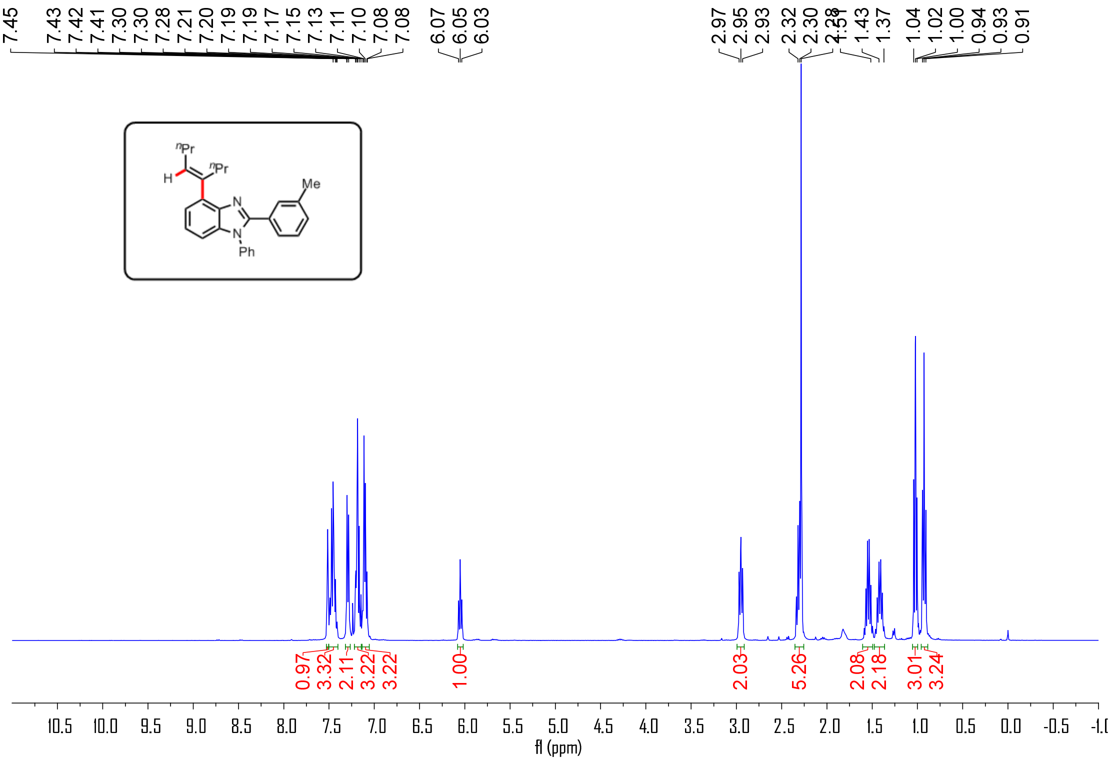

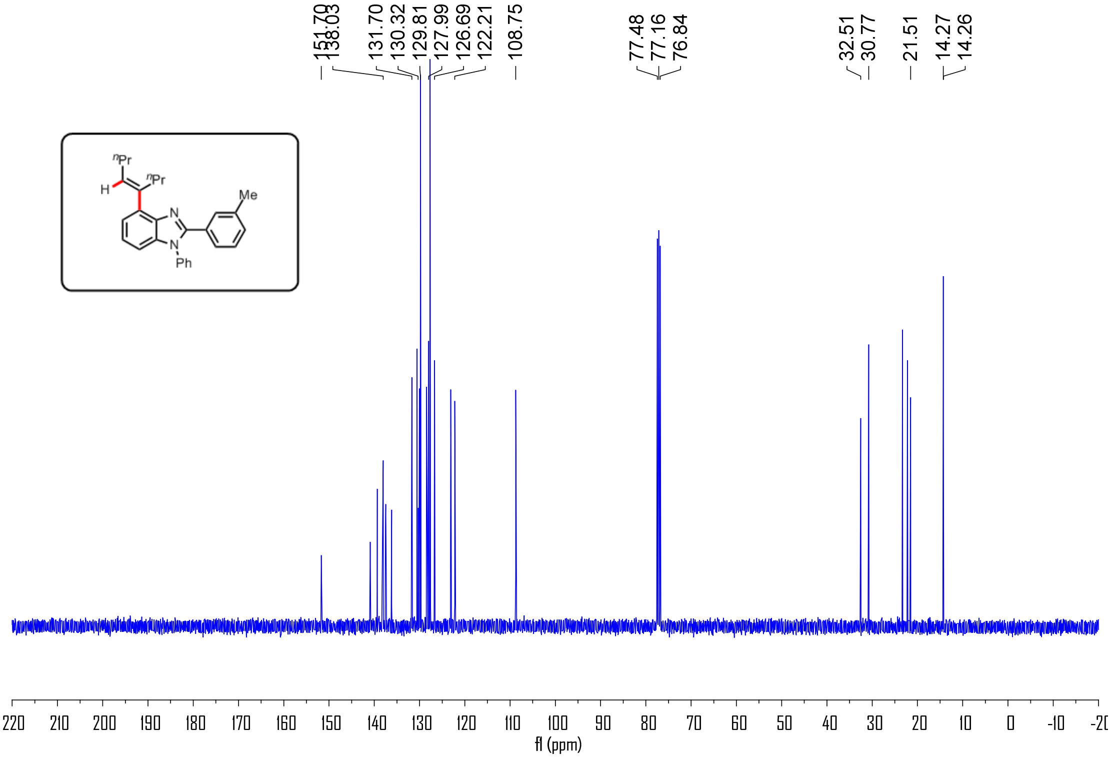

Supplementary Figure 95. ^{1}H (**4k**) and ^{13}C (**4k**) NMR spectra in $CDCl_3$.

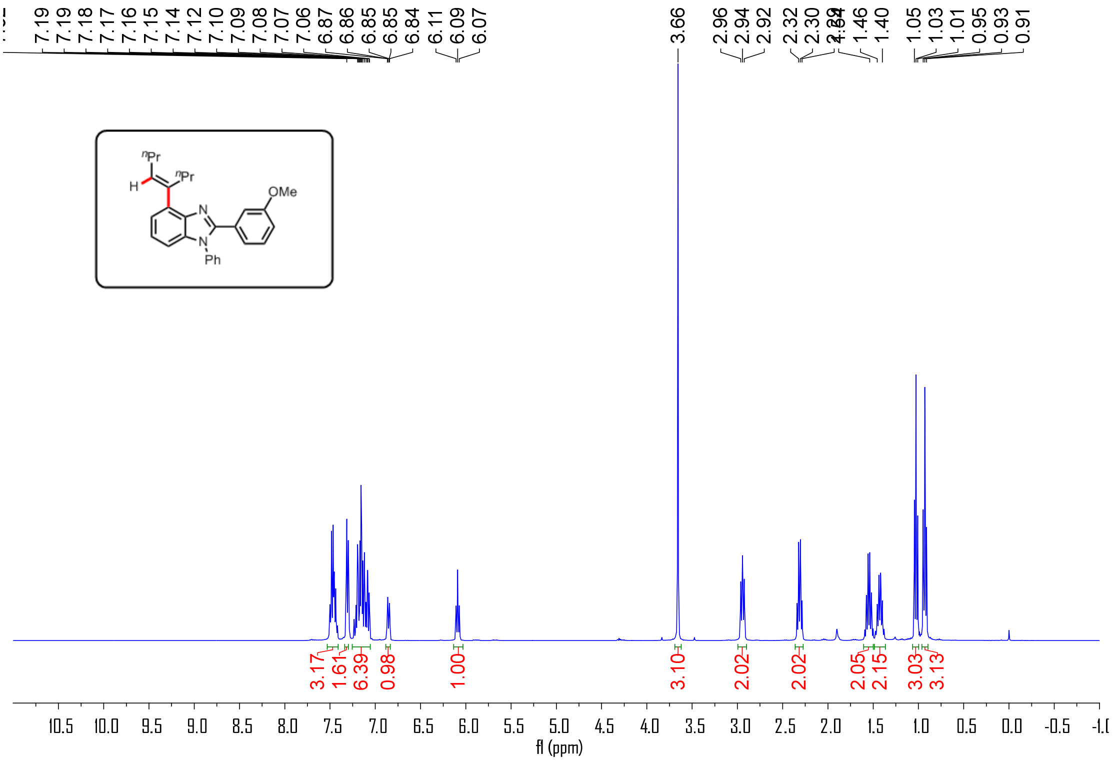

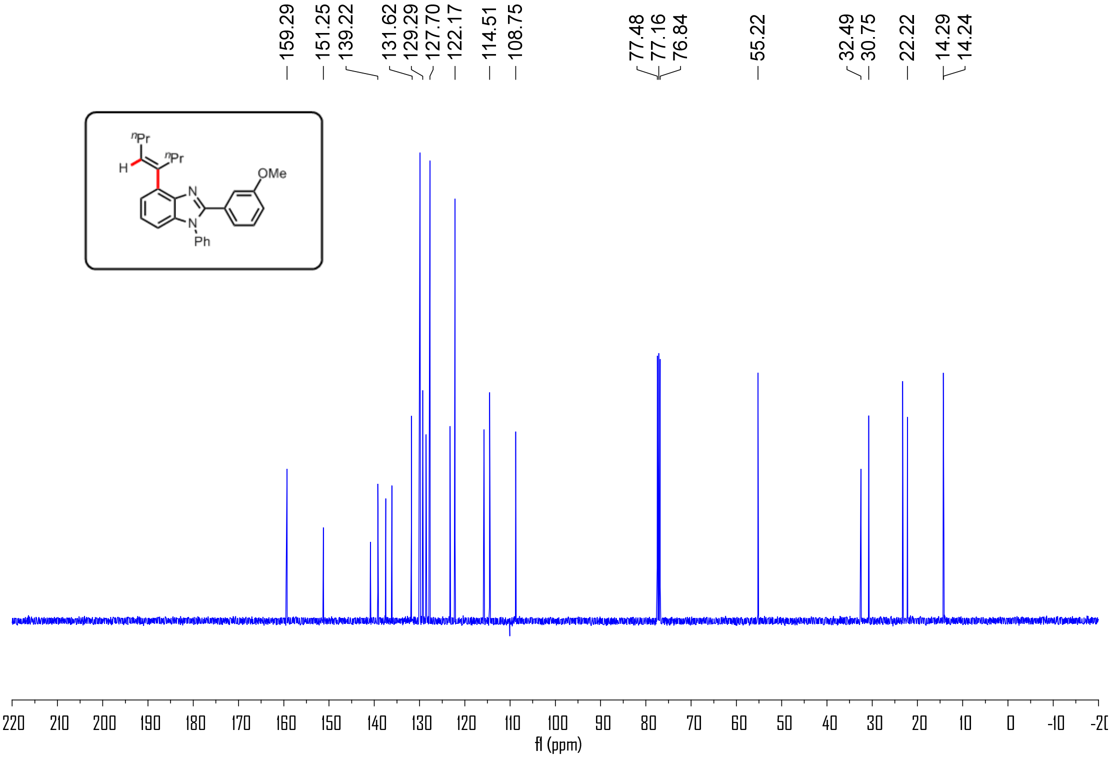

Supplementary Figure 96. ^{1}H (**4l**) and ^{13}C (**4l**) NMR spectra in $CDCl_3$.

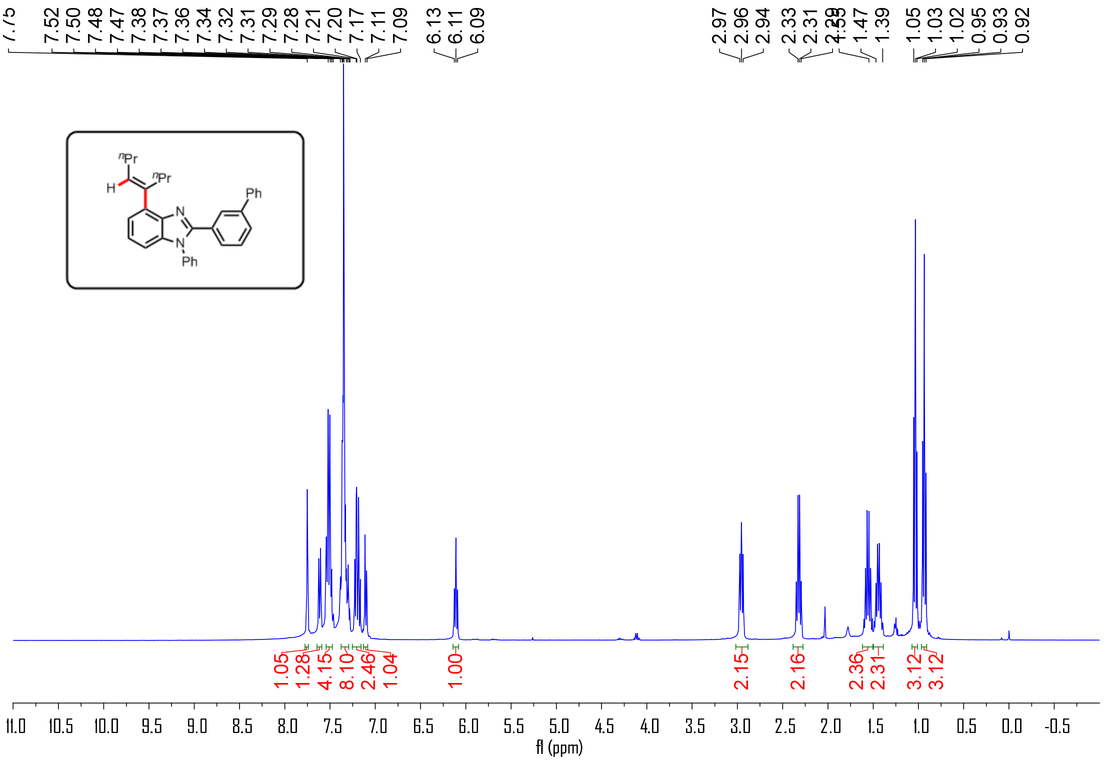

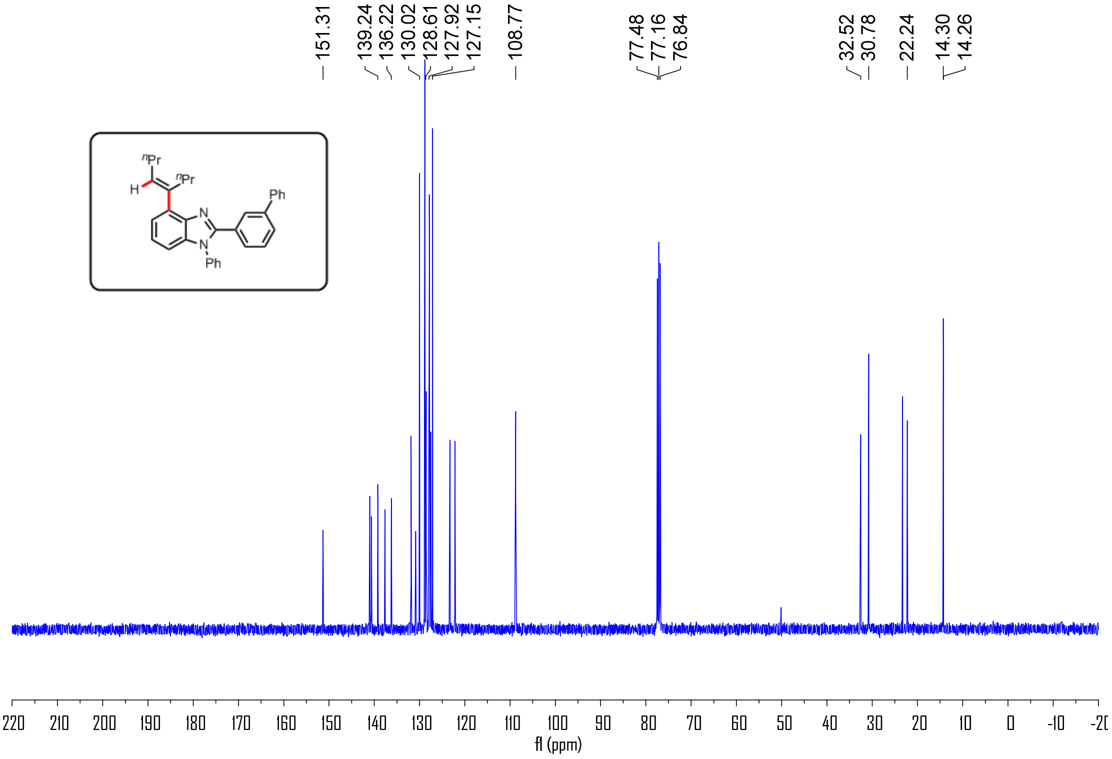

Supplementary Figure 97. ^{1}H (**4m**) and ^{13}C (**4m**) NMR spectra in $CDCl_3$.

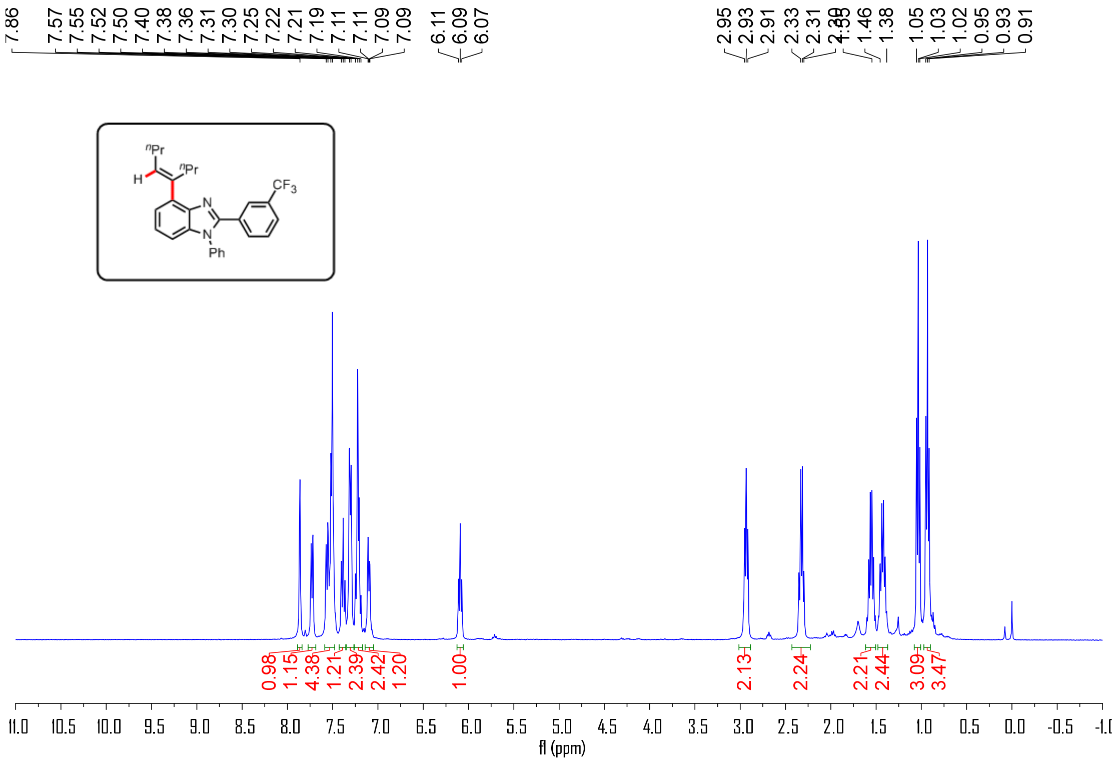

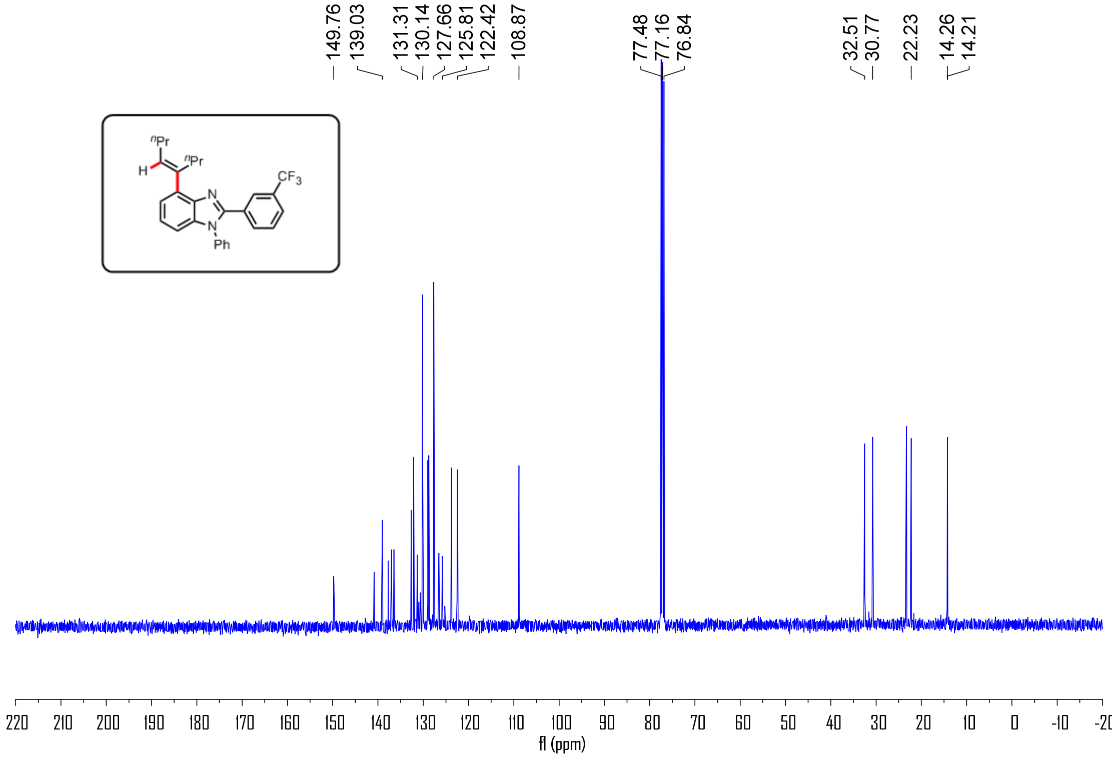

Supplementary Figure 98. ^{1}H (**4n**) and ^{13}C (**4n**) NMR spectra in $CDCl_3$.

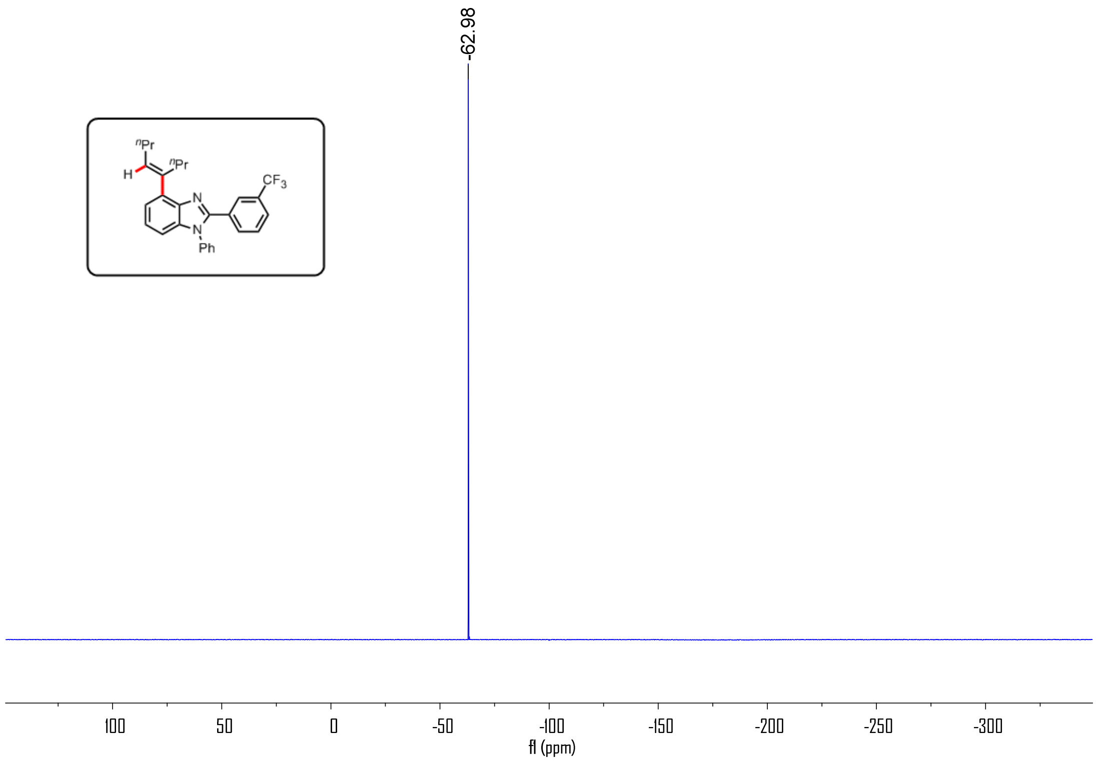

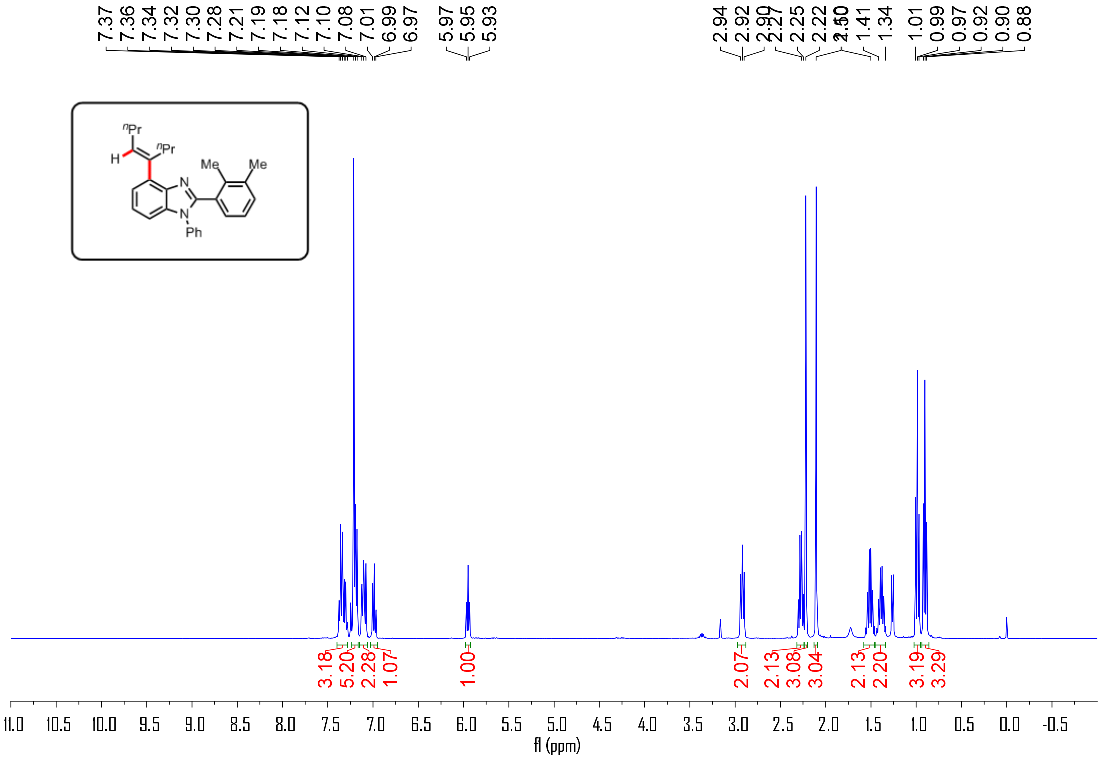

Supplementary Figure 99. ^{19}F (**4n**) and ^{1}H (**4o**) NMR spectra in $CDCl_3$.

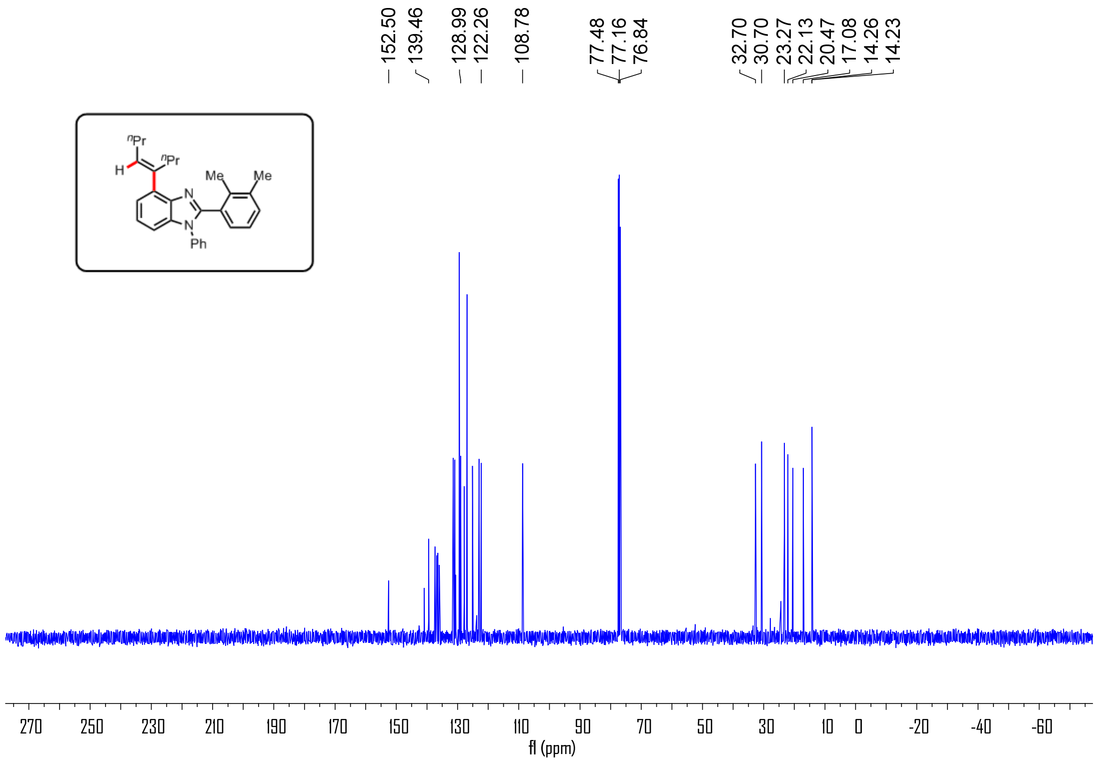

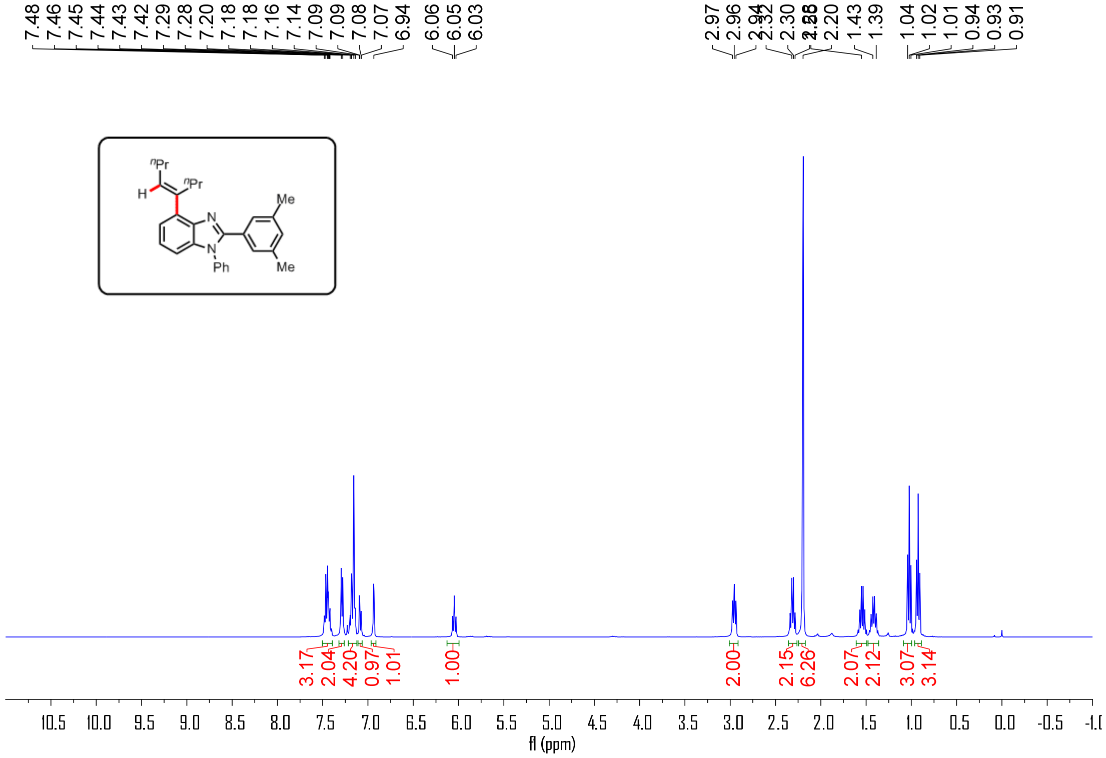

Supplementary Figure 100. ^{13}F (**4o**) and ^{1}H (**4p**) NMR spectra in $CDCl_3$.

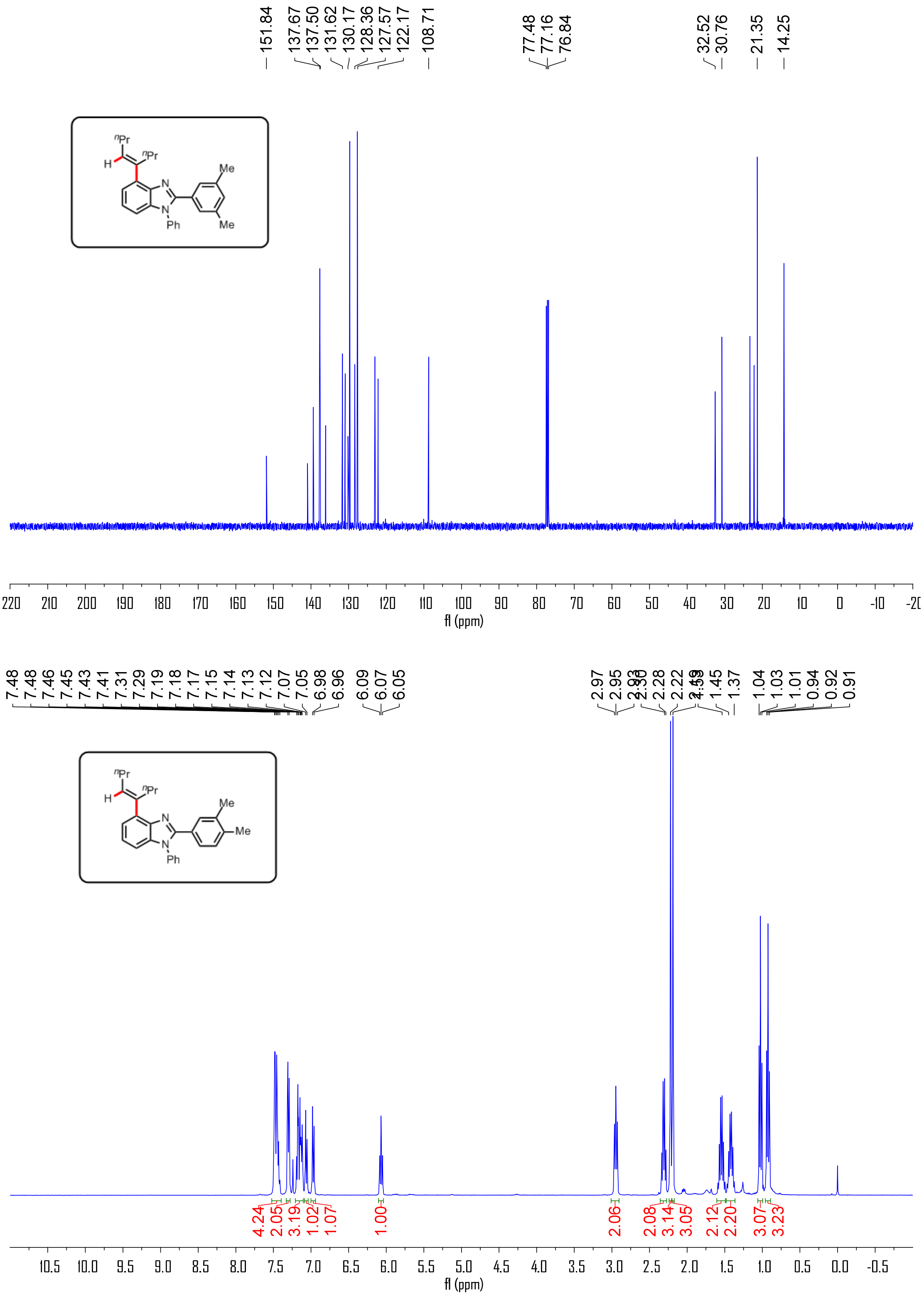

Supplementary Figure 101. ^{13}C (**4p**) and ^{1}H (**4q**) NMR spectra in $CDCl_3$.

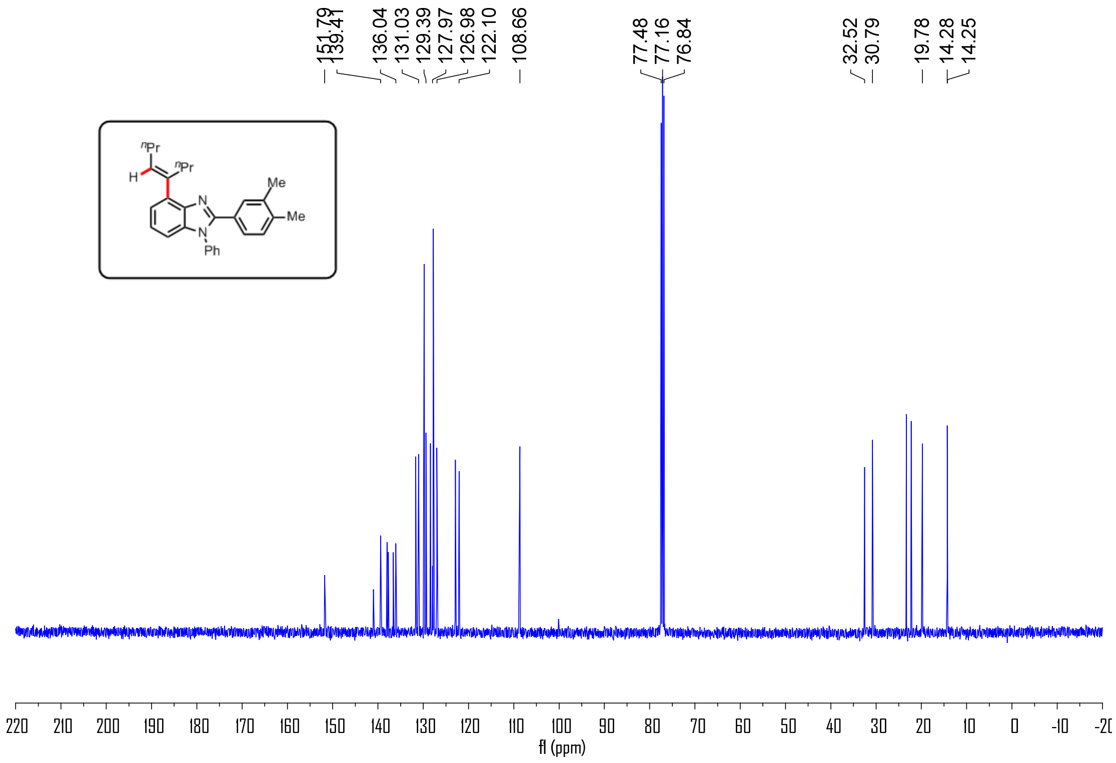

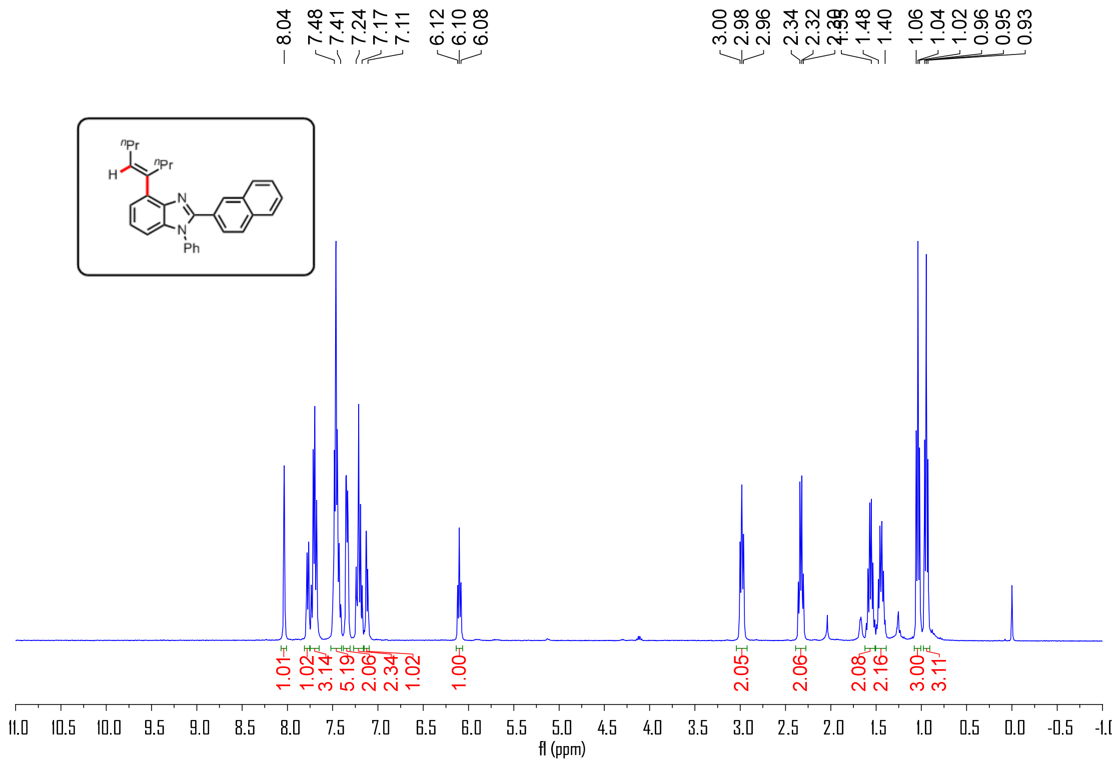

Supplementary Figure 102. ^{13}C (**4q**) and ^{1}H (**4r**) NMR spectra in $CDCl_3$.

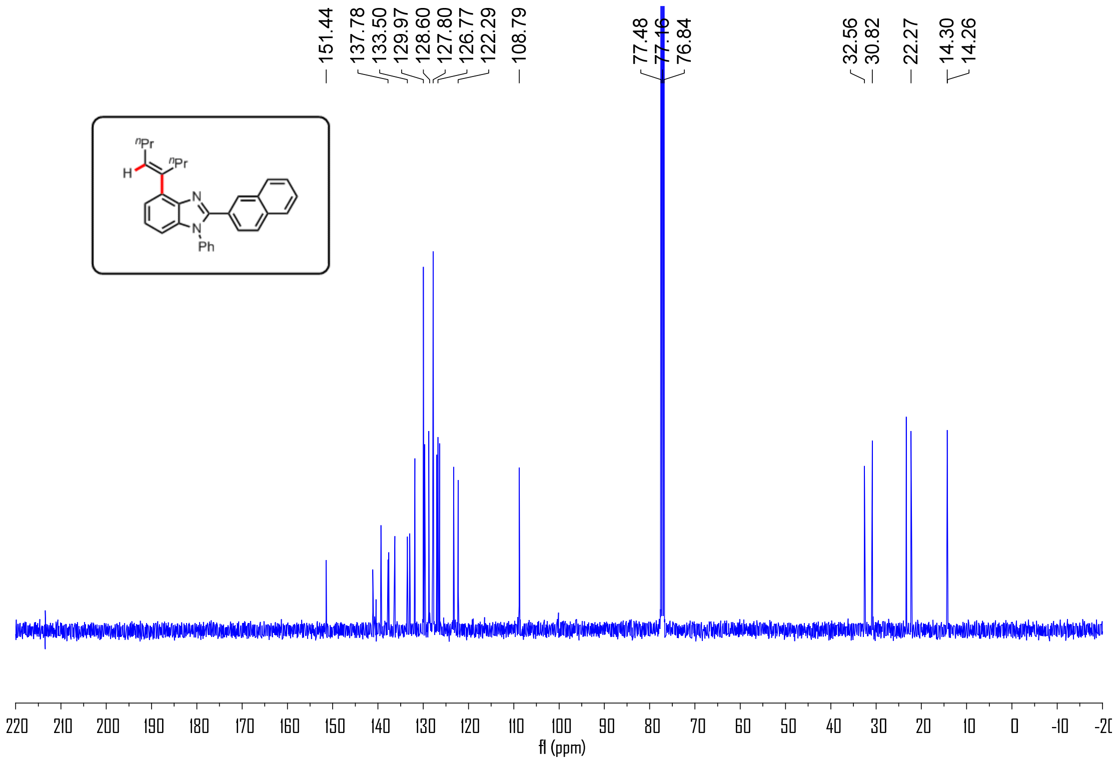

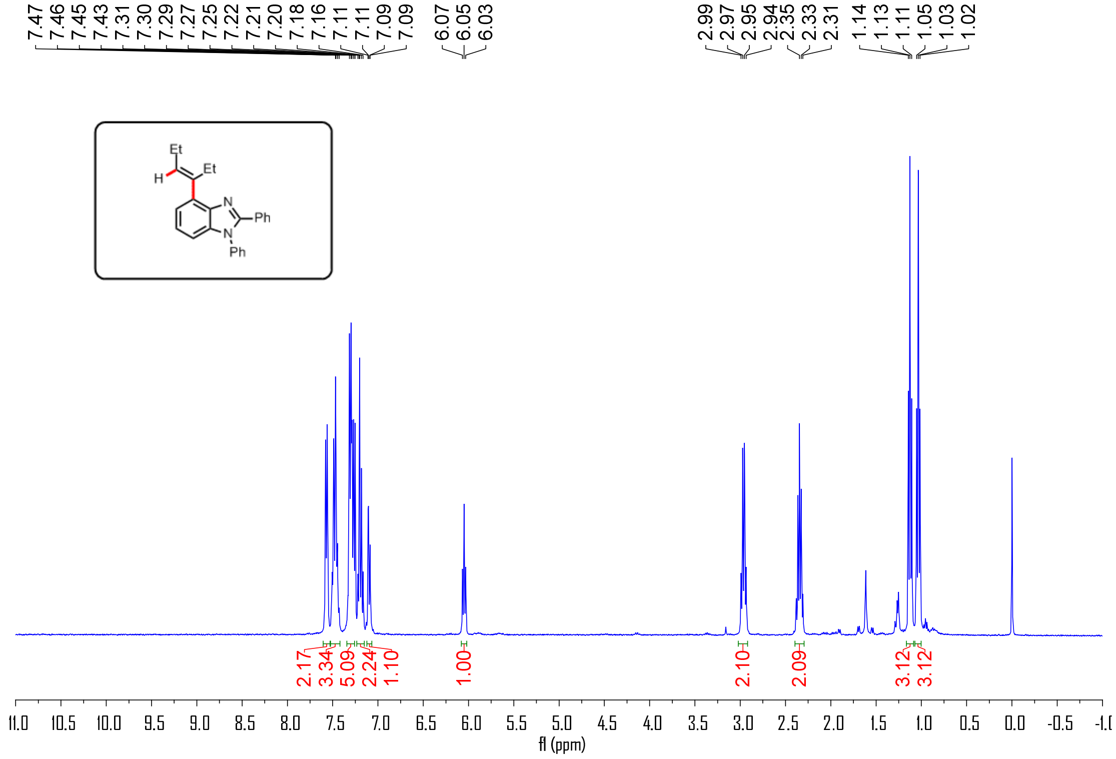

Supplementary Figure 103. ^{13}C (**4r**) and ^{1}H (**5a**) NMR spectra in $CDCl_3$.

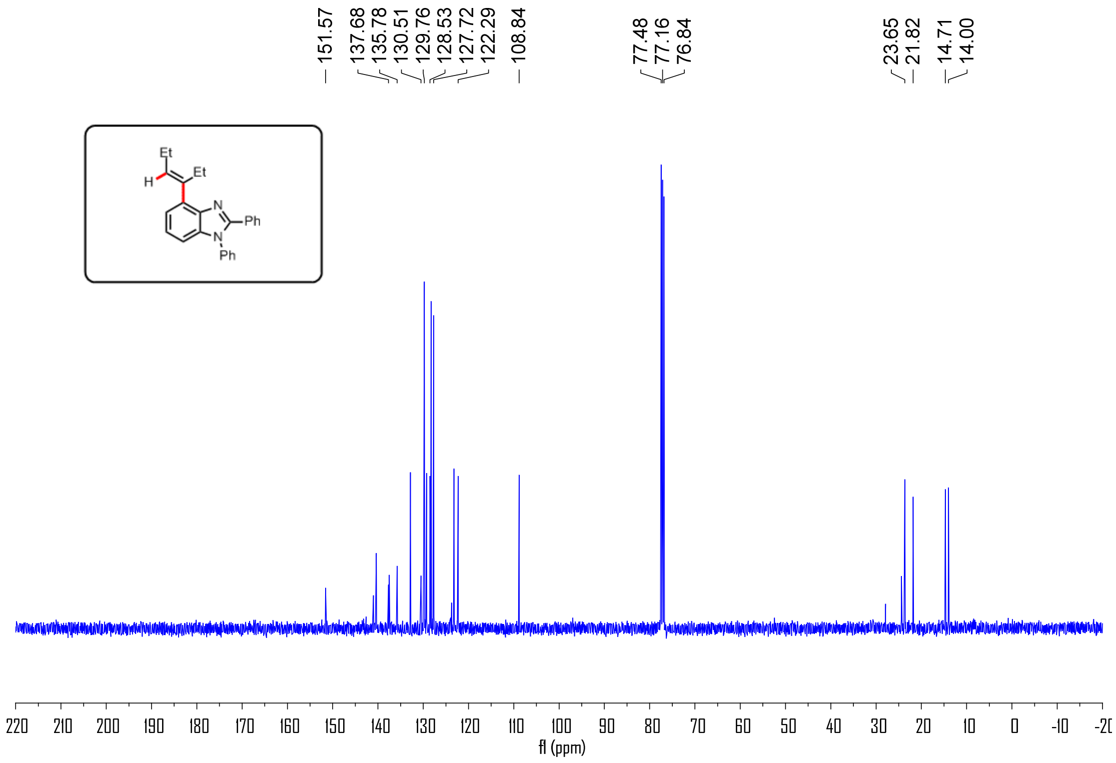

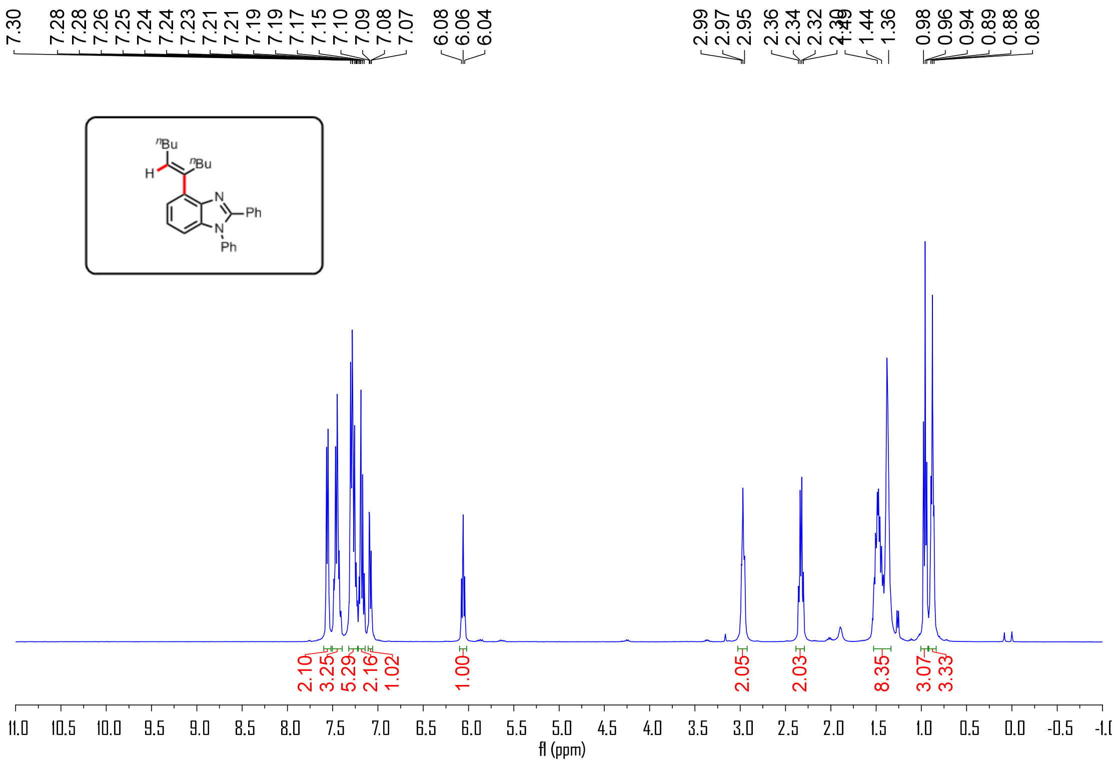

Supplementary Figure 104. ^{13}C (**5a**) and ^{1}H (**5b**) NMR spectra in $CDCl_3$.

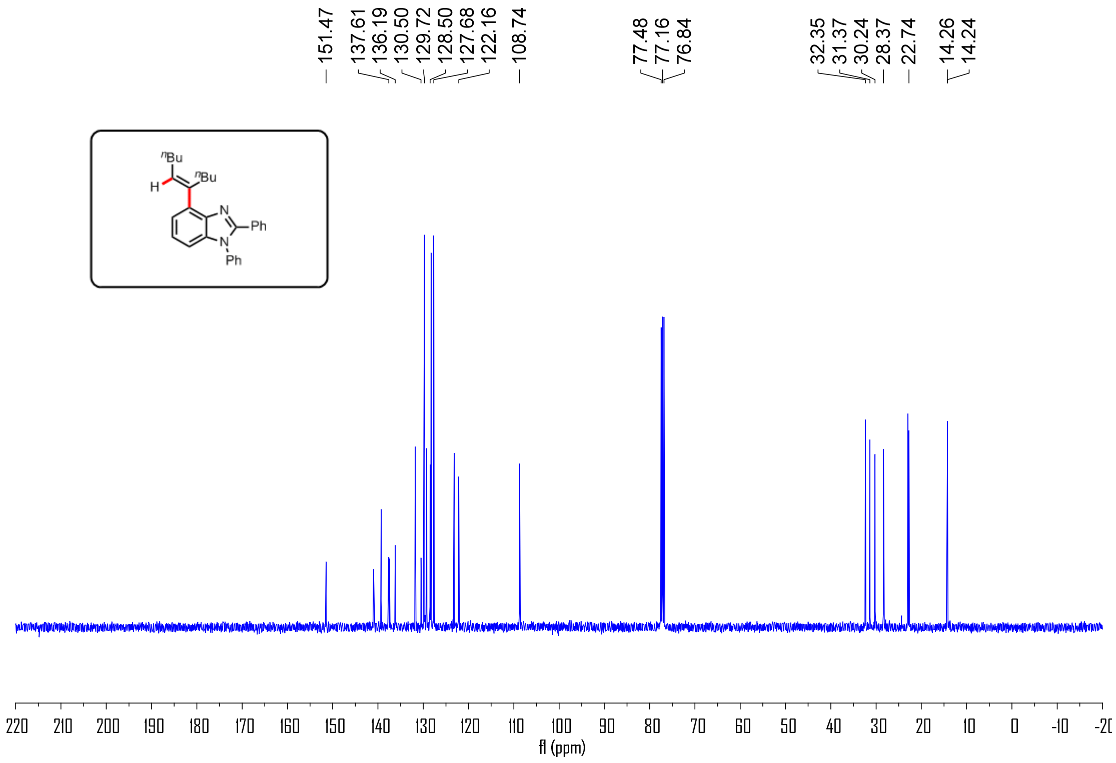

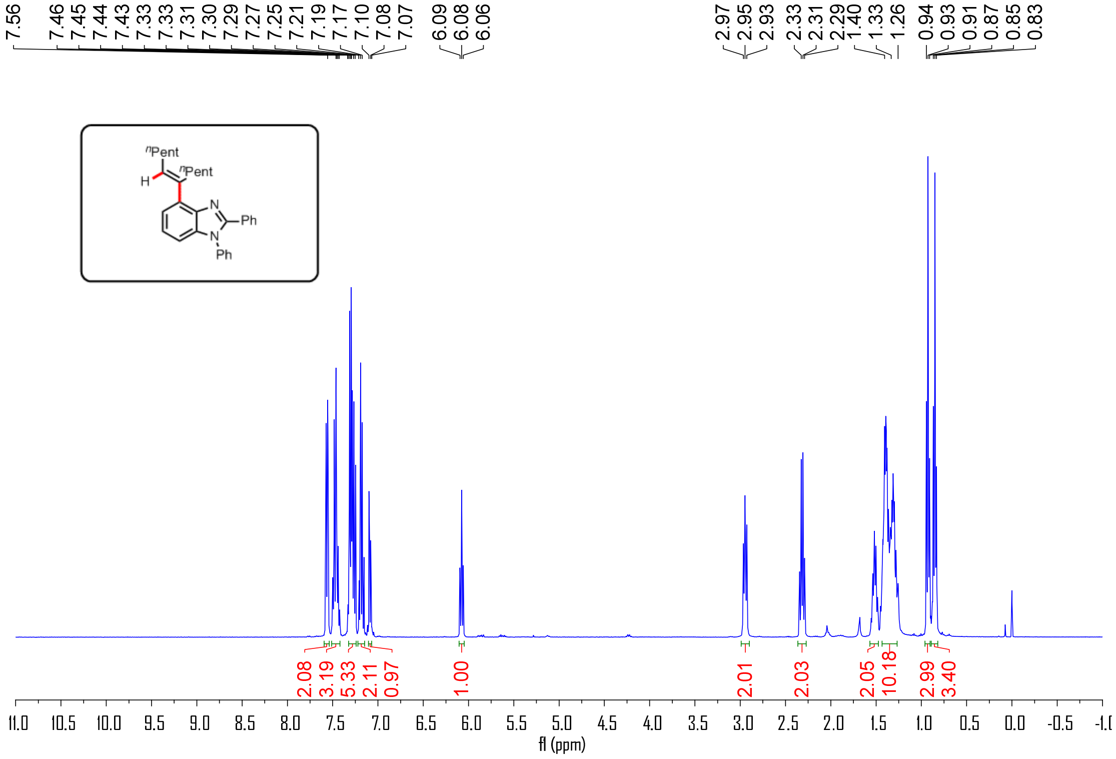

Supplementary Figure 105. ^{13}C (**5b**) and ^{1}H (**5c**) NMR spectra in $CDCl_3$.

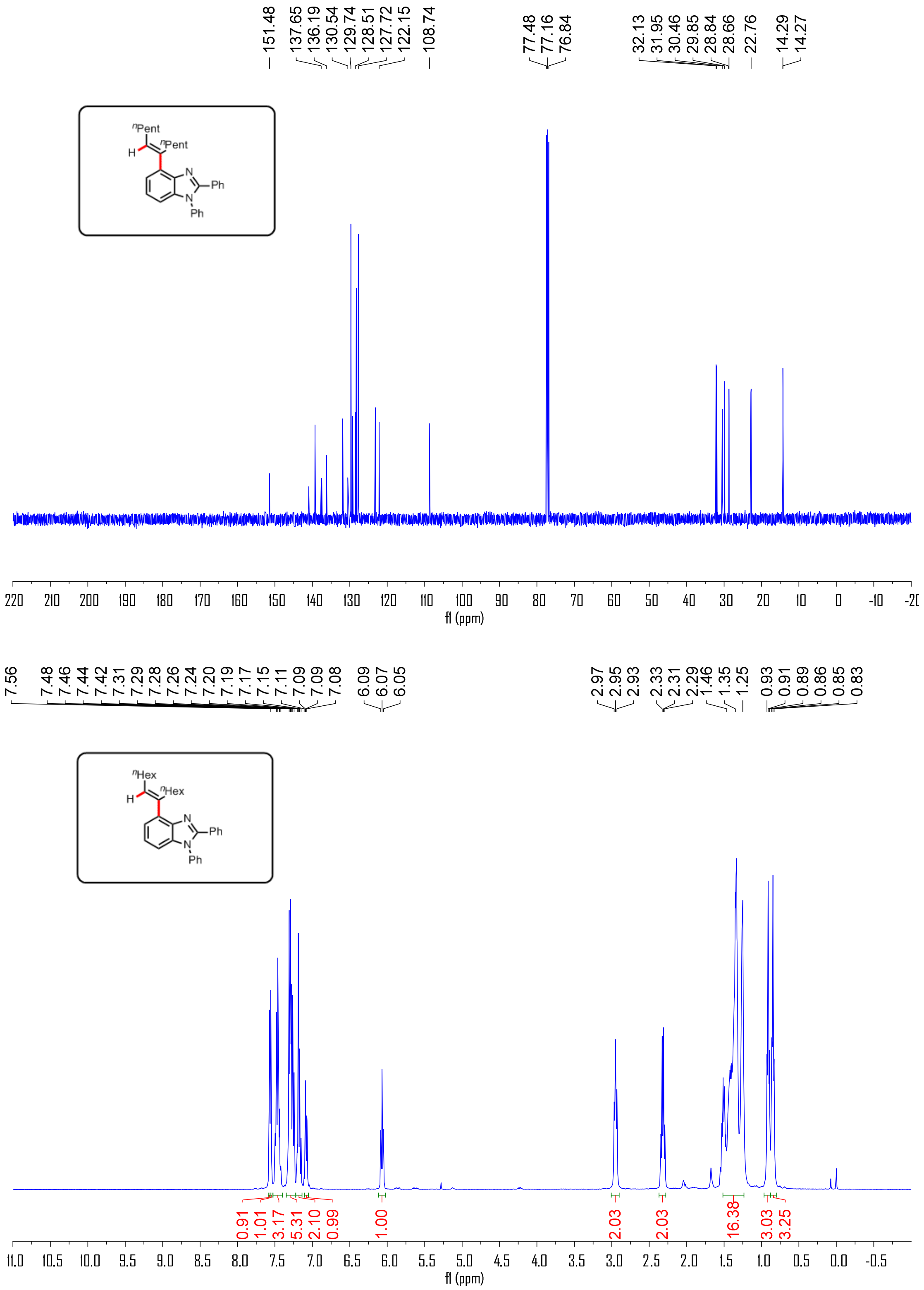

Supplementary Figure 106. ^{13}C (**5c**) and ^{1}H (**5d**) NMR spectra in $CDCl_3$.

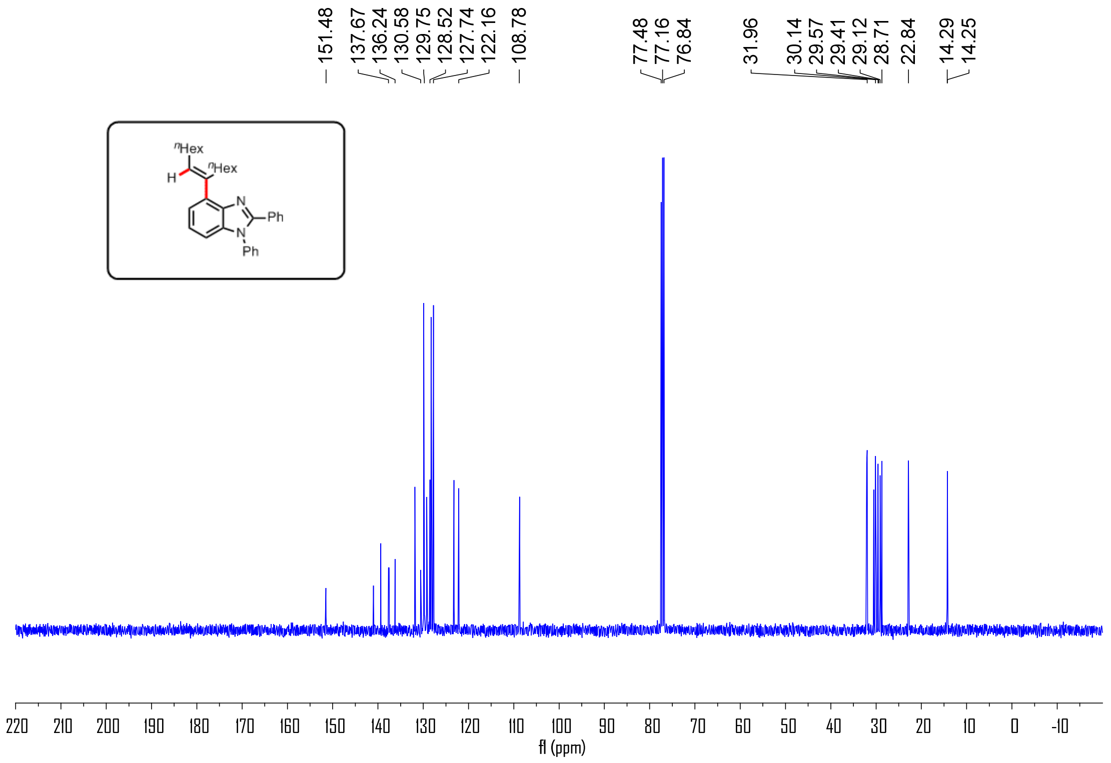

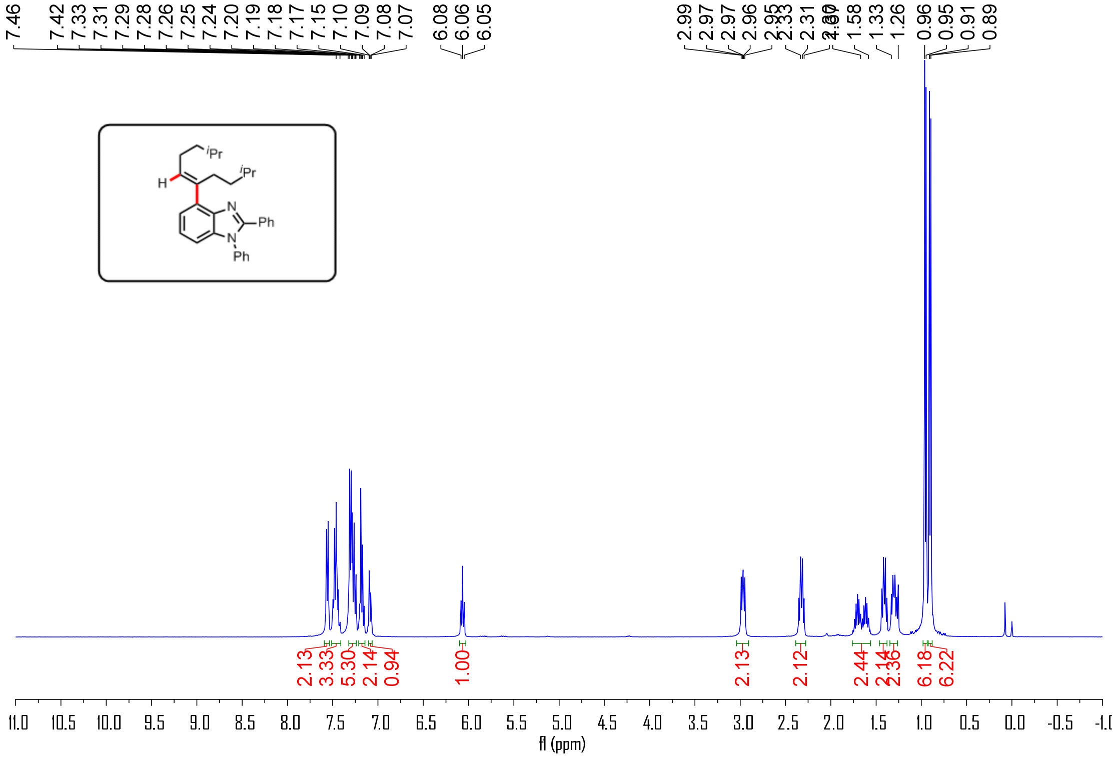

Supplementary Figure 107. ^{13}C (**5d**) and ^{1}H (**5e**) NMR spectra in $CDCl_3$.

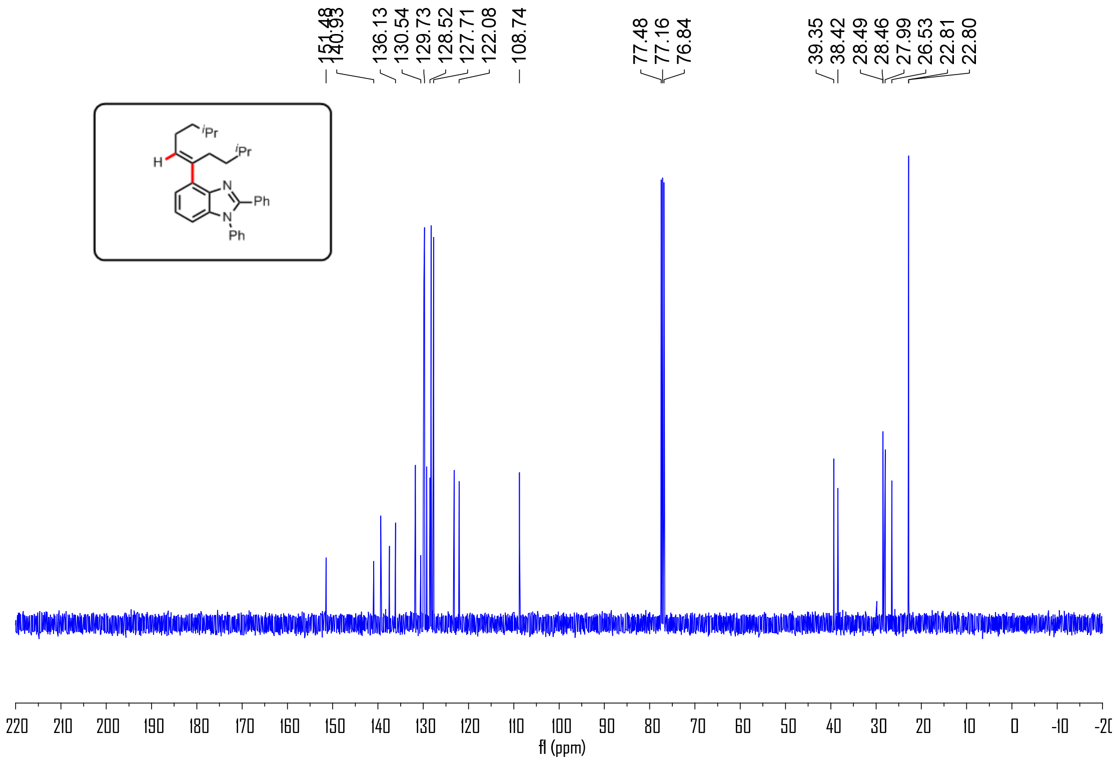

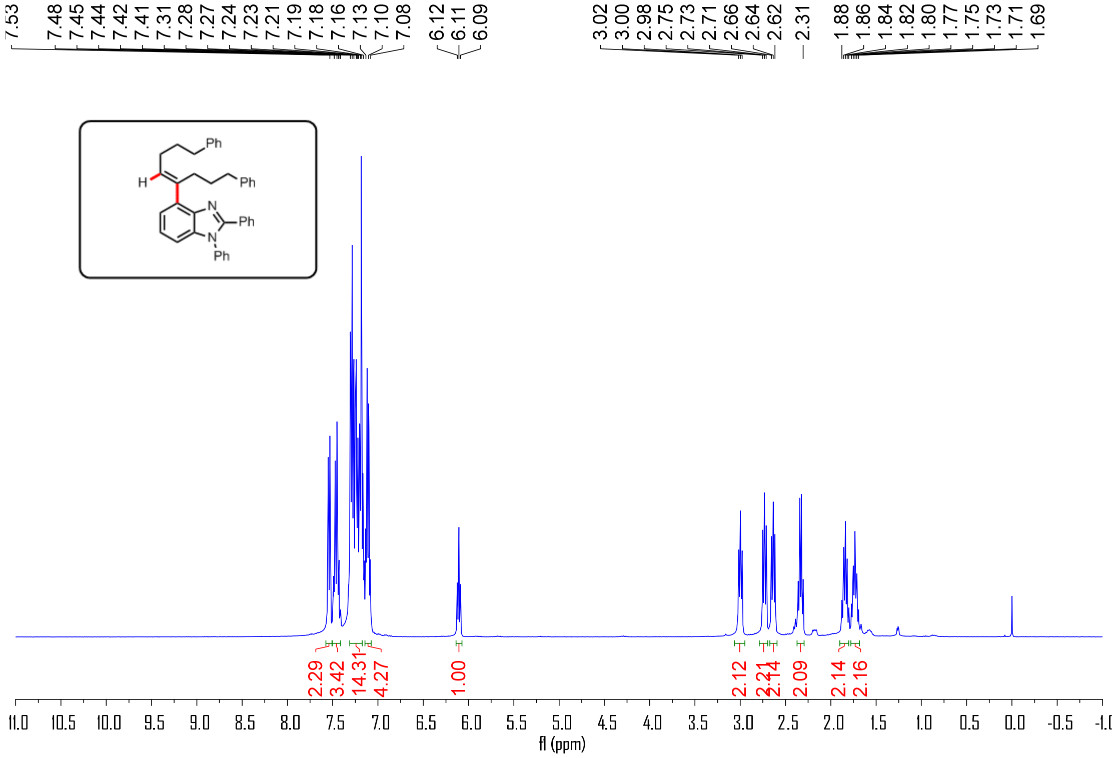

Supplementary Figure 108. ^{13}C (**5e**) and ^{1}H (**5f**) NMR spectra in $CDCl_3$.

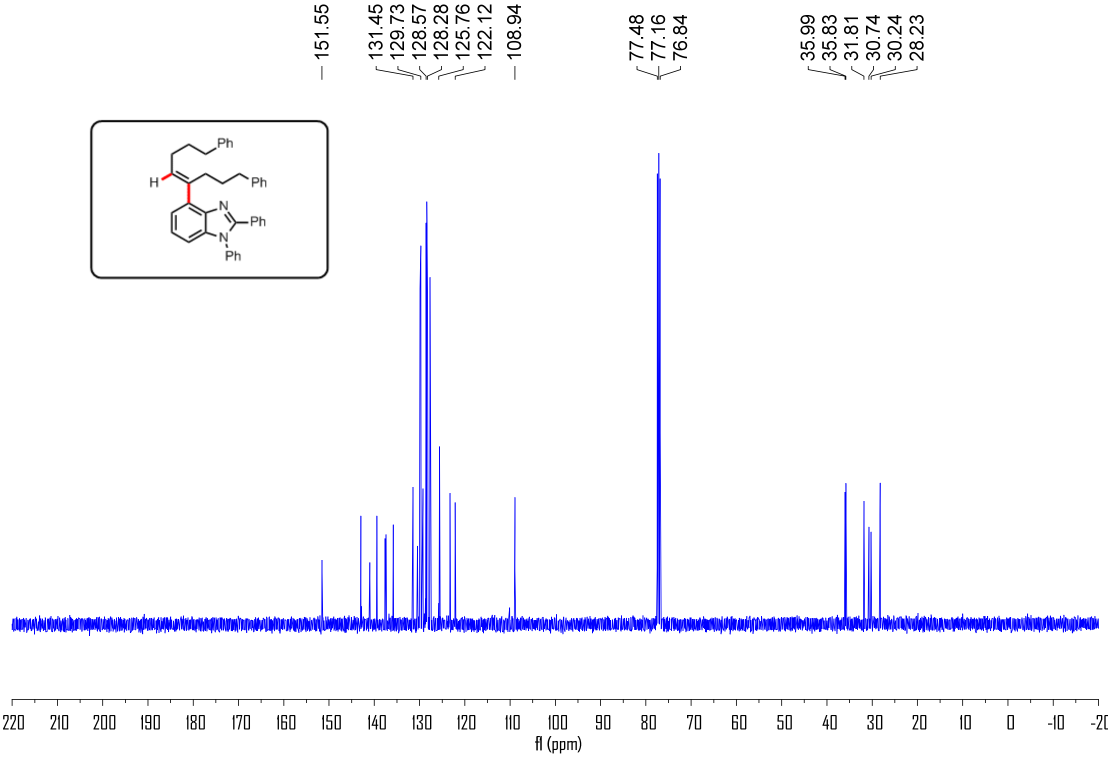

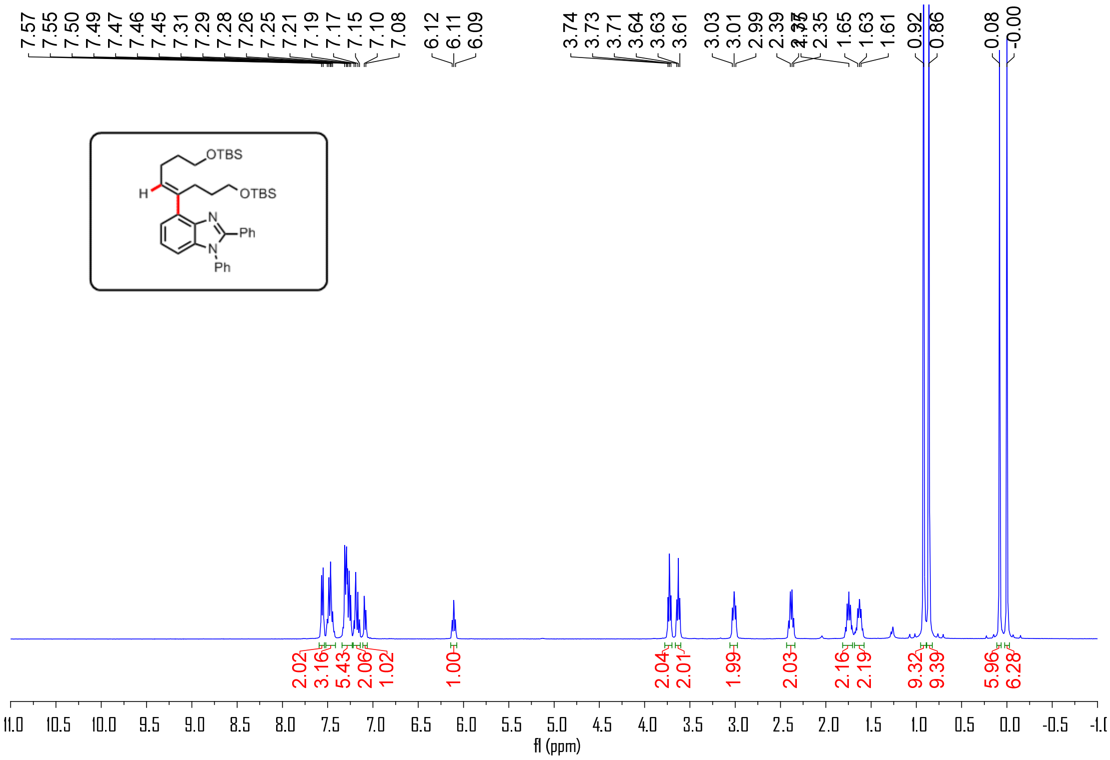

Supplementary Figure 109. ^{13}C (**5f**) and ^{1}H (**5g**) NMR spectra in $CDCl_3$.

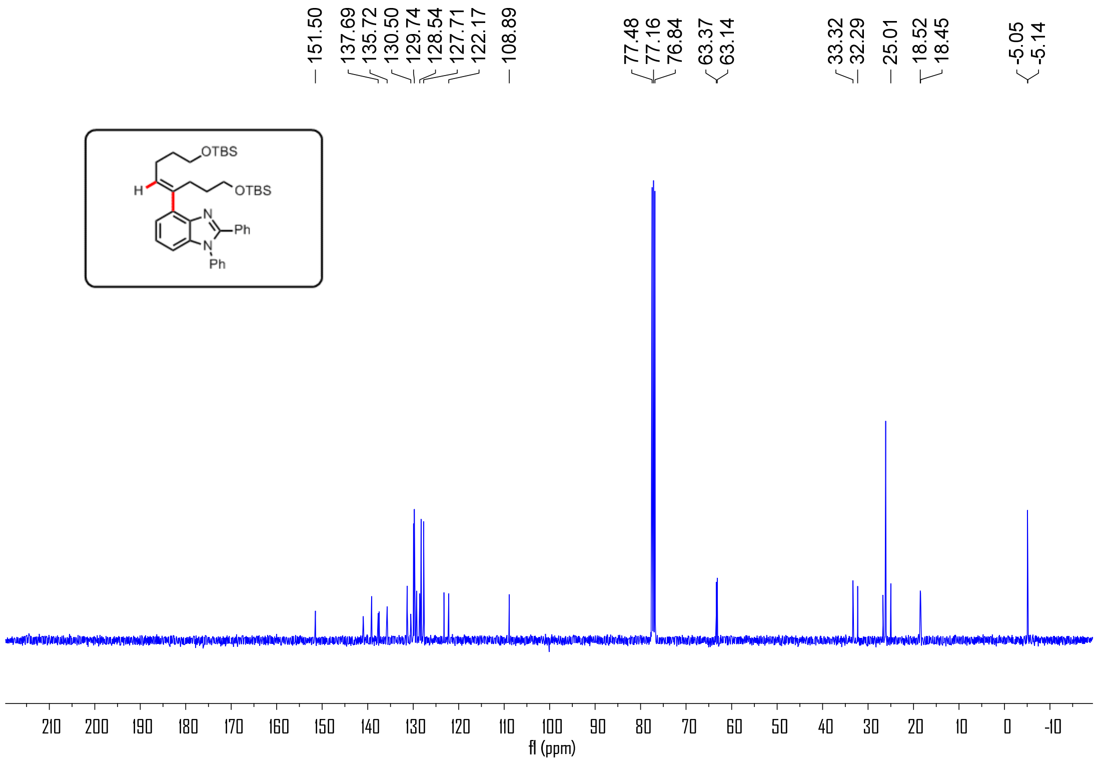

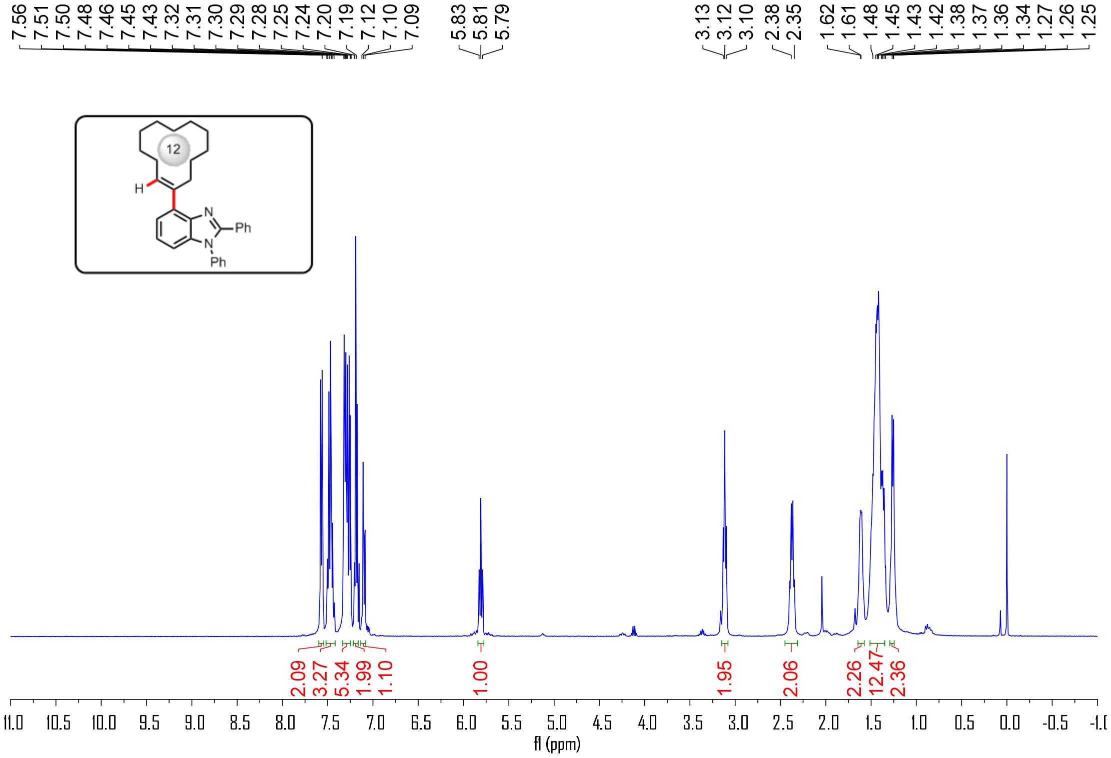

Supplementary Figure 110. ^{13}C (**5g**) and ^{1}H (**5h**) NMR spectra in $CDCl_3$.

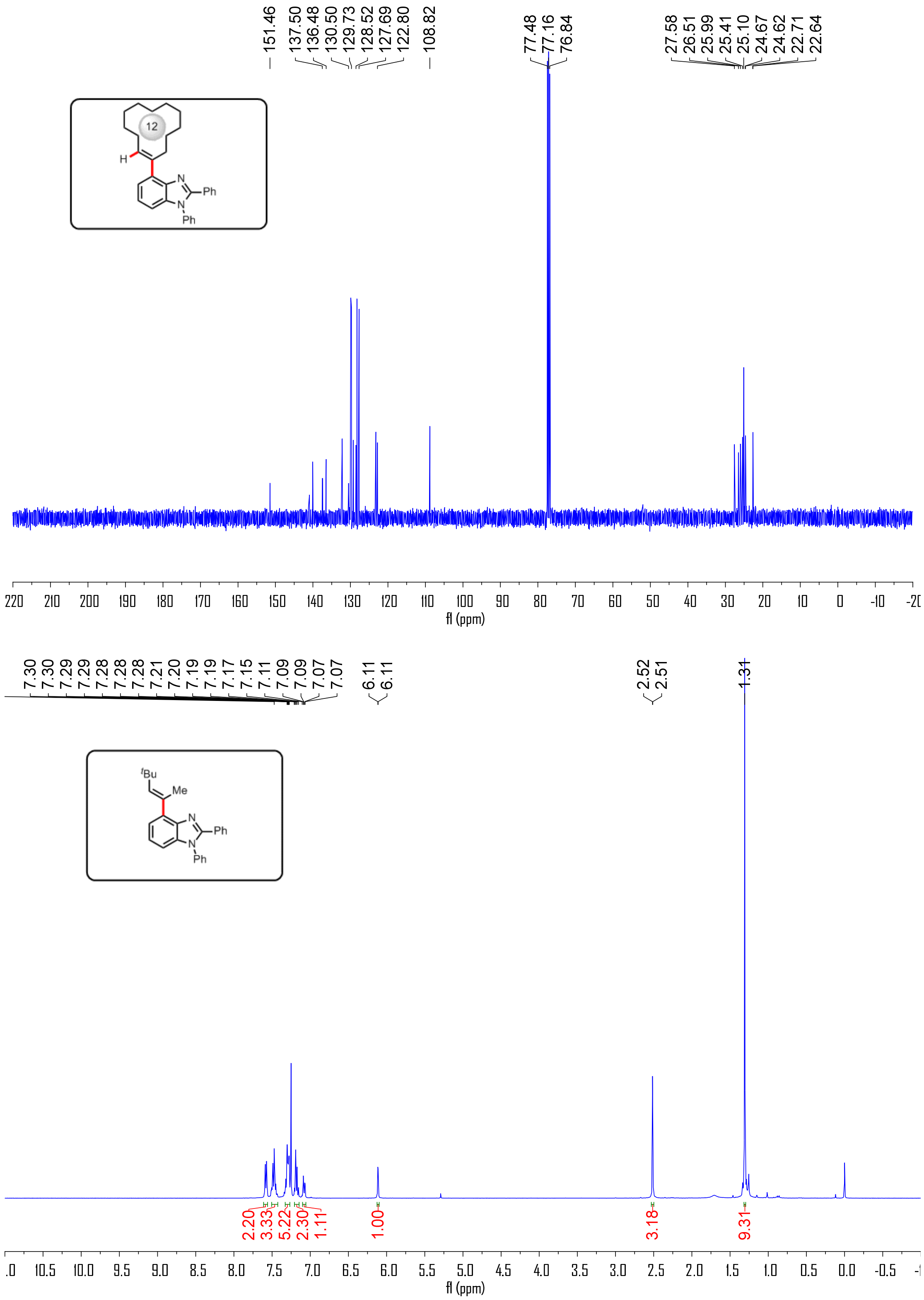

Supplementary Figure 111. ^{13}C (**5h**) and ^{1}H (**5i**) NMR spectra in $CDCl_3$.

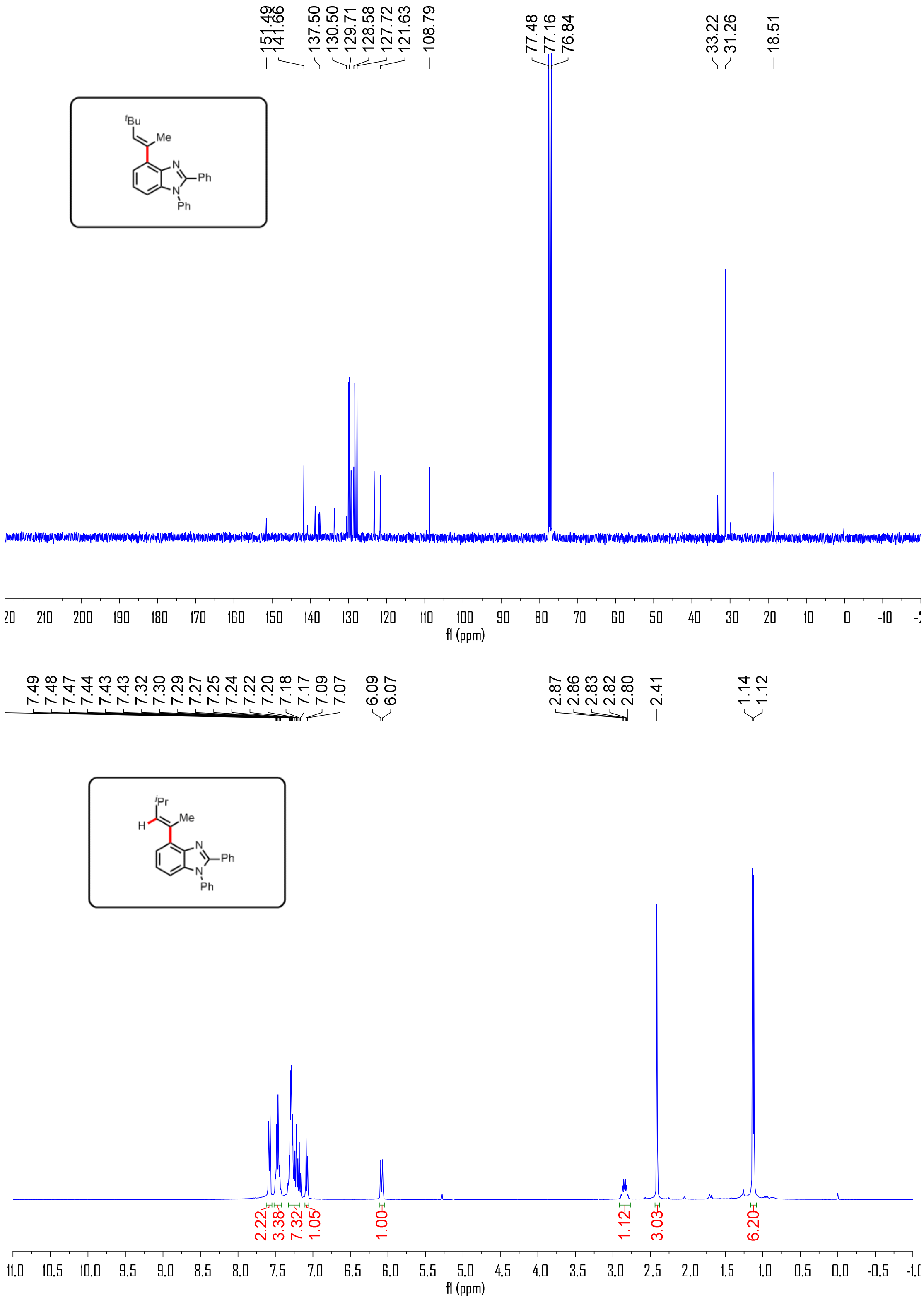

Supplementary Figure 112. ^{13}C (**5i**) and ^{1}H (**5j**) NMR spectra in $CDCl_3$.

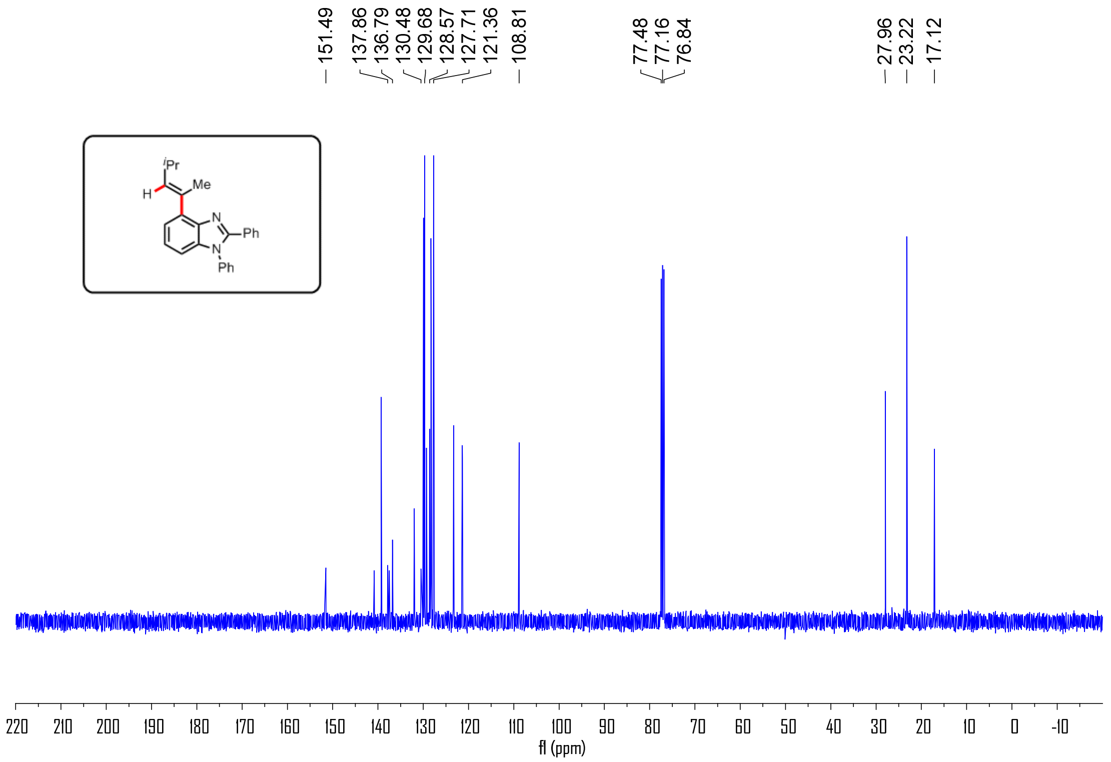

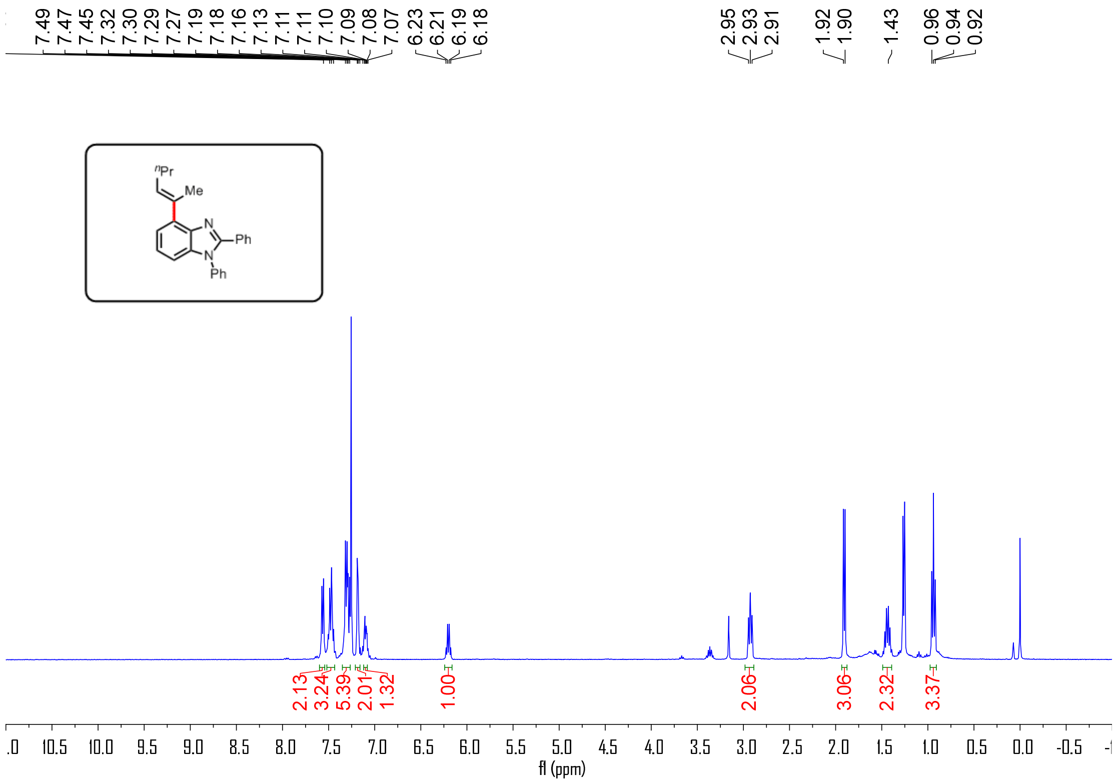

Supplementary Figure 113. ^{13}C (**5j**) and ^{1}H (**5k**) NMR spectra in $CDCl_3$.

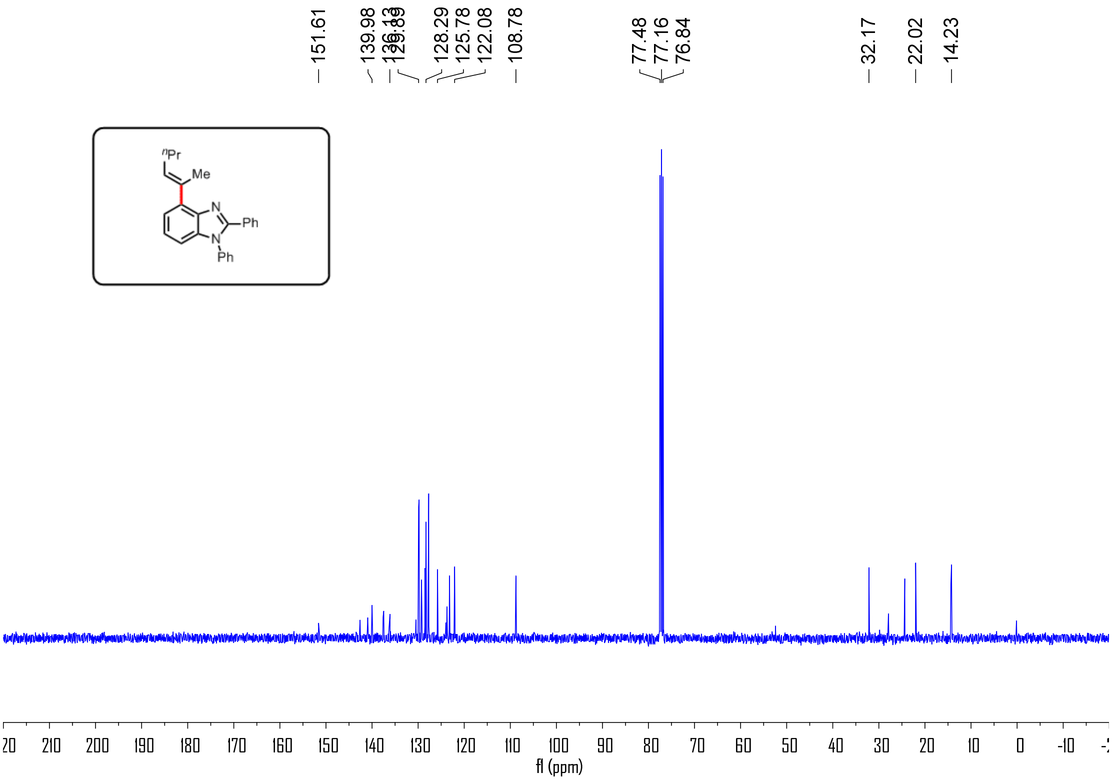

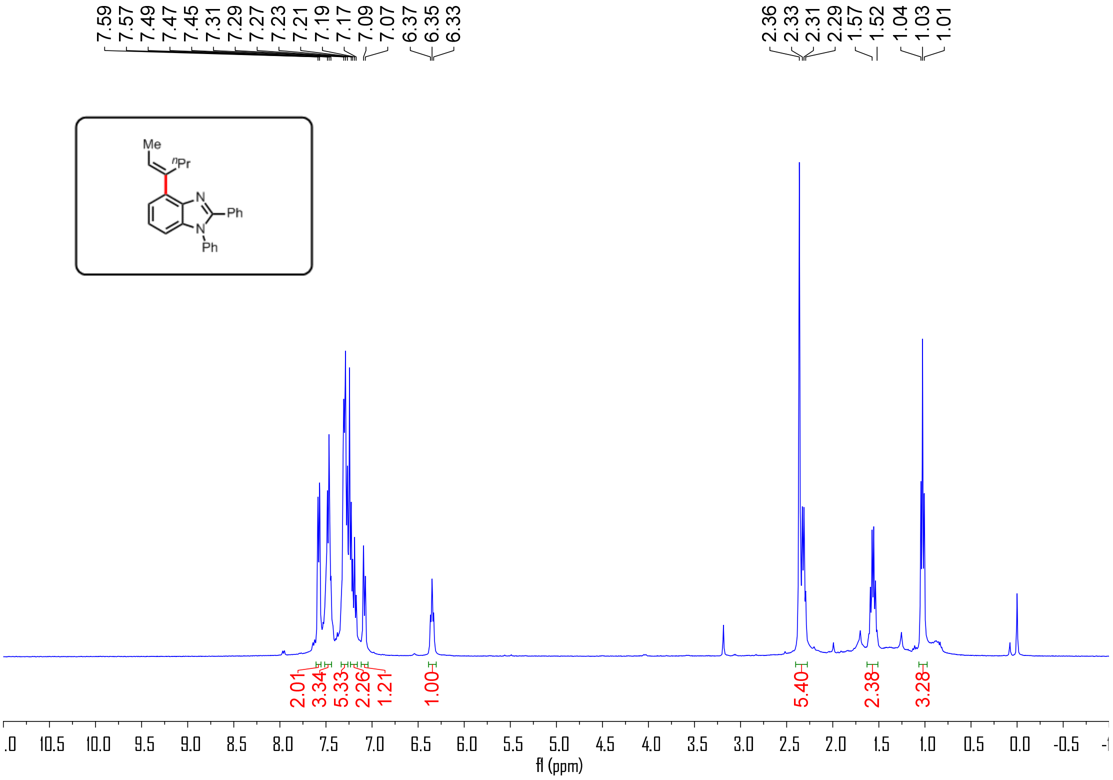

Supplementary Figure 114. ^{13}C (**5k**) and ^{1}H (**5k′**) NMR spectra in $CDCl_3$.

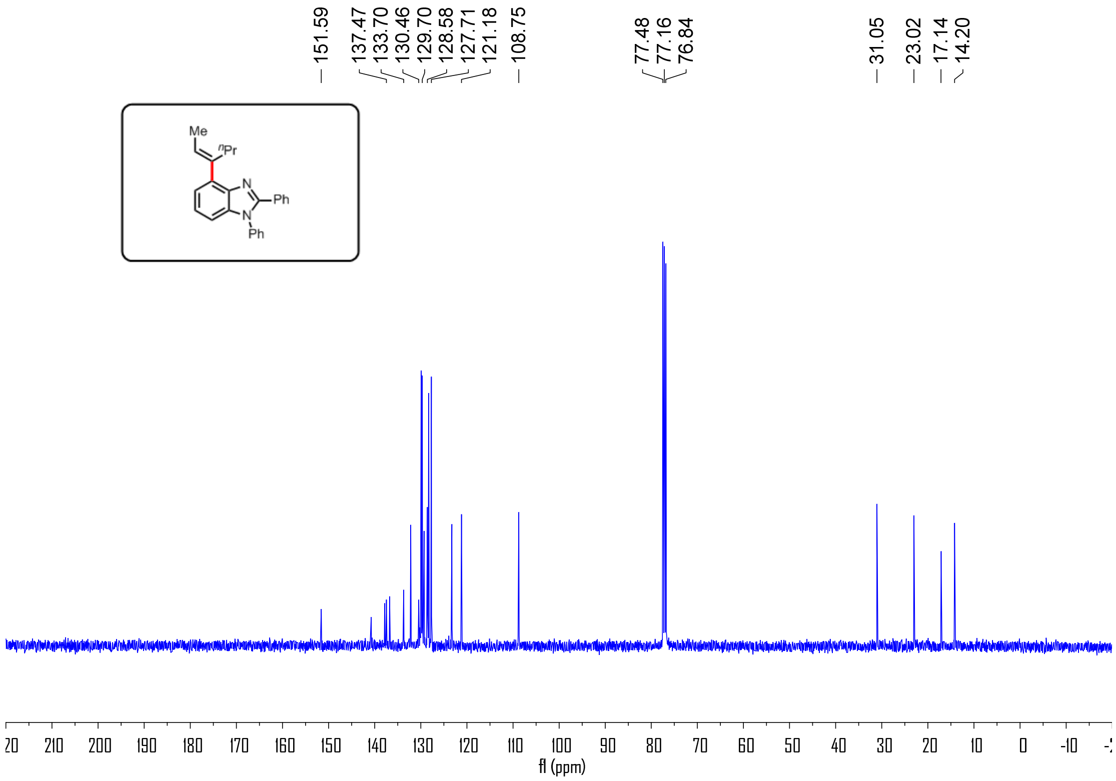

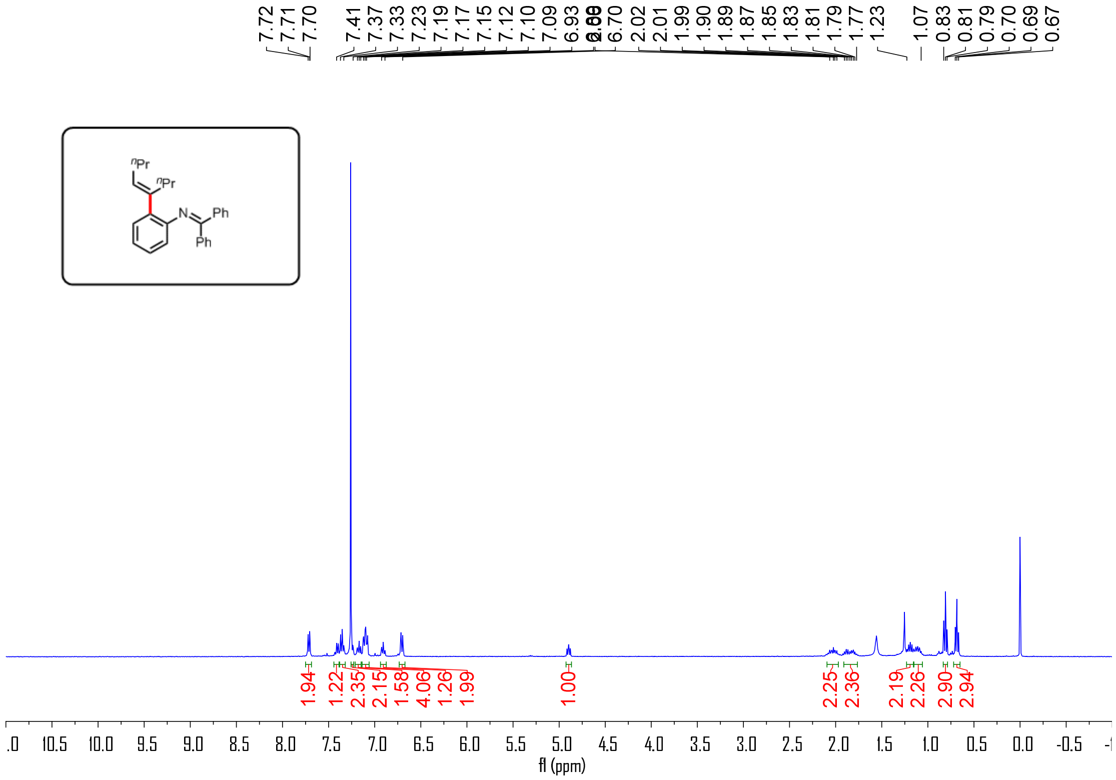

Supplementary Figure 115. ^{13}C (**5k′**) and ^{1}H (**imine**) NMR spectra in $CDCl_3$.

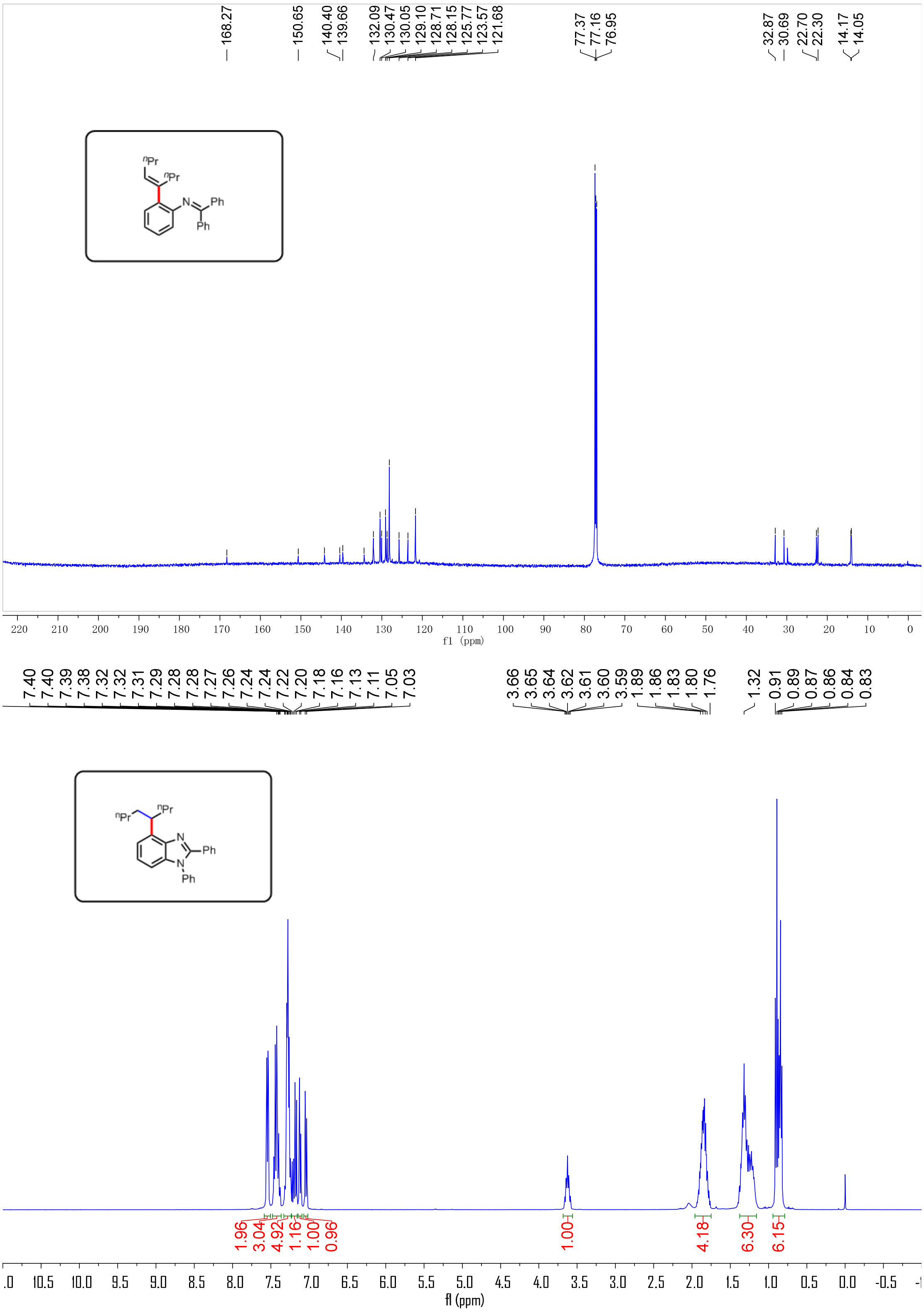

Supplementary Figure 116. ^{13}C (**imine**) and ^{1}H (**6**) NMR spectra in $CDCl_3$.

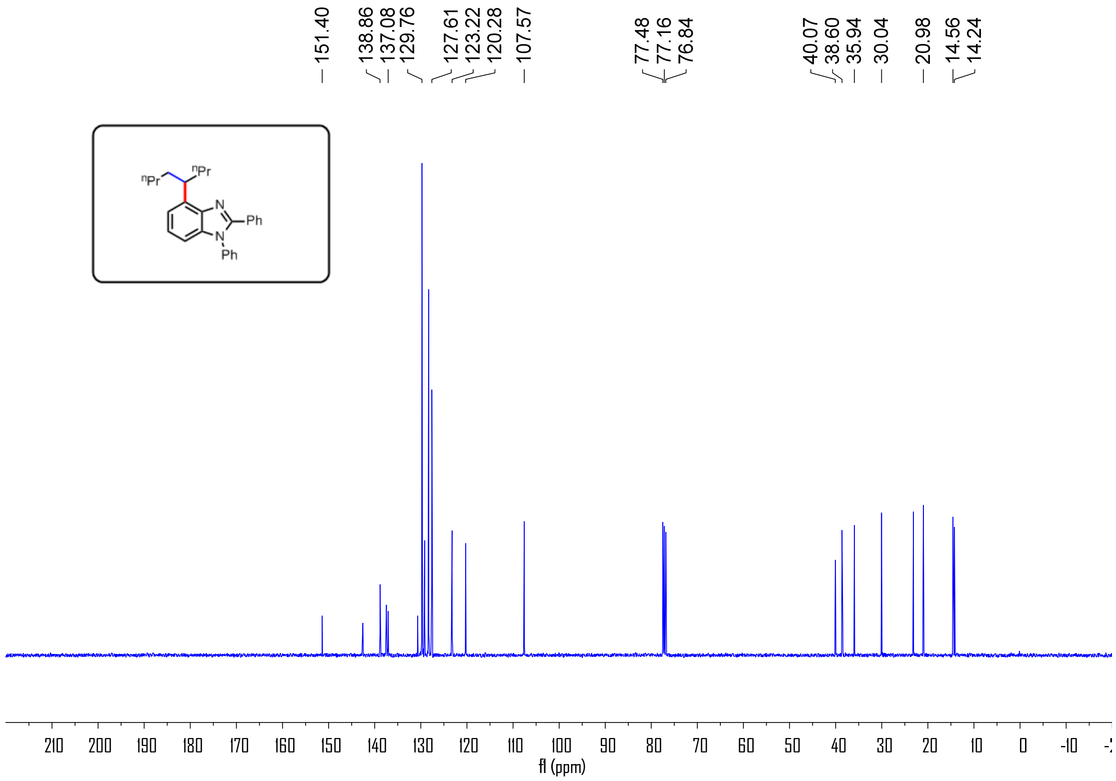

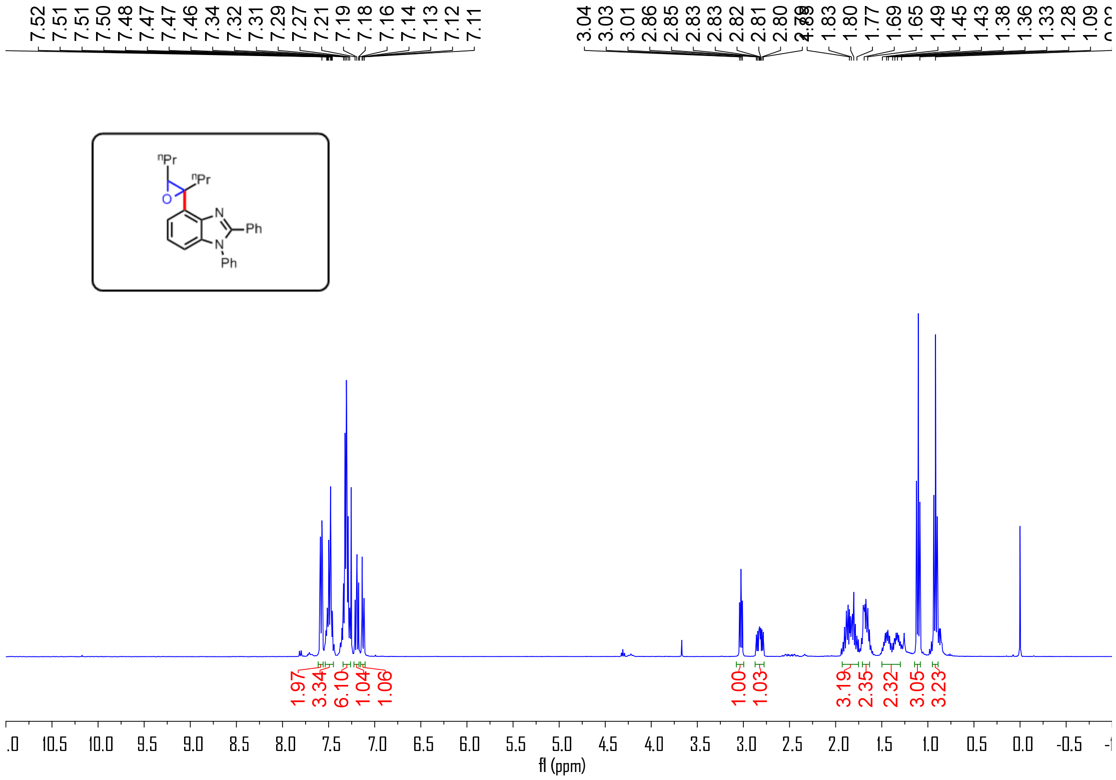

Supplementary Figure 117. ^{13}C (**6**) and ^{1}H (**7**) NMR spectra in $CDCl_3$.

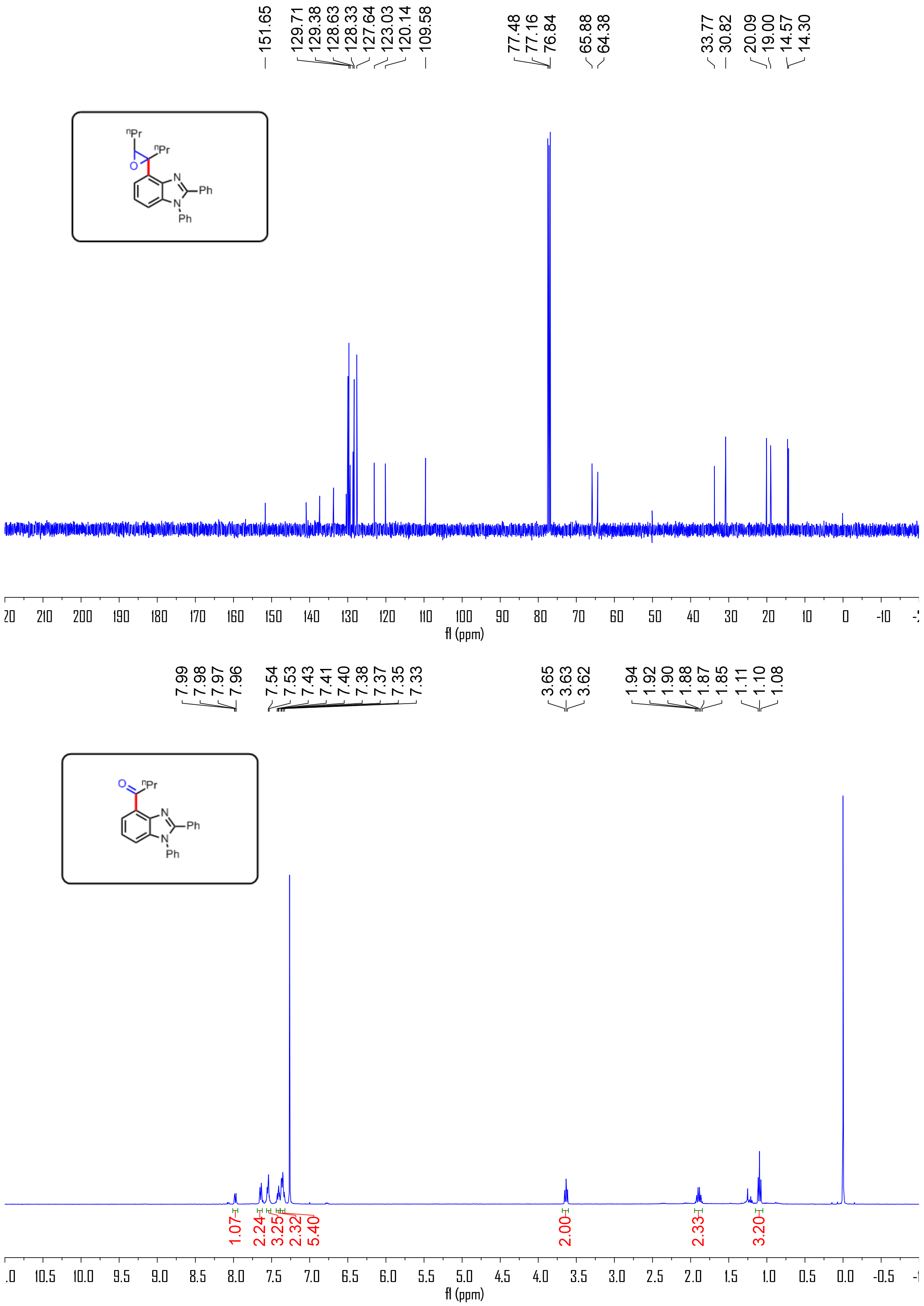

Supplementary Figure 118. ^{13}C (**7**) and ^{1}H (**8**) NMR spectra in $CDCl_3$.

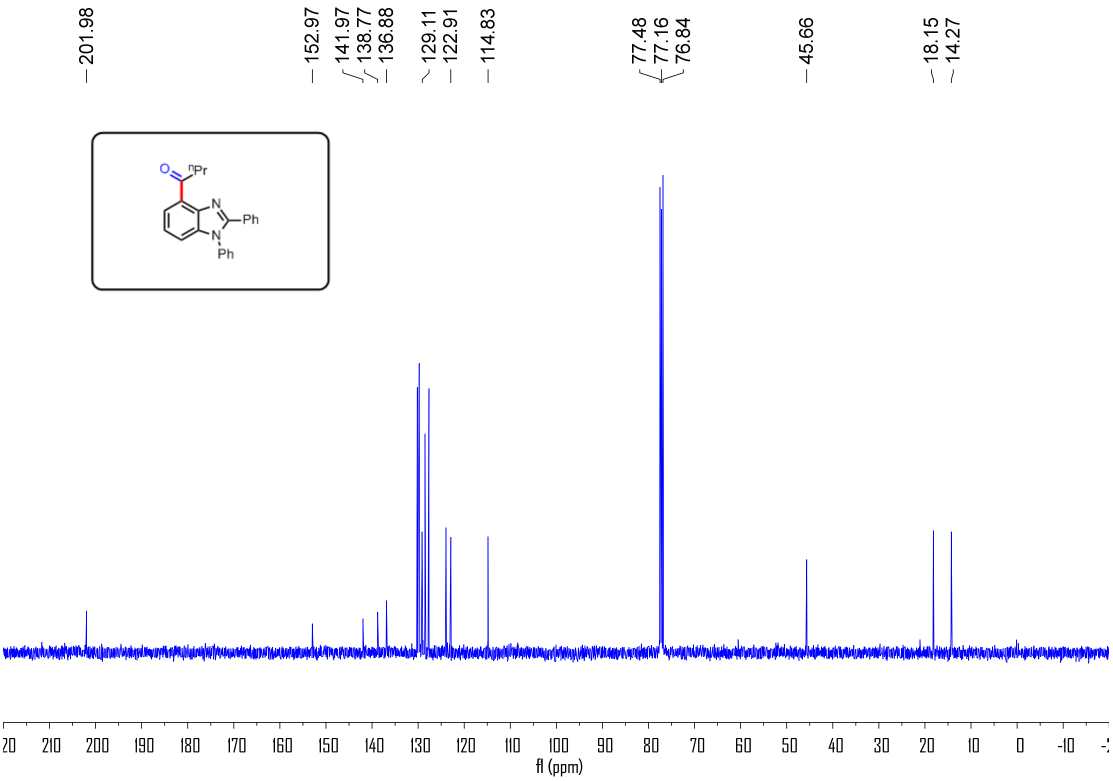

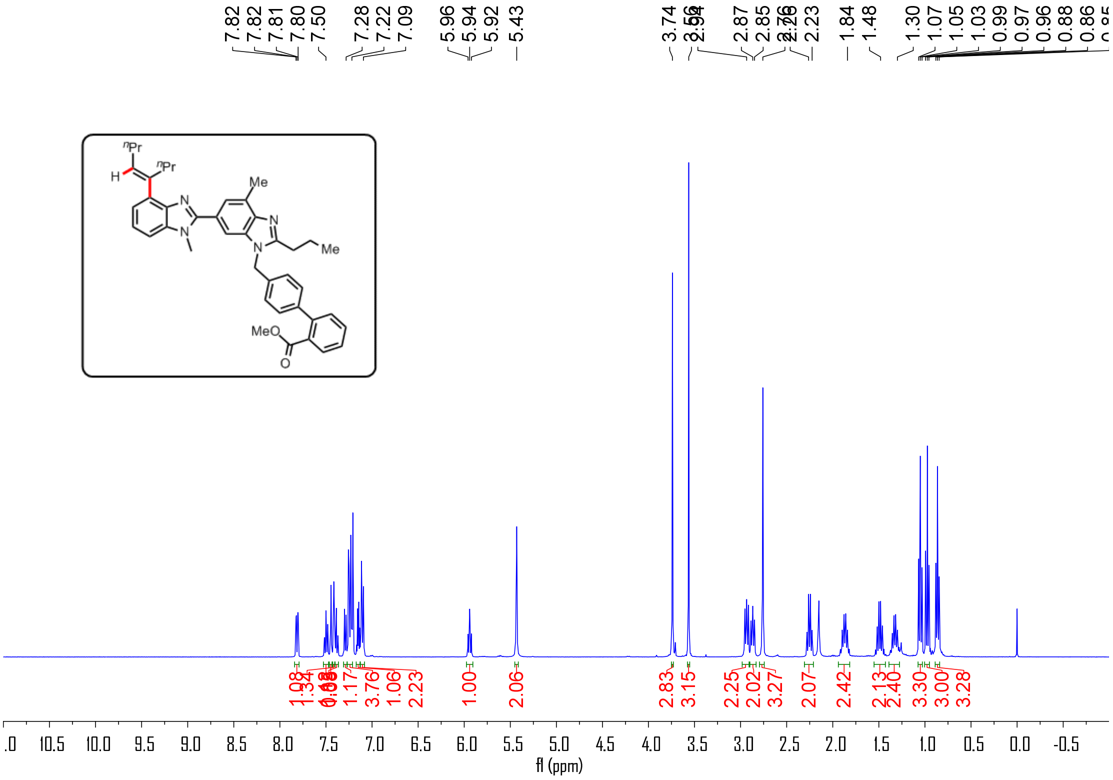

Supplementary Figure 119. ^{13}C (**8**) and ^{1}H (**9**) NMR spectra in $CDCl_3$.

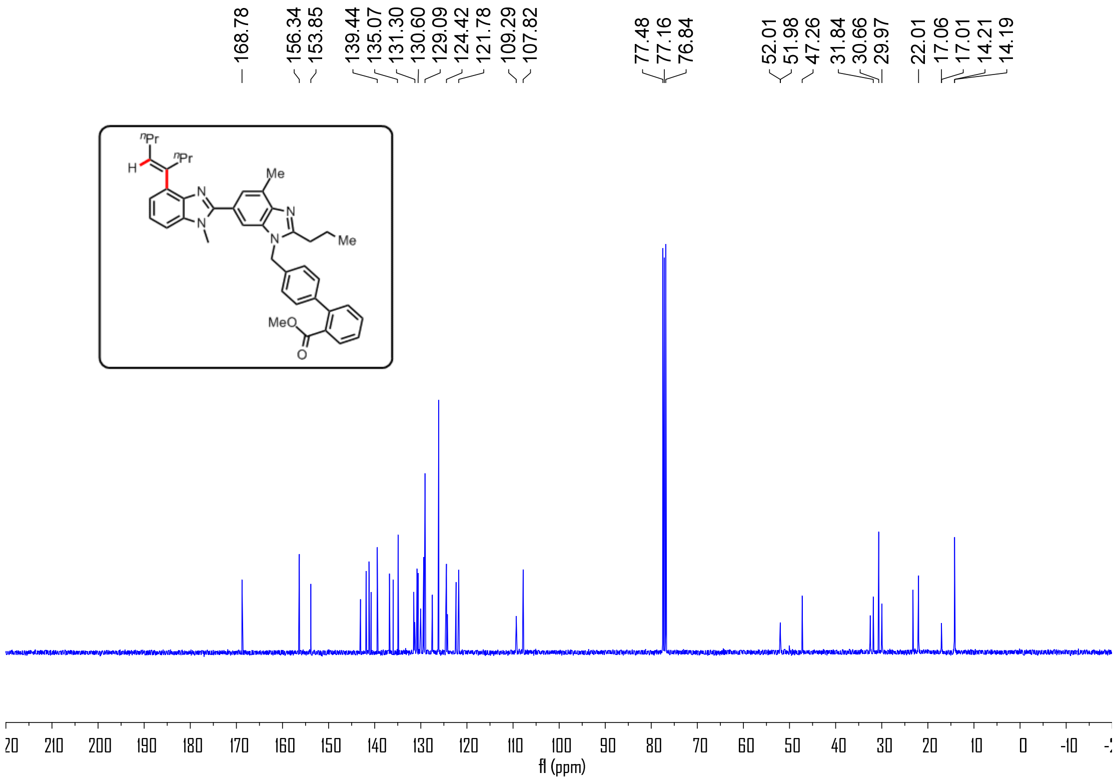

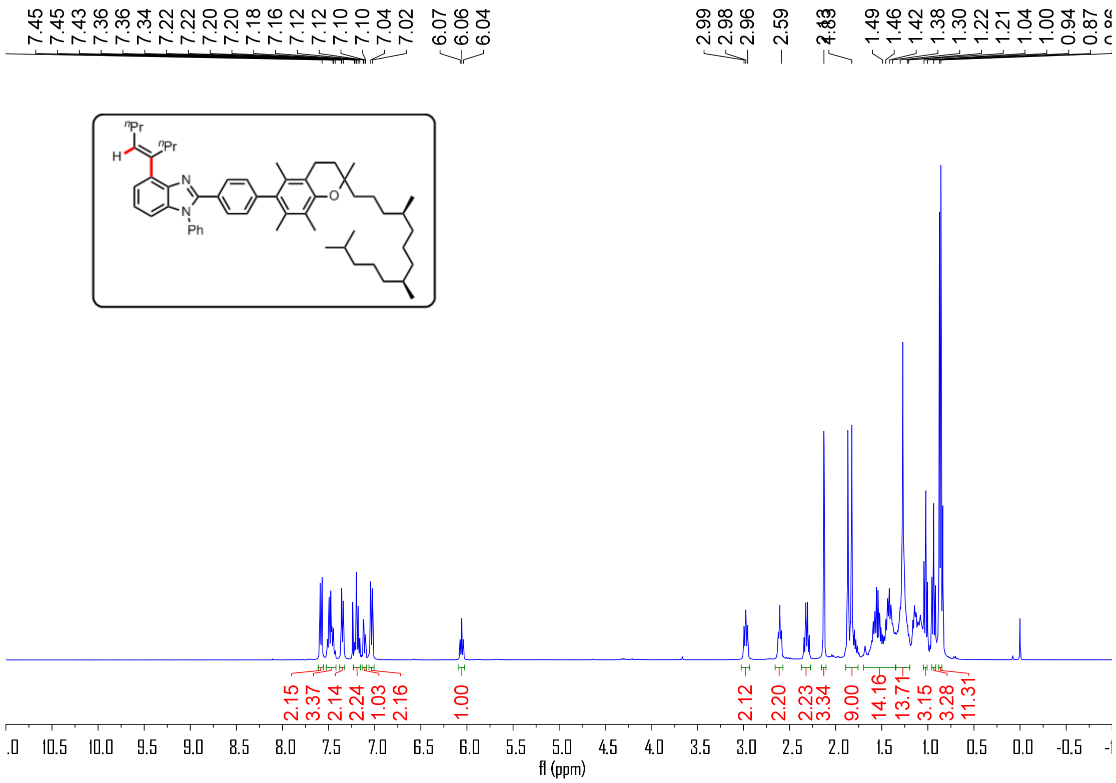

Supplementary Figure 120. ^{13}C (**9**) and ^{1}H (**10**) NMR spectra in $CDCl_3$.

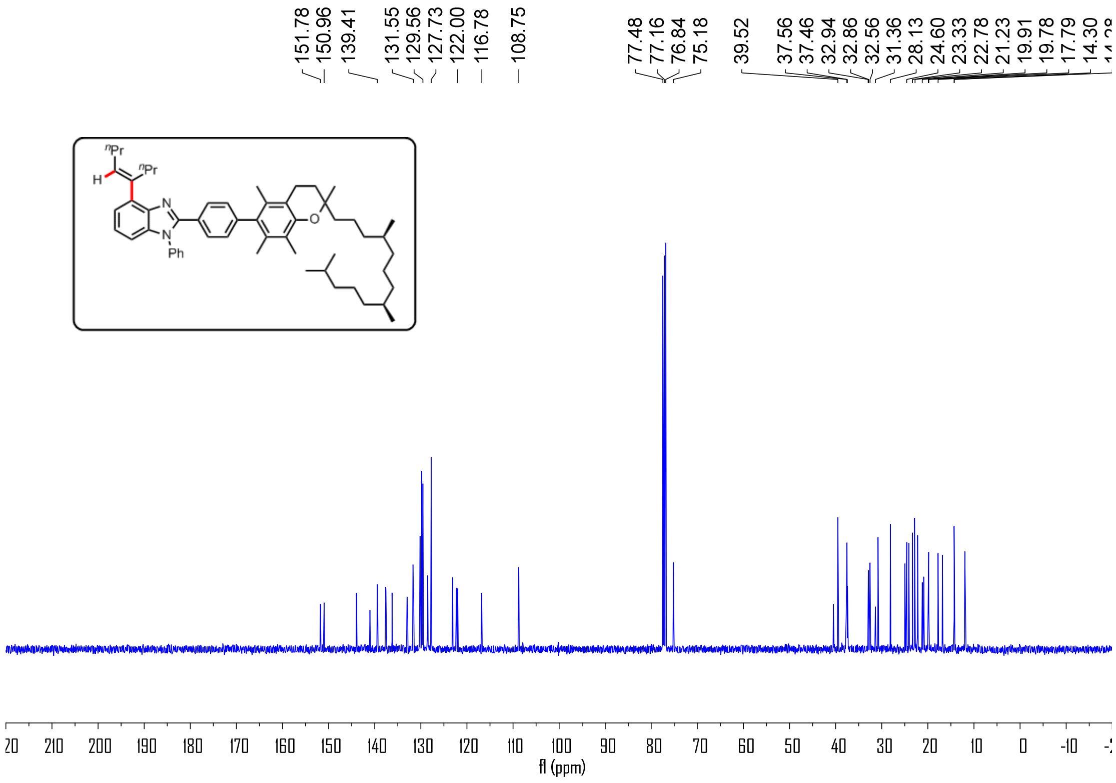

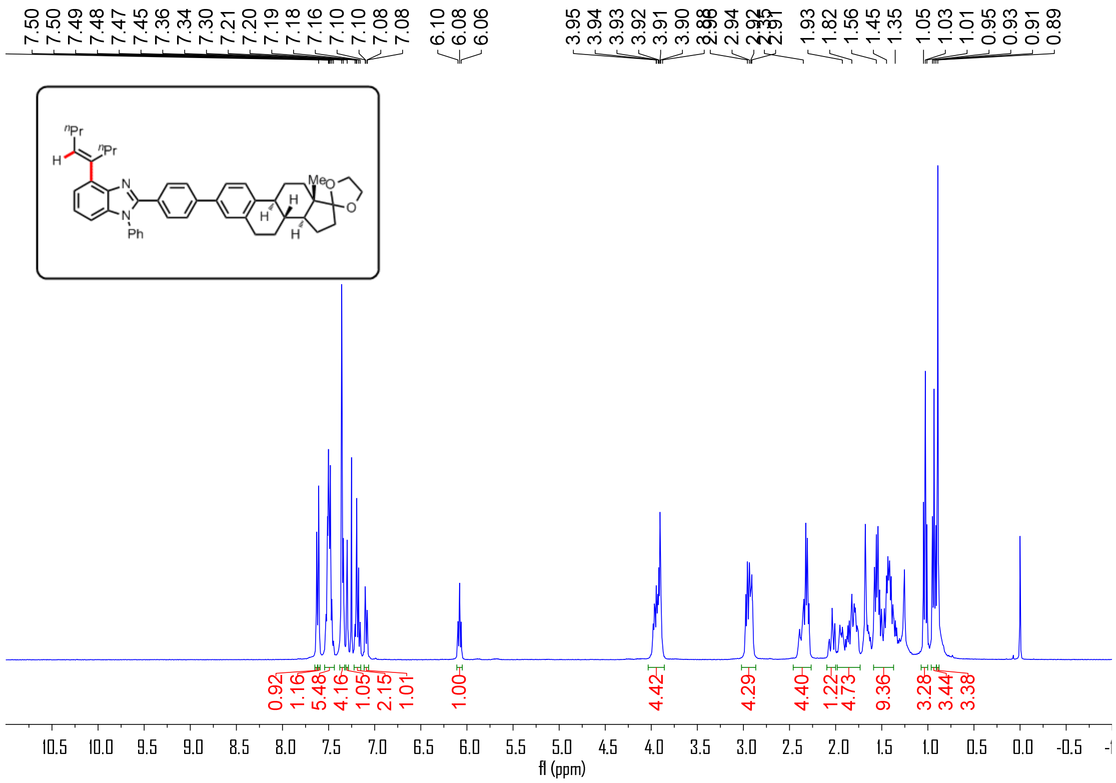

Supplementary Figure 121. ^{13}C (**10**) and ^{1}H (**11**) NMR spectra in $CDCl_3$.

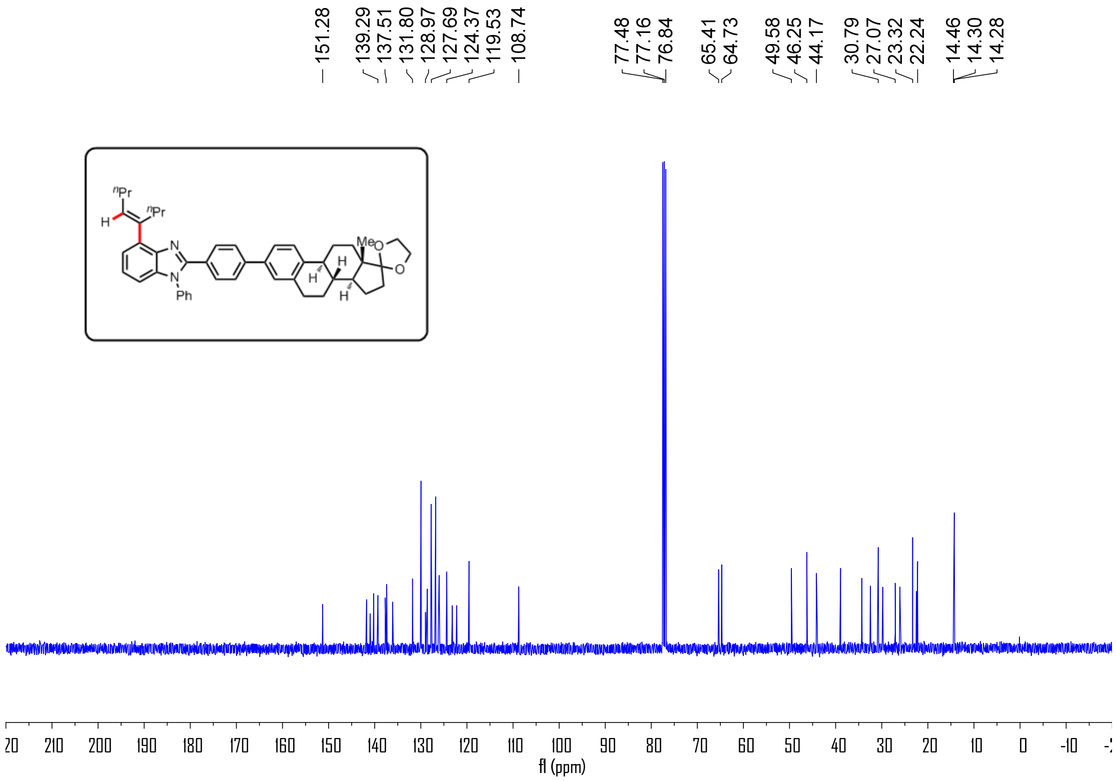

Supplementary Figure 122. ^{13}C NMR spectrum of **11** in $CDCl_3$.

Supplementary References

1 Lim, Y.-G., Lee, K.-H., Koo, B. T. & Kang, J.-B. Rhodium(I)-catalyzed *ortho*-alkenylation of 2-phenylpyridines with alkynes. *Tetrahedron Lett.* **42**, 7609−7612 (2001).

2 Hashimoto, Y., Hirano, K., Satoh, T., Kakiuchi, F. & Miura, M. Regioselective C−H Bond Cleavage/Alkyne Insertion under Ruthenium Catalysis. *J. Org. Chem.* **78**, 638−646 (2013).

3 Wang, Y.-X. et al. Enantioselective Ni−Al Bimetallic Catalyzed *exo*-Selective C−H Cyclization of Imidazoles with Alkenes. *J. Am. Chem. Soc.* **140**, 5360–5364 (2018).

4 Chen, H., Wang, Y.-X., Luan, Y.-X. & Ye, M. Enantioselective Twofold C−H Annulation of Formamides and Alkynes without Built-in Chelating Groups. *Angew. Chem. Int. Ed.* **59**, 9428–9432 (2020).
